# Supplementary material for: Fitting Contralateral Neuroanatomical Asymmetry into the Amyloid Cascade Hypothesis
Source: Healthcare (Basel). 2022 Aug 29;10(9):1643. doi: 10.3390/healthcare10091643 (PMC9498691; doi:10.3390/healthcare10091643)
Supplement: Supplementary file 1 [file healthcare-10-01643-s001.zip › healthcare-1868262-supplementary.pdf]

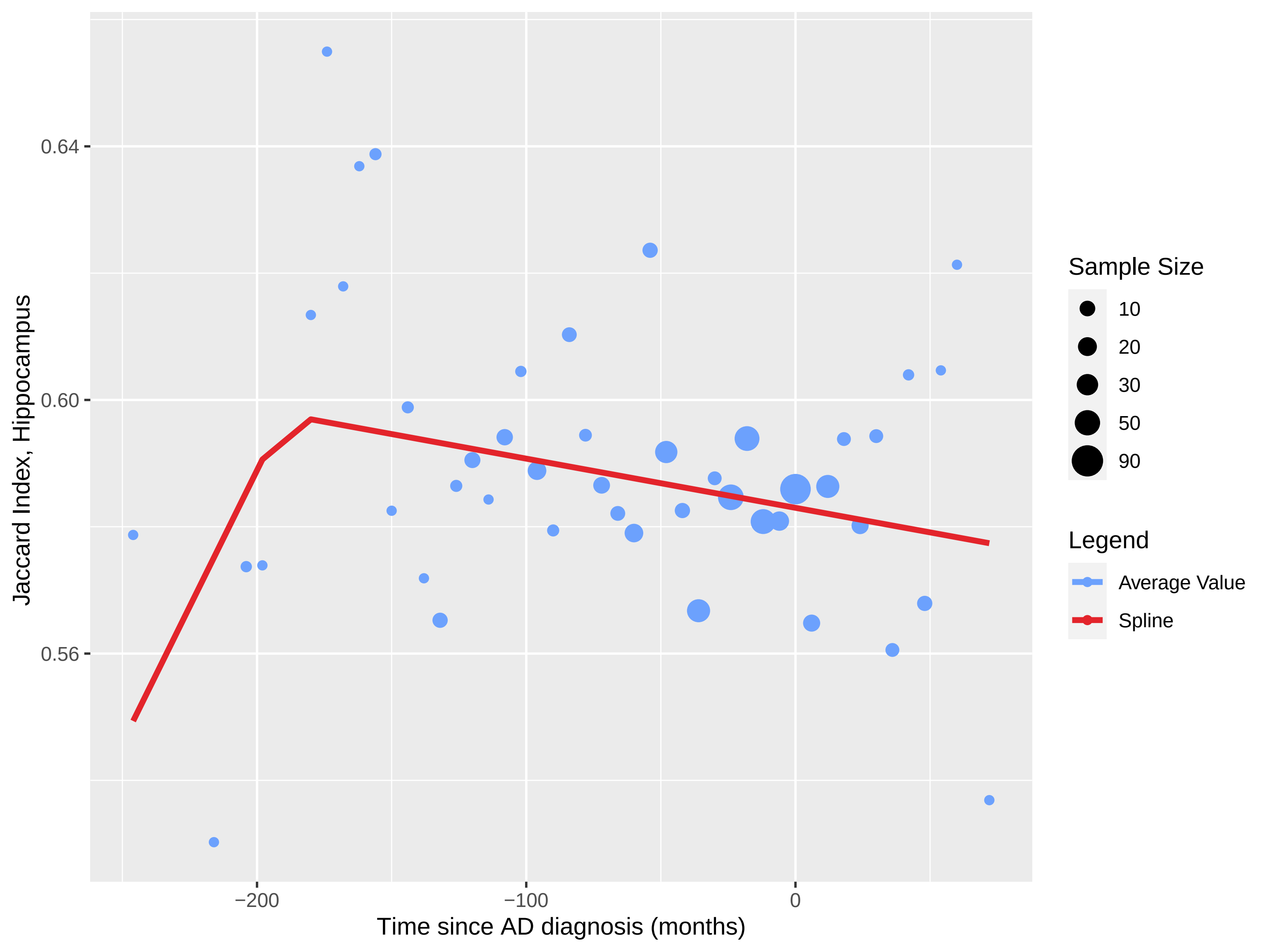

0.64
0.60
0.56
−200
−100
0
Time since AD diagnosis (months)
Jaccard Index, Hippocampus
Sample Size
10
20
30
50
90
Legend
Average Value
Spline

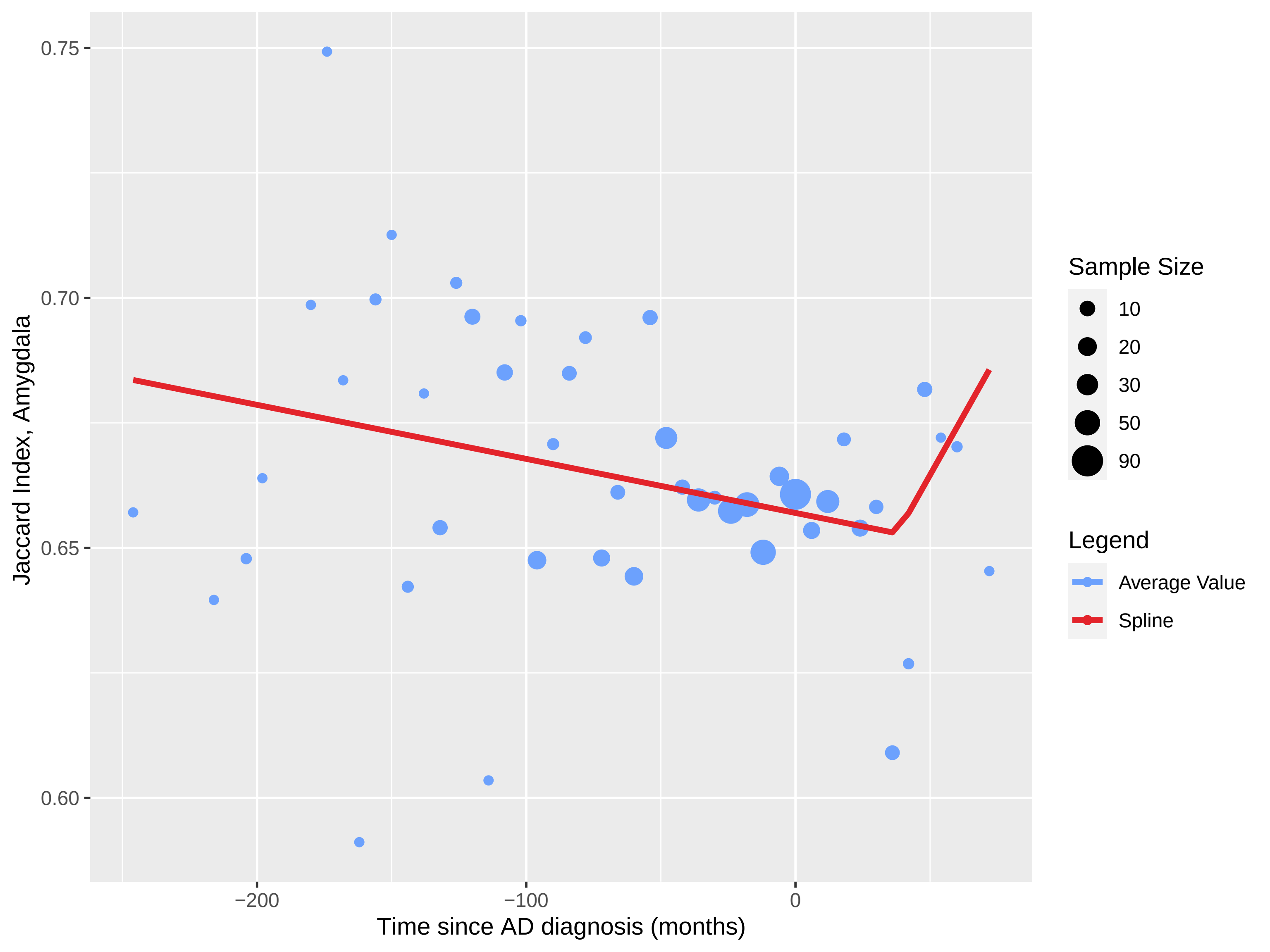

0.75
0.70
0.65
0.60
Jaccard Index, Amygdala
−200
−100
0
Time since AD diagnosis (months)
Sample Size
10
20
30
50
90
Legend
Average Value
Spline

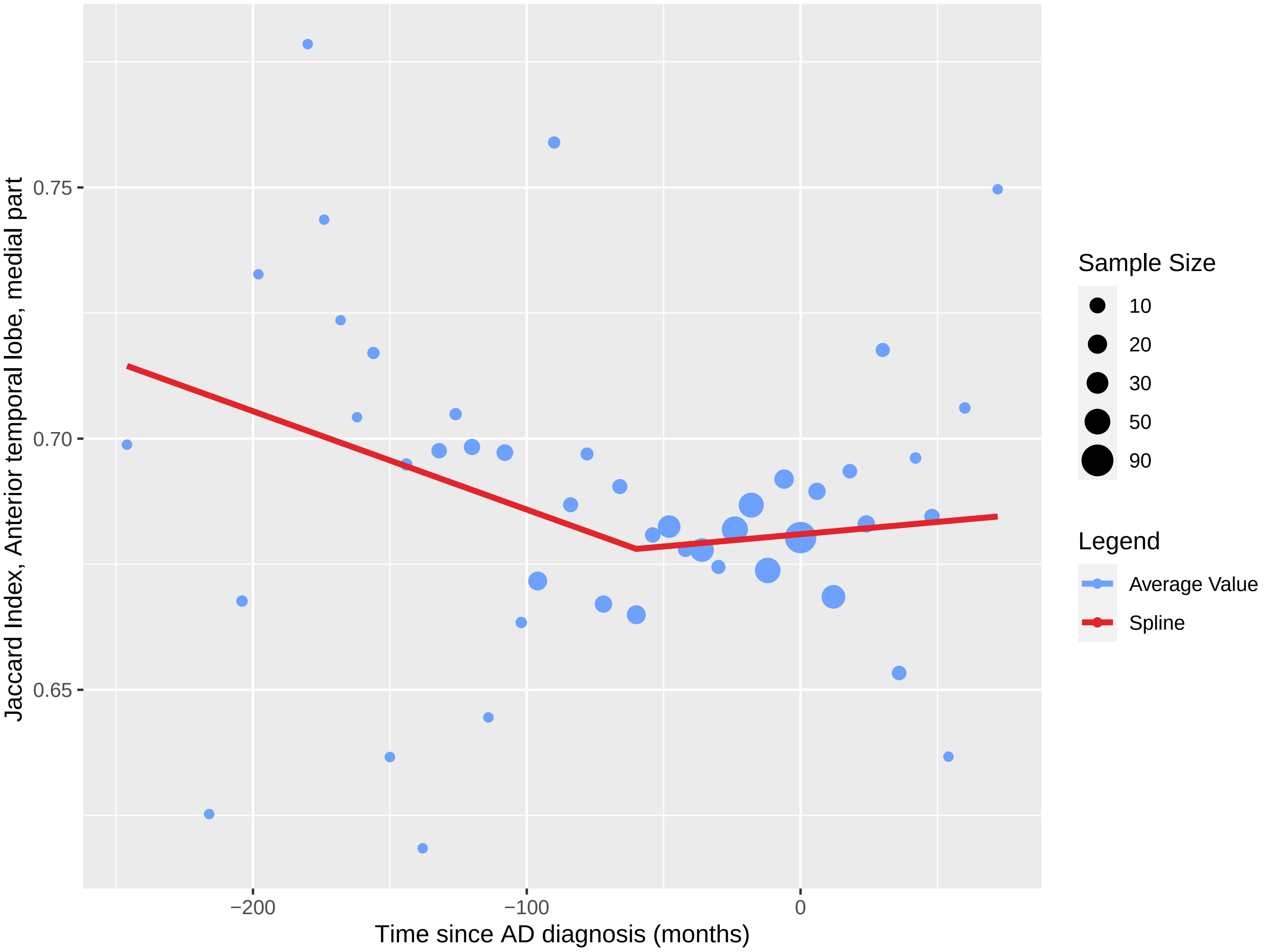

0.75
0.70
0.65
−200
−100
0
Jaccard Index, Anterior temporal lobe, medial part
Time since AD diagnosis (months)
Sample Size
10
20
30
50
90
Legend
Average Value
Spline

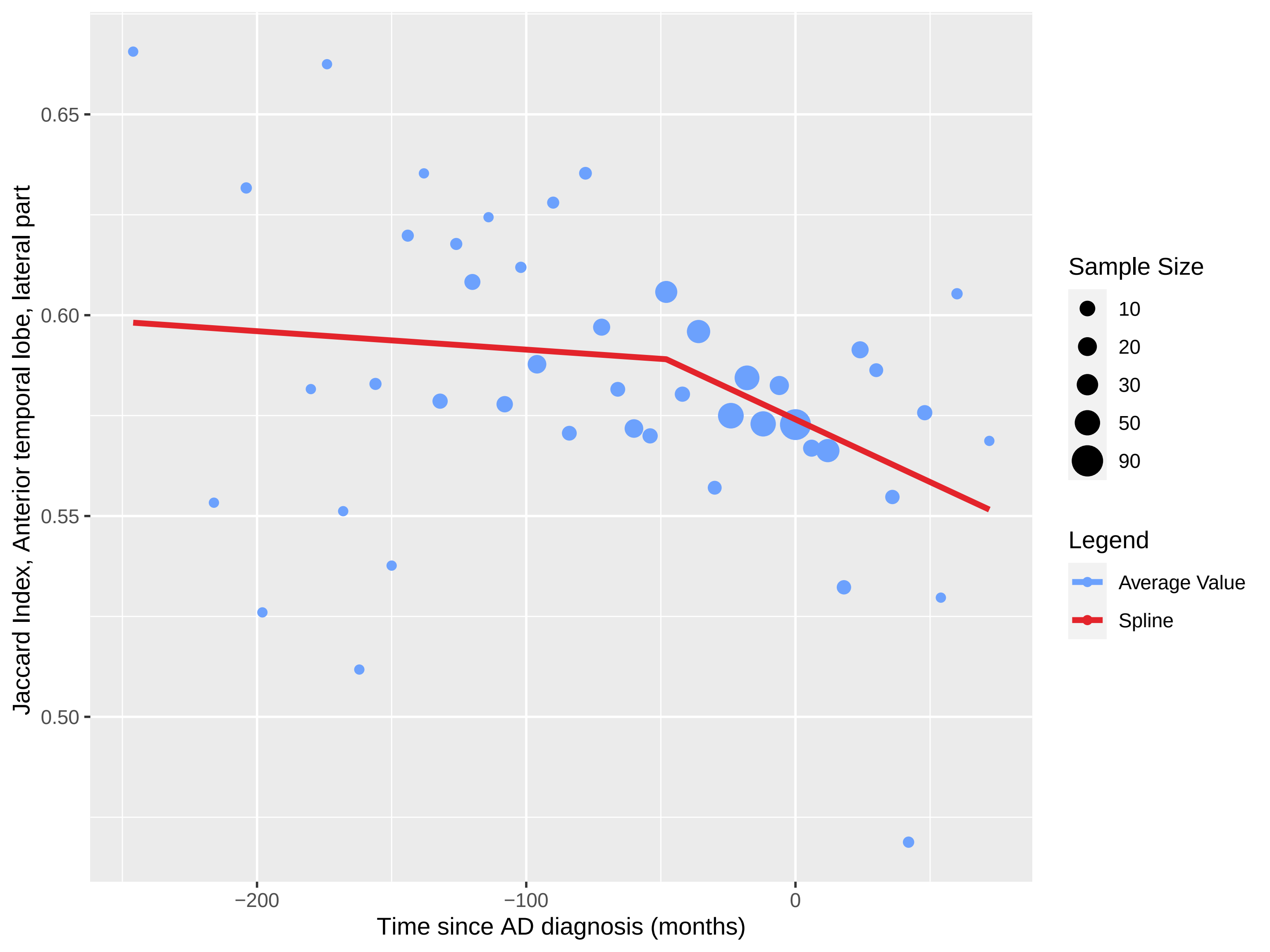

Jaccard Index, Anterior temporal lobe, lateral part
0.65
0.60
0.55
0.50
−200
−100
0
Time since AD diagnosis (months)
Sample Size
10
20
30
50
90
Legend
Average Value
Spline

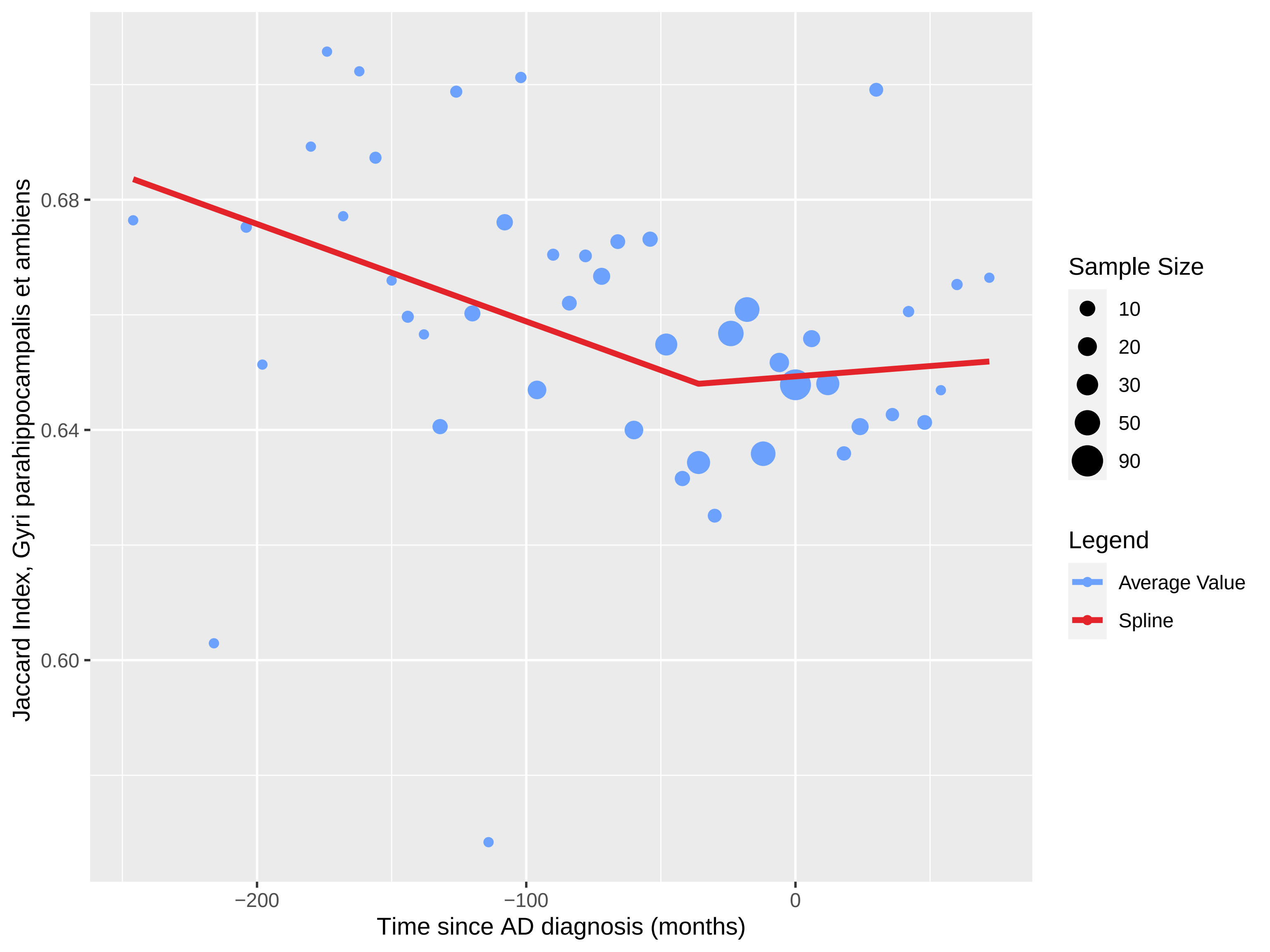

Jaccard Index, Gyri parahippocampalis et ambiens
0.68
0.64
0.60
−200
−100
0
Time since AD diagnosis (months)
Sample Size
10
20
30
50
90
Legend
Average Value
Spline

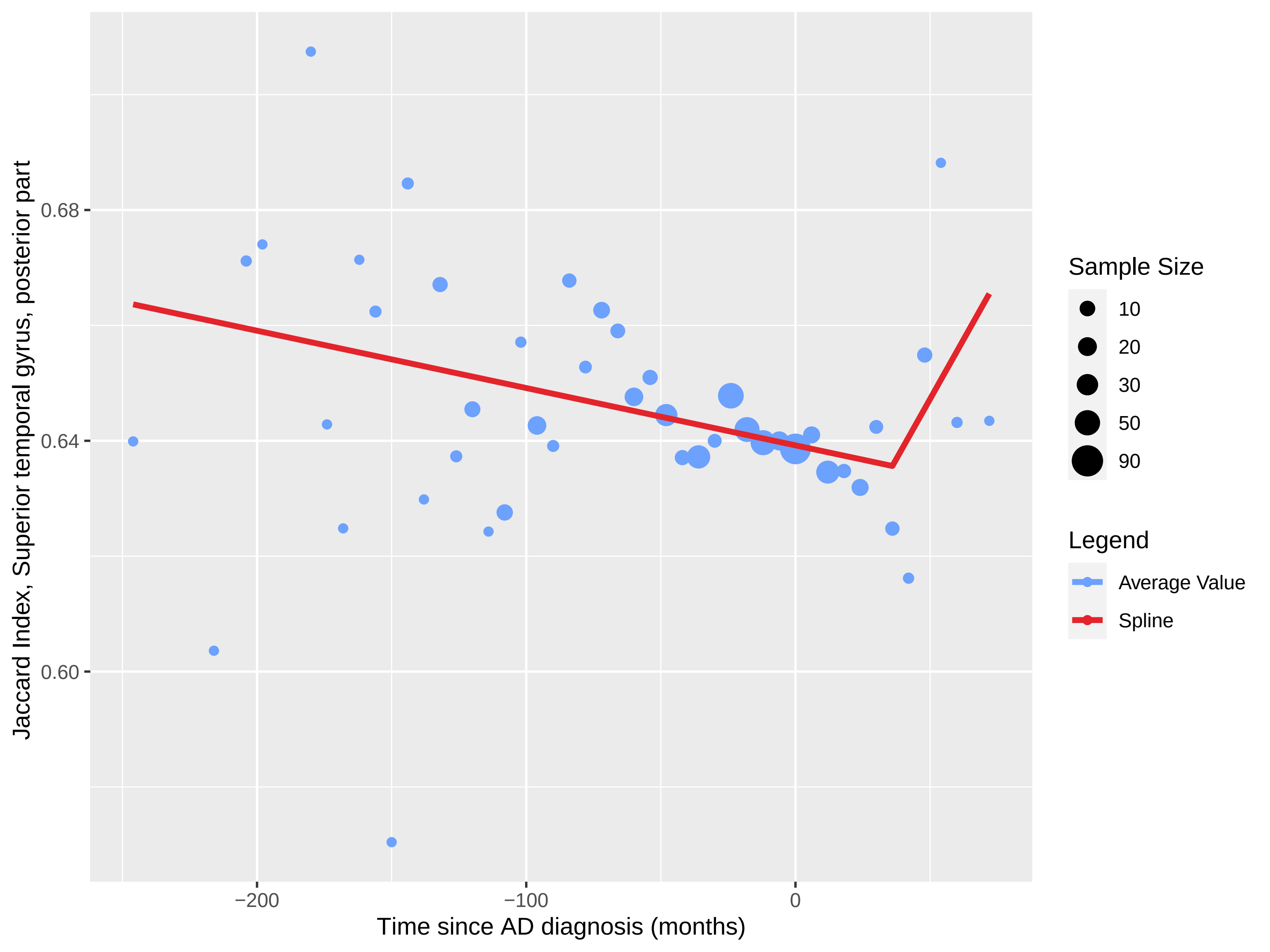

Jaccard Index, Superior temporal gyrus, posterior part
0.68
0.64
0.60
−200
−100
0
Time since AD diagnosis (months)
Sample Size
10
20
30
50
90
Legend
Average Value
Spline

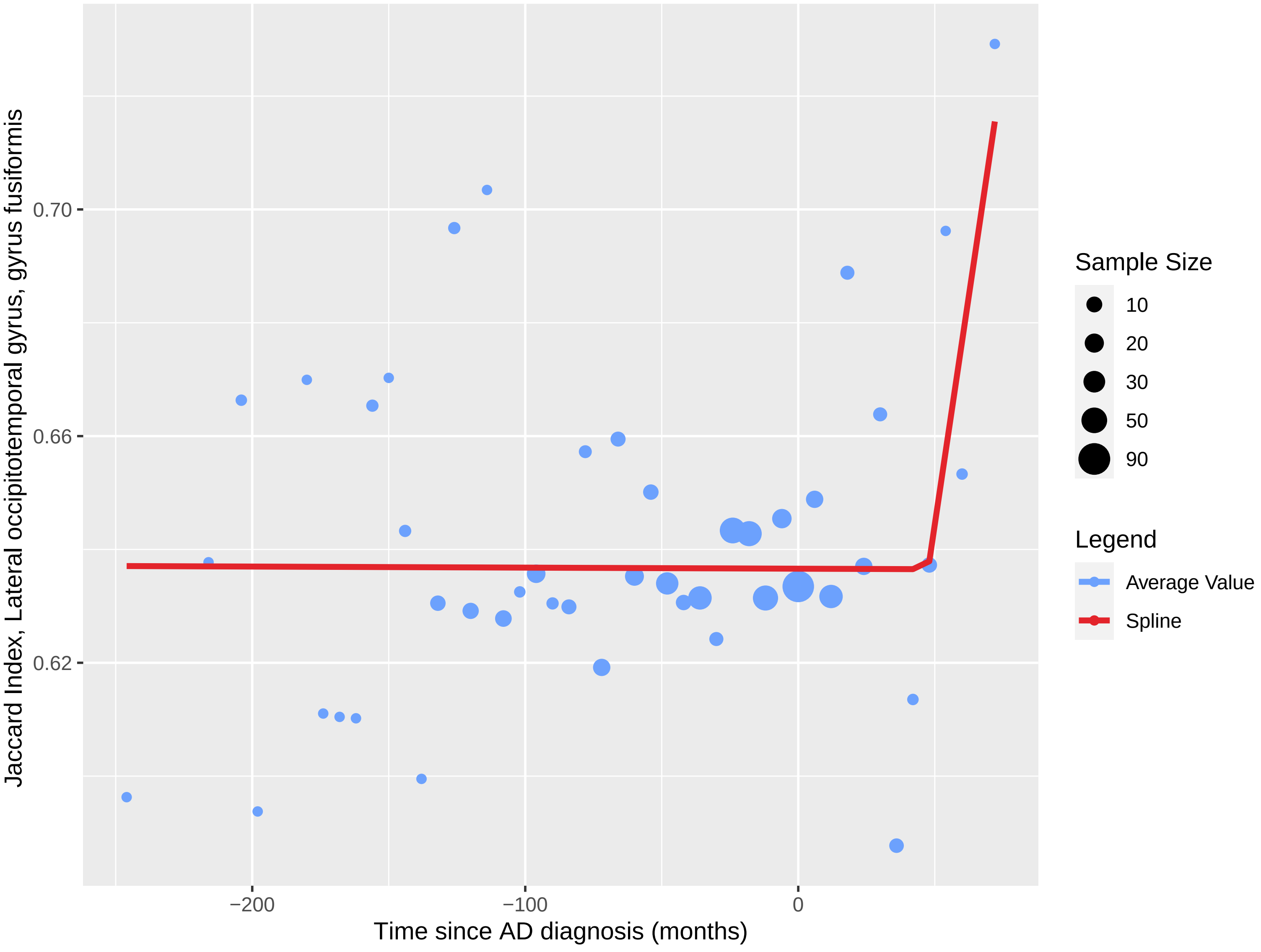

Jaccard Index, Lateral occipitotemporal gyrus, gyrus fusiformis
0.70
0.66
0.62
−200
−100
0
Time since AD diagnosis (months)
Sample Size
10
20
30
50
90
Legend
Average Value
Spline

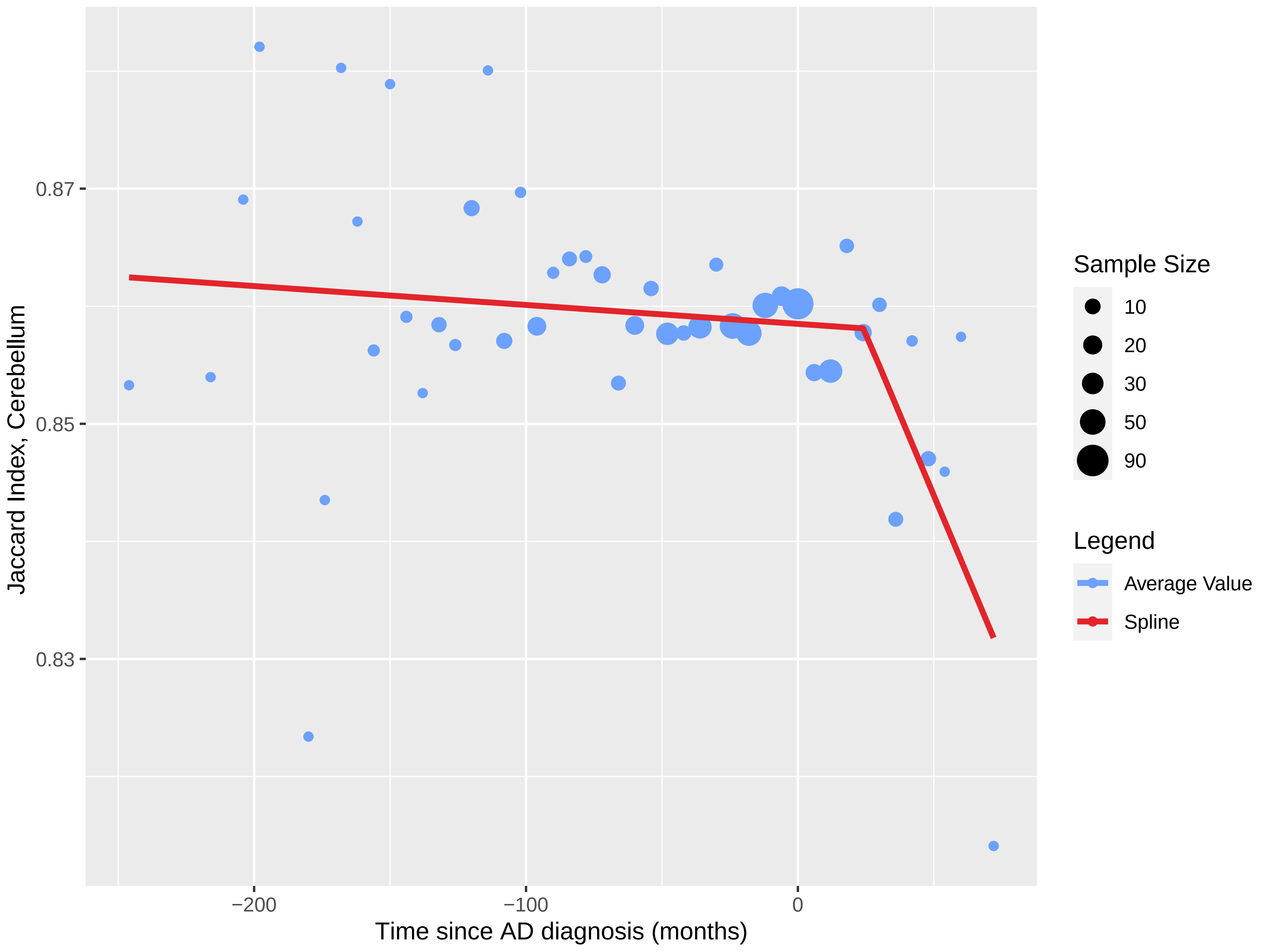

0.87
0.85
0.83
Jaccard Index, Cerebellum
−200
−100
0
Time since AD diagnosis (months)
Sample Size
10
20
30
50
90
Legend
Average Value
Spline

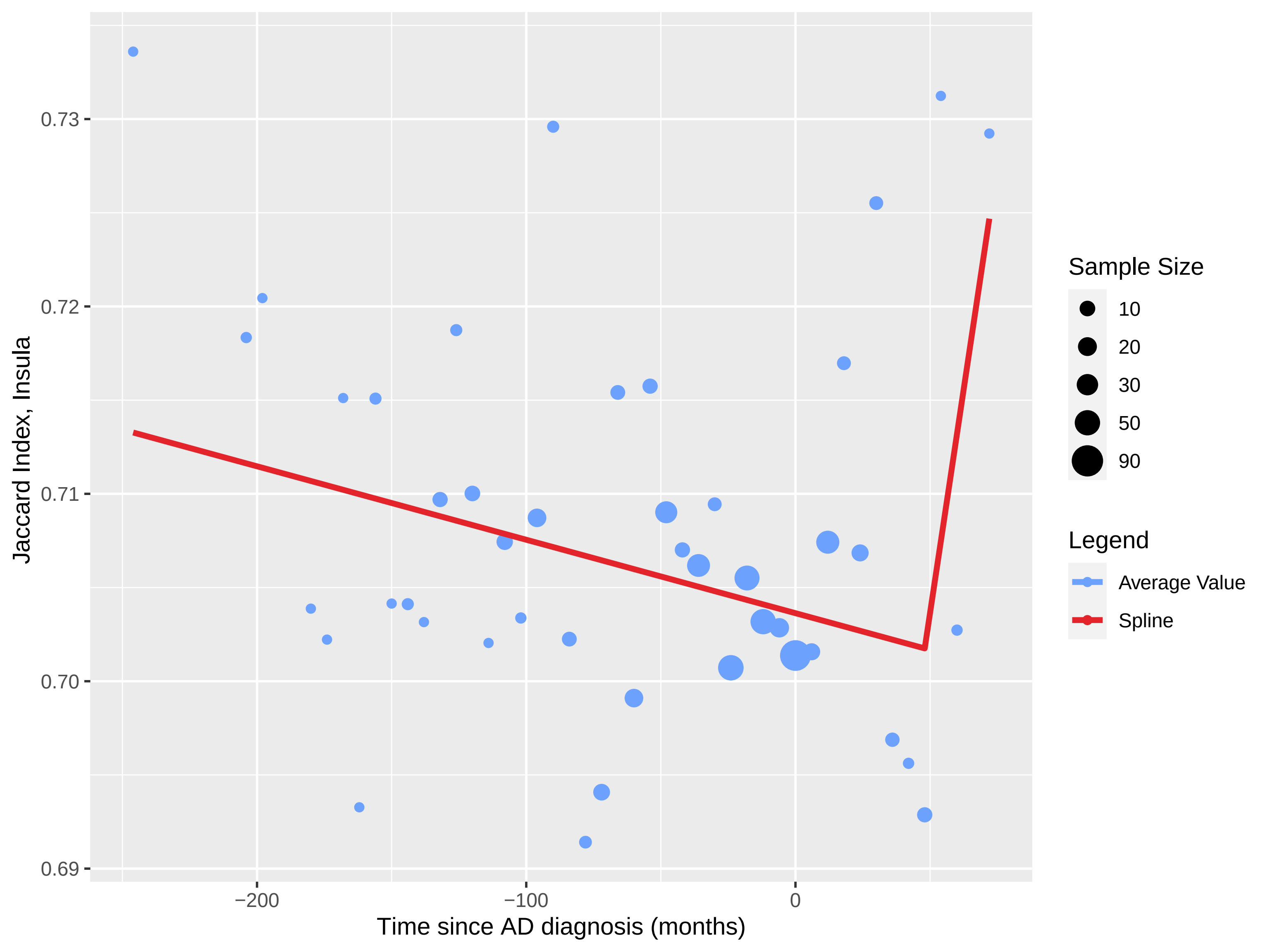

Jaccard Index, Insula
0.73
0.72
0.71
0.70
0.69
−200
−100
0
Time since AD diagnosis (months)
Sample Size
10
20
30
50
90
Legend
Average Value
Spline

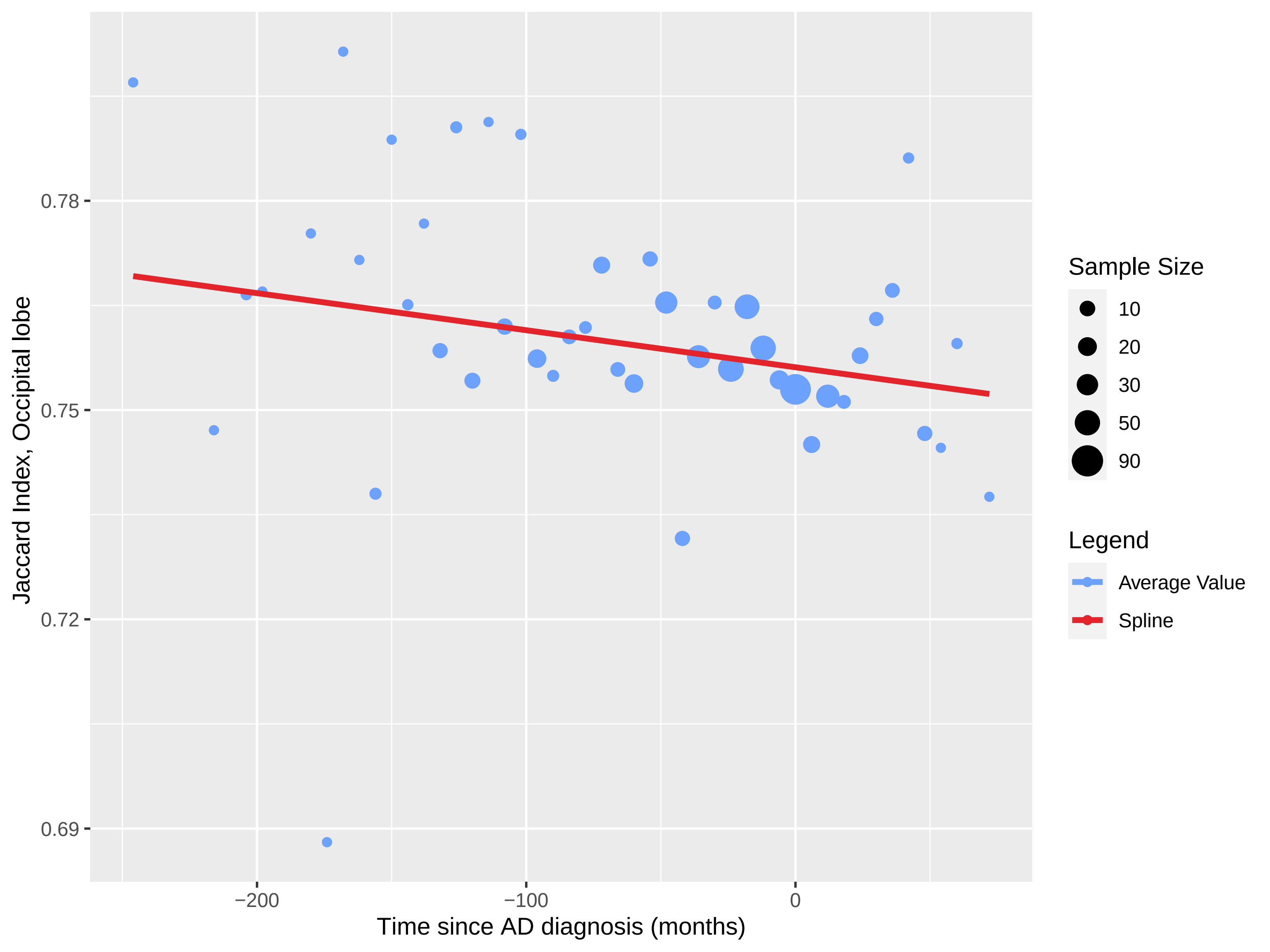

Jaccard Index, Occipital lobe
0.78
0.75
0.72
0.69
−200
−100
0
Time since AD diagnosis (months)
Sample Size
10
20
30
50
90
Legend
Average Value
Spline

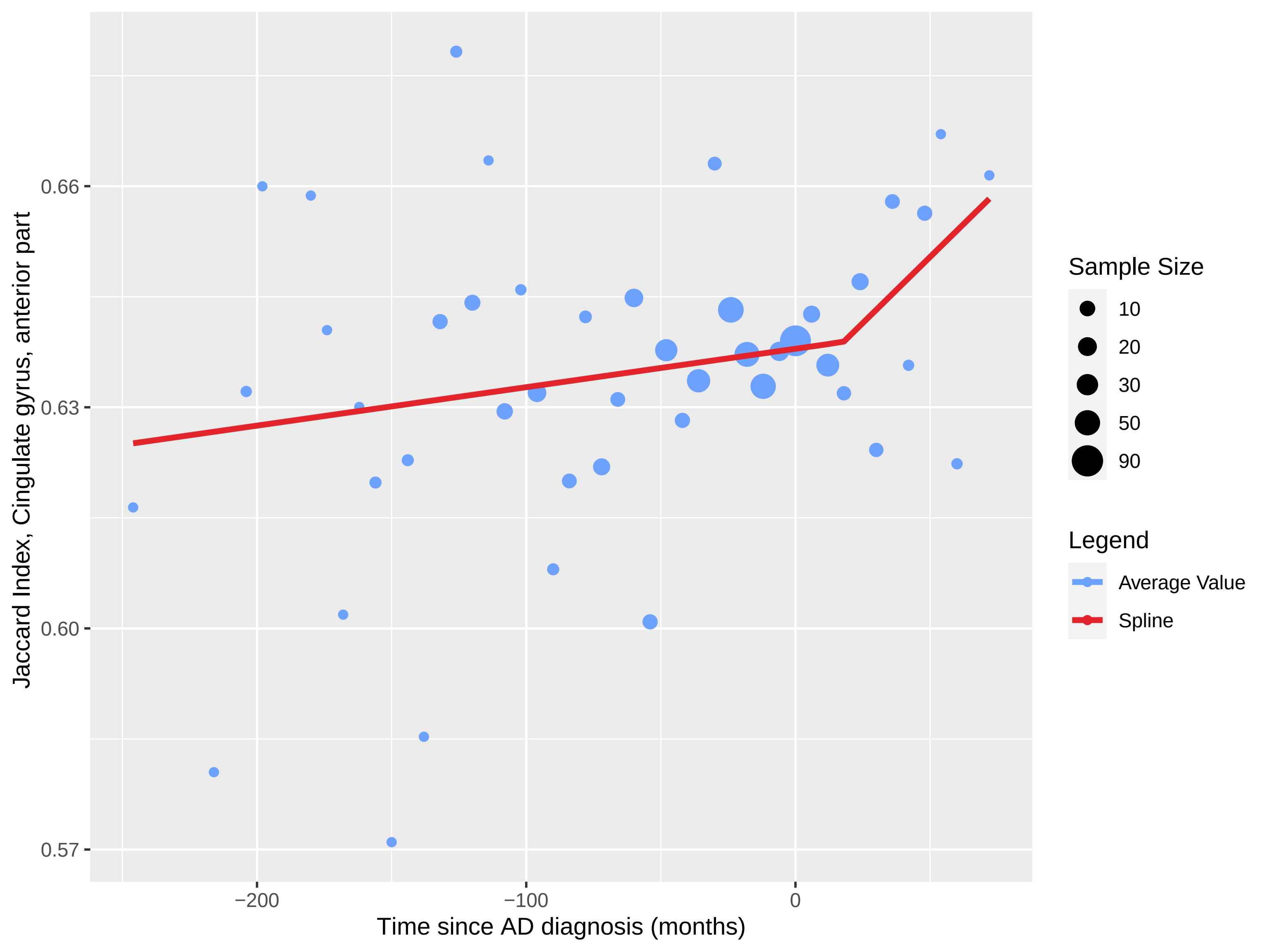

Jaccard Index, Cingulate gyrus, anterior part
0.66
0.63
0.60
0.57
−200
−100
0
Time since AD diagnosis (months)
Sample Size
10
20
30
50
90
Legend
Average Value
Spline

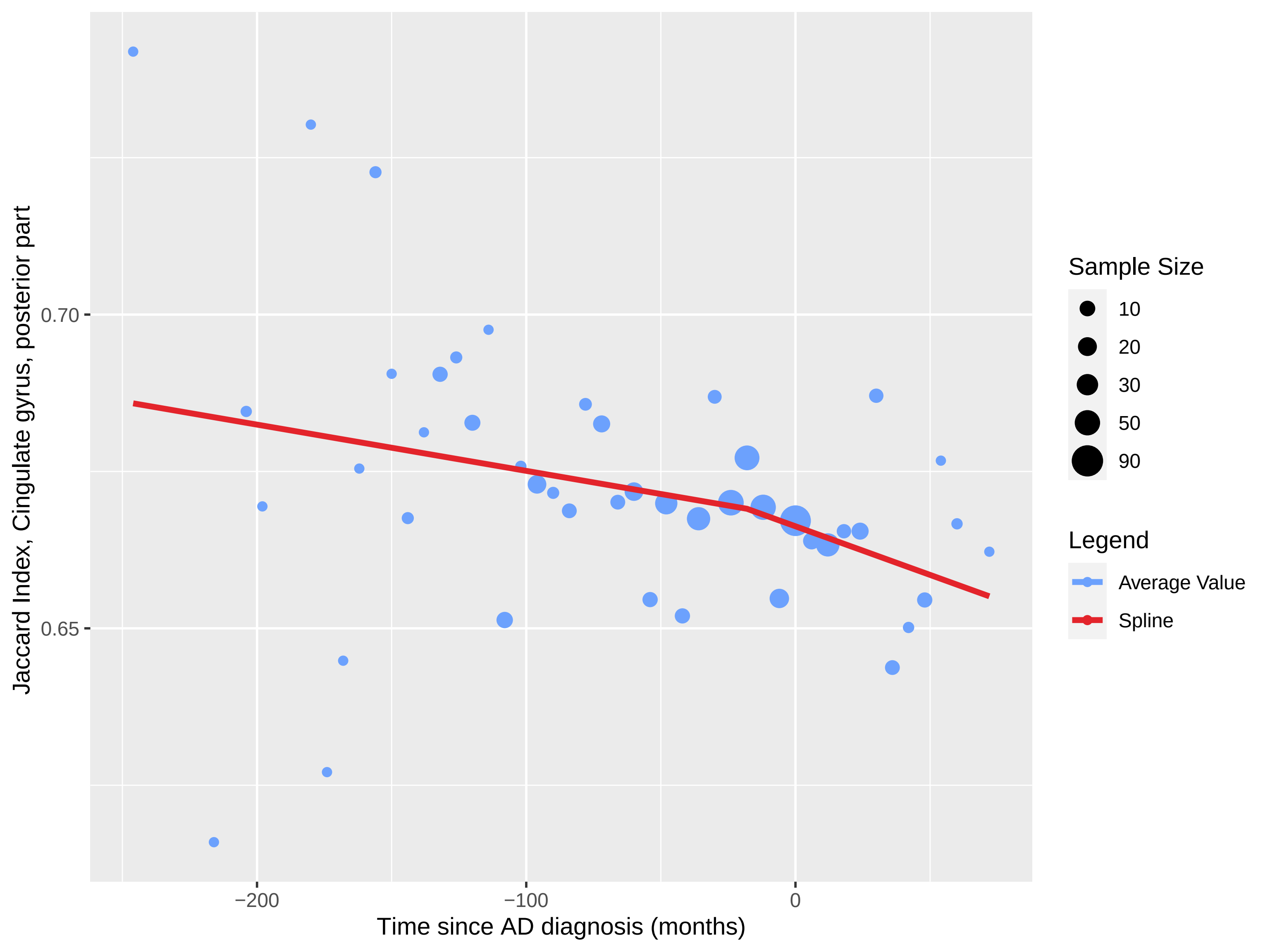

Jaccard Index, Cingulate gyrus, posterior part
0.70
0.65
−200
−100
0
Time since AD diagnosis (months)
Sample Size
10
20
30
50
90
Legend
Average Value
Spline

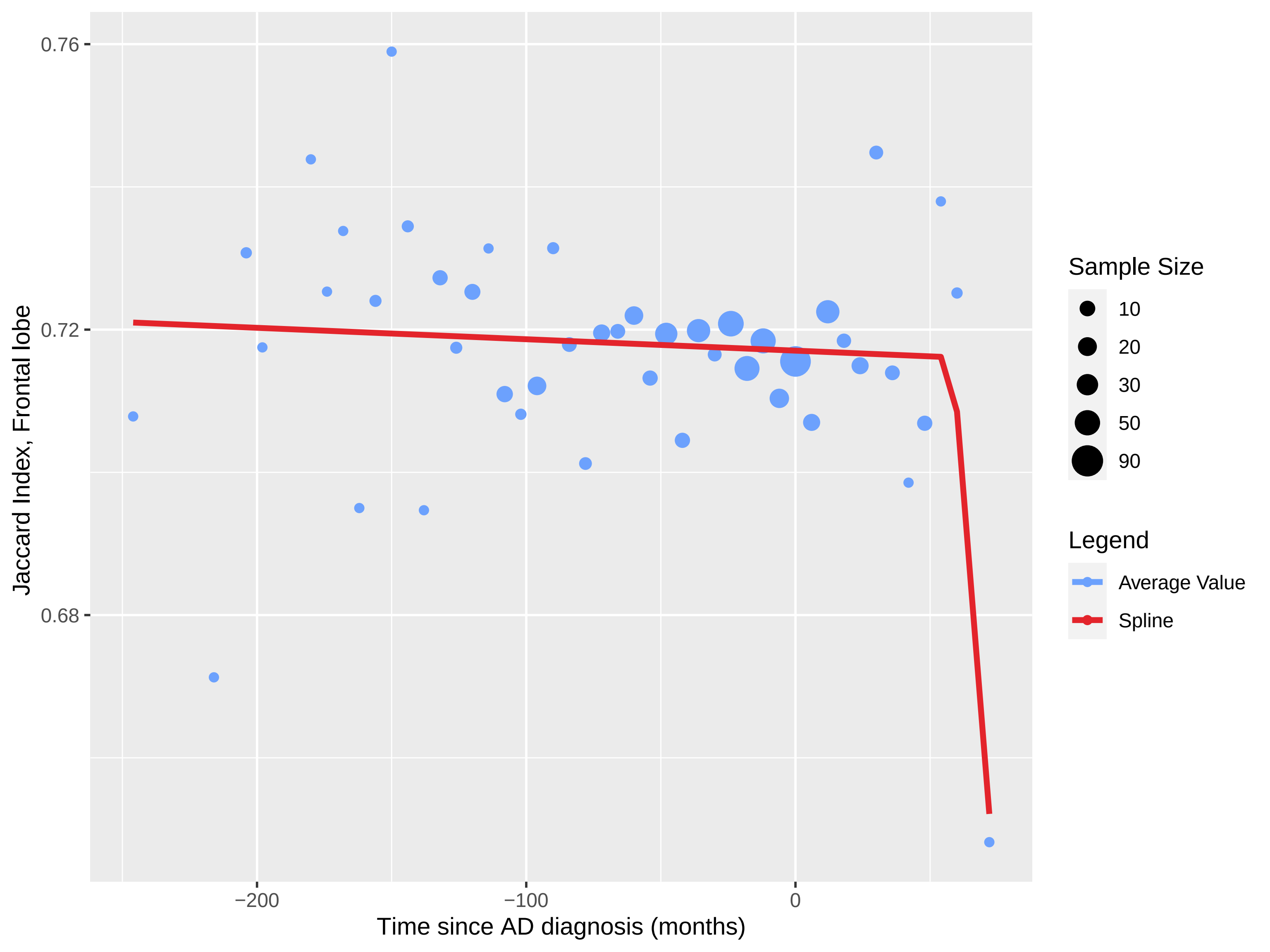

0.76
0.72
0.68
−200
−100
0
Time since AD diagnosis (months)
Jaccard Index, Frontal lobe
Sample Size
10
20
30
50
90
Legend
Average Value
Spline

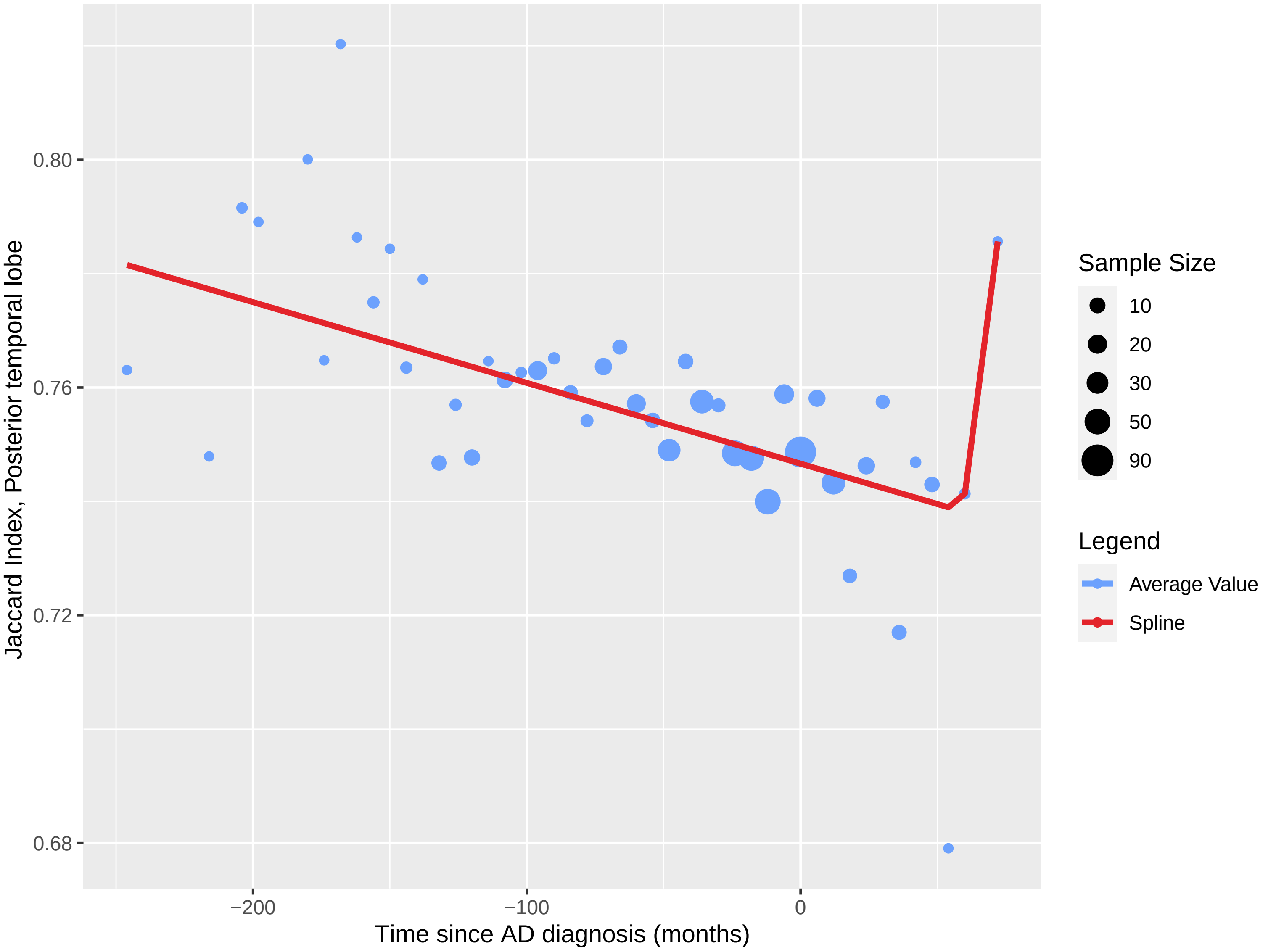

Jaccard Index, Posterior temporal lobe
0.80
0.76
0.72
0.68
−200
−100
0
Time since AD diagnosis (months)
Sample Size
10
20
30
50
90
Legend
Average Value
Spline

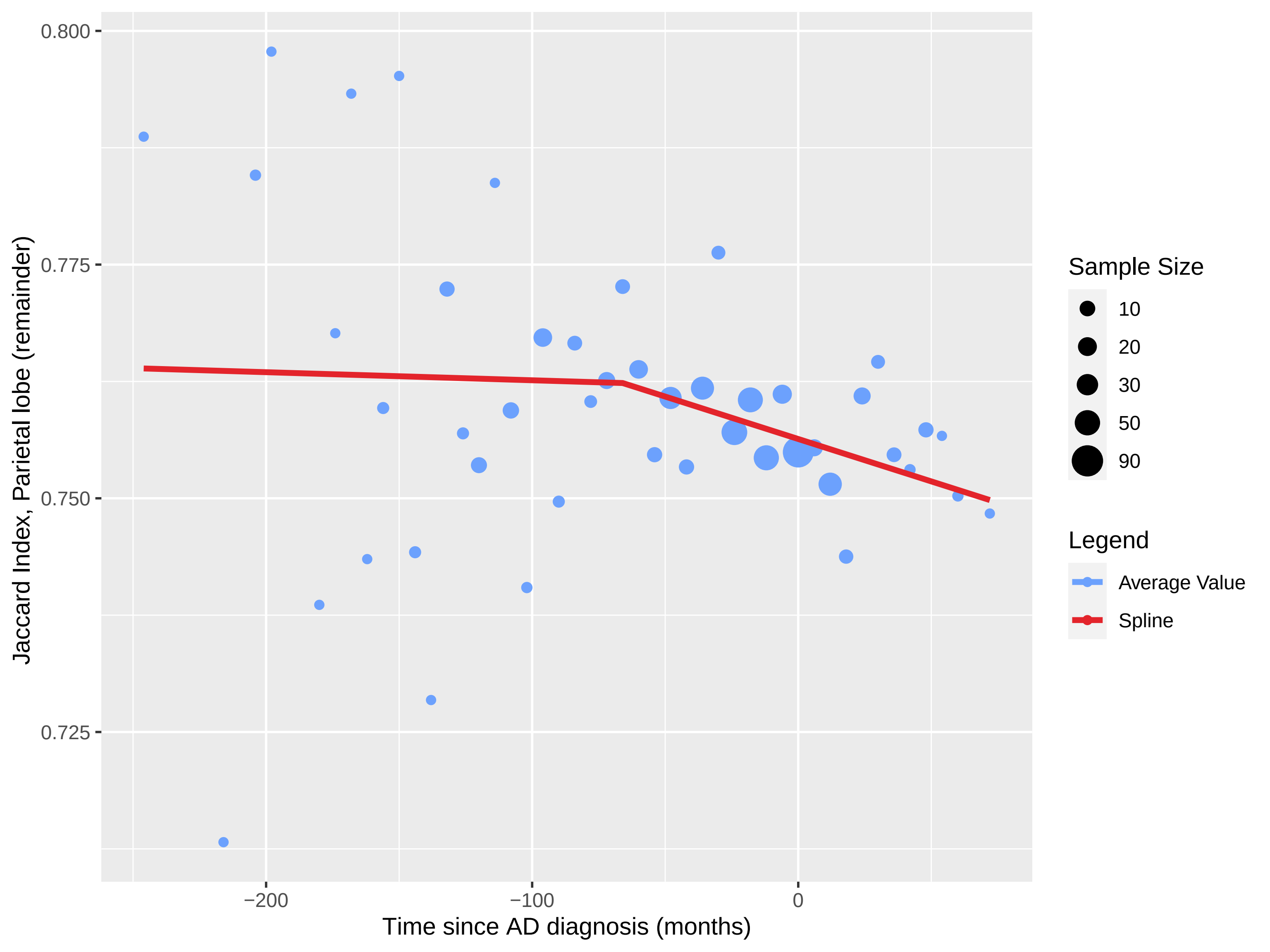

0.800
0.775
0.750
0.725
Jaccard Index, Parietal lobe (remainder)
−200
−100
0
Time since AD diagnosis (months)
Sample Size
10
20
30
50
90
Legend
Average Value
Spline

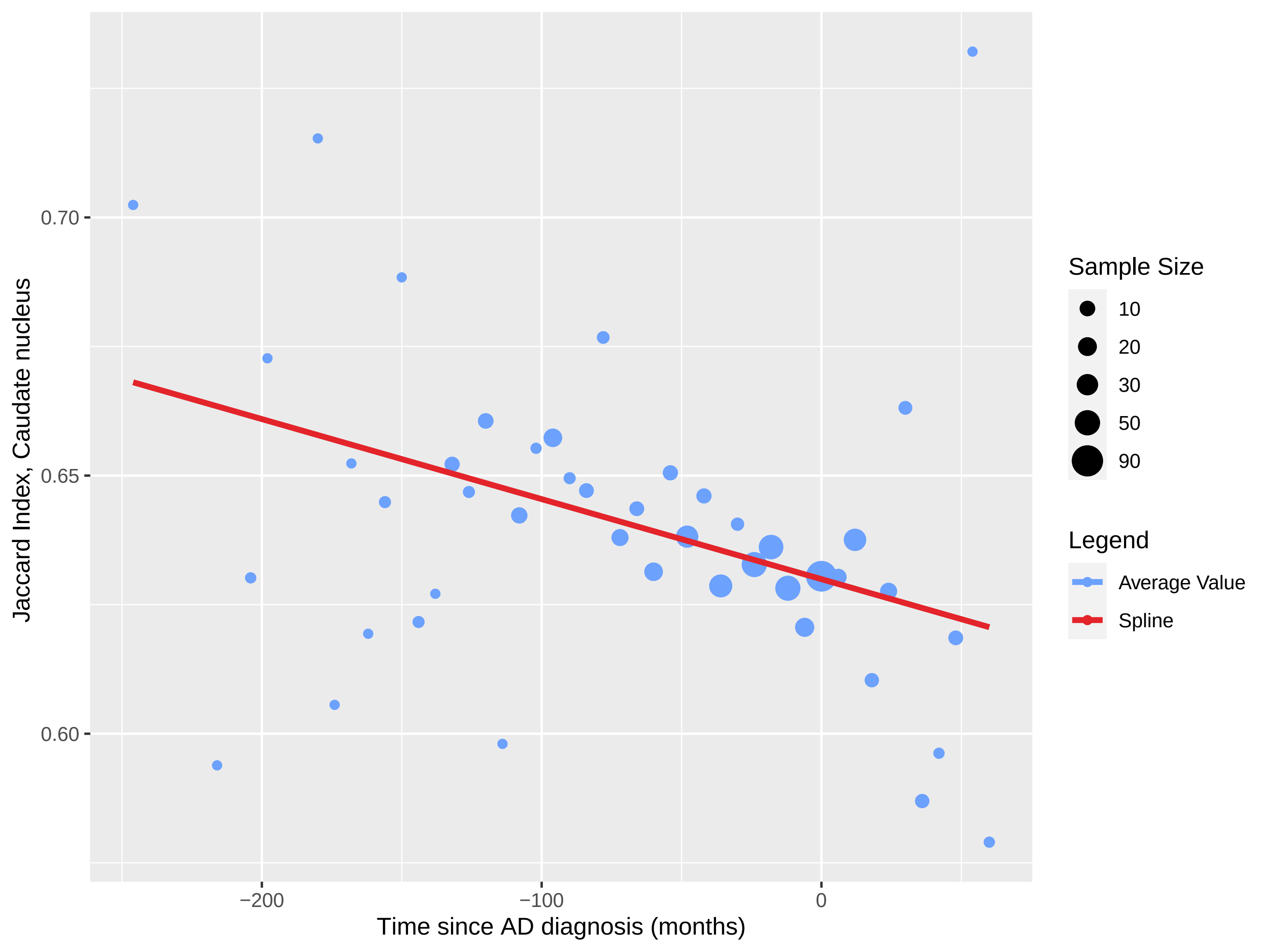

Jaccard Index, Caudate nucleus
0.70
0.65
0.60
−200
−100
0
Time since AD diagnosis (months)
Sample Size
10
20
30
50
90
Legend
Average Value
Spline

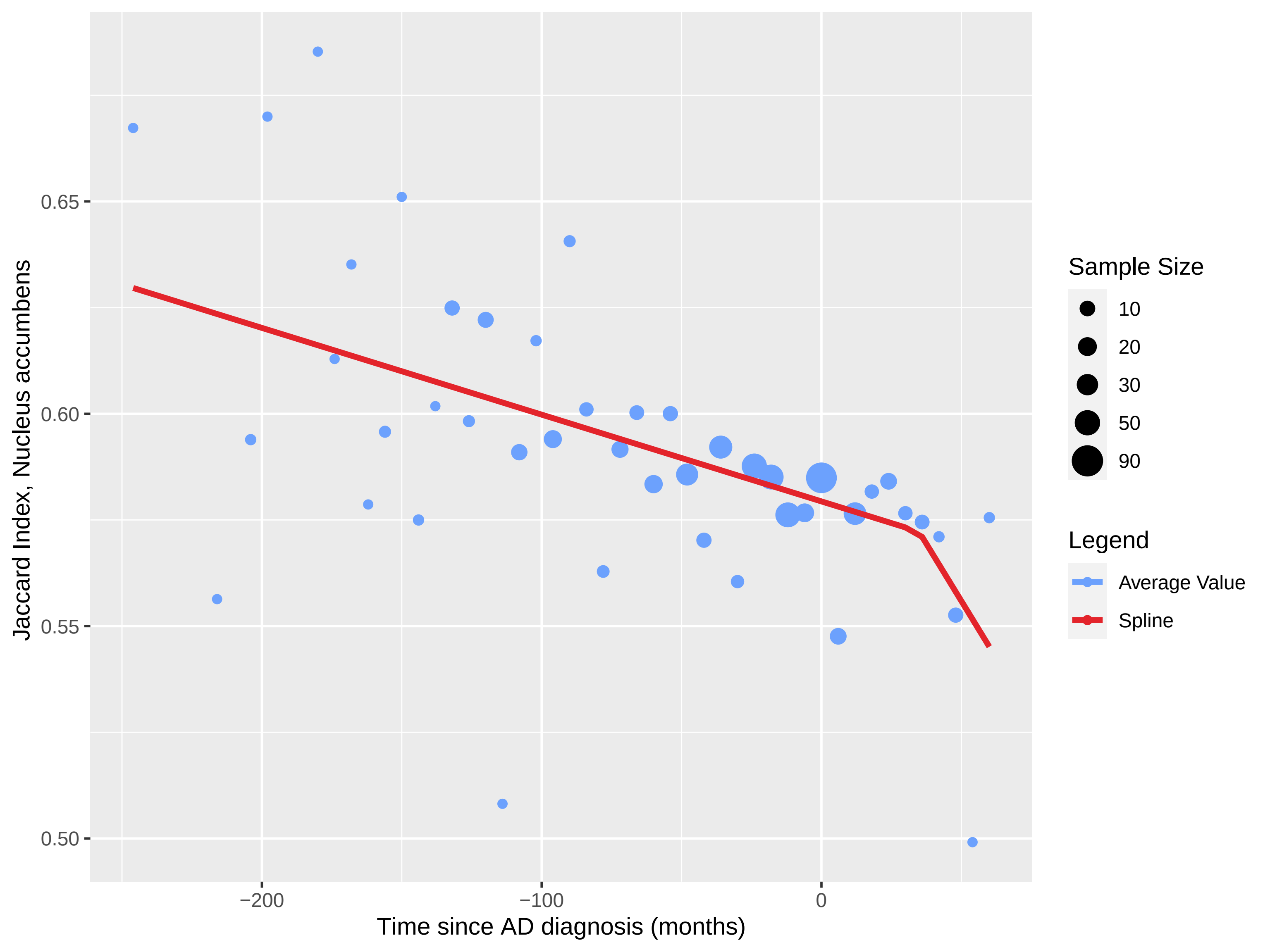

Jaccard Index, Nucleus accumbens
0.65
0.60
0.55
0.50
−200
−100
0
Time since AD diagnosis (months)
Sample Size
10
20
30
50
90
Legend
Average Value
Spline

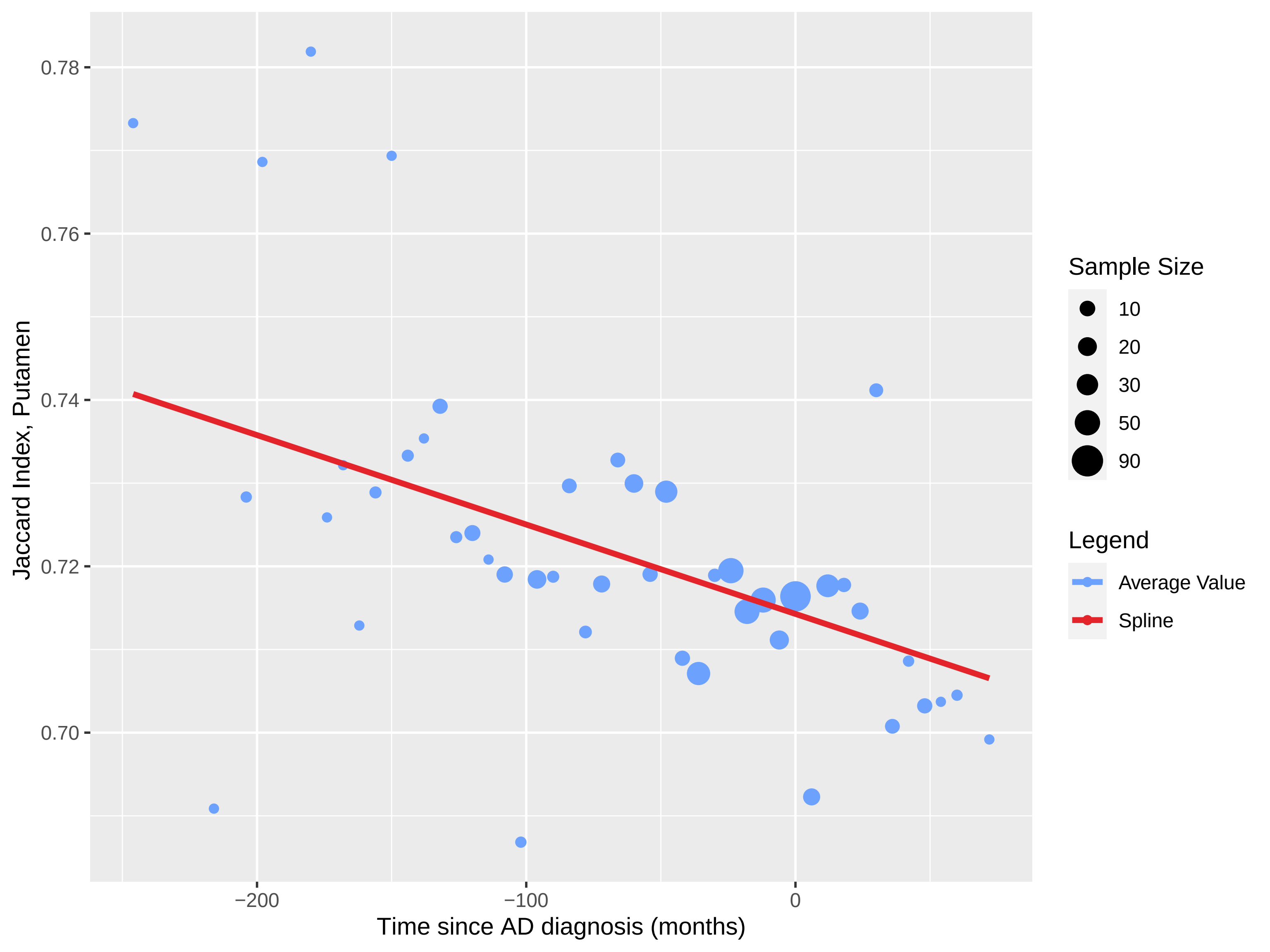

0.78
0.76
0.74
0.72
0.70
−200
−100
0
Time since AD diagnosis (months)
Jaccard Index, Putamen
Sample Size
10
20
30
50
90
Legend
Average Value
Spline

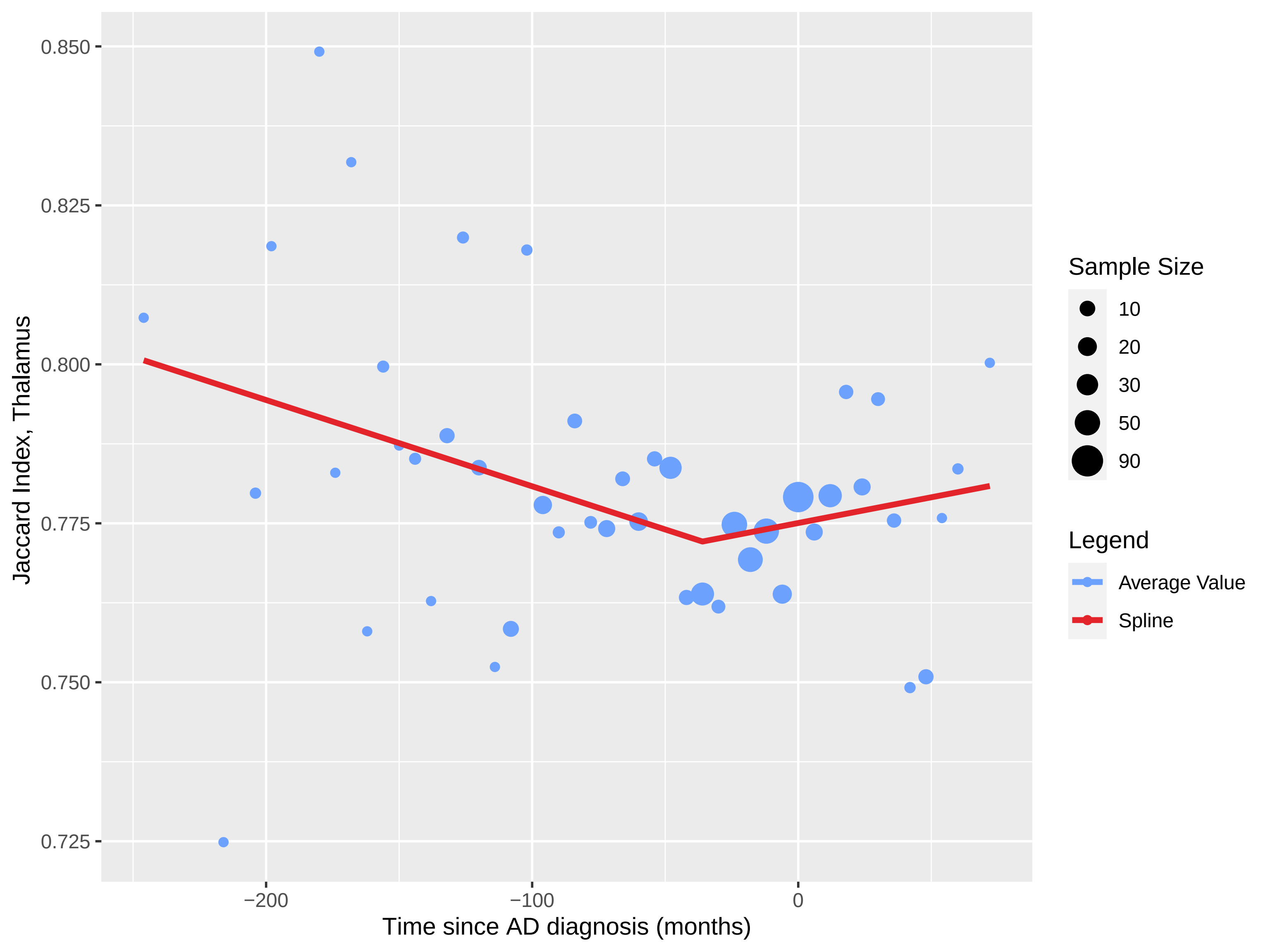

0.850
0.825
0.800
0.775
0.750
0.725
Jaccard Index, Thalamus
−200
−100
0
Time since AD diagnosis (months)
Sample Size
10
20
30
50
90
Legend
Average Value
Spline

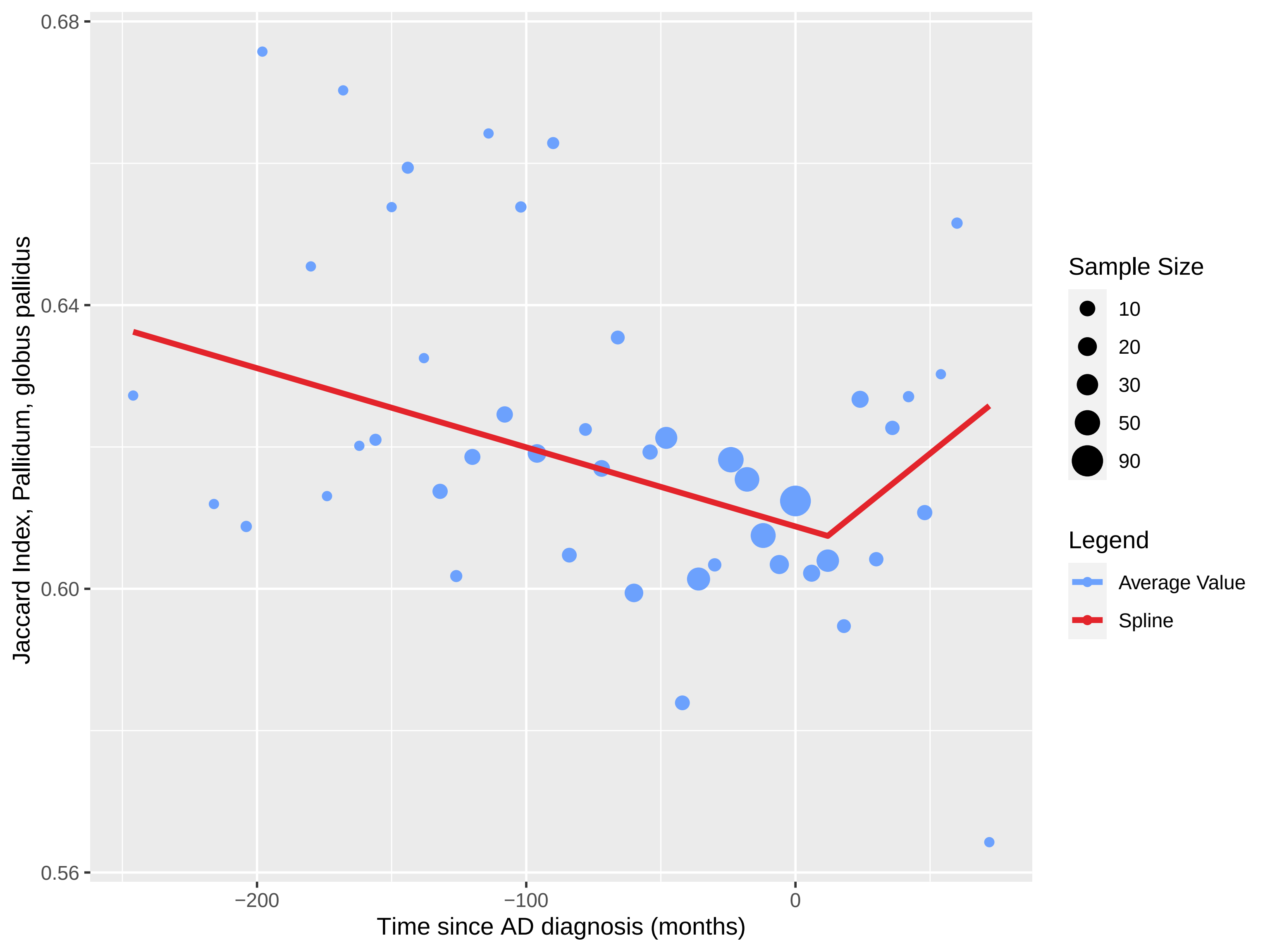

0.68
0.64
0.60
0.56
Jaccard Index, Pallidum, globus pallidus
−200
−100
0
Time since AD diagnosis (months)
Sample Size
10
20
30
50
90
Legend
Average Value
Spline

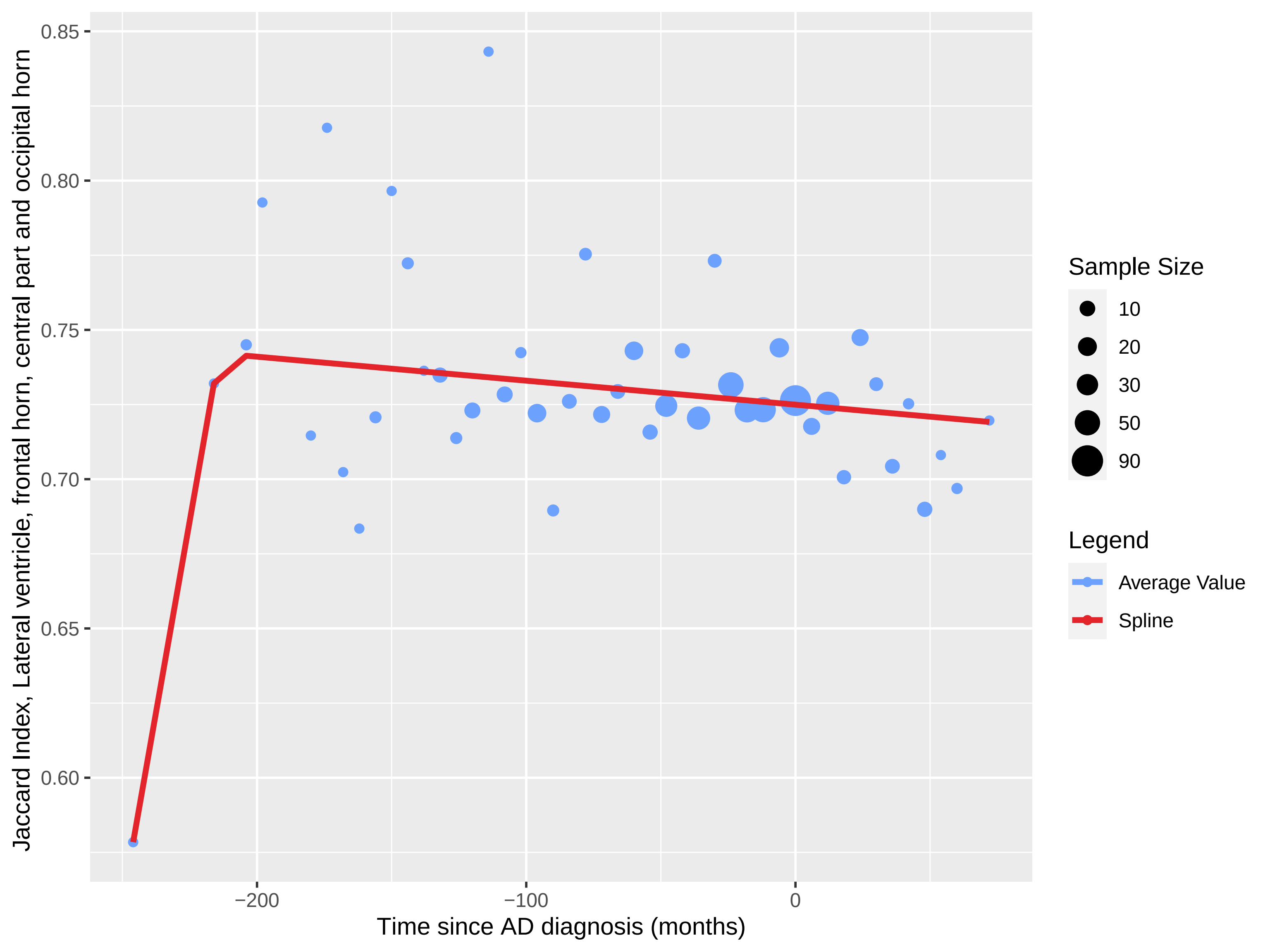

Jaccard Index, Lateral ventricle, frontal horn, central part and occipital horn
0.85
0.80
0.75
0.70
0.65
0.60
−200
−100
0
Time since AD diagnosis (months)
Sample Size
10
20
30
50
90
Legend
Average Value
Spline

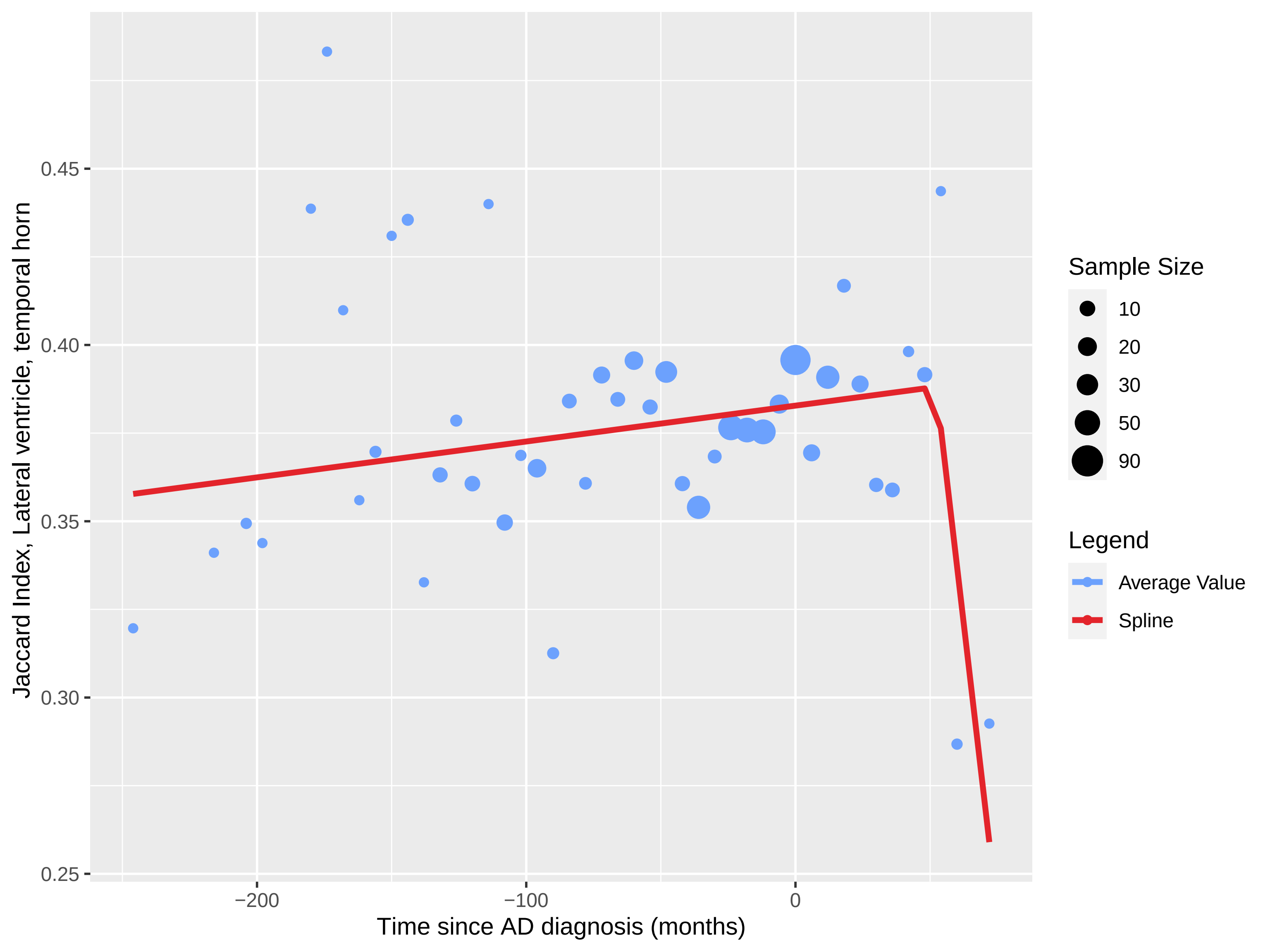

Jaccard Index, Lateral ventricle, temporal horn
0.25
0.30
0.35
0.40
0.45
−200
−100
0
Time since AD diagnosis (months)
Sample Size
10
20
30
50
90
Legend
Average Value
Spline

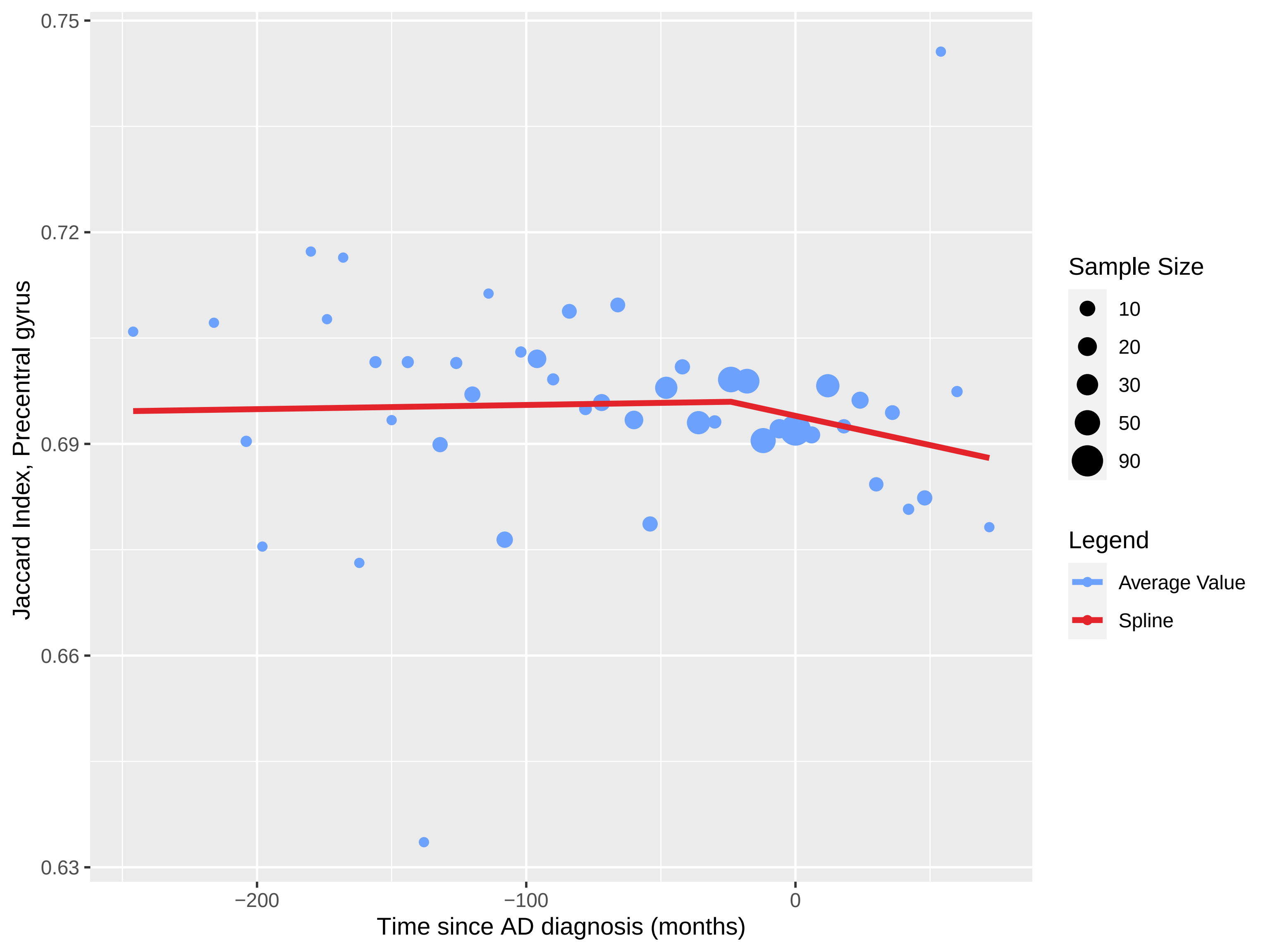

0.75
0.72
0.69
0.66
0.63
Jaccard Index, Precentral gyrus
−200
−100
0
Time since AD diagnosis (months)
Sample Size
10
20
30
50
90
Legend
Average Value
Spline

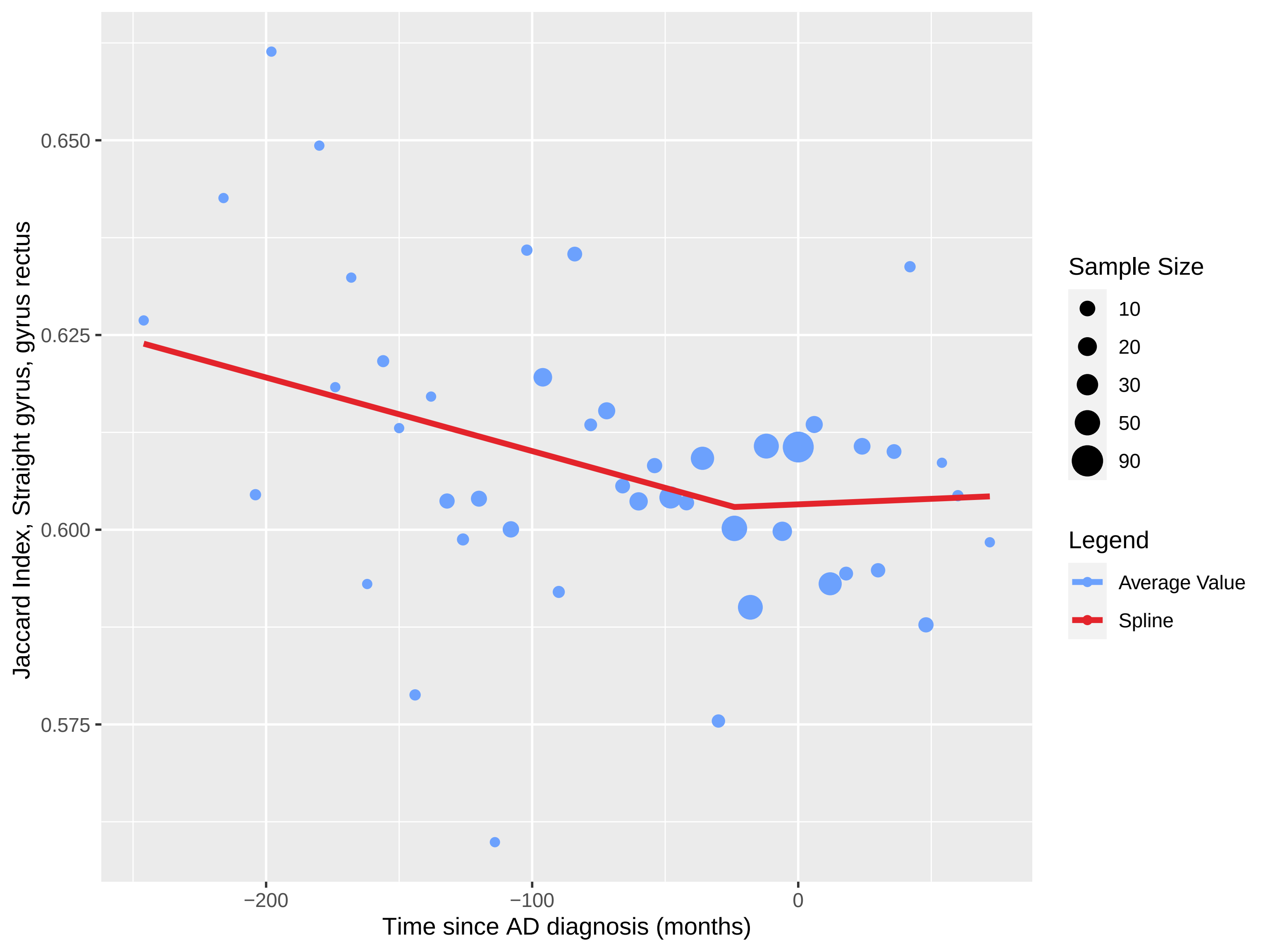

Jaccard Index, Straight gyrus, gyrus rectus
0.650
0.625
0.600
0.575
−200
−100
0
Time since AD diagnosis (months)
Sample Size
10
20
30
50
90
Legend
Average Value
Spline

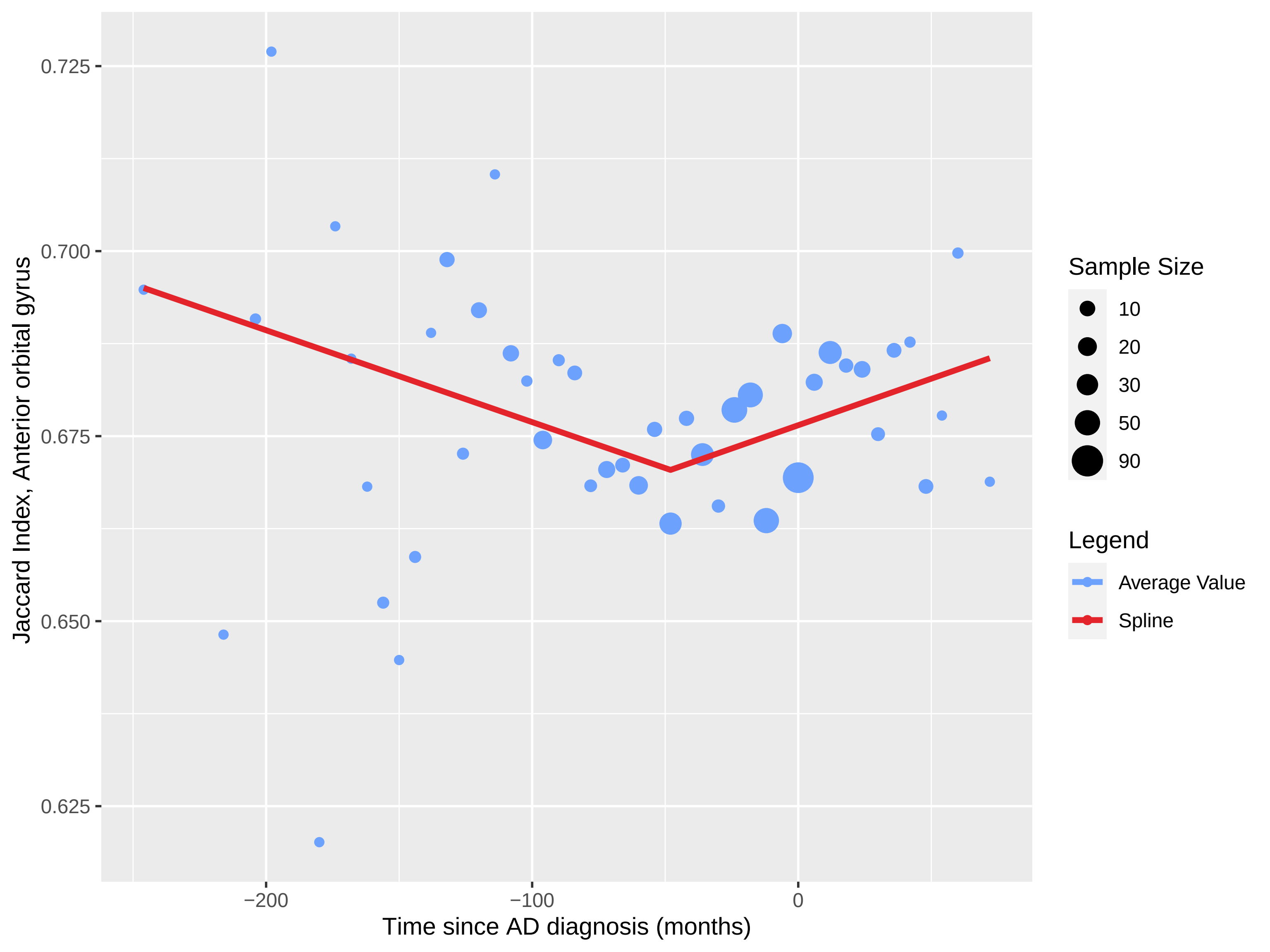

Jaccard Index, Anterior orbital gyrus
0.725
0.700
0.675
0.650
0.625
−200
−100
0
Time since AD diagnosis (months)
Sample Size
10
20
30
50
90
Legend
Average Value
Spline

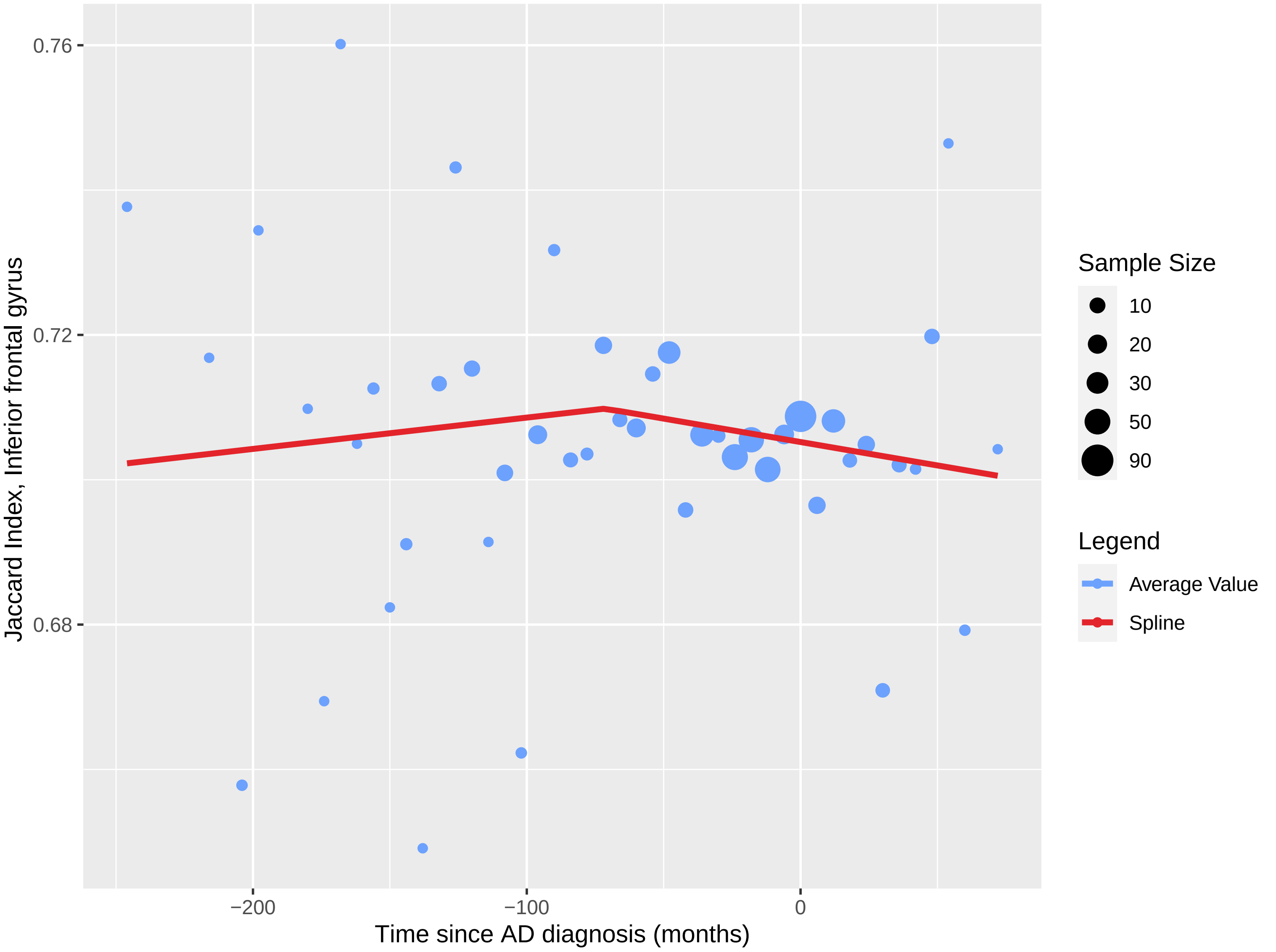

Jaccard Index, Inferior frontal gyrus
0.76
0.72
0.68
−200
−100
0
Time since AD diagnosis (months)
Sample Size
10
20
30
50
90
Legend
Average Value
Spline

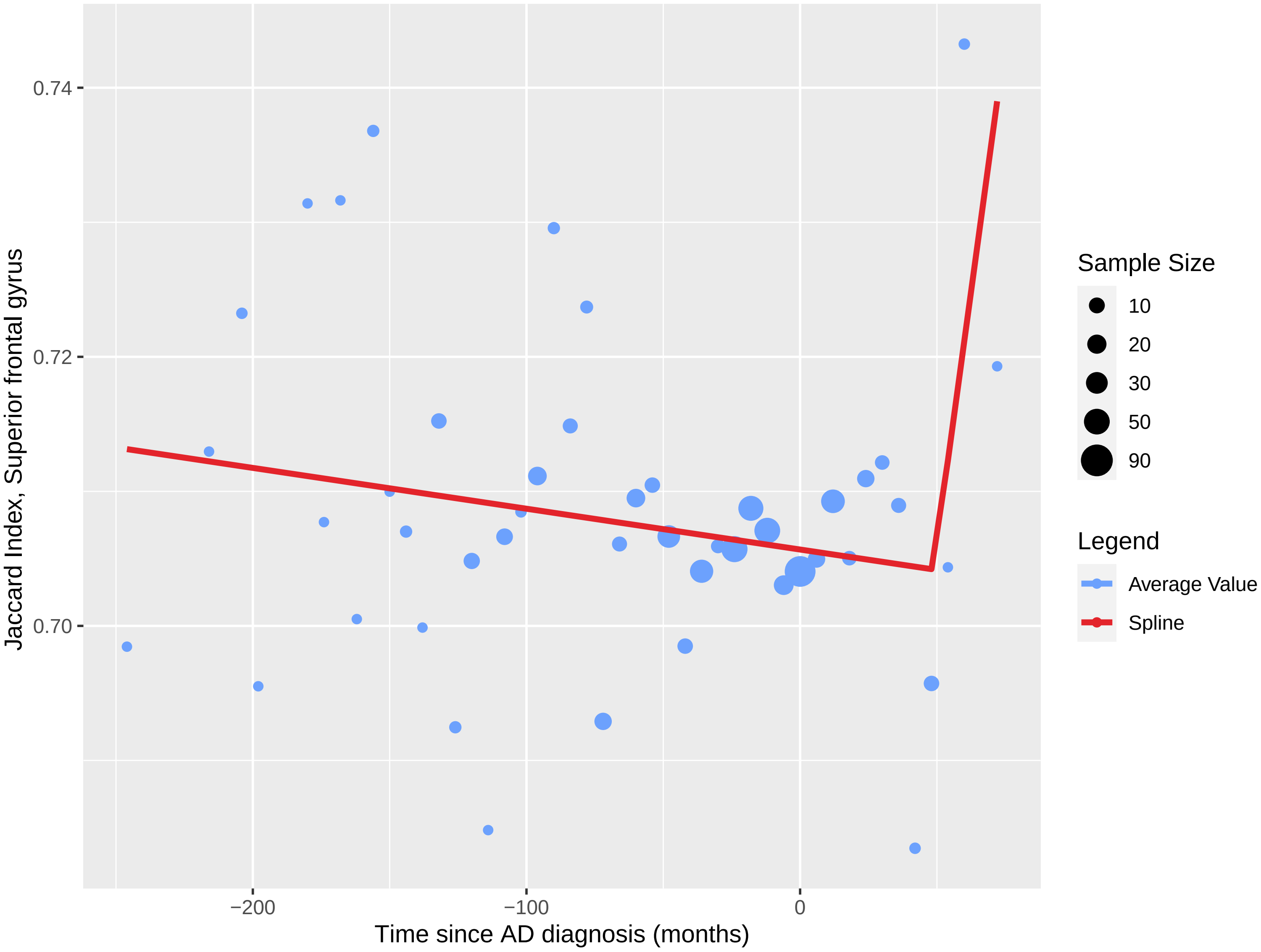

Jaccard Index, Superior frontal gyrus
0.74
0.72
0.70
−200
−100
0
Time since AD diagnosis (months)
Sample Size
10
20
30
50
90
Legend
Average Value
Spline

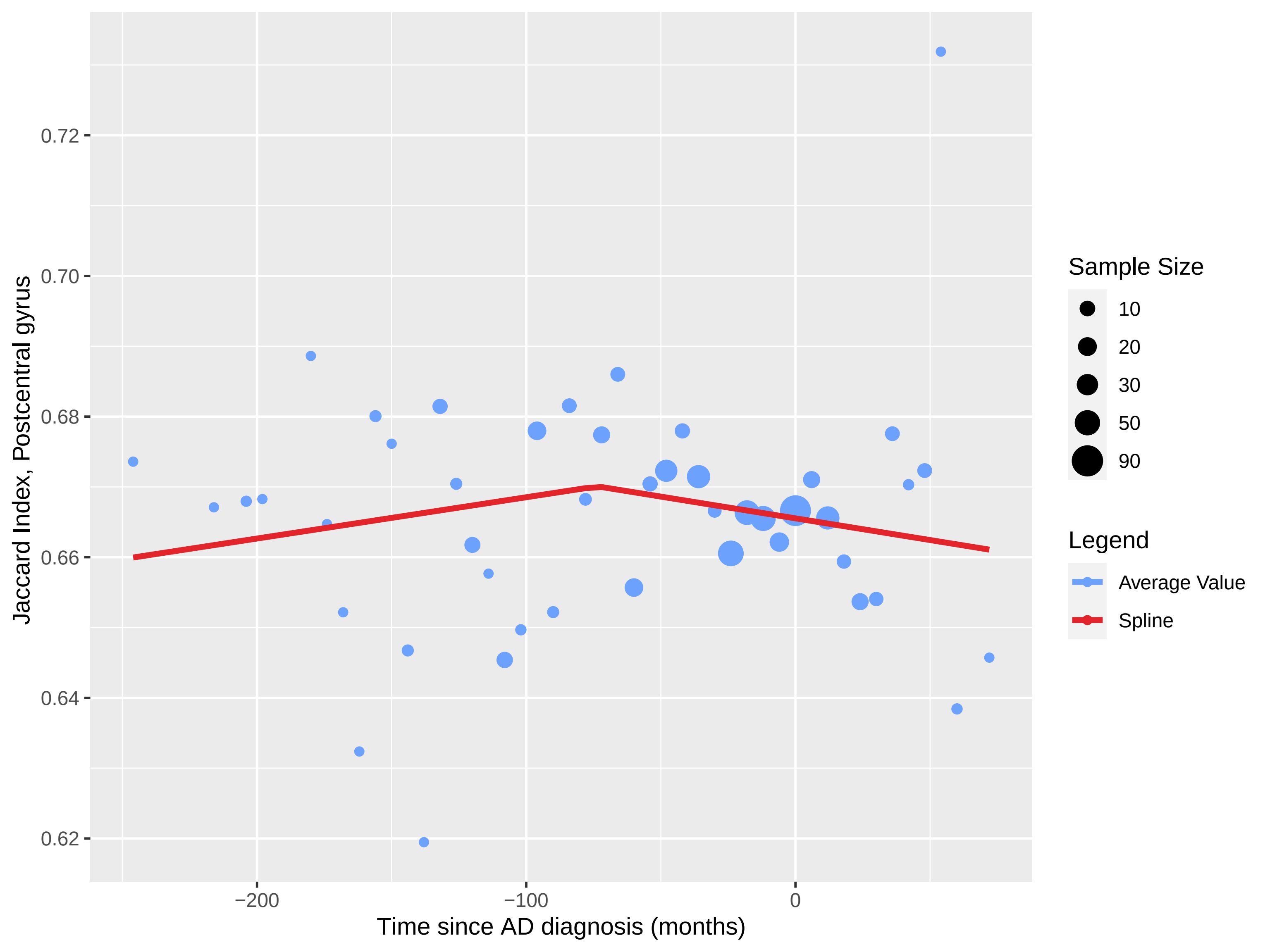

Jaccard Index, Postcentral gyrus
0.72
0.70
0.68
0.66
0.64
0.62
−200
−100
0
Time since AD diagnosis (months)
Sample Size
10
20
30
50
90
Legend
Average Value
Spline

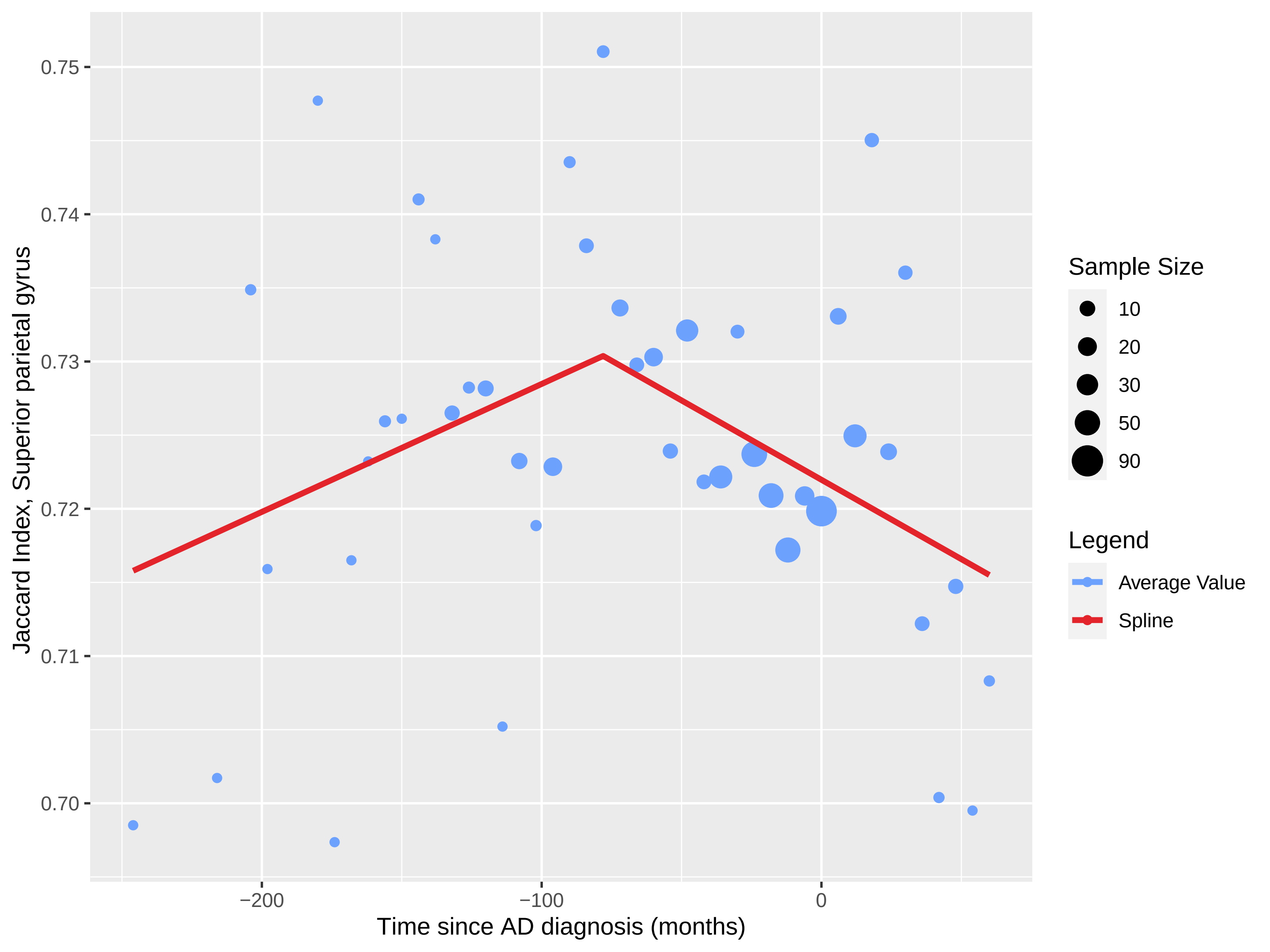

Jaccard Index, Superior parietal gyrus
0.75
0.74
0.73
0.72
0.71
0.70
−200
−100
0
Time since AD diagnosis (months)
Sample Size
10
20
30
50
90
Legend
Average Value
Spline

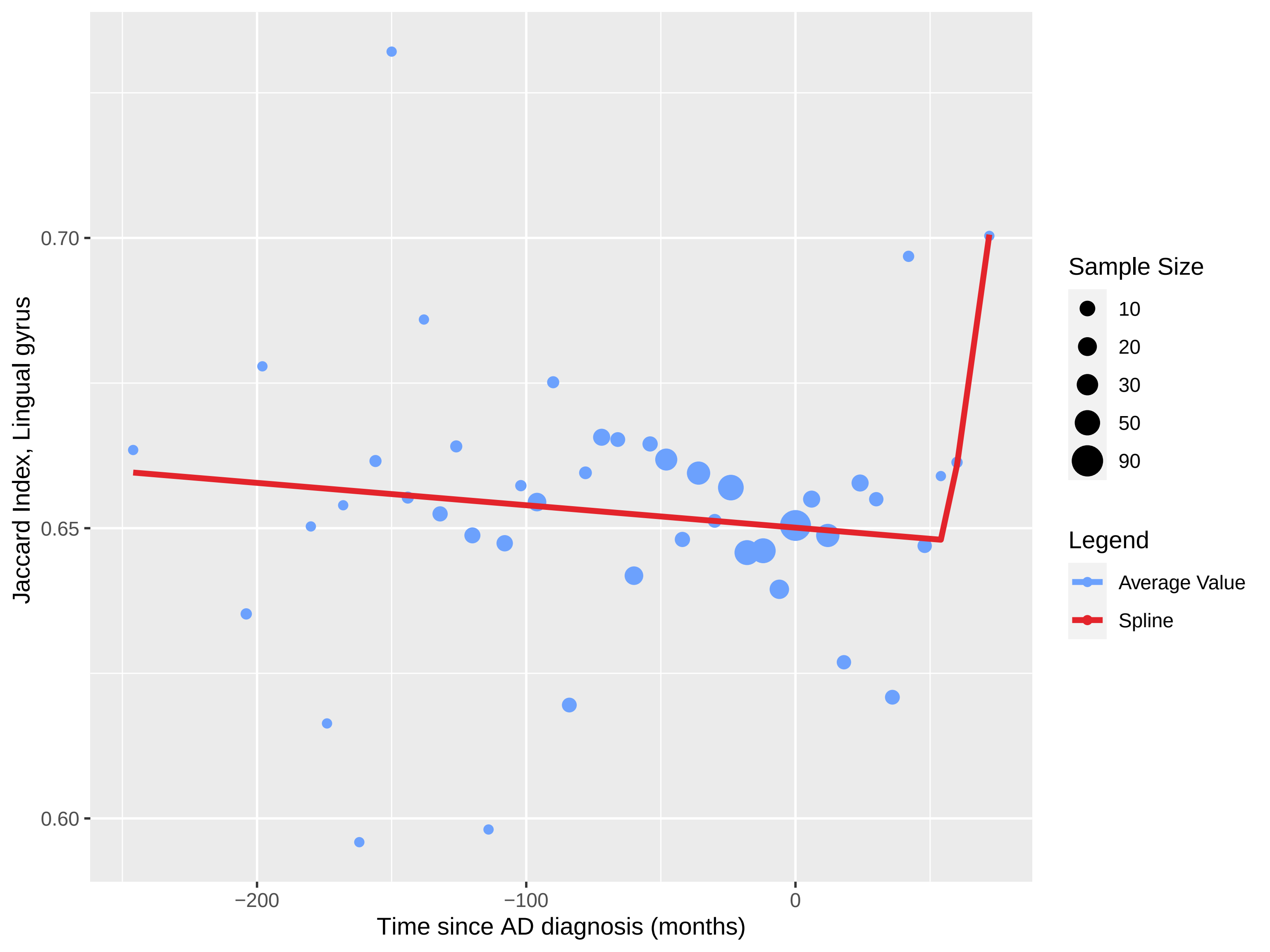

Jaccard Index, Lingual gyrus
0.70
0.65
0.60
−200
−100
0
Time since AD diagnosis (months)
Sample Size
10
20
30
50
90
Legend
Average Value
Spline

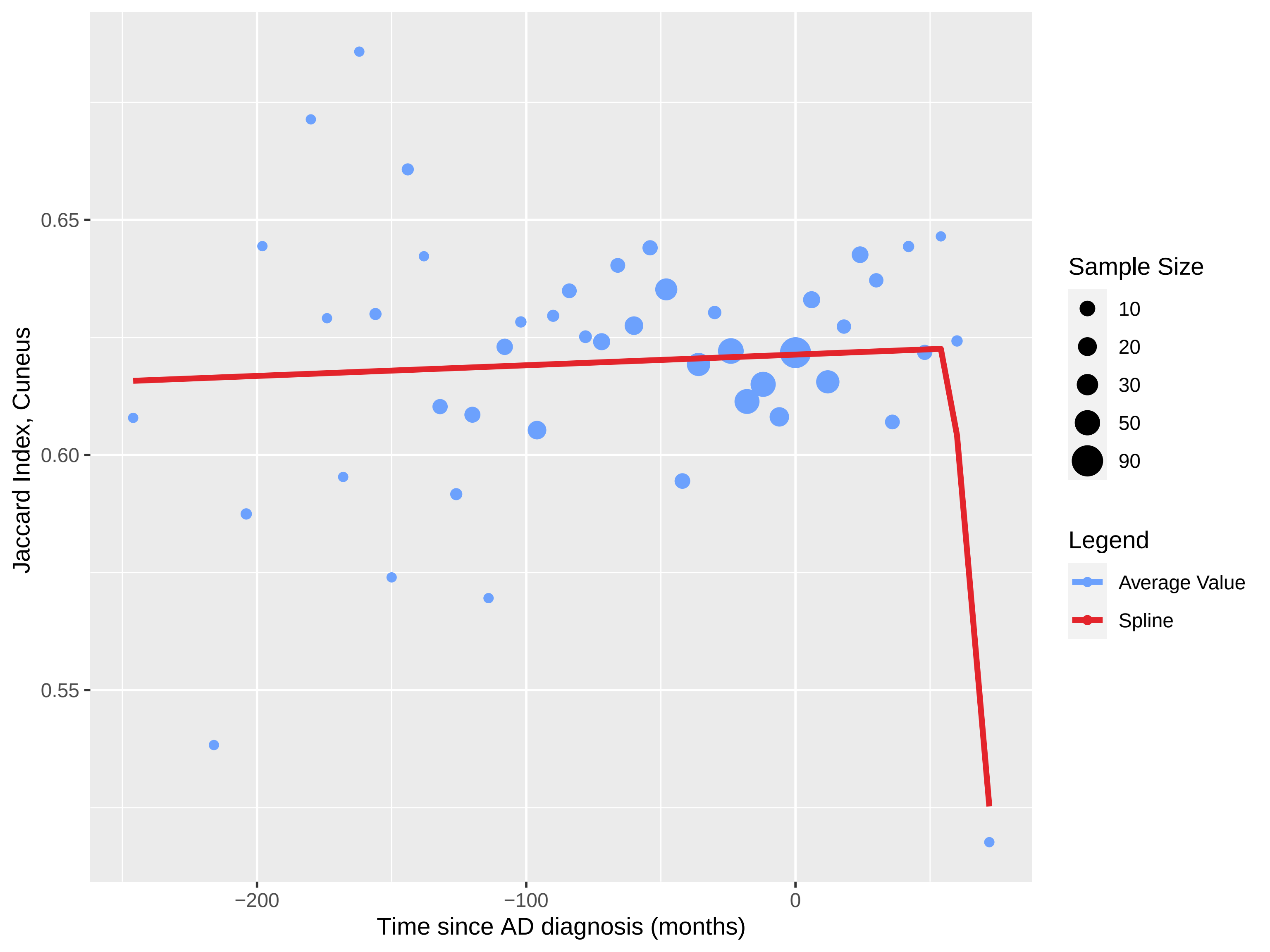

0.65
0.60
0.55
−200
−100
0
Time since AD diagnosis (months)
Jaccard Index, Cuneus
Sample Size
10
20
30
50
90
Legend
Average Value
Spline

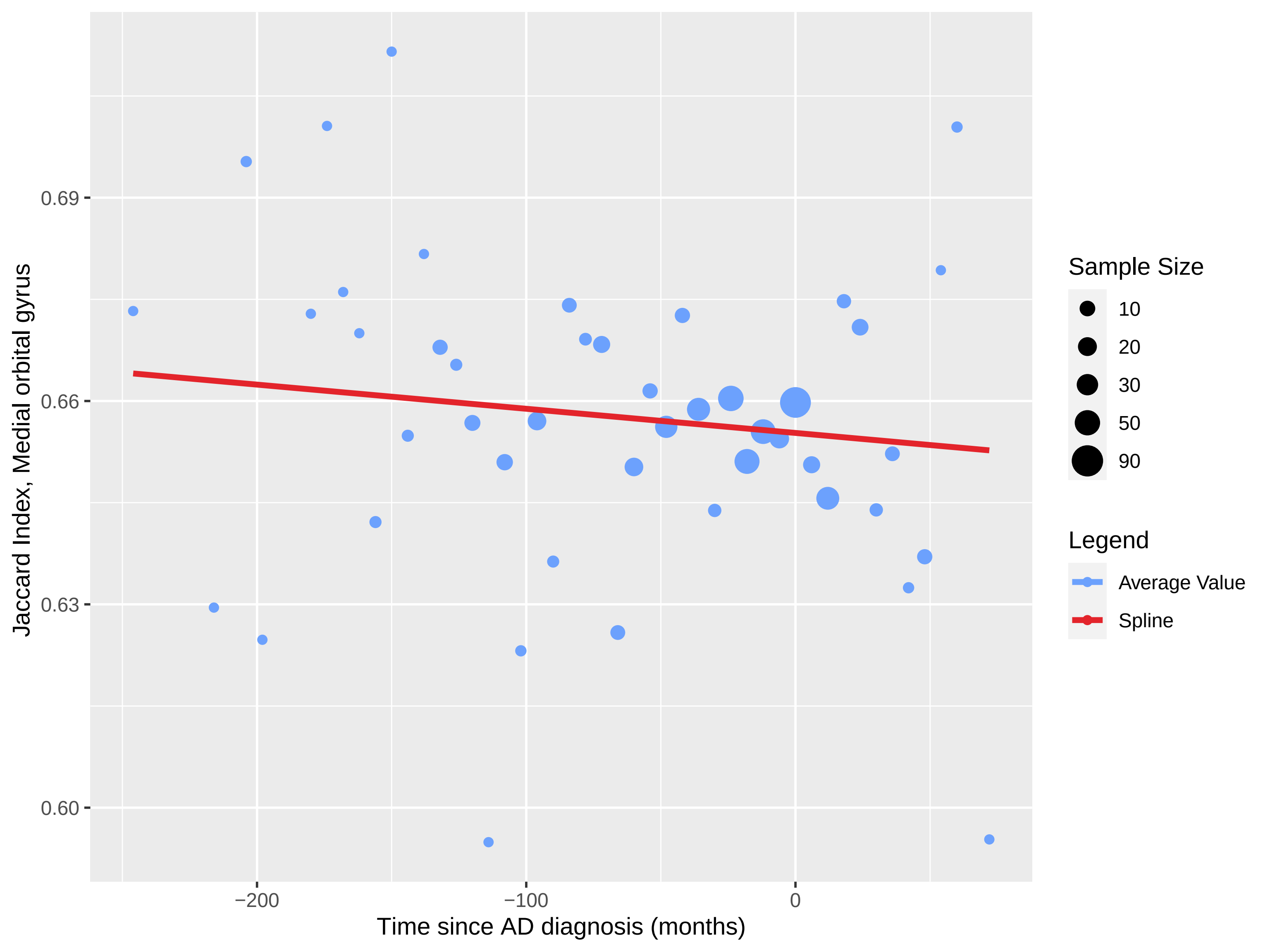

Jaccard Index, Medial orbital gyrus
0.69
0.66
0.63
0.60
−200
−100
0
Time since AD diagnosis (months)
Sample Size
10
20
30
50
90
Legend
Average Value
Spline

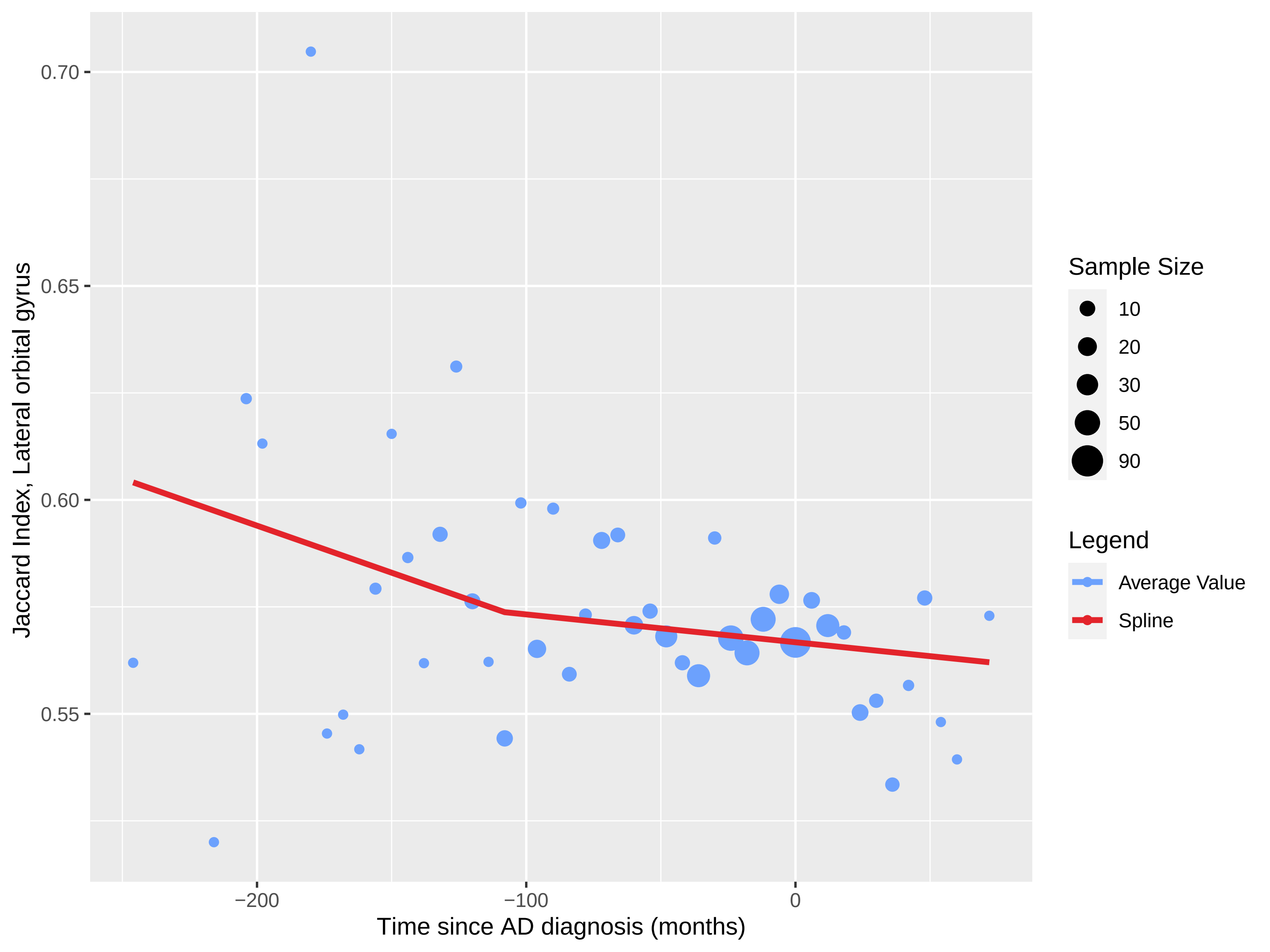

Jaccard Index, Lateral orbital gyrus
0.70
0.65
0.60
0.55
−200
−100
0
Time since AD diagnosis (months)
Sample Size
10
20
30
50
90
Legend
Average Value
Spline

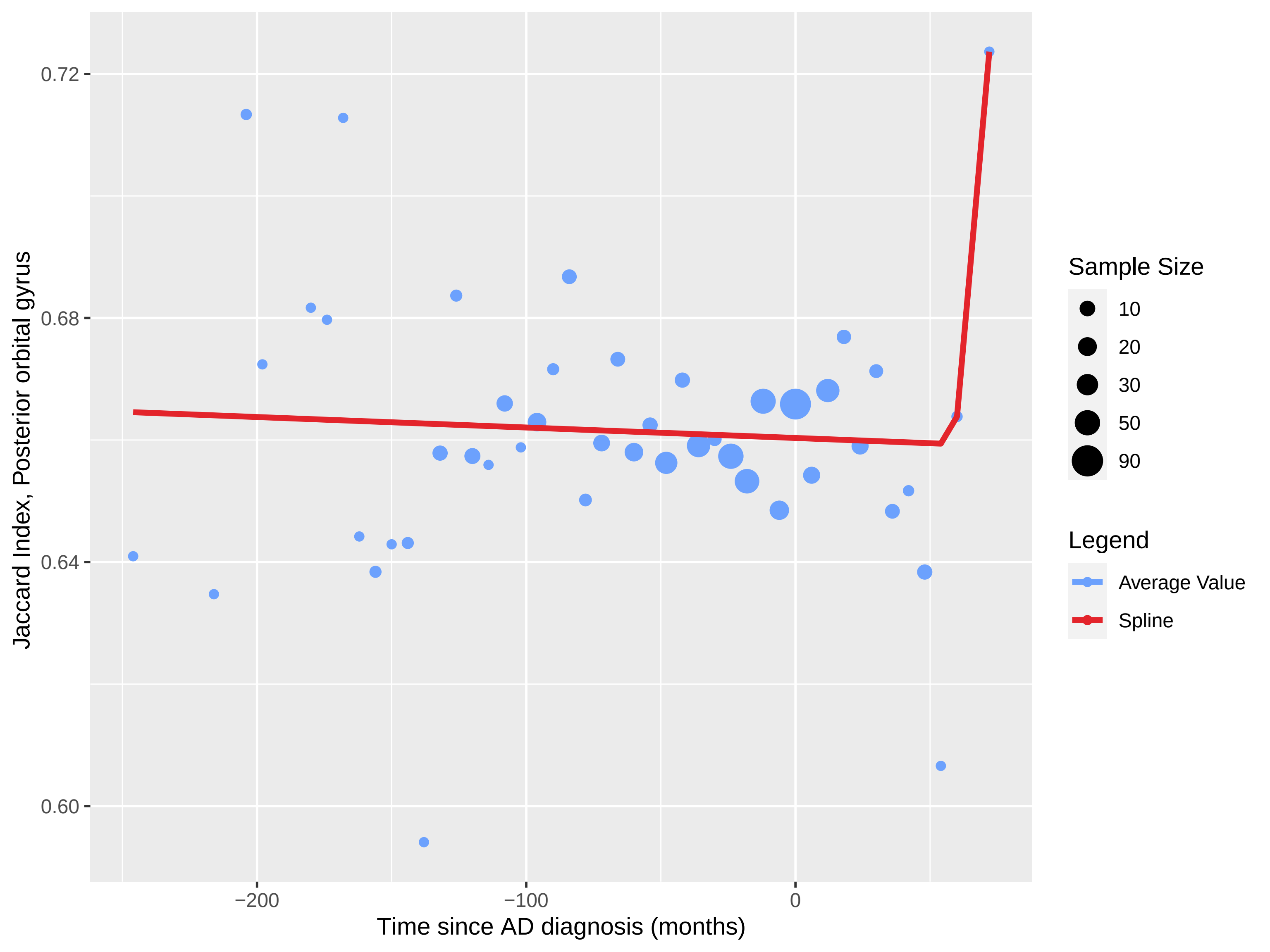

Jaccard Index, Posterior orbital gyrus
0.72
0.68
0.64
0.60
−200
−100
0
Time since AD diagnosis (months)
Sample Size
10
20
30
50
90
Legend
Average Value
Spline

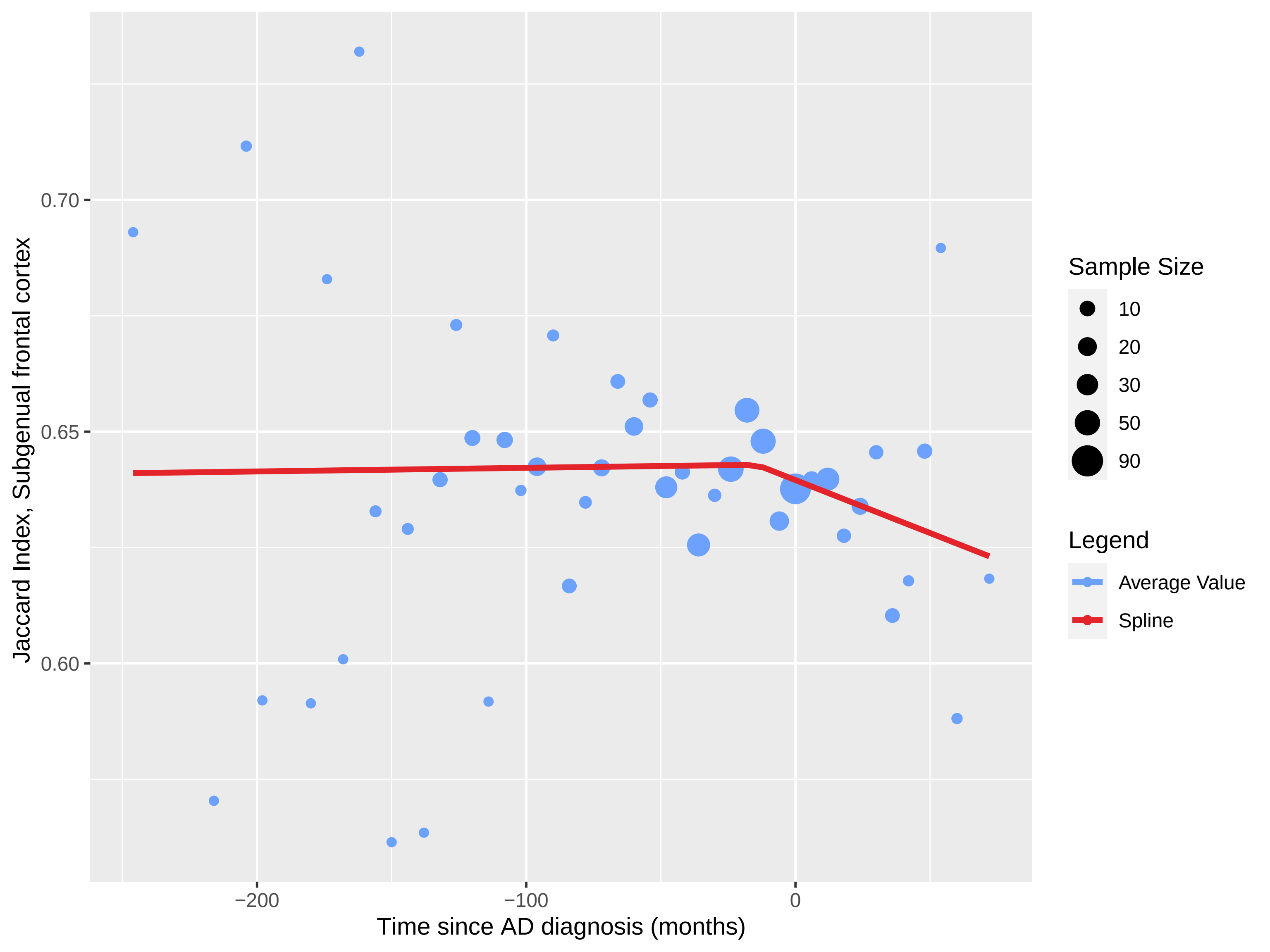

Jaccard Index, Subgenual frontal cortex
0.70
0.65
0.60
−200
−100
0
Time since AD diagnosis (months)
Sample Size
10
20
30
50
90
Legend
Average Value
Spline

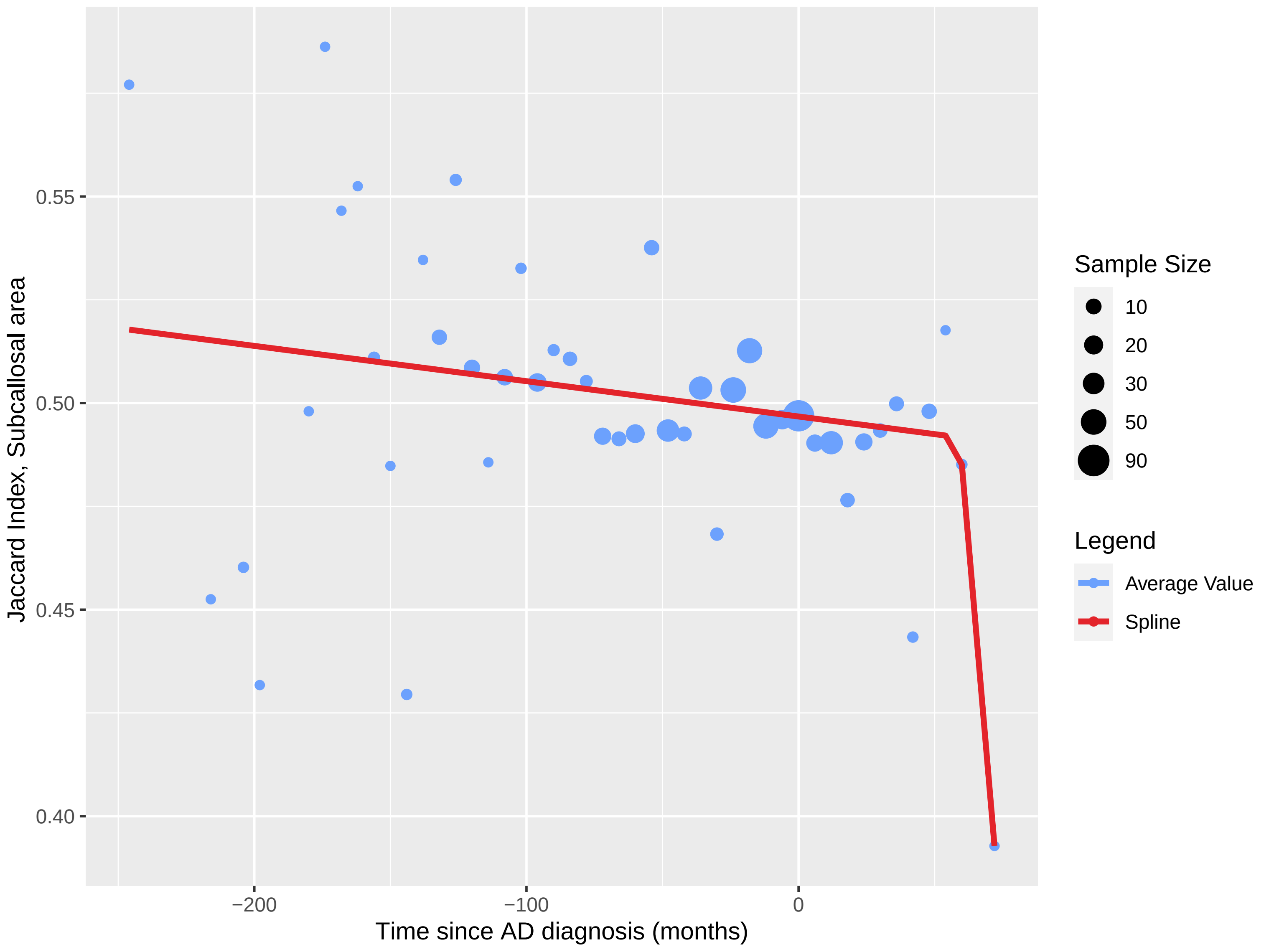

Jaccard Index, Subcallosal area
0.55
0.50
0.45
0.40
−200
−100
0
Time since AD diagnosis (months)
Sample Size
10
20
30
50
90
Legend
Average Value
Spline

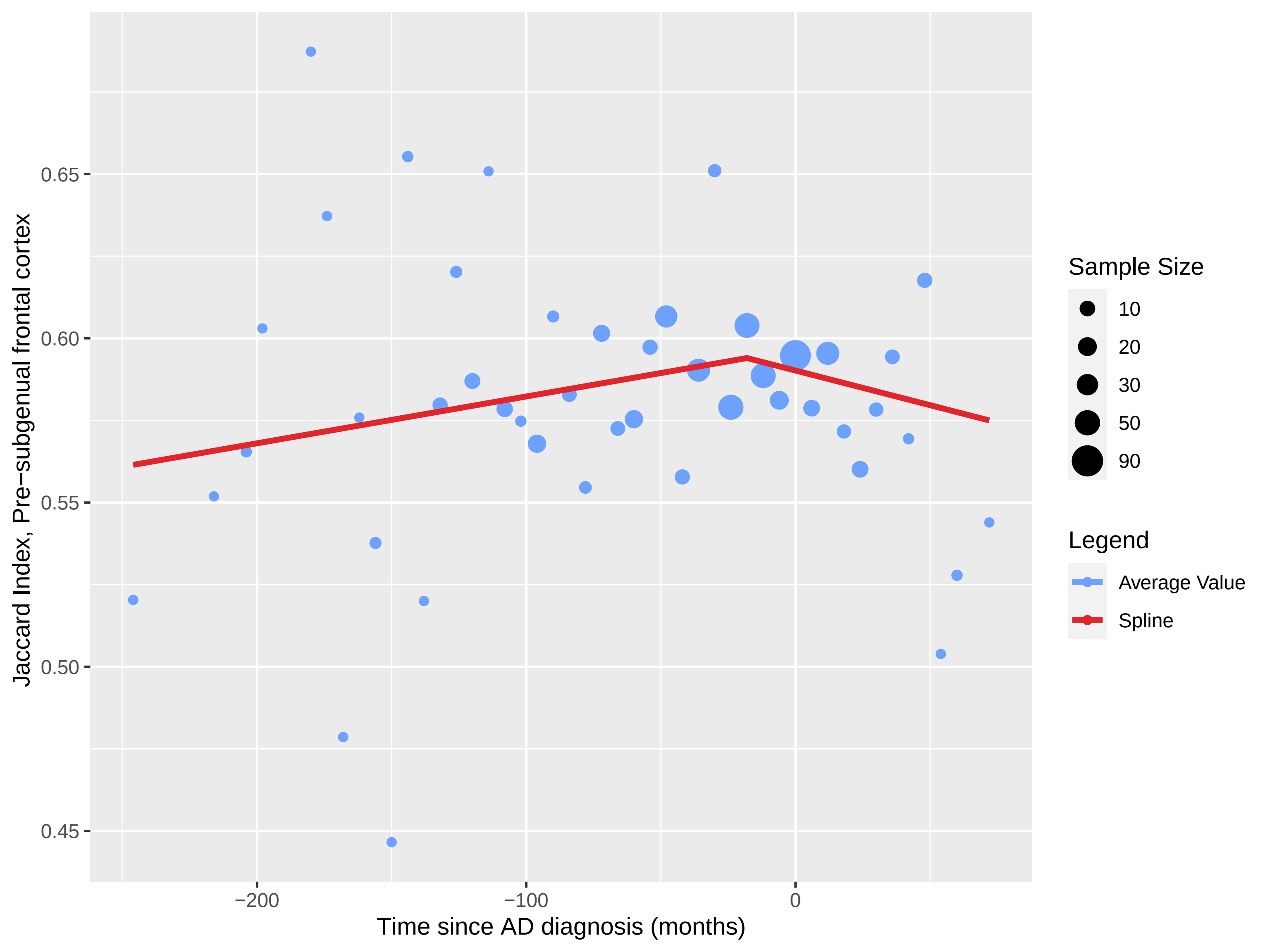

Jaccard Index, Pre−subgenual frontal cortex
0.65
0.60
0.55
0.50
0.45
−200
−100
0
Time since AD diagnosis (months)
Sample Size
10
20
30
50
90
Legend
Average Value
Spline

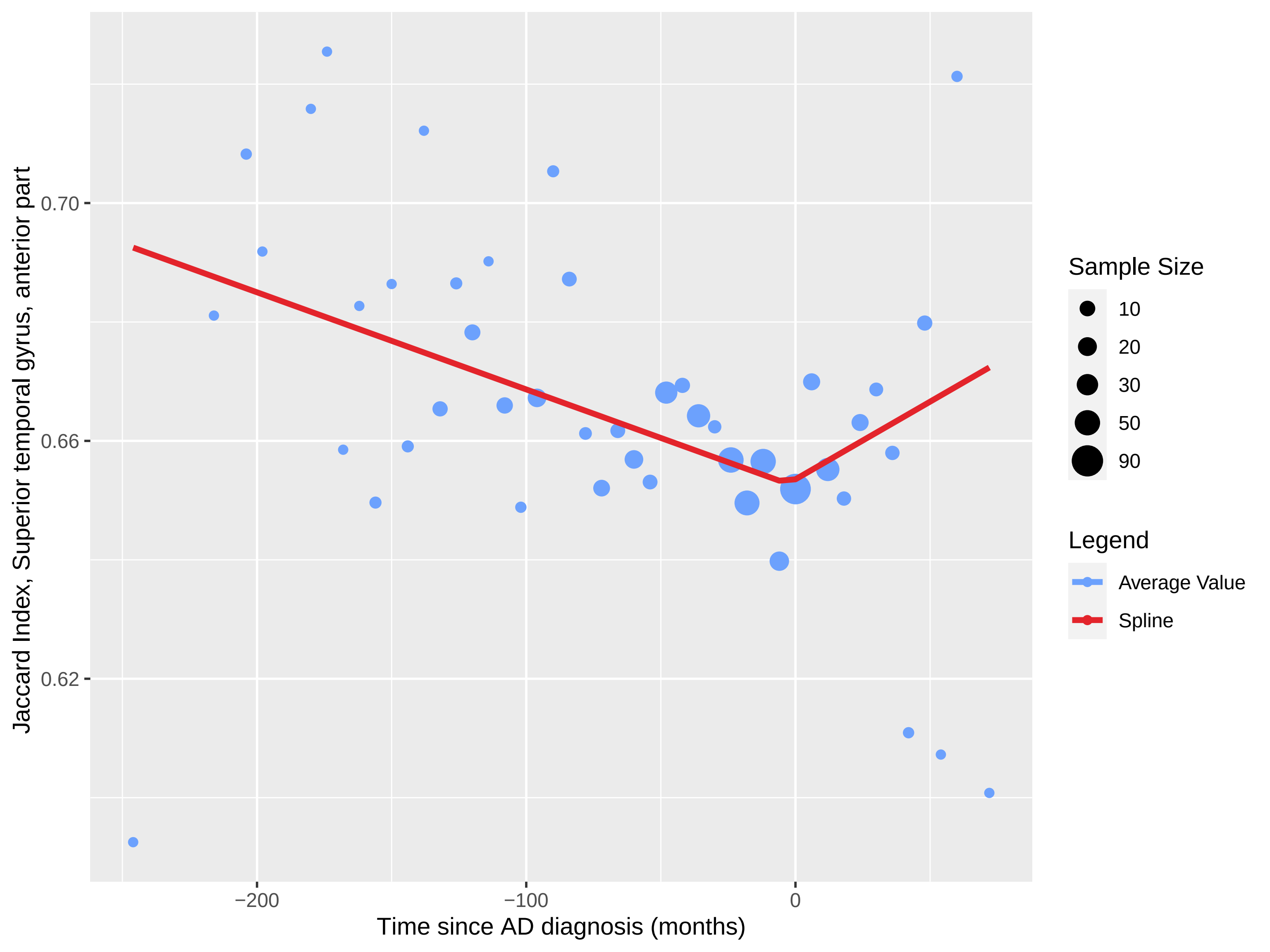

Jaccard Index, Superior temporal gyrus, anterior part
0.70
0.66
0.62
−200
−100
0
Time since AD diagnosis (months)
Sample Size
10
20
30
50
90
Legend
Average Value
Spline

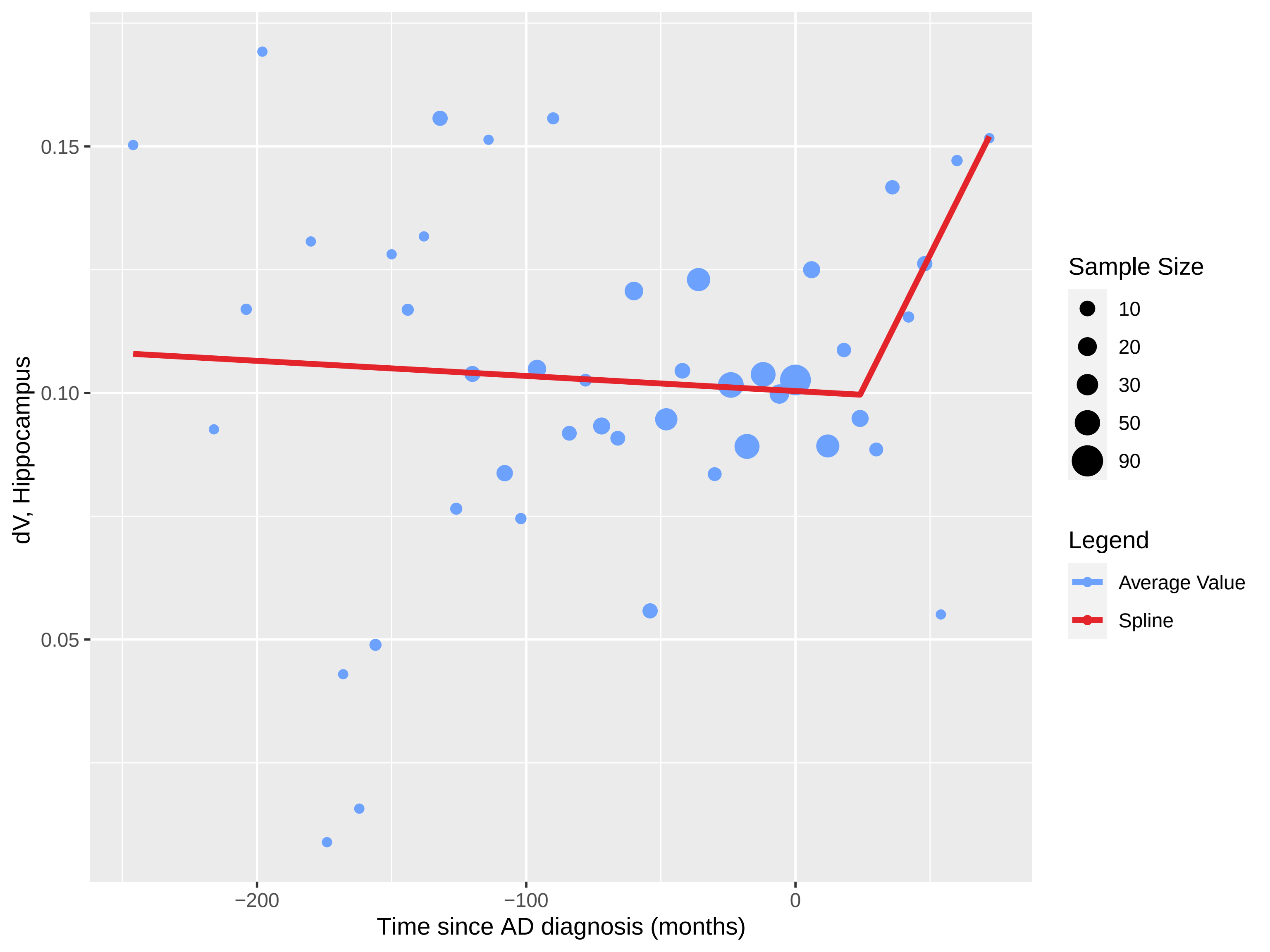

0.15
0.10
0.05
dV, Hippocampus
−200
−100
0
Time since AD diagnosis (months)
Sample Size
10
20
30
50
90
Legend
Average Value
Spline

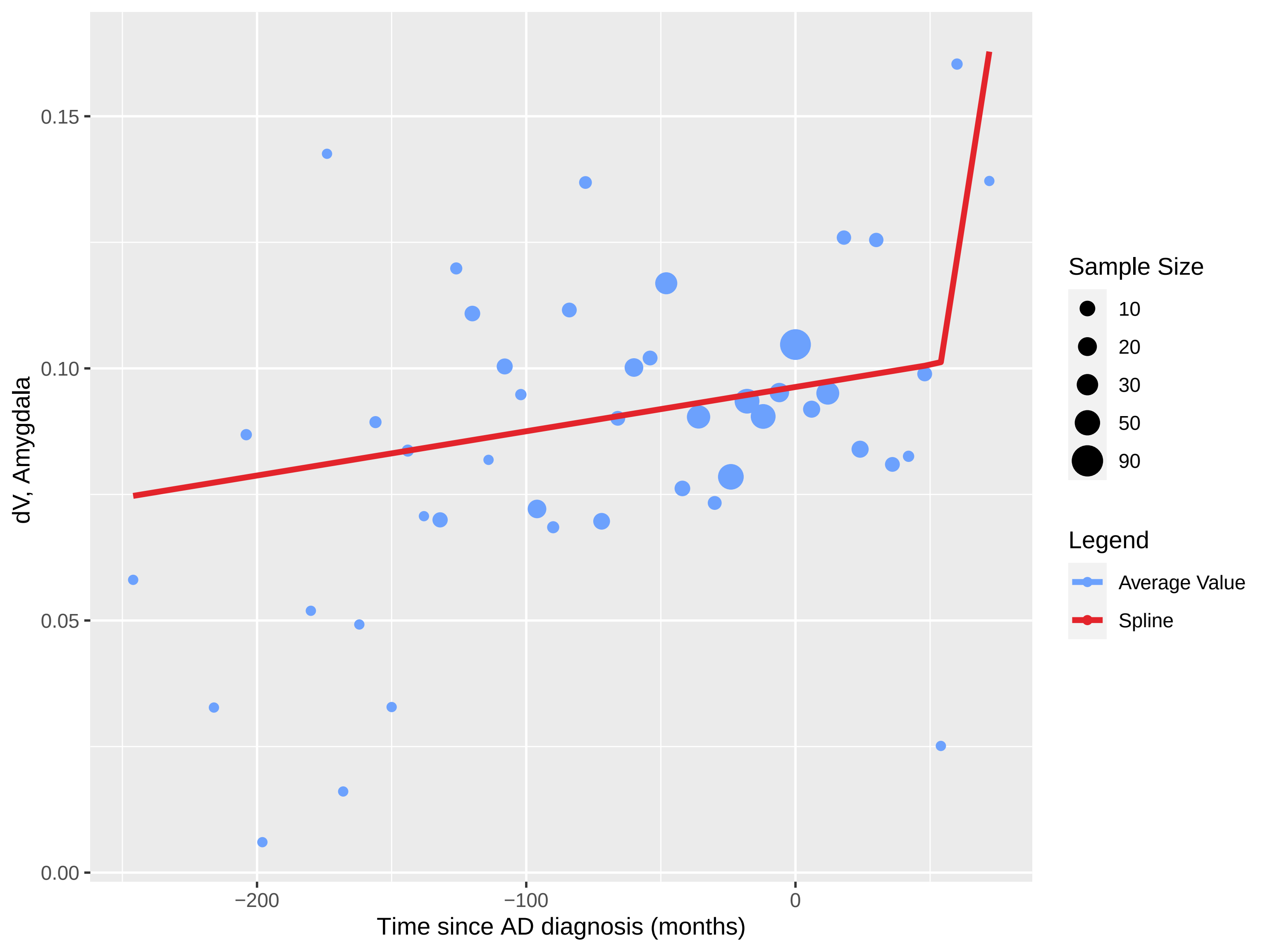
0.15
0.10
0.05
0.00
−200
−100
0
dV, Amygdala
Time since AD diagnosis (months)
Sample Size
10
20
30
50
90
Legend
Average Value
Spline

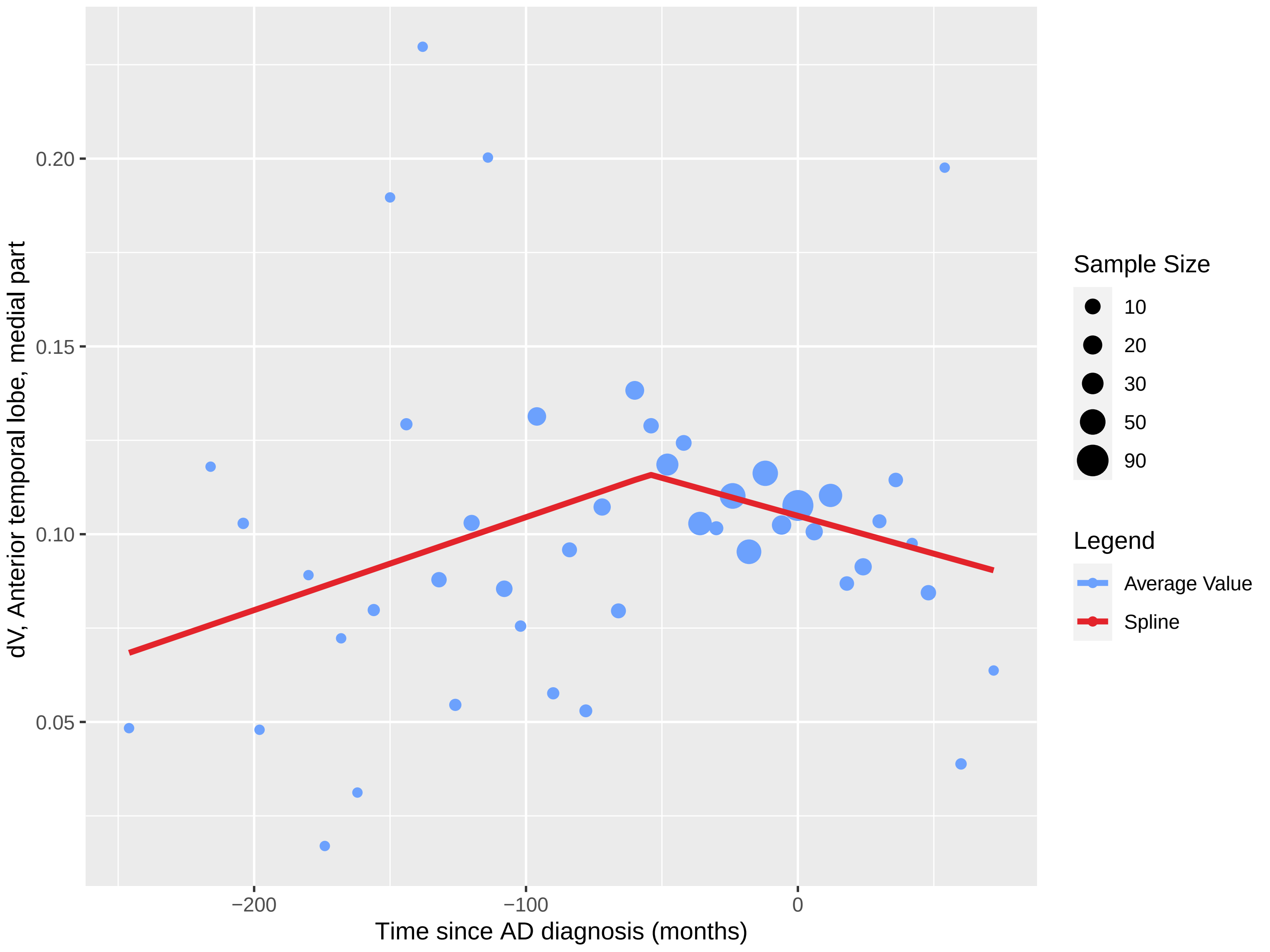

dV, Anterior temporal lobe, medial part
0.20
0.15
0.10
0.05
−200
−100
0
Time since AD diagnosis (months)
Sample Size
10
20
30
50
90
Legend
Average Value
Spline

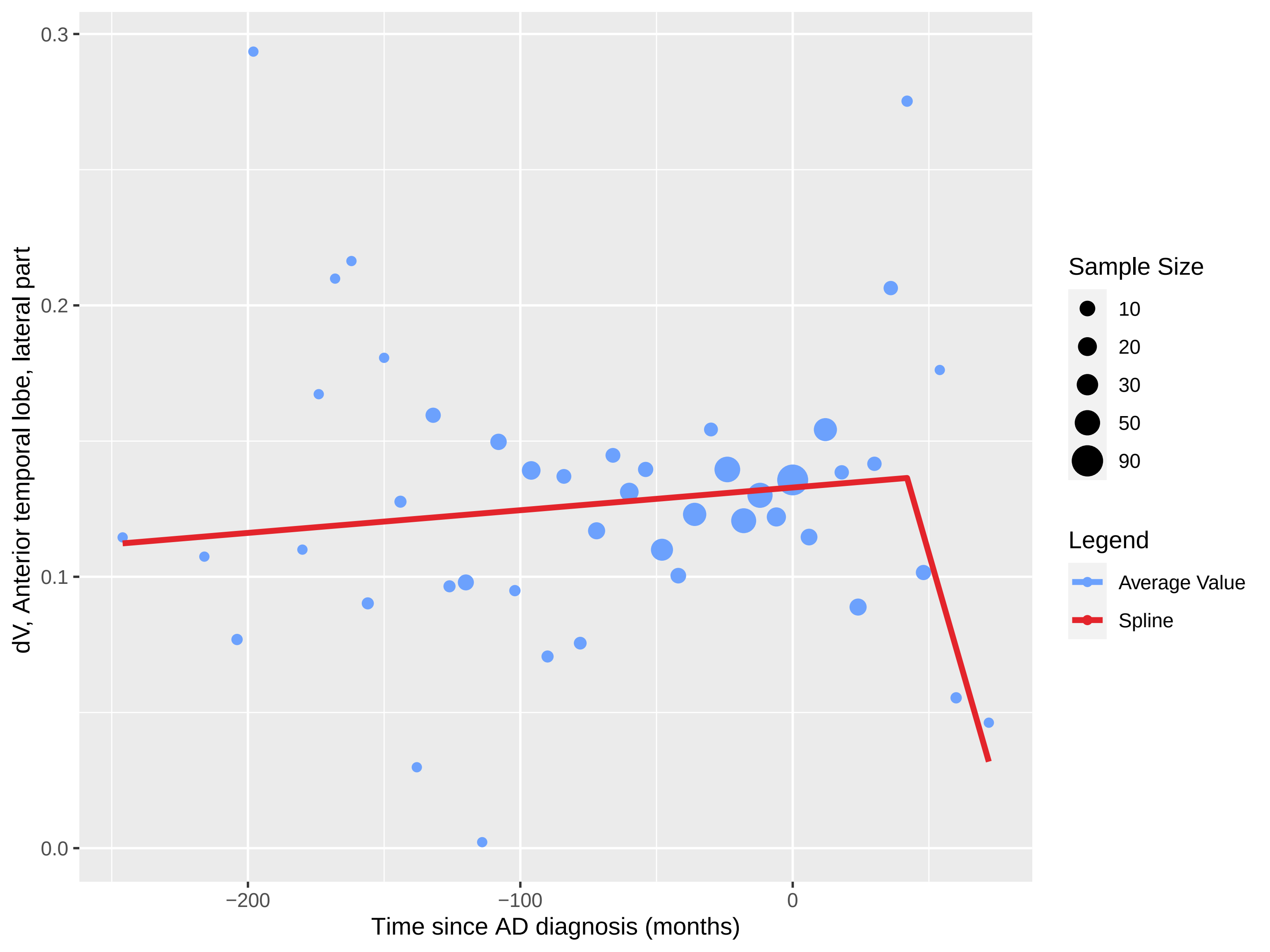

dV, Anterior temporal lobe, lateral part
0.3
0.2
0.1
0.0
−200
−100
0
Time since AD diagnosis (months)
Sample Size
10
20
30
50
90
Legend
Average Value
Spline

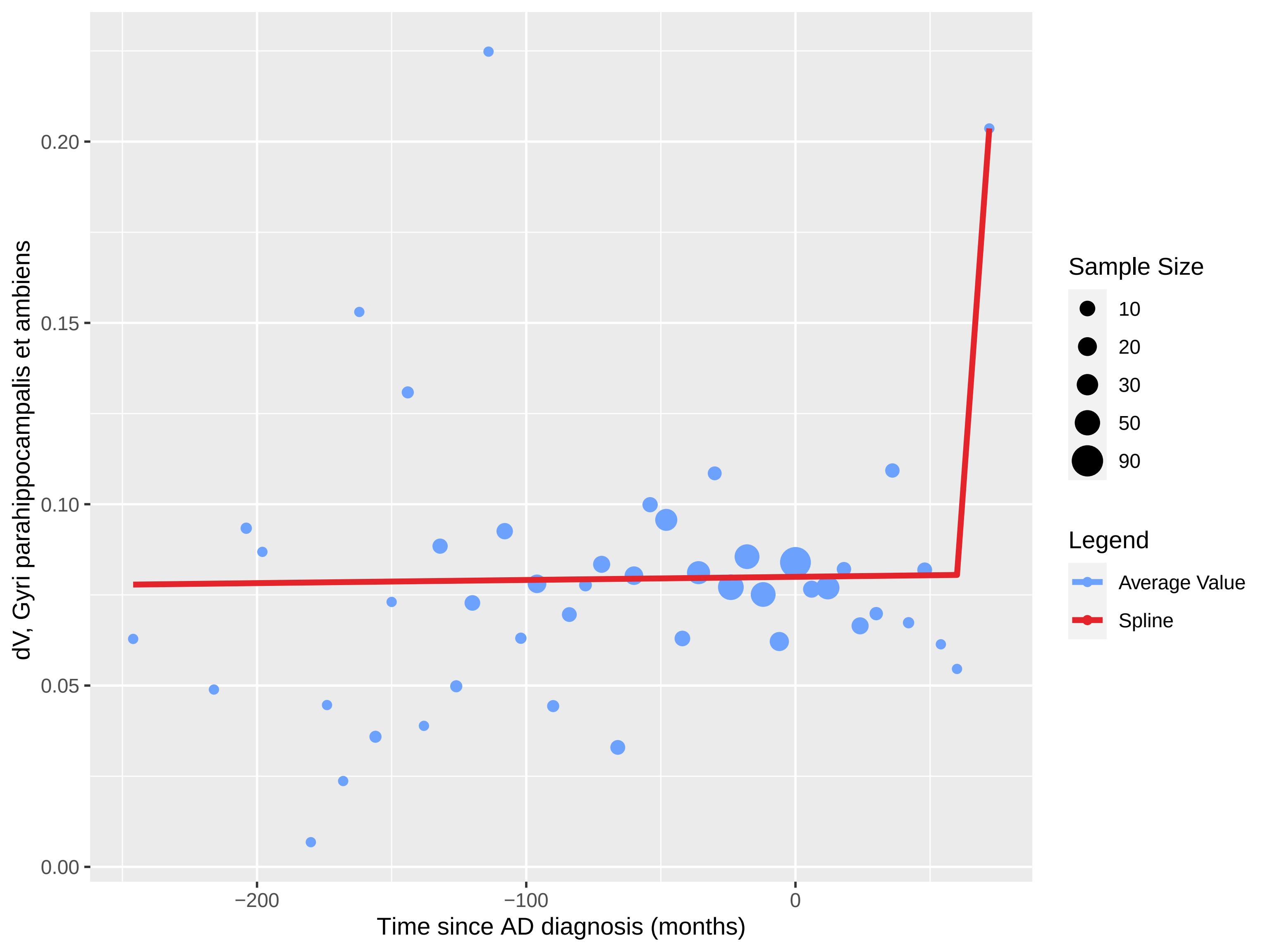

dV, Gyri parahippocampalis et ambiens
0.00
0.05
0.10
0.15
0.20
−200
−100
0
Time since AD diagnosis (months)
Sample Size
10
20
30
50
90
Legend
Average Value
Spline

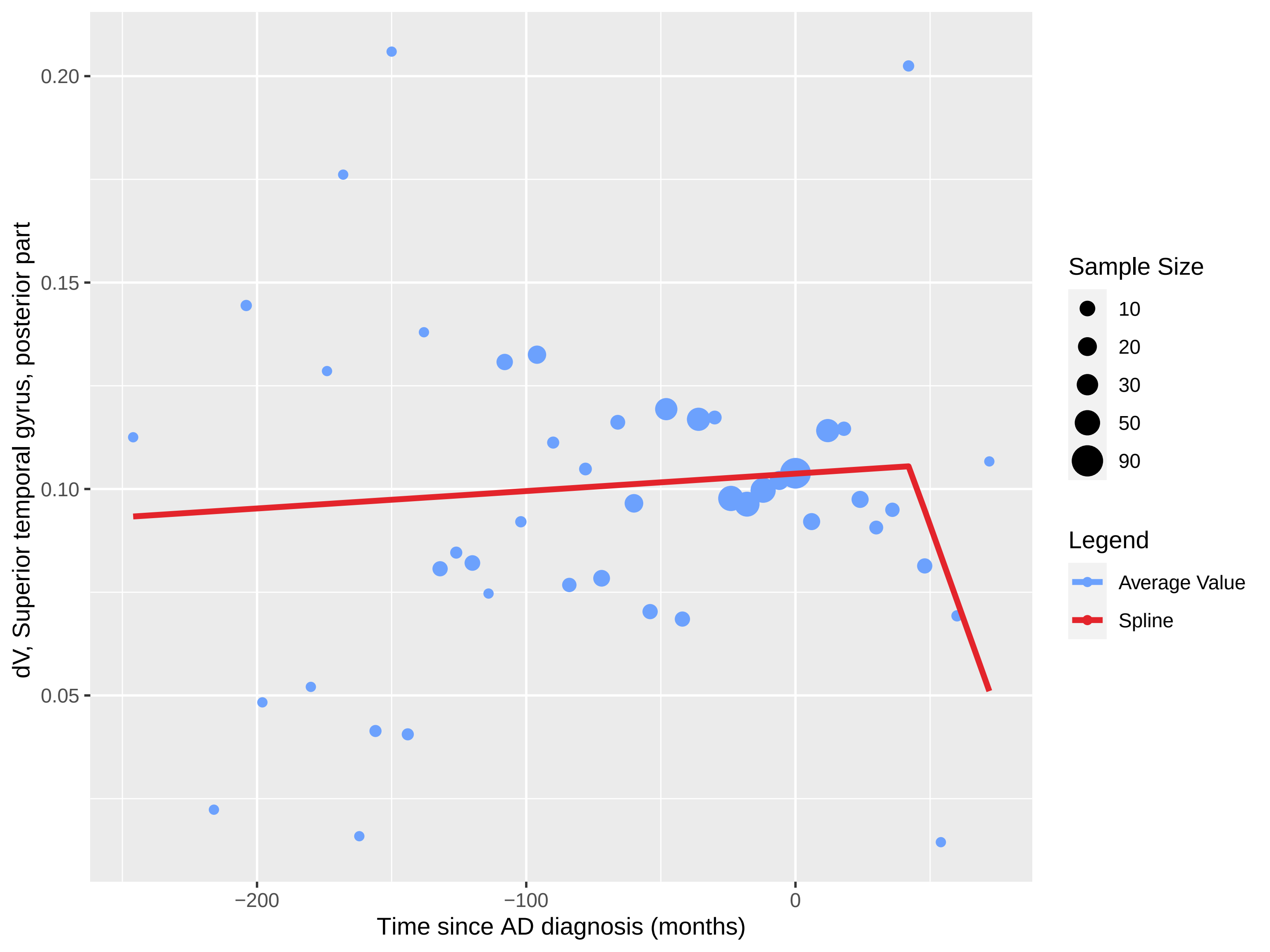

dV, Superior temporal gyrus, posterior part
0.20
0.15
0.10
0.05
−200
−100
0
Time since AD diagnosis (months)
Sample Size
10
20
30
50
90
Legend
Average Value
Spline

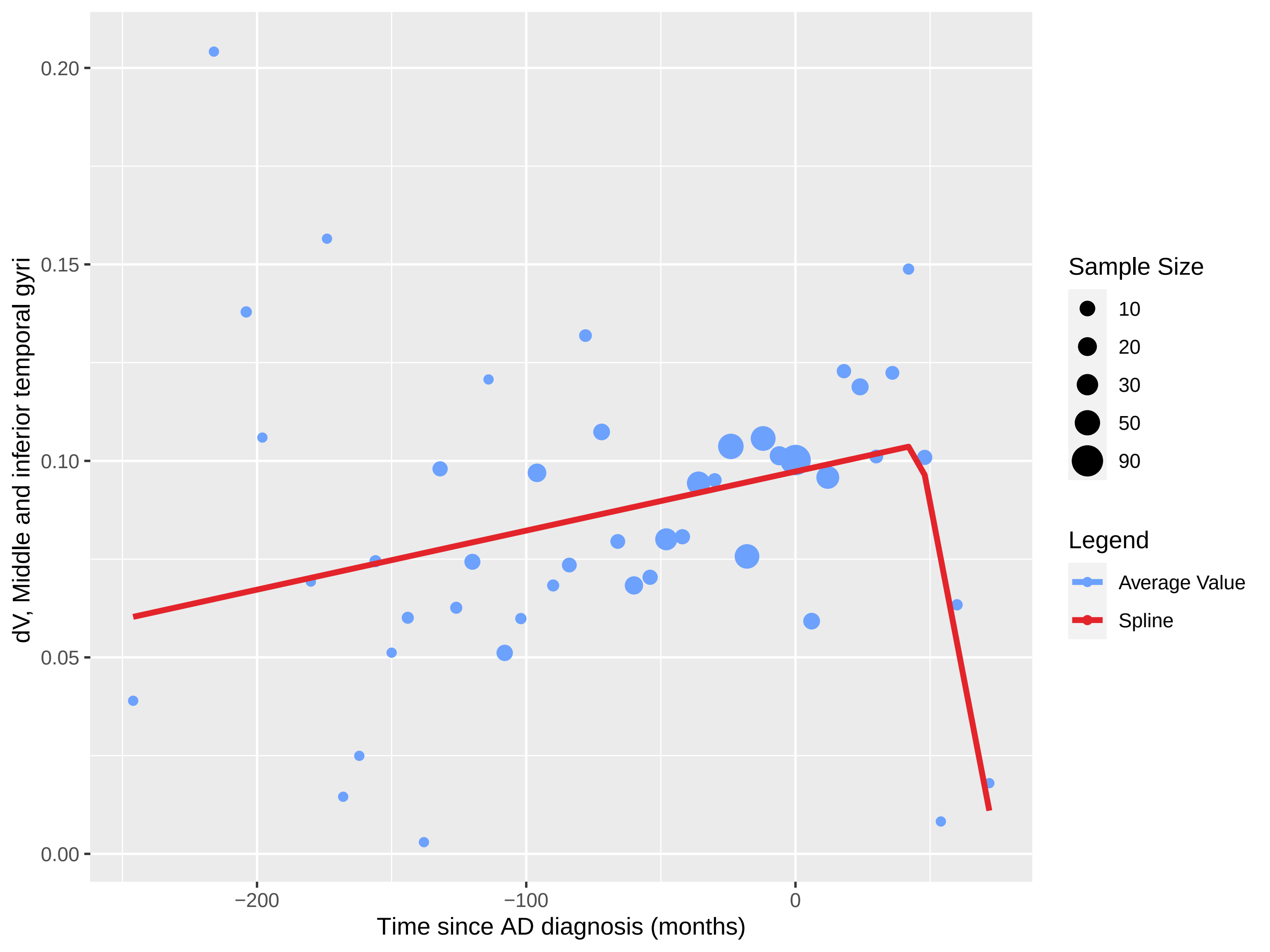

0.20
0.15
0.10
0.05
0.00
dV, Middle and inferior temporal gyri
−200
−100
0
Time since AD diagnosis (months)
Sample Size
10
20
30
50
90
Legend
Average Value
Spline

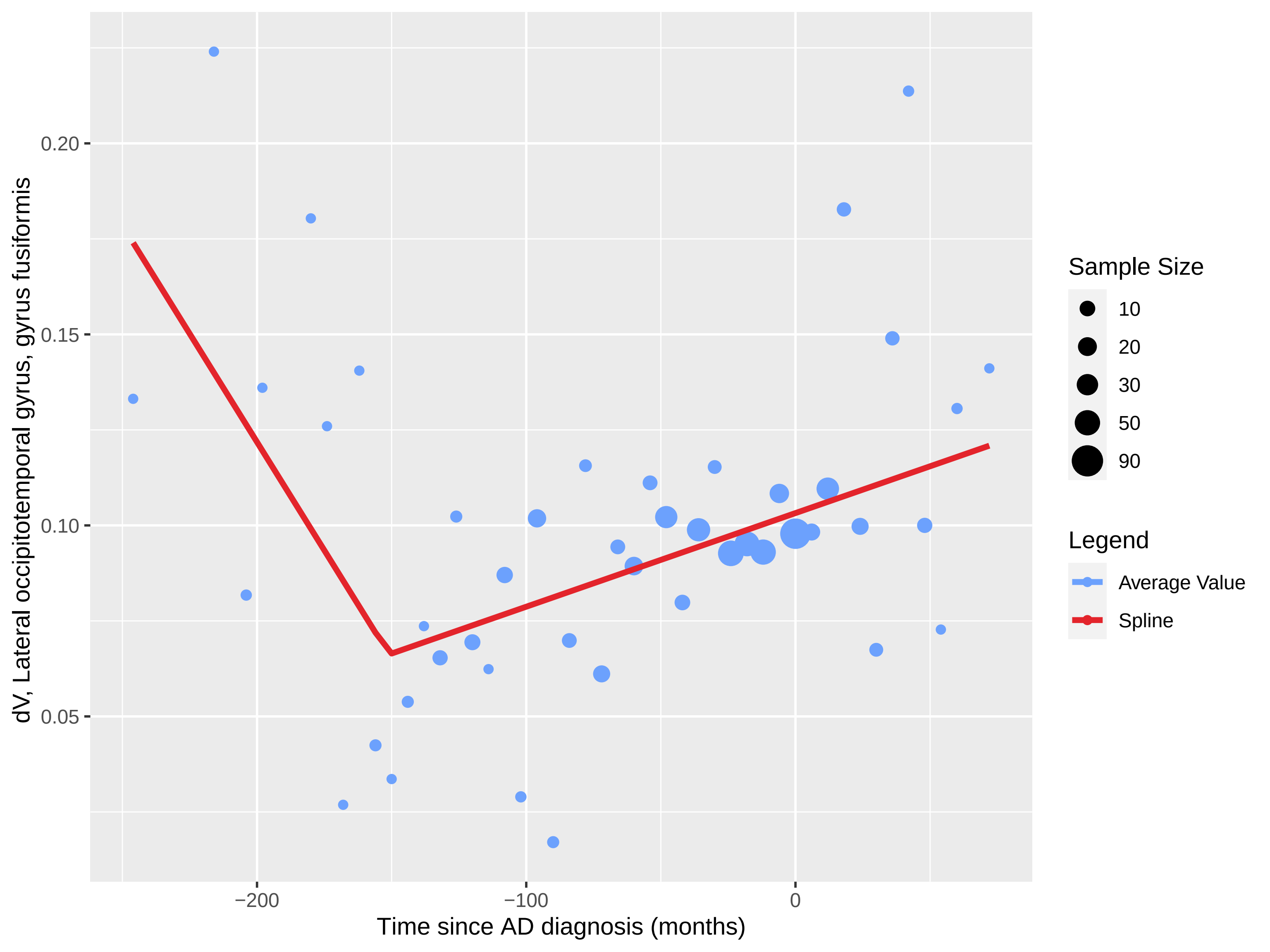

dV, Lateral occipitotemporal gyrus, gyrus fusiformis
0.20
0.15
0.10
0.05
−200
−100
0
Time since AD diagnosis (months)
Sample Size
10
20
30
50
90
Legend
Average Value
Spline

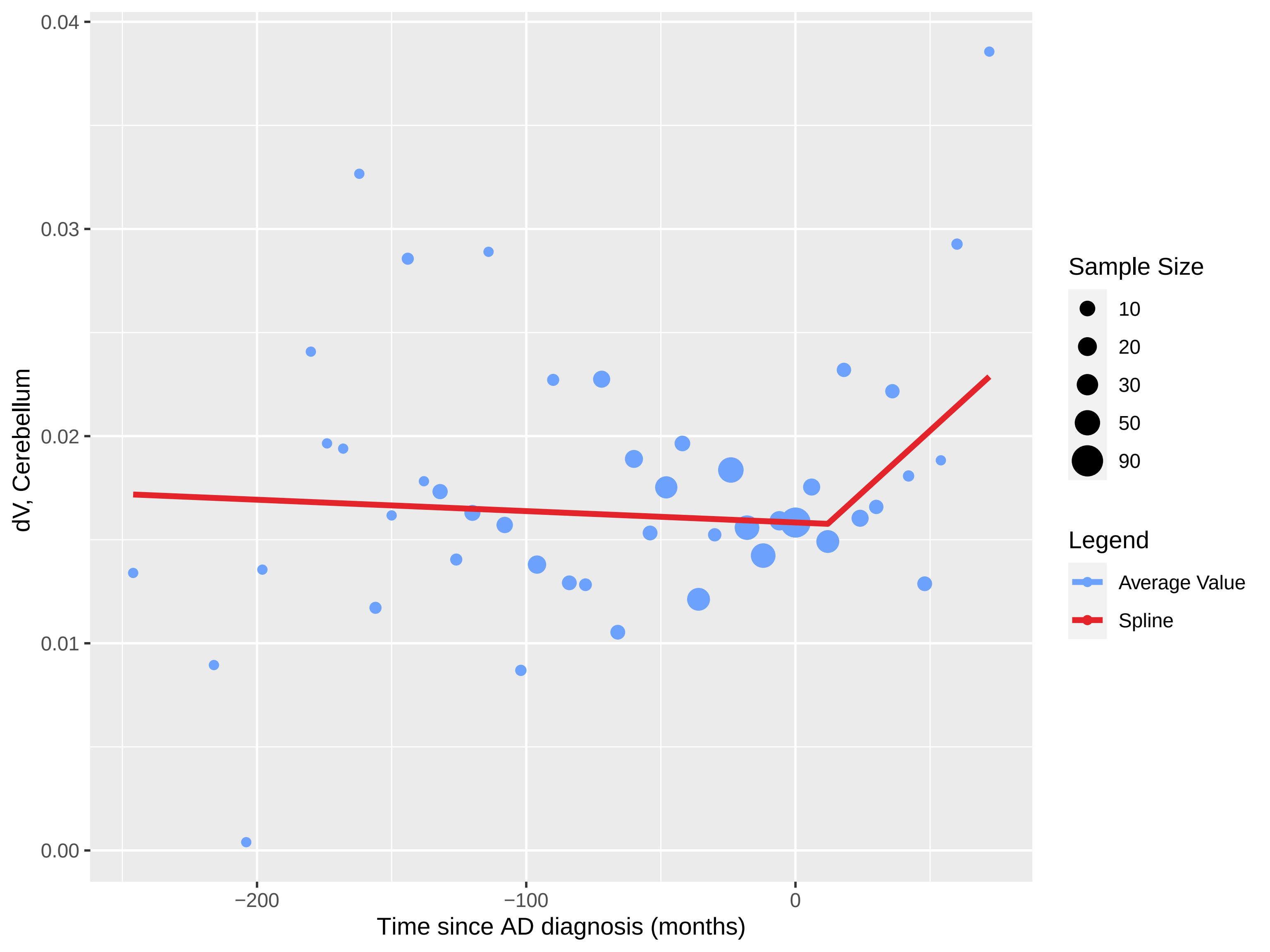

0.04
0.03
0.02
0.01
0.00
dV, Cerebellum
−200
−100
0
Time since AD diagnosis (months)
Sample Size
10
20
30
50
90
Legend
Average Value
Spline

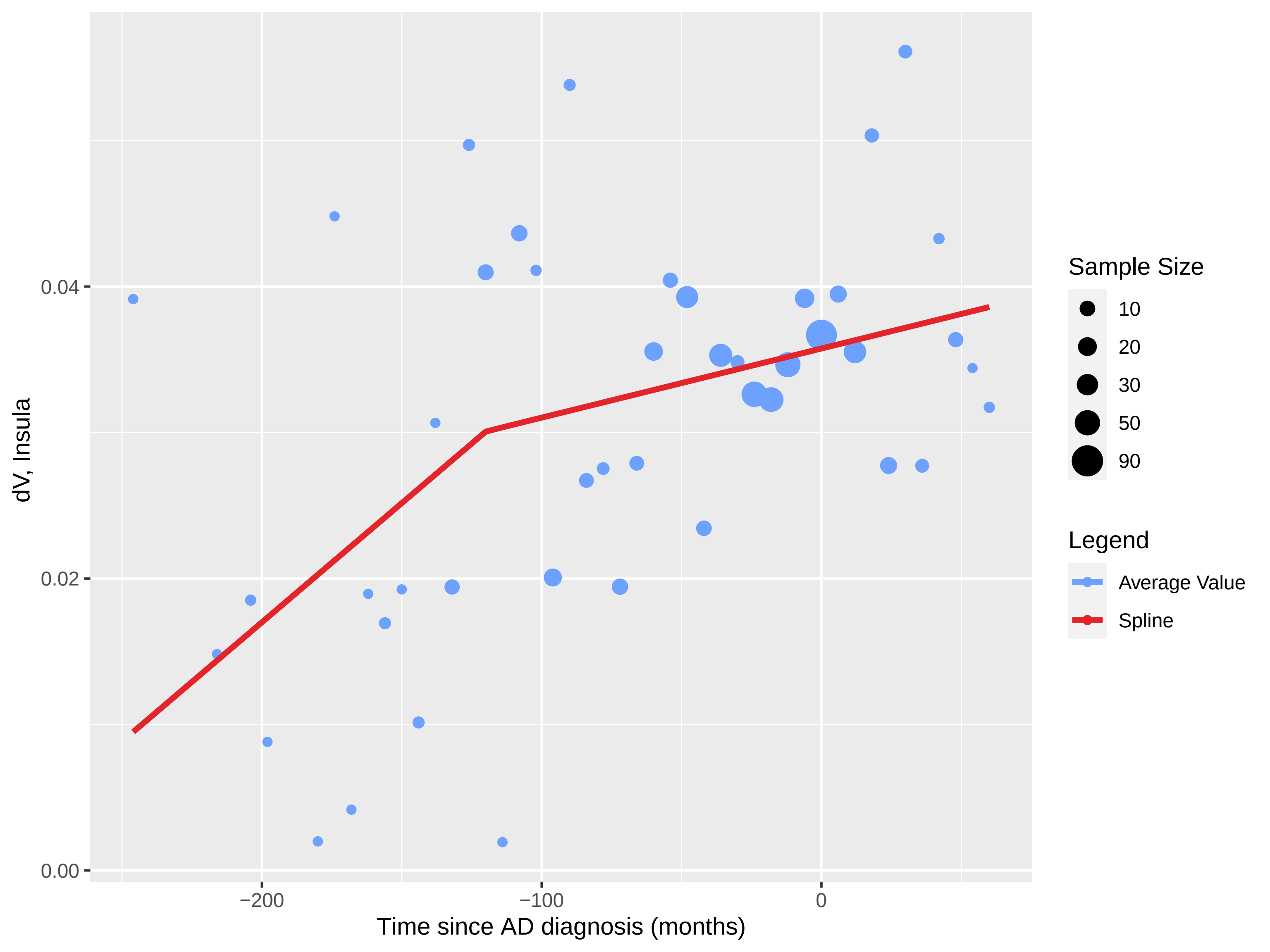

dV, Insula
0.04
0.02
0.00
−200
−100
0
Time since AD diagnosis (months)
Sample Size
10
20
30
50
90
Legend
Average Value
Spline

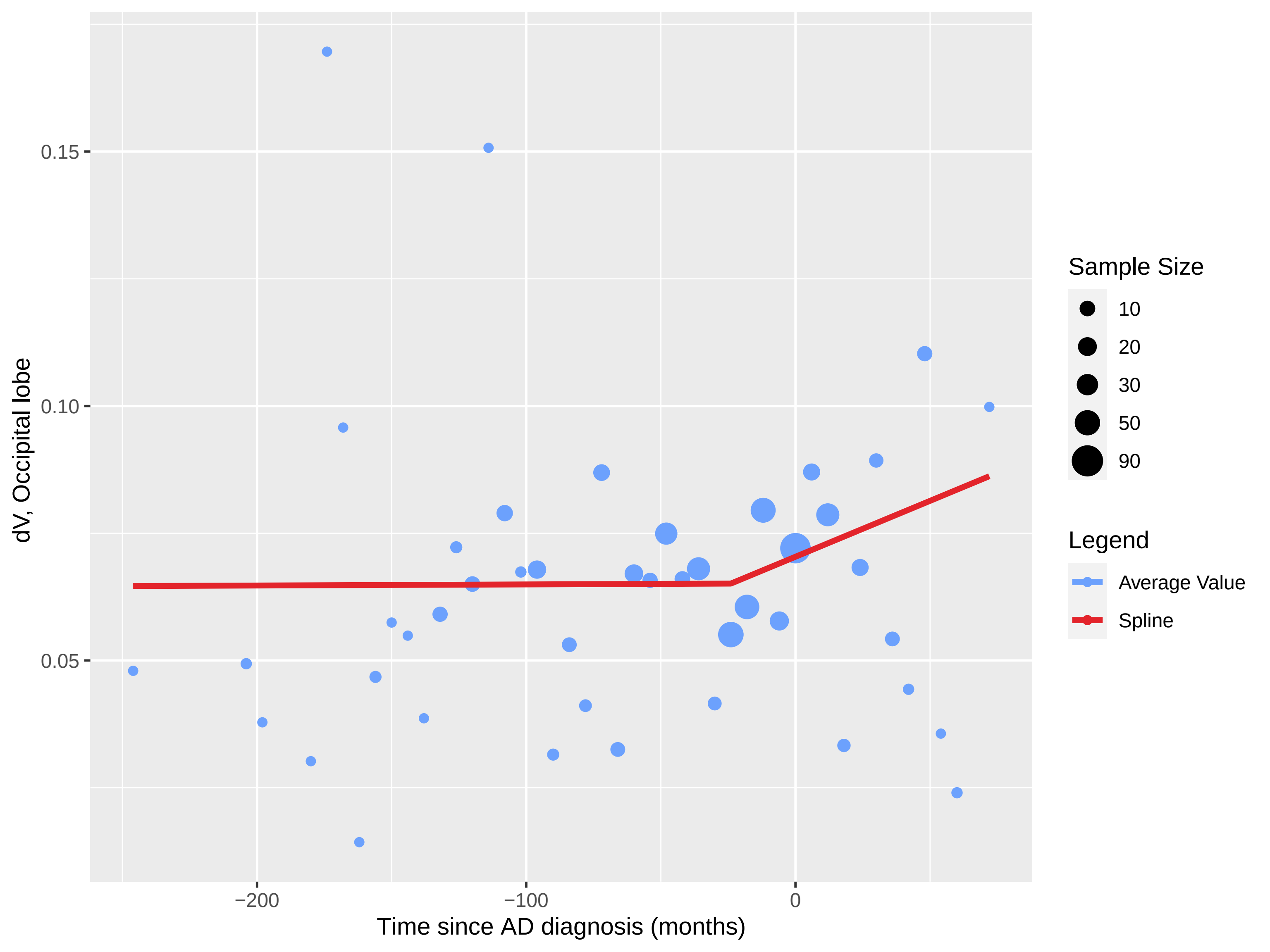

0.15
0.10
0.05
−200
−100
0
Time since AD diagnosis (months)
dV, Occipital lobe
Sample Size
10
20
30
50
90
Legend
Average Value
Spline

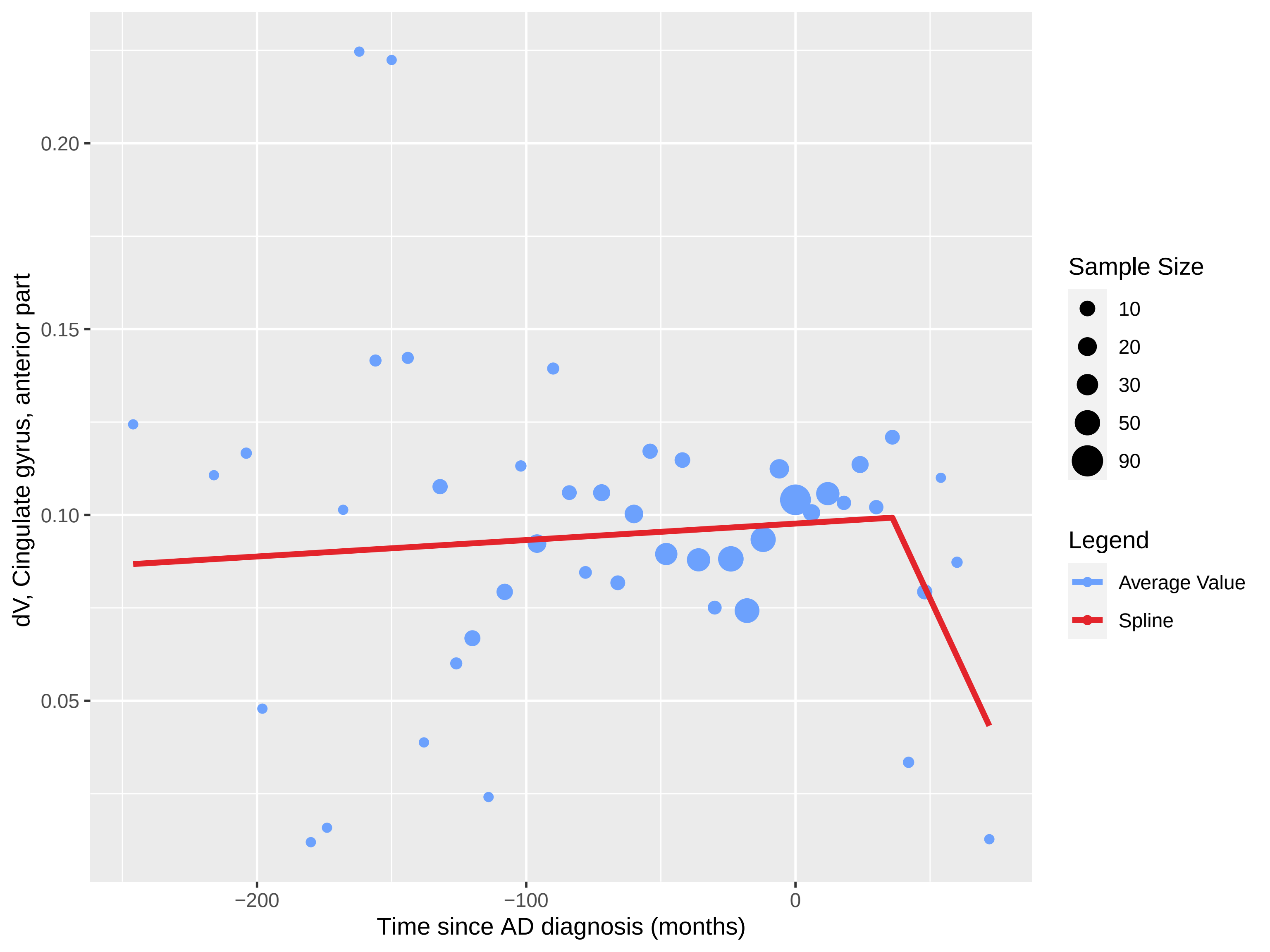

0.20
0.15
0.10
0.05
−200
−100
0
dV, Cingulate gyrus, anterior part
Time since AD diagnosis (months)
Sample Size
10
20
30
50
90
Legend
Average Value
Spline

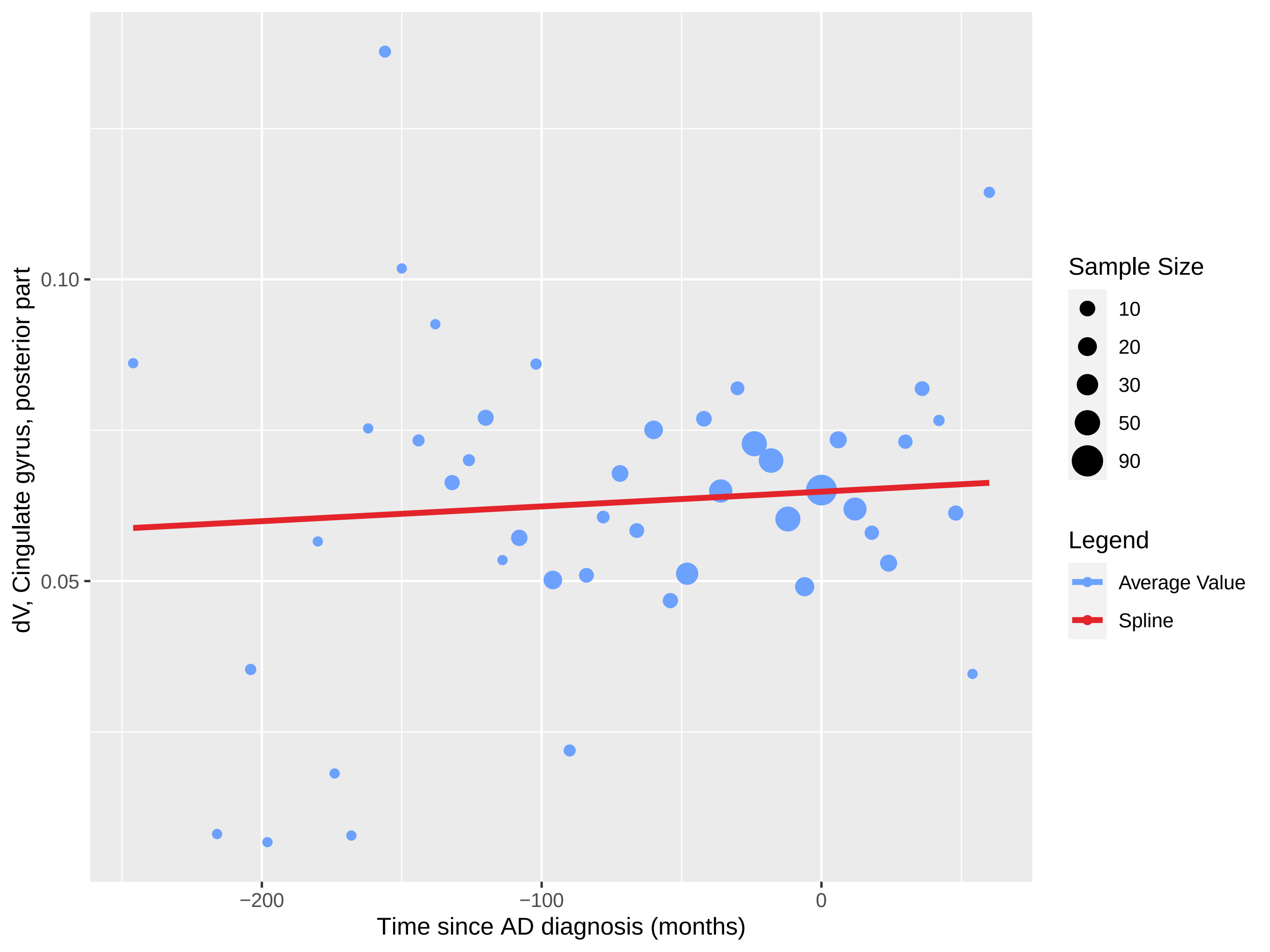

dV, Cingulate gyrus, posterior part
0.10
0.05
−200
−100
0
Time since AD diagnosis (months)
Sample Size
10
20
30
50
90
Legend
Average Value
Spline

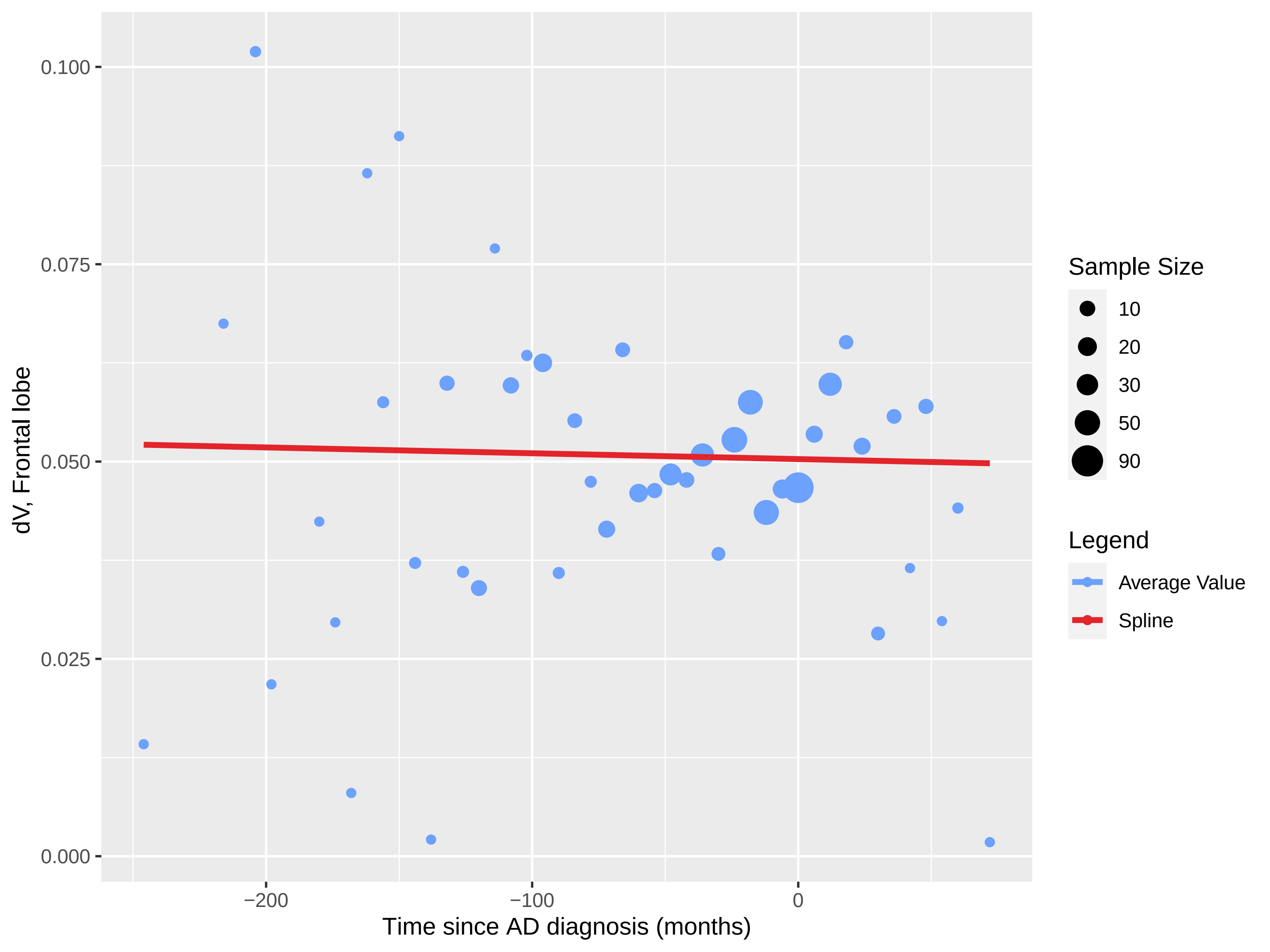

0.100
0.075
0.050
0.025
0.000
dV, Frontal lobe
−200
−100
0
Time since AD diagnosis (months)
Sample Size
10
20
30
50
90
Legend
Average Value
Spline

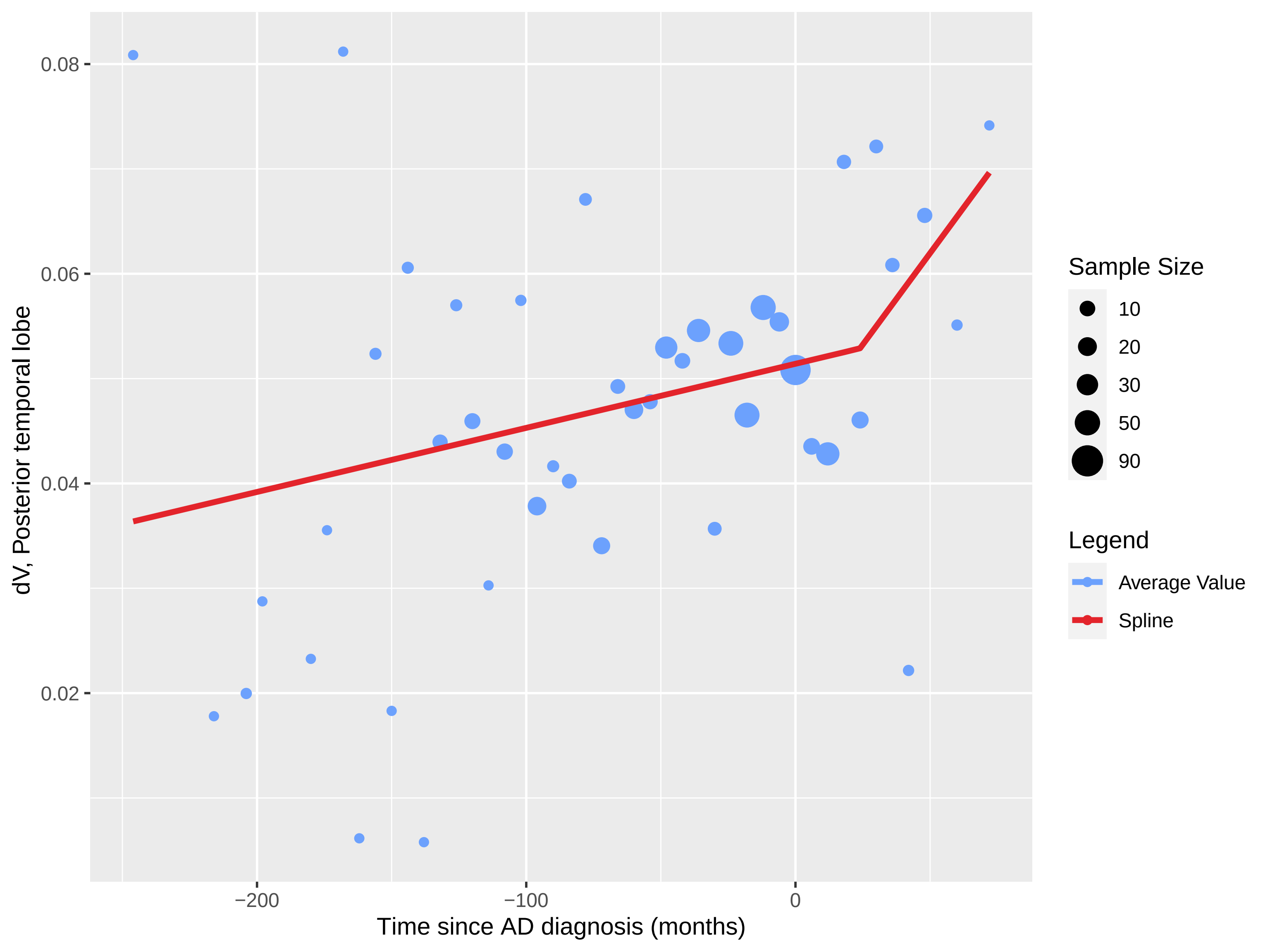

0.08
0.06
0.04
0.02
−200
−100
0
Time since AD diagnosis (months)
dV, Posterior temporal lobe
Sample Size
10
20
30
50
90
Legend
Average Value
Spline

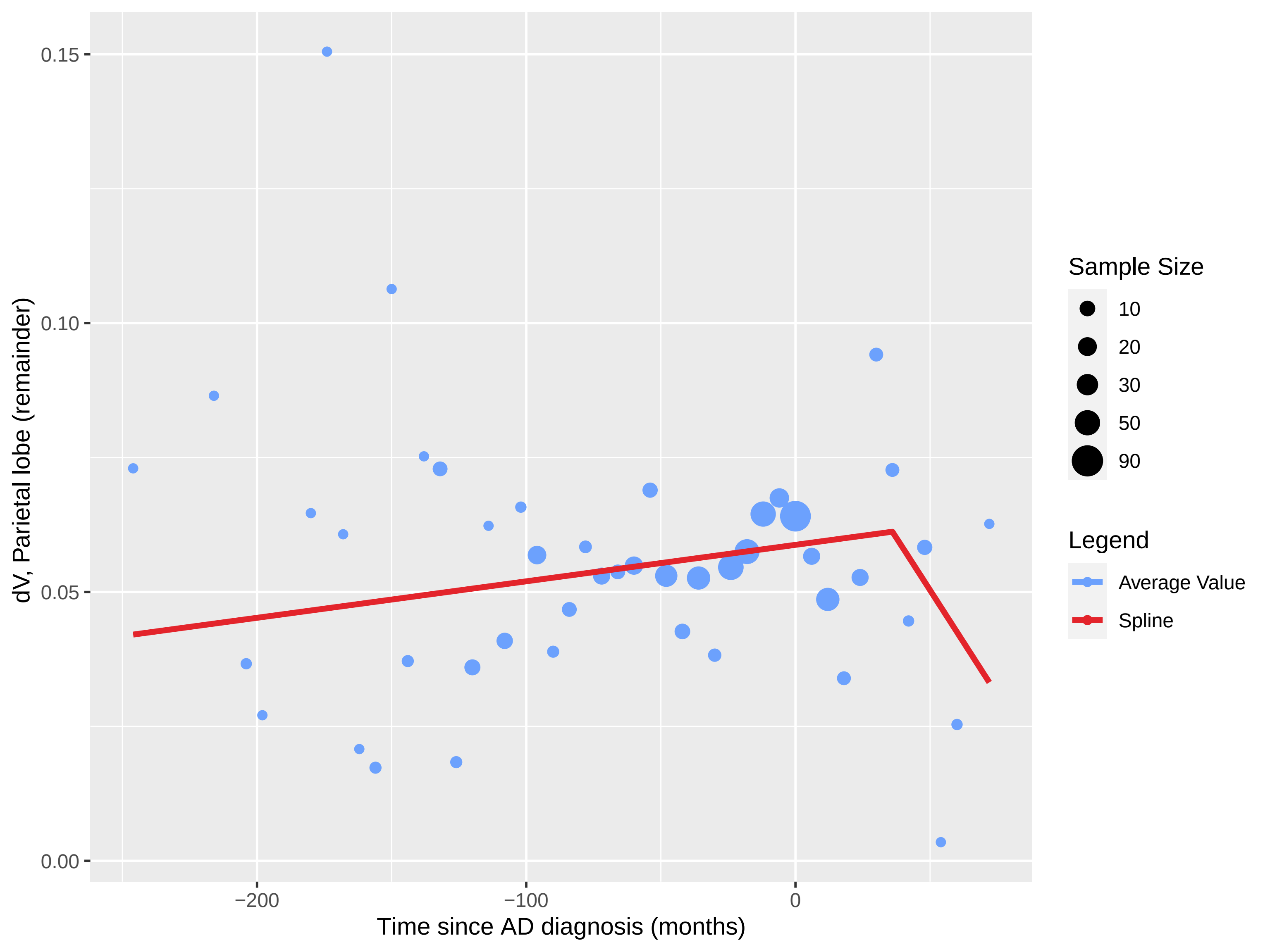

0.15
0.10
0.05
0.00
dV, Parietal lobe (remainder)
−200
−100
0
Time since AD diagnosis (months)
Sample Size
10
20
30
50
90
Legend
Average Value
Spline

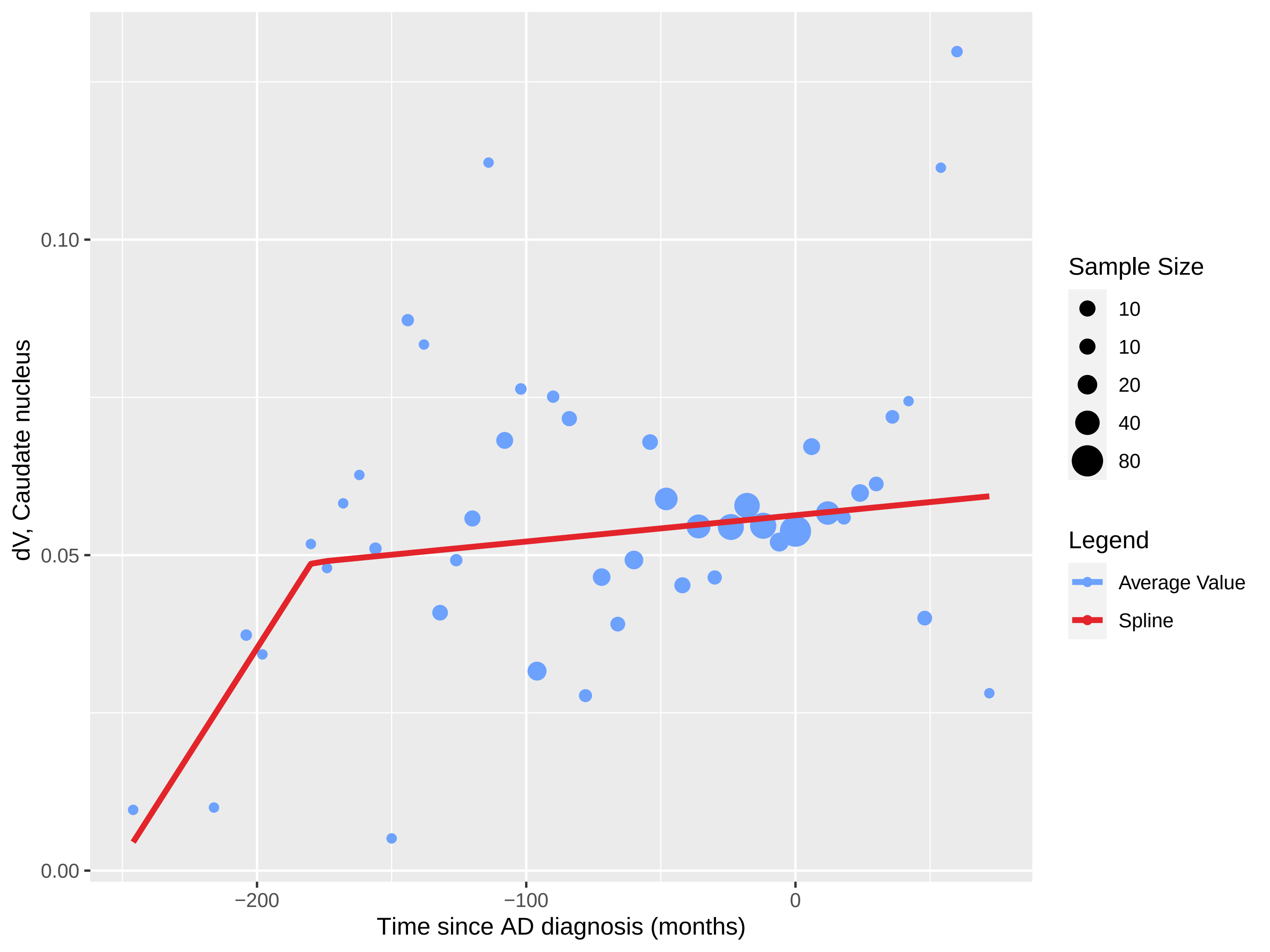

dV, Caudate nucleus
0.10
0.05
0.00
−200
−100
0
Time since AD diagnosis (months)
Sample Size
10
10
20
40
80
Legend
Average Value
Spline

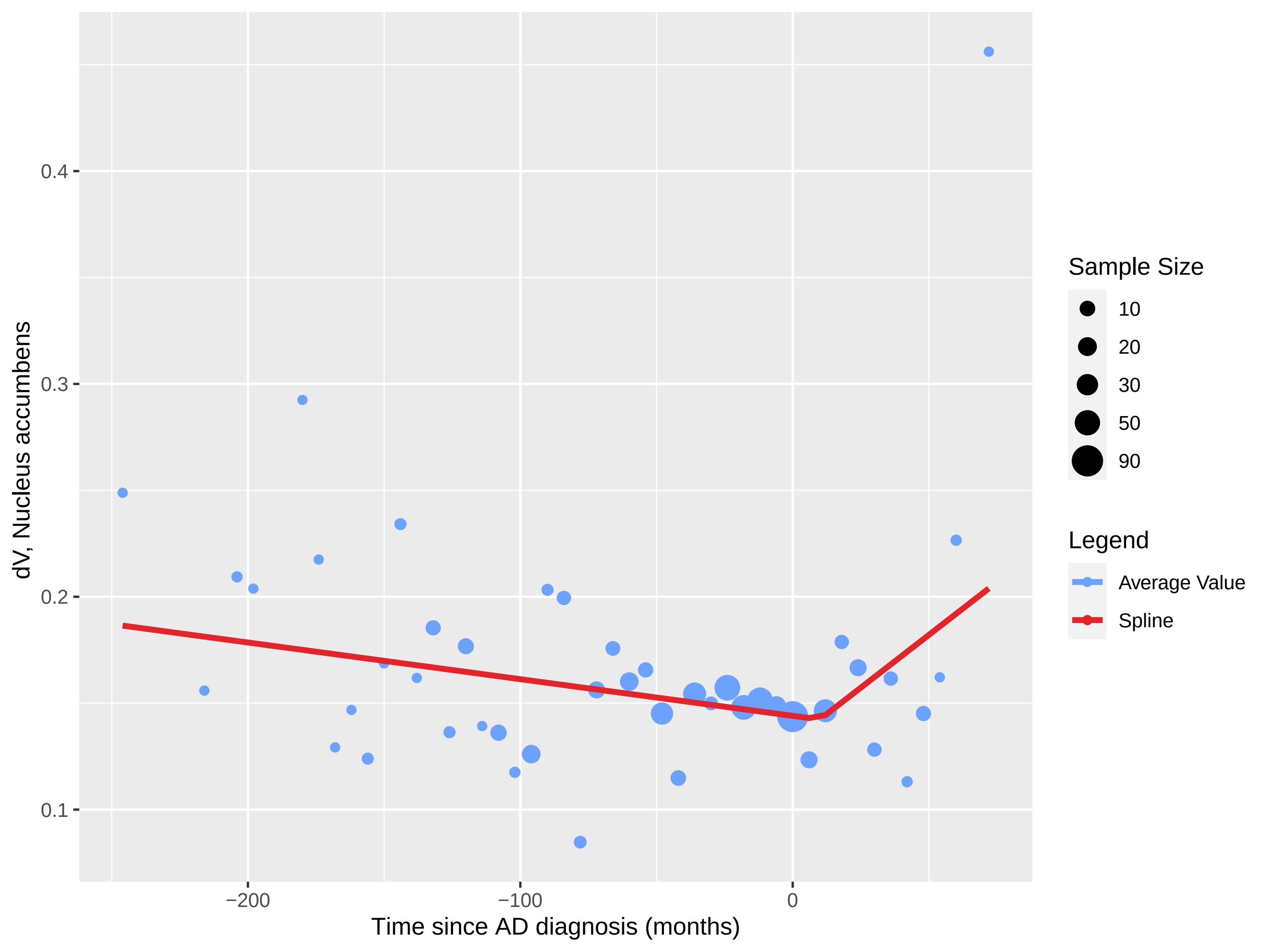
dV, Nucleus accumbens
0.4
0.3
0.2
0.1
−200
−100
0
Time since AD diagnosis (months)
Sample Size
10
20
30
50
90
Legend
Average Value
Spline

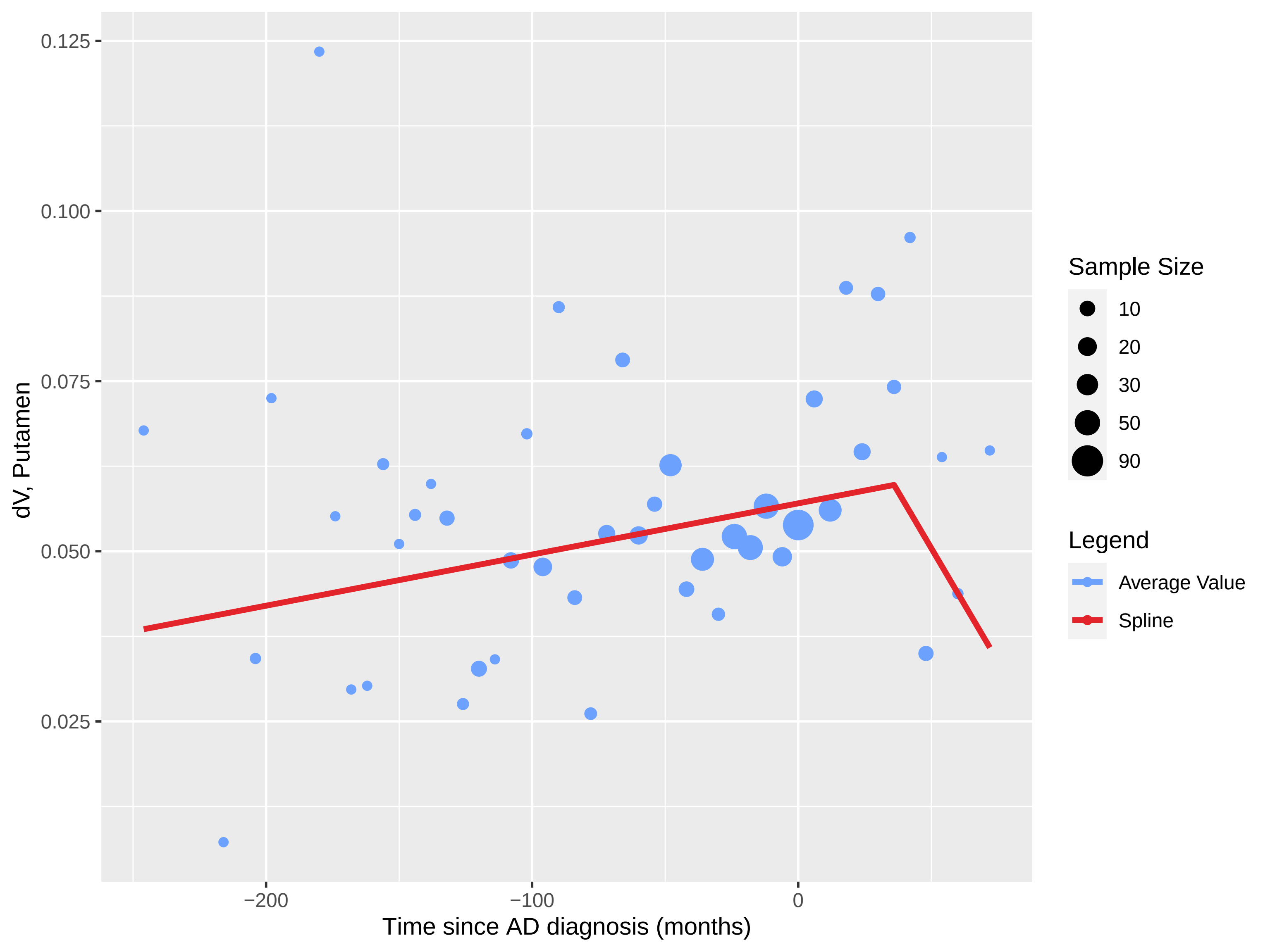
0.125
0.100
0.075
0.050
0.025
dV, Putamen
−200
−100
0
Time since AD diagnosis (months)
Sample Size
10
20
30
50
90
Legend
Average Value
Spline

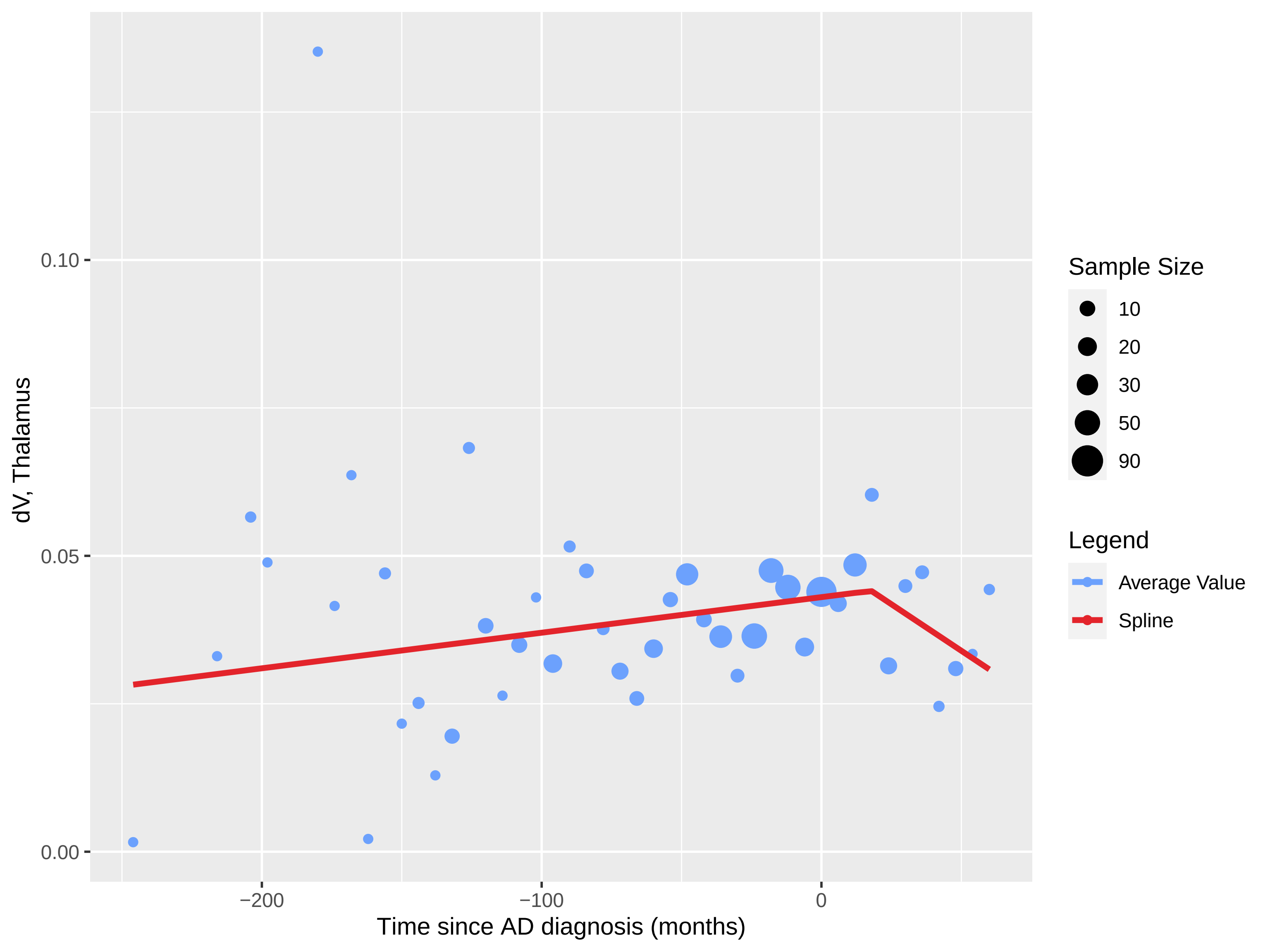

dV, Thalamus
0.10
0.05
0.00
−200
−100
0
Time since AD diagnosis (months)
Sample Size
10
20
30
50
90
Legend
Average Value
Spline

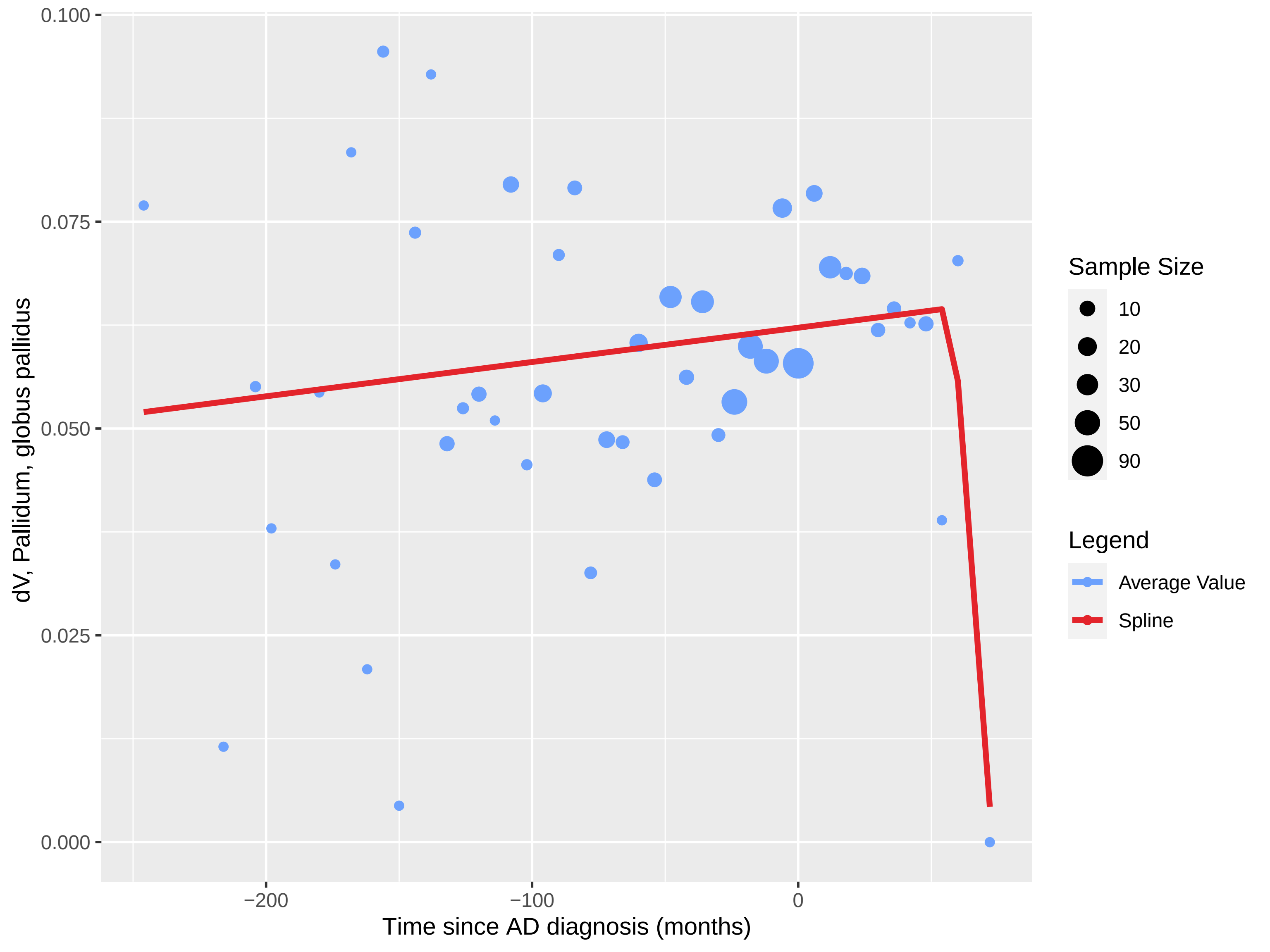

0.100
0.075
0.050
0.025
0.000
dV, Pallidum, globus pallidus
−200
−100
0
Time since AD diagnosis (months)
Sample Size
10
20
30
50
90
Legend
Average Value
Spline

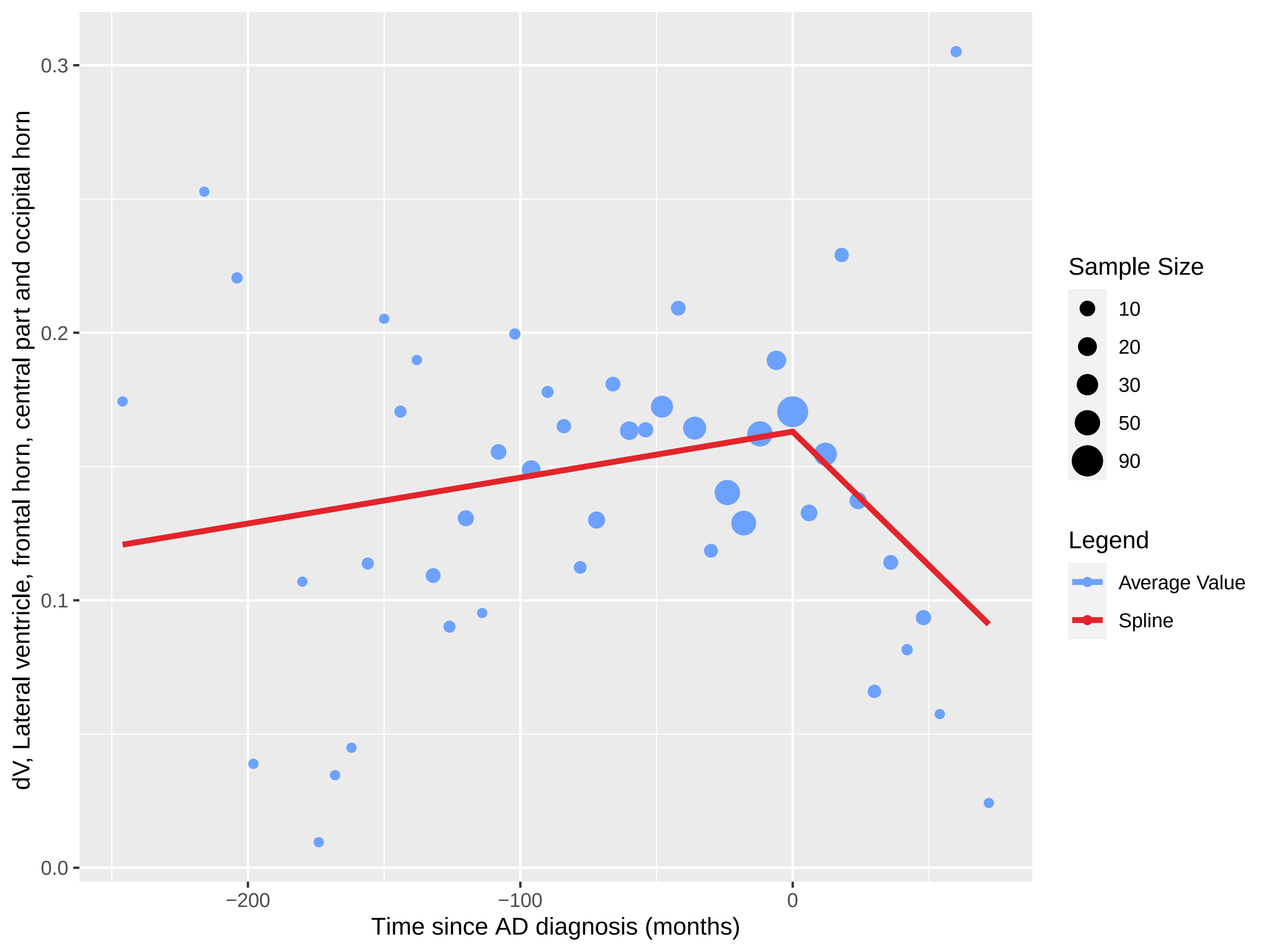

dV, Lateral ventricle, frontal horn, central part and occipital horn
0.3
0.2
0.1
0.0
−200
−100
0
Time since AD diagnosis (months)
Sample Size
10
20
30
50
90
Legend
Average Value
Spline

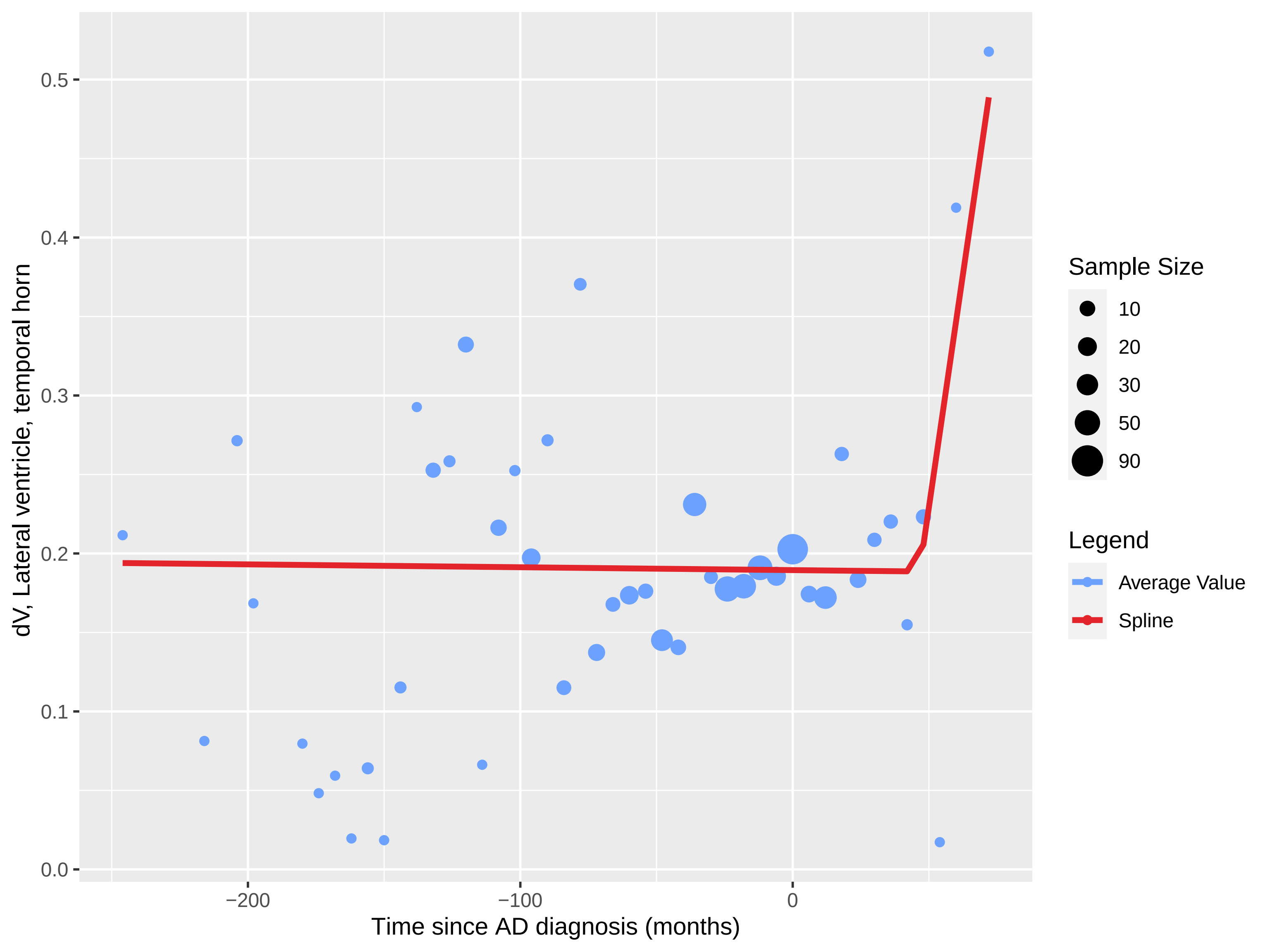

dV, Lateral ventricle, temporal horn
0.5
0.4
0.3
0.2
0.1
0.0
−200
−100
0
Time since AD diagnosis (months)
Sample Size
10
20
30
50
90
Legend
Average Value
Spline

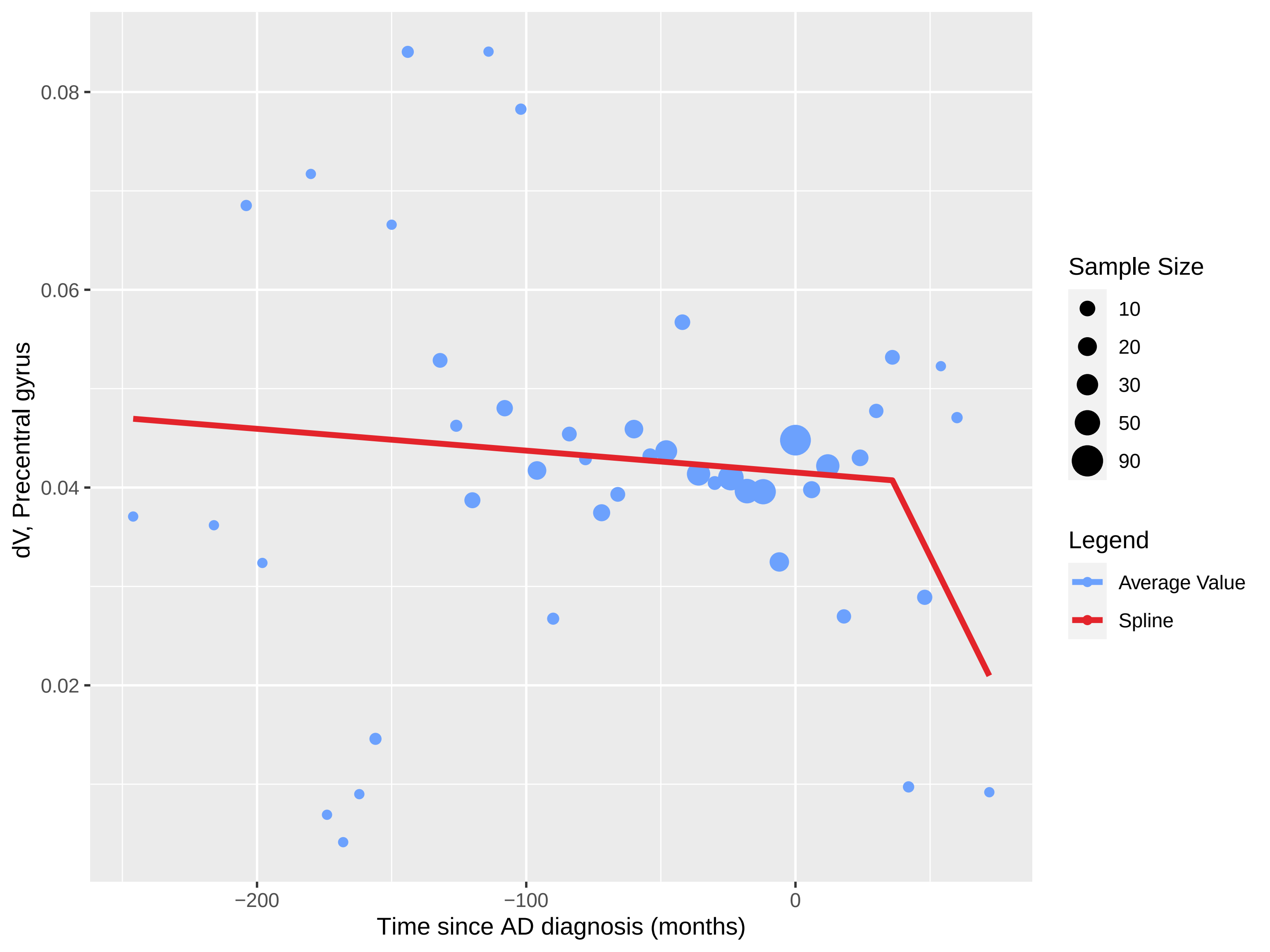

0.08
0.06
0.04
0.02
−200
−100
0
dV, Precentral gyrus
Time since AD diagnosis (months)
Sample Size
10
20
30
50
90
Legend
Average Value
Spline

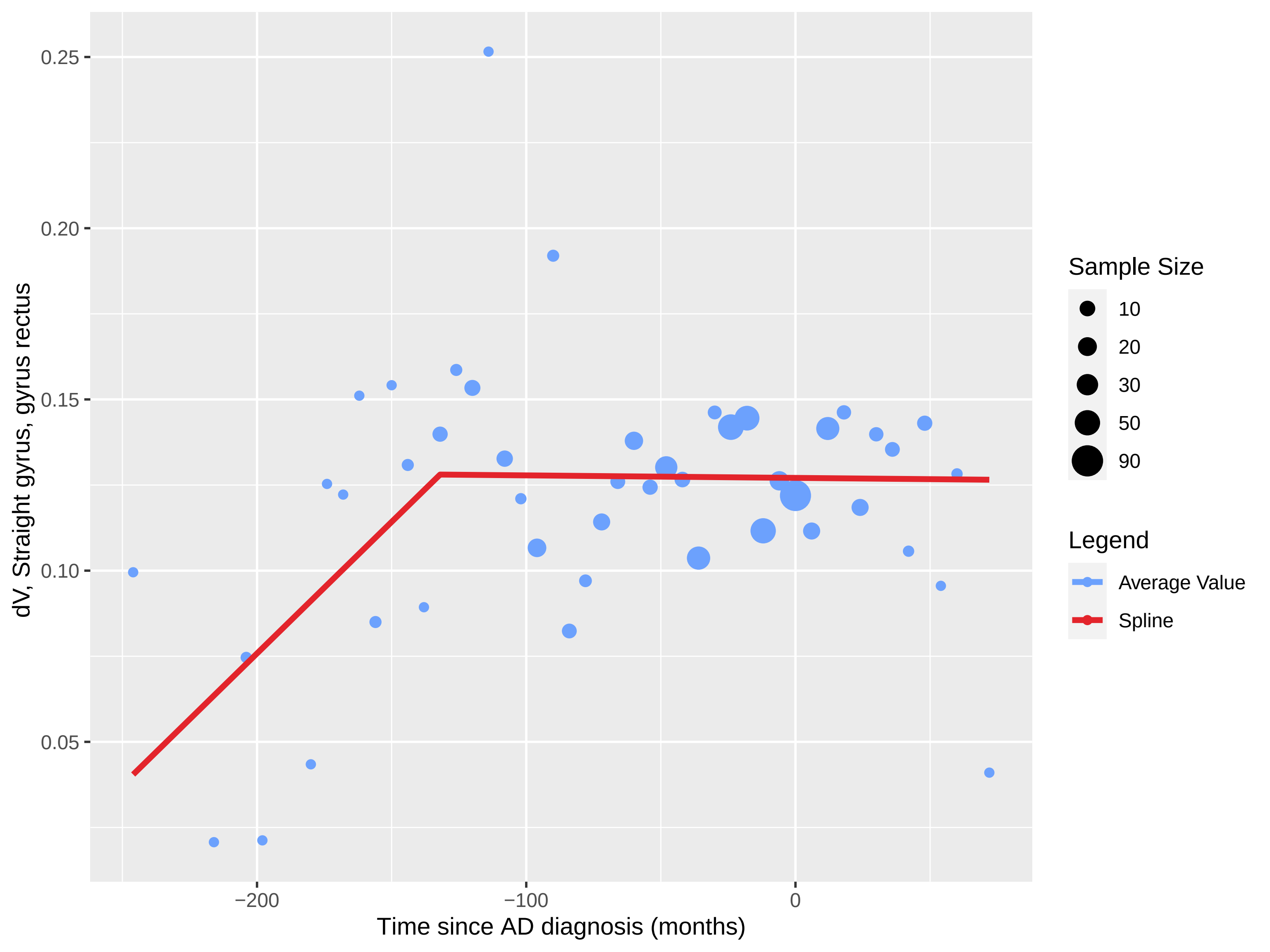

0.25
0.20
0.15
0.10
0.05
dV, Straight gyrus, gyrus rectus
−200
−100
0
Time since AD diagnosis (months)
Sample Size
10
20
30
50
90
Legend
Average Value
Spline

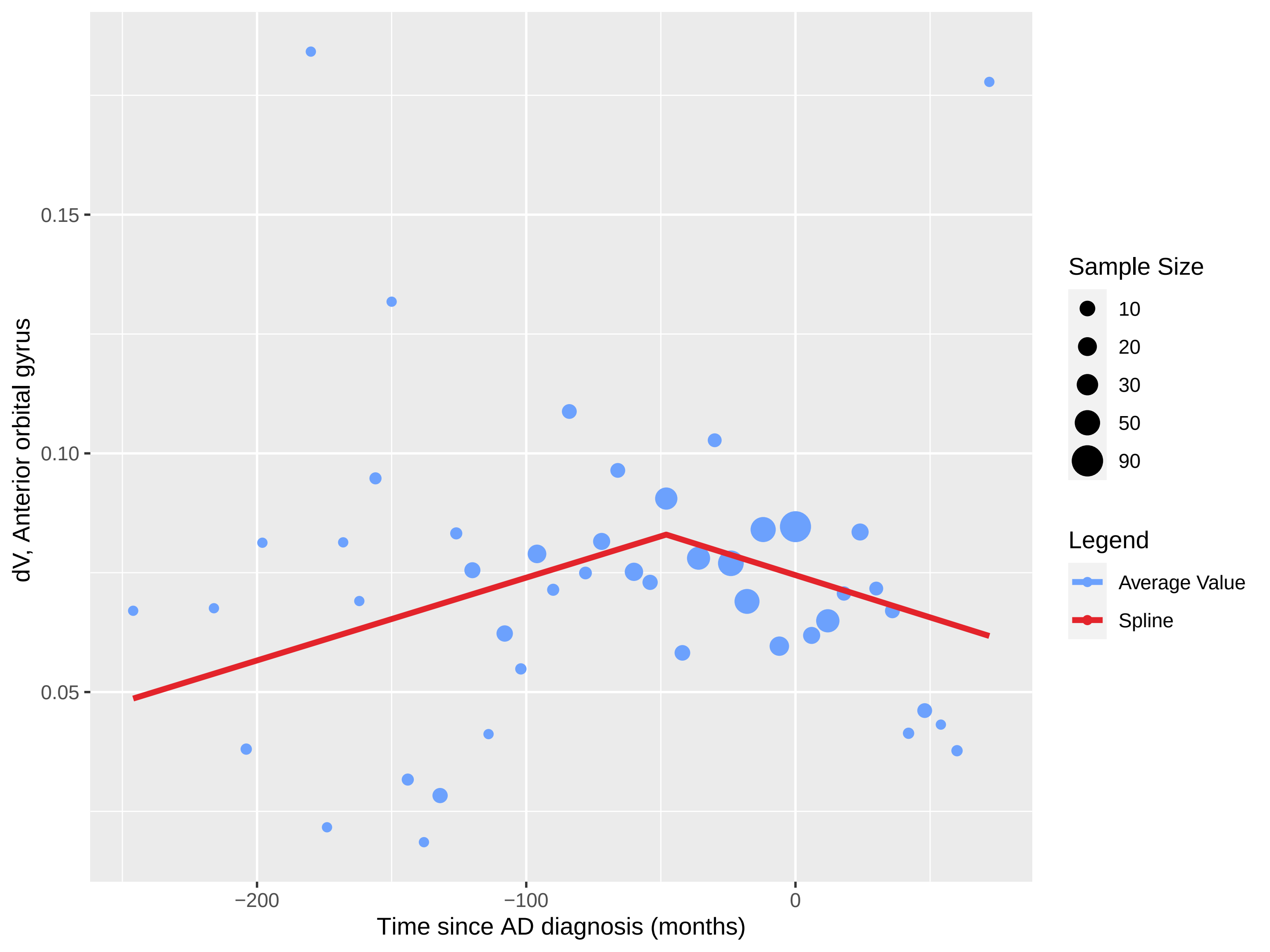

0.15
0.10
0.05
−200
−100
0
Time since AD diagnosis (months)
dV, Anterior orbital gyrus
Sample Size
10
20
30
50
90
Legend
Average Value
Spline

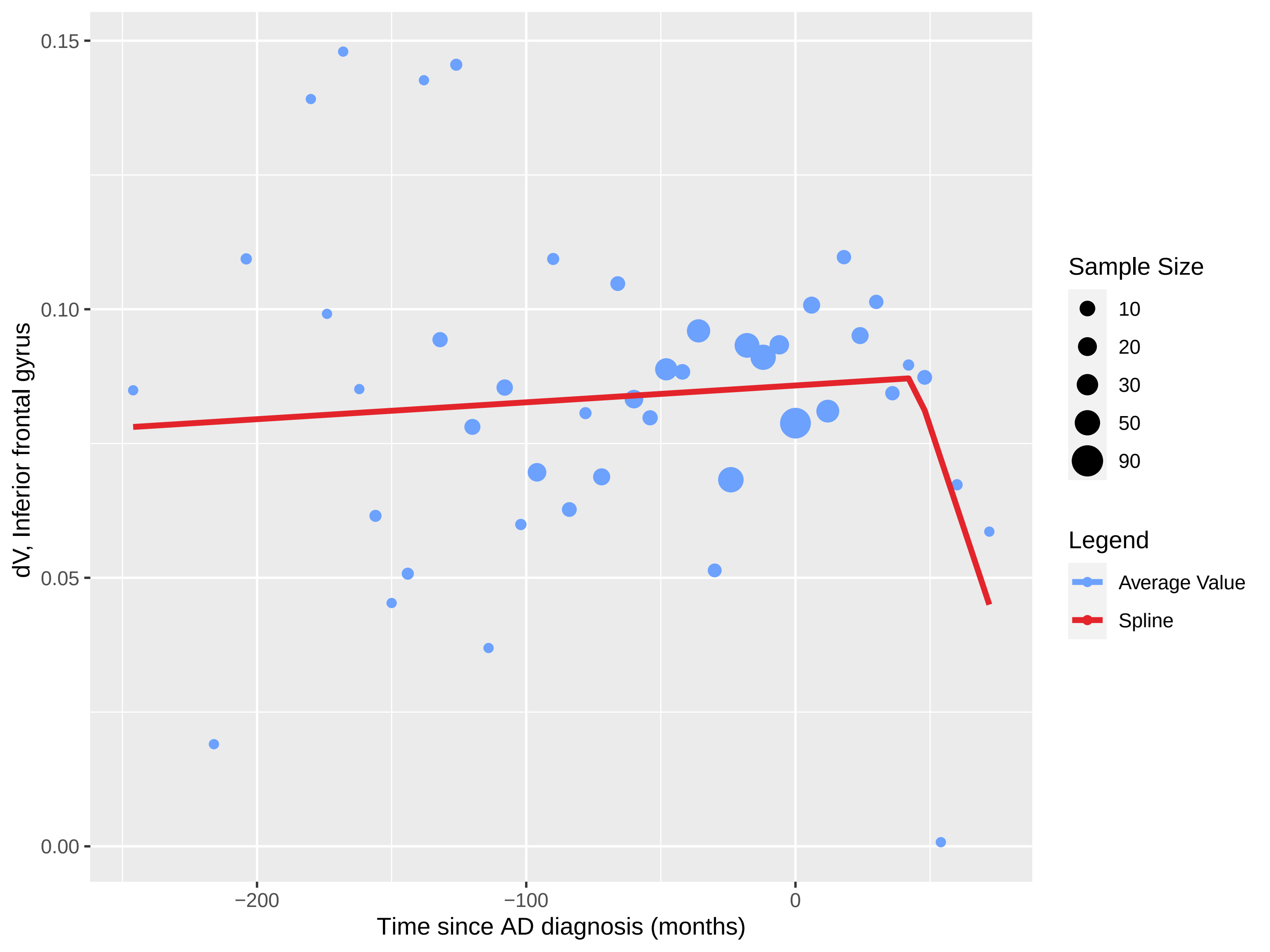
0.15
0.10
0.05
0.00
dV, Inferior frontal gyrus
−200
−100
0
Time since AD diagnosis (months)
Sample Size
10
20
30
50
90
Legend
Average Value
Spline

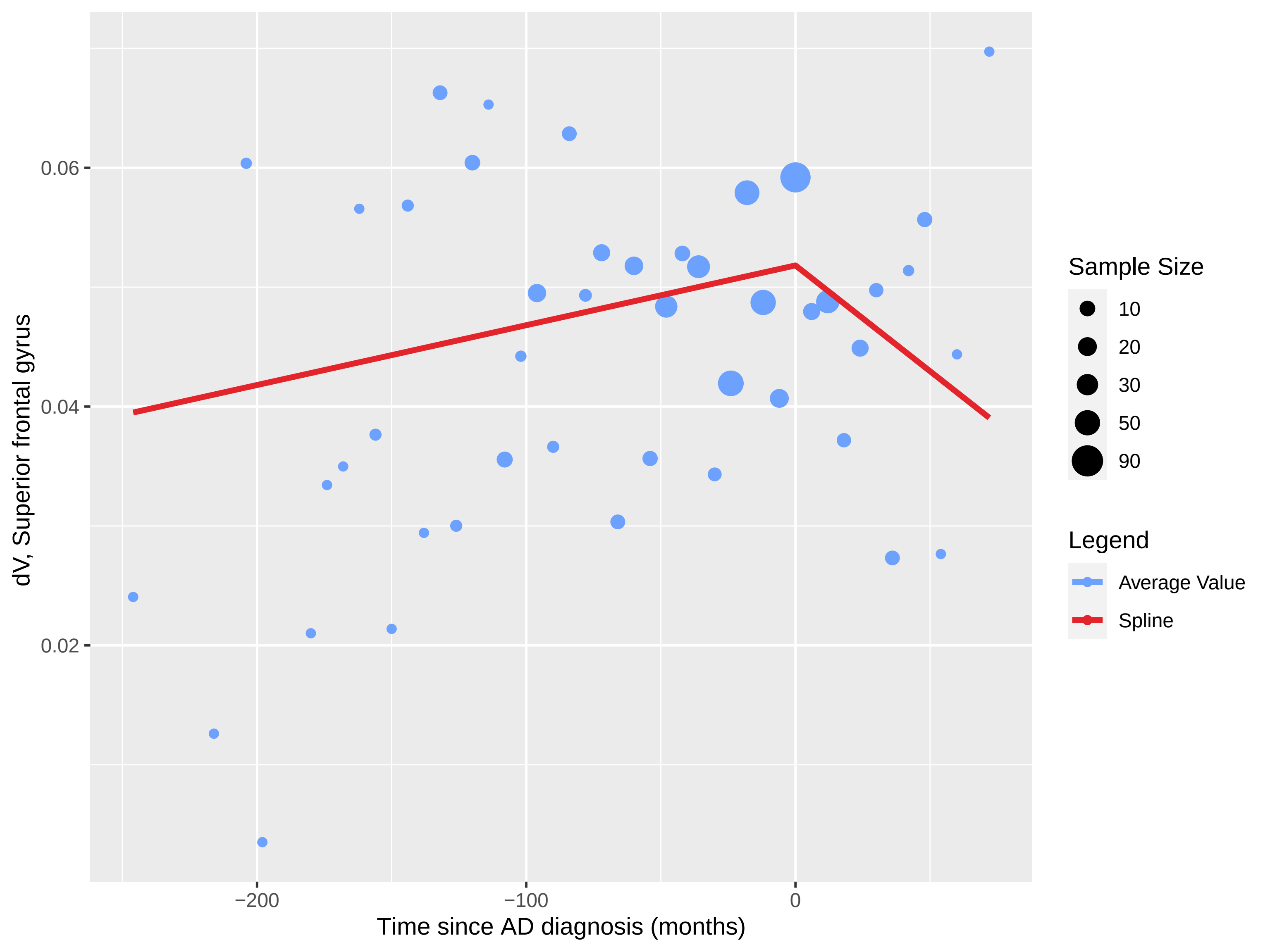

0.06
0.04
0.02
−200
−100
0
Time since AD diagnosis (months)
dV, Superior frontal gyrus
Sample Size
10
20
30
50
90
Legend
Average Value
Spline

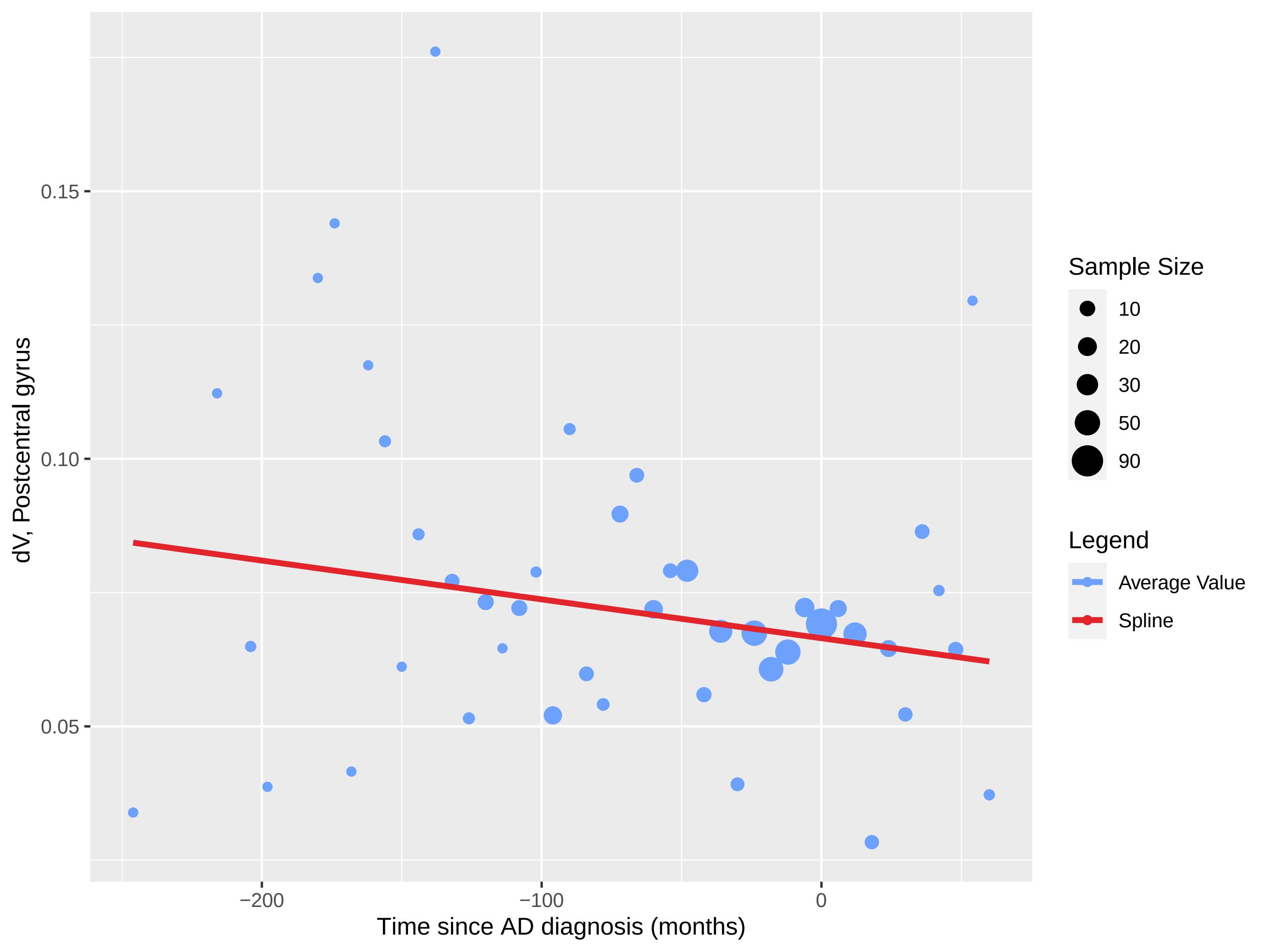

dV, Postcentral gyrus
0.15
0.10
0.05
−200
−100
0
Time since AD diagnosis (months)
Sample Size
10
20
30
50
90
Legend
Average Value
Spline

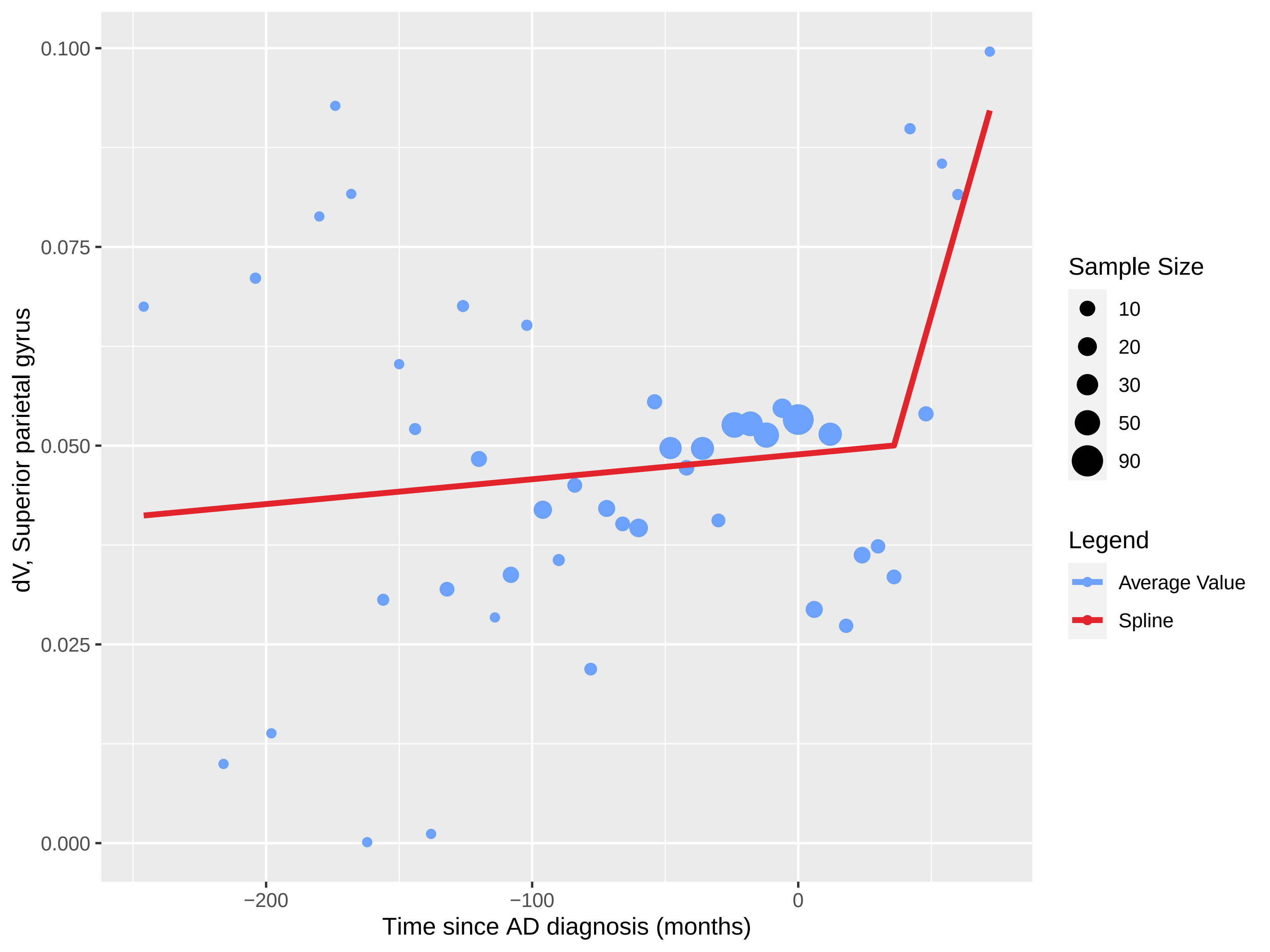

0.100
0.075
0.050
0.025
0.000
dV, Superior parietal gyrus
−200
−100
0
Time since AD diagnosis (months)
Sample Size
10
20
30
50
90
Legend
Average Value
Spline

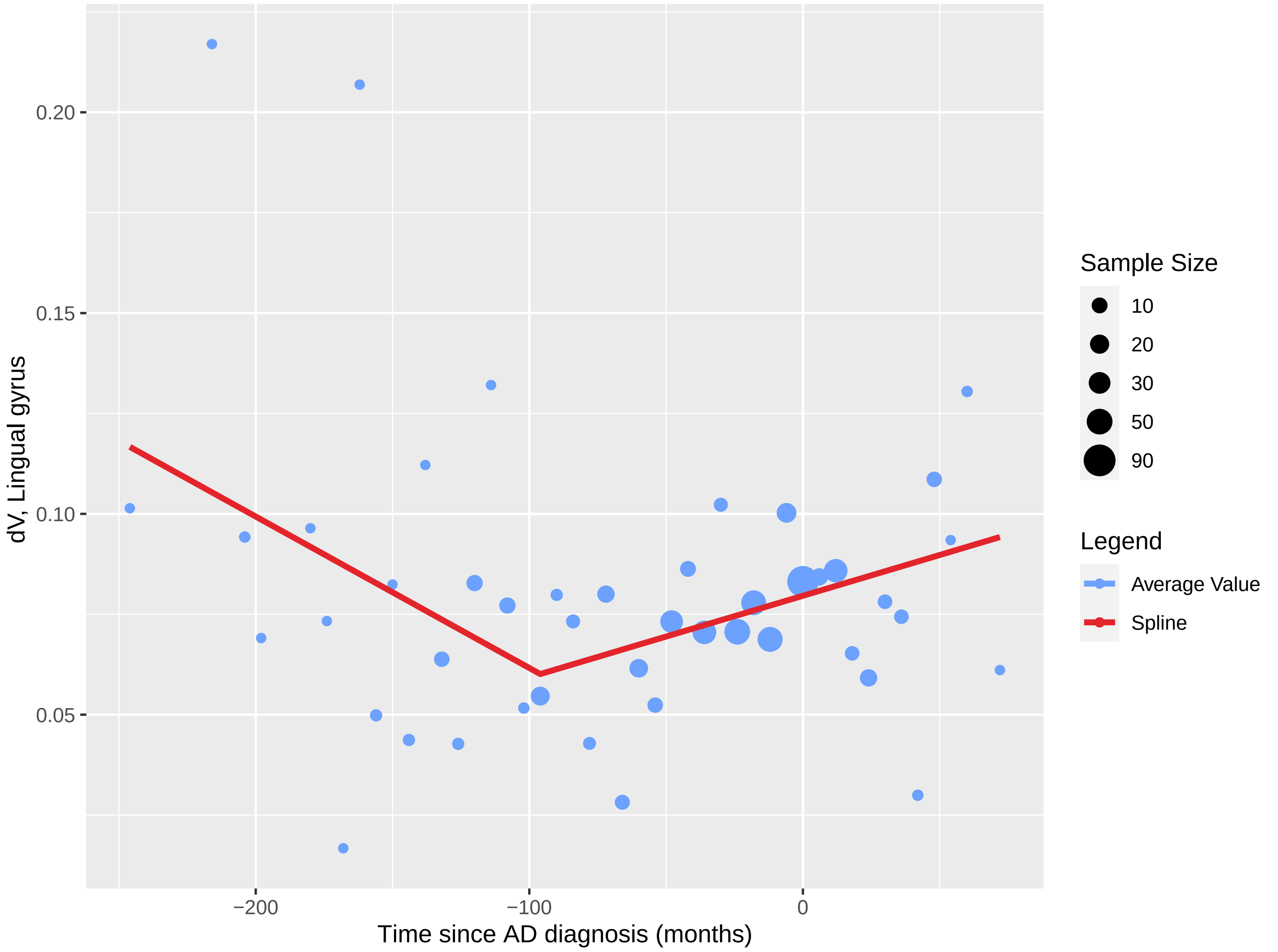

0.20
0.15
0.10
0.05
dV, Lingual gyrus
−200
−100
0
Time since AD diagnosis (months)
Sample Size
10
20
30
50
90
Legend
Average Value
Spline

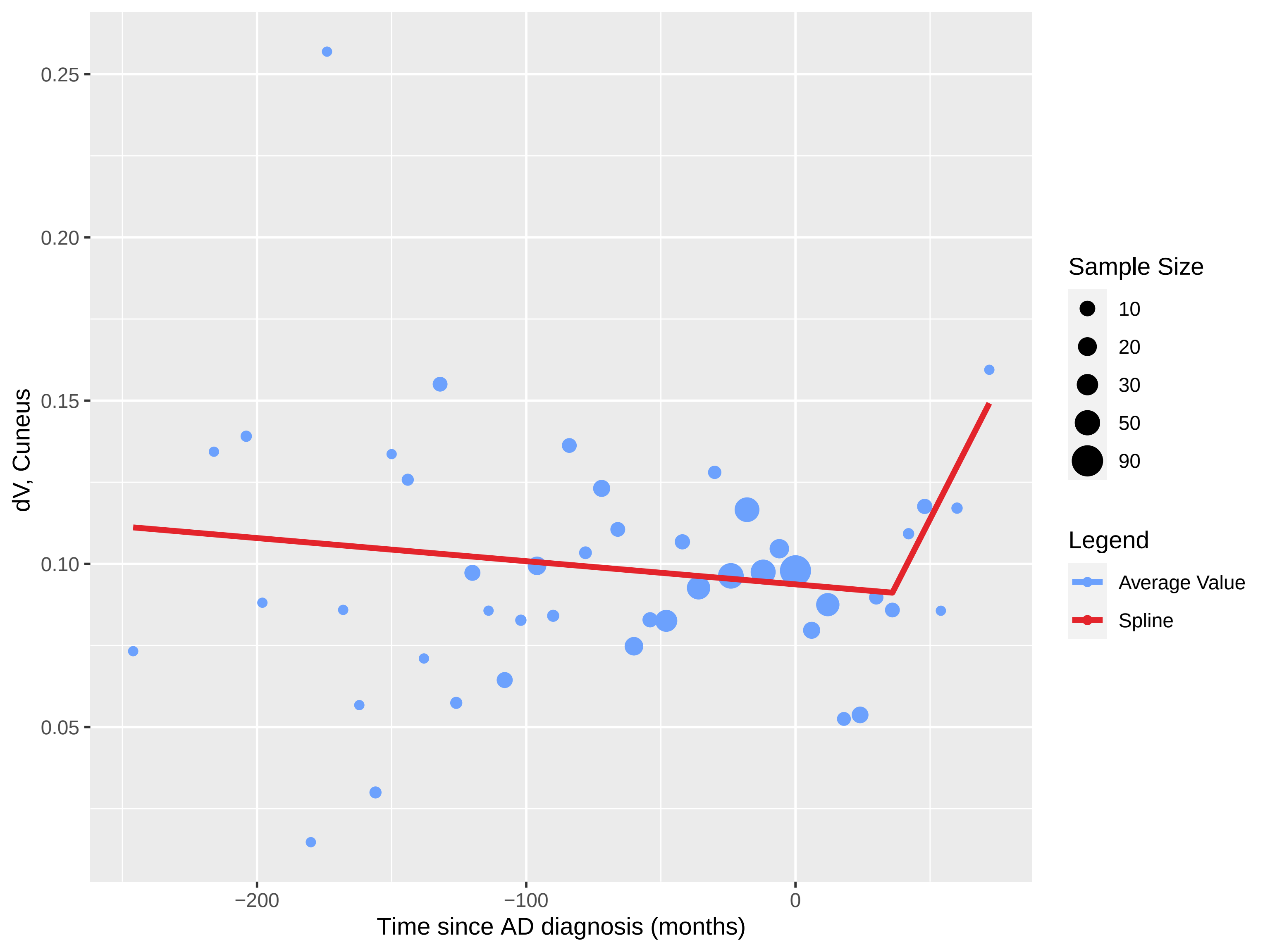

0.25
0.20
0.15
0.10
0.05
−200
−100
0
Time since AD diagnosis (months)
dV, Cuneus
Sample Size
10
20
30
50
90
Legend
Average Value
Spline

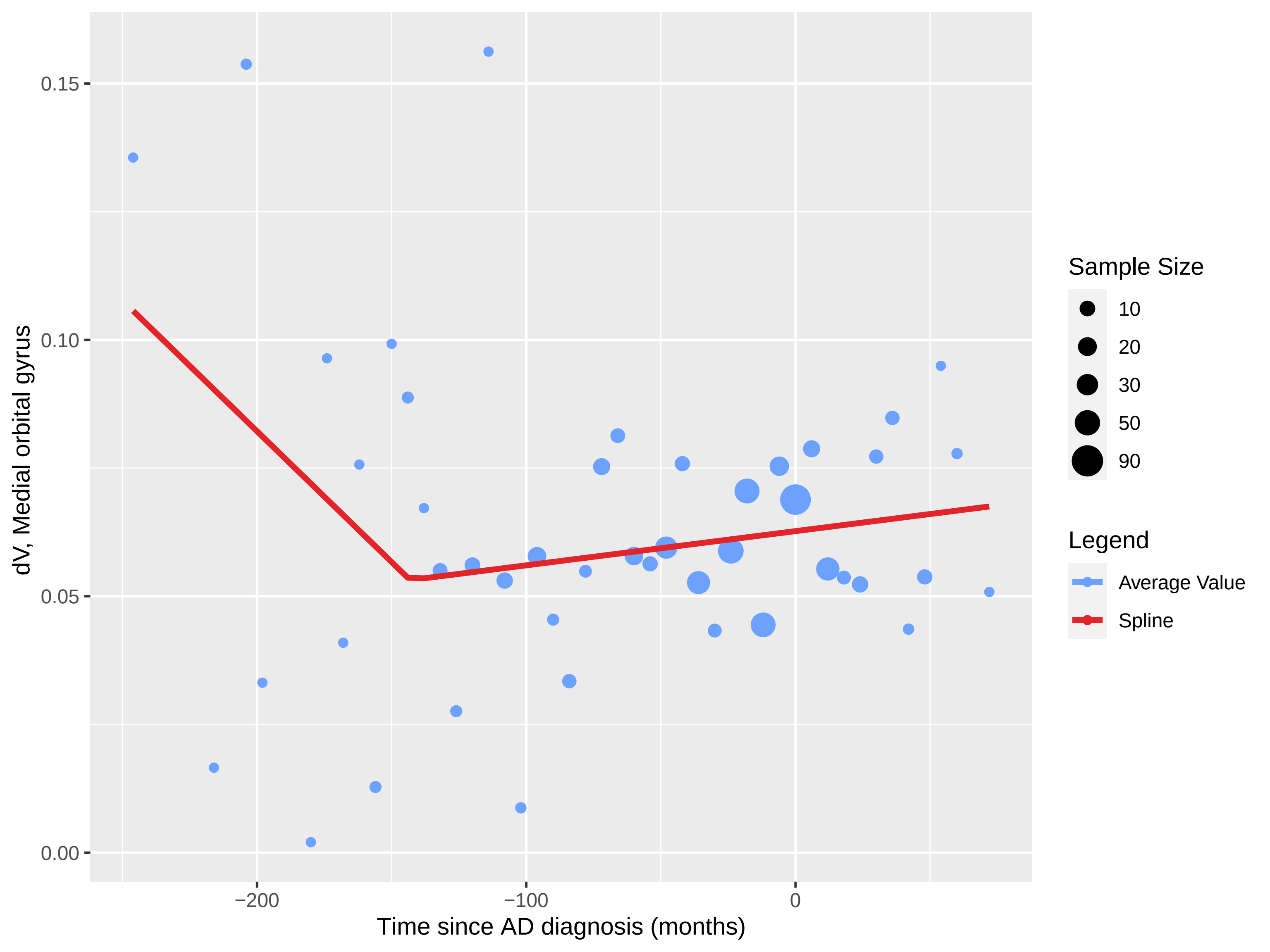

0.15
0.10
0.05
0.00
−200
−100
0
Time since AD diagnosis (months)
dV, Medial orbital gyrus
Sample Size
10
20
30
50
90
Legend
Average Value
Spline

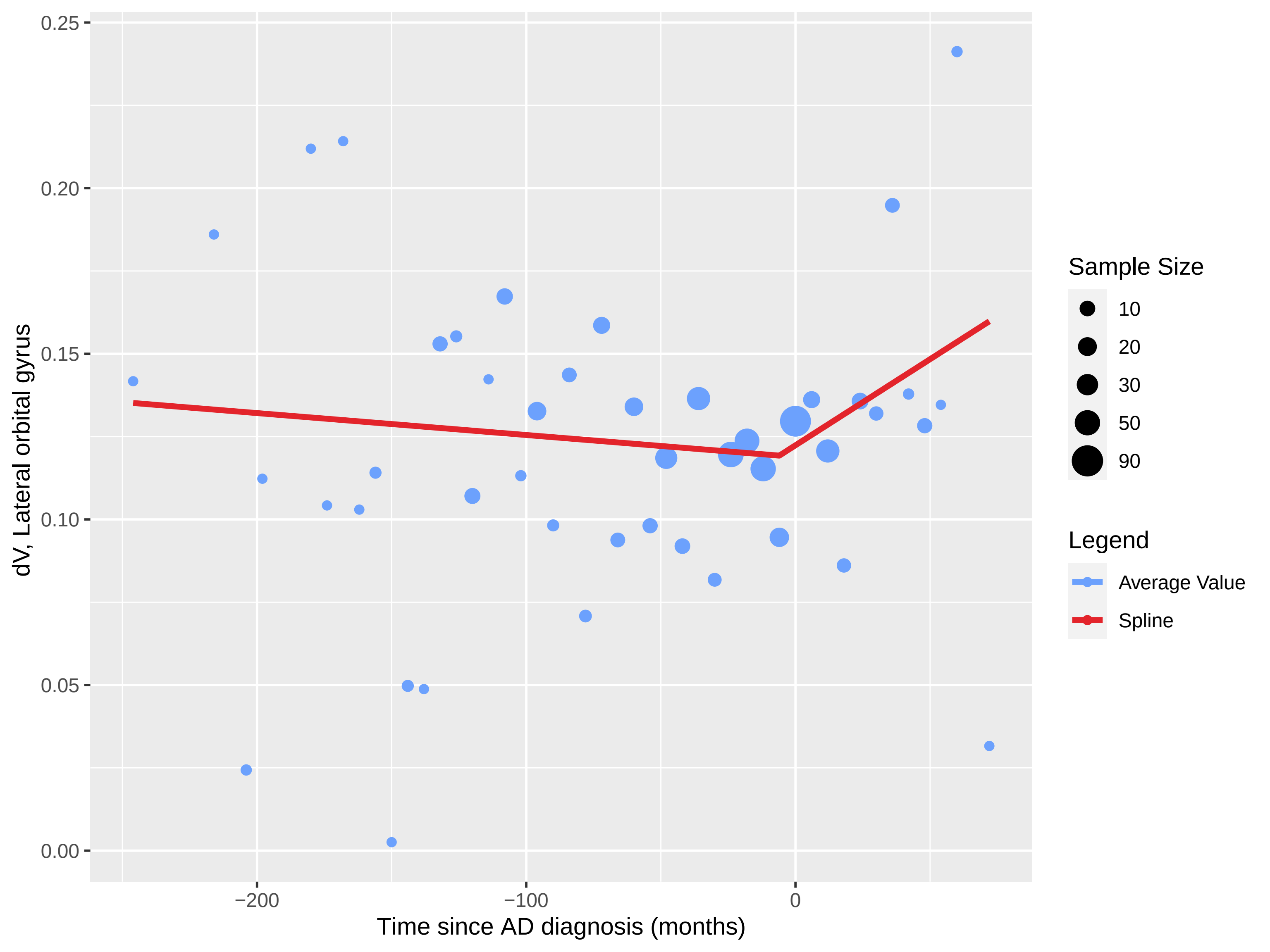

0.25
0.20
0.15
0.10
0.05
0.00
dV, Lateral orbital gyrus
−200
−100
0
Time since AD diagnosis (months)
Sample Size
10
20
30
50
90
Legend
Average Value
Spline

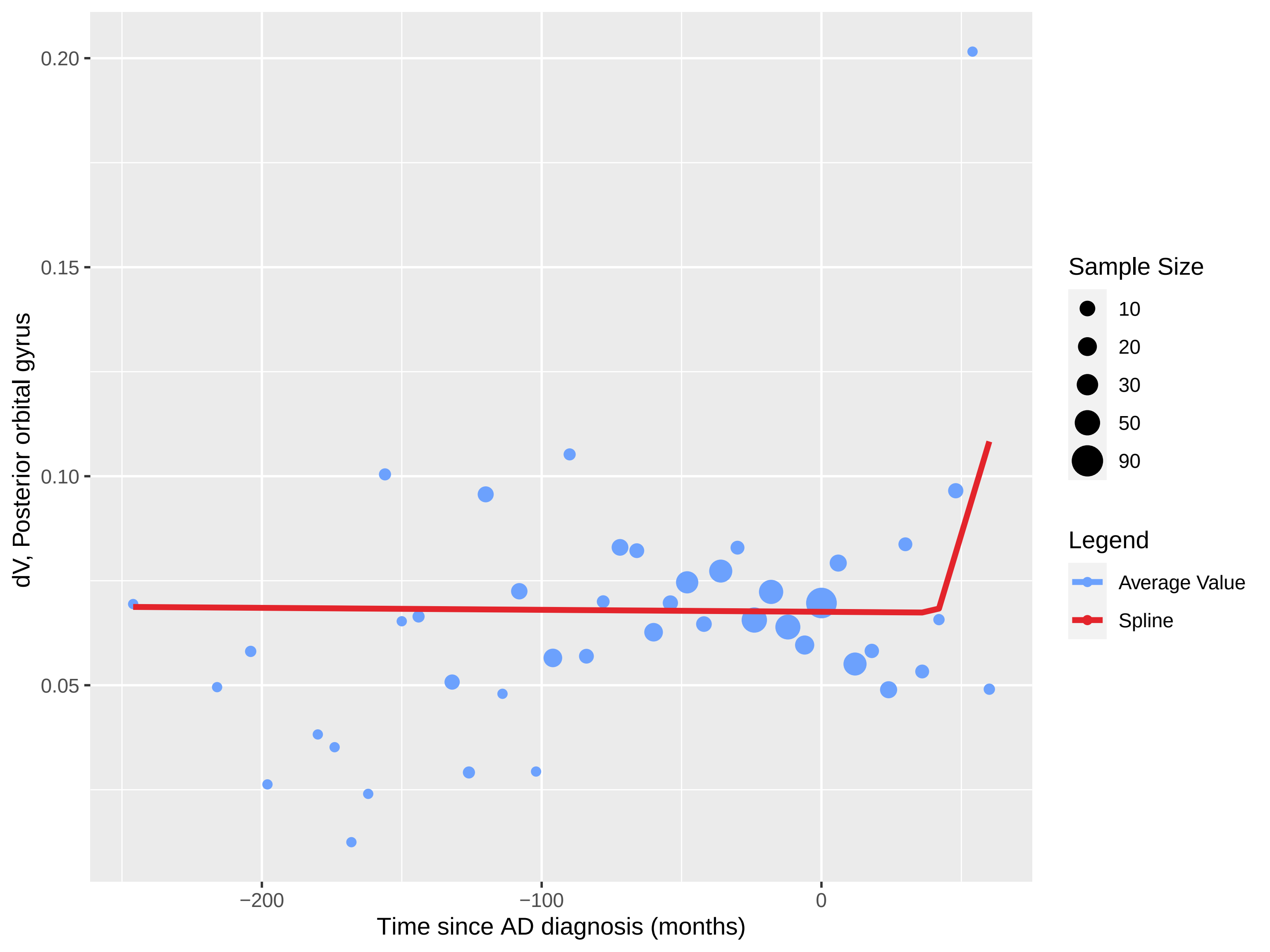

0.20
0.15
0.10
0.05
dV, Posterior orbital gyrus
−200
−100
0
Time since AD diagnosis (months)
Sample Size
10
20
30
50
90
Legend
Average Value
Spline

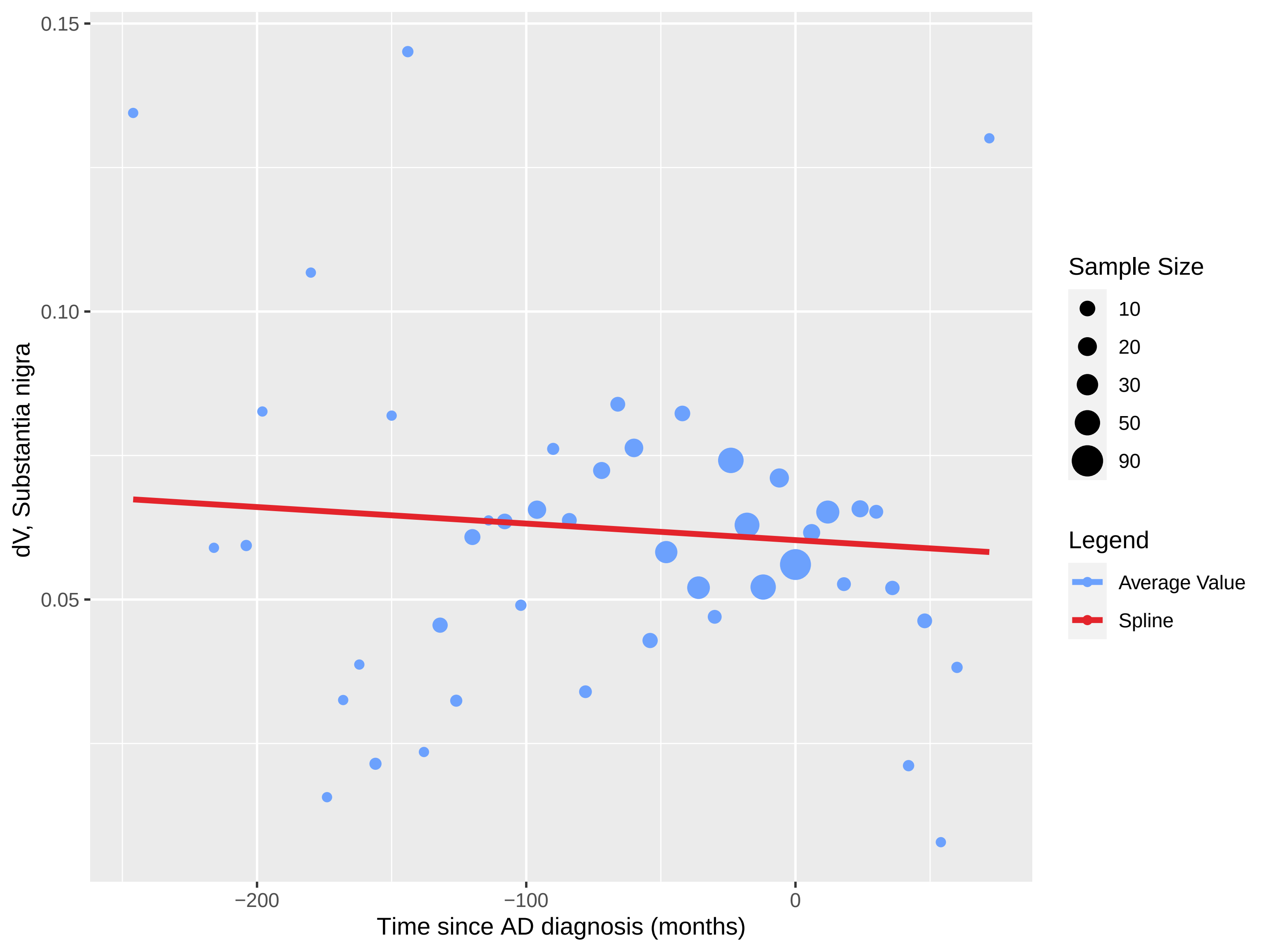

0.15
0.10
0.05
dV, Substantia nigra
−200
−100
0
Time since AD diagnosis (months)
Sample Size
10
20
30
50
90
Legend
Average Value
Spline

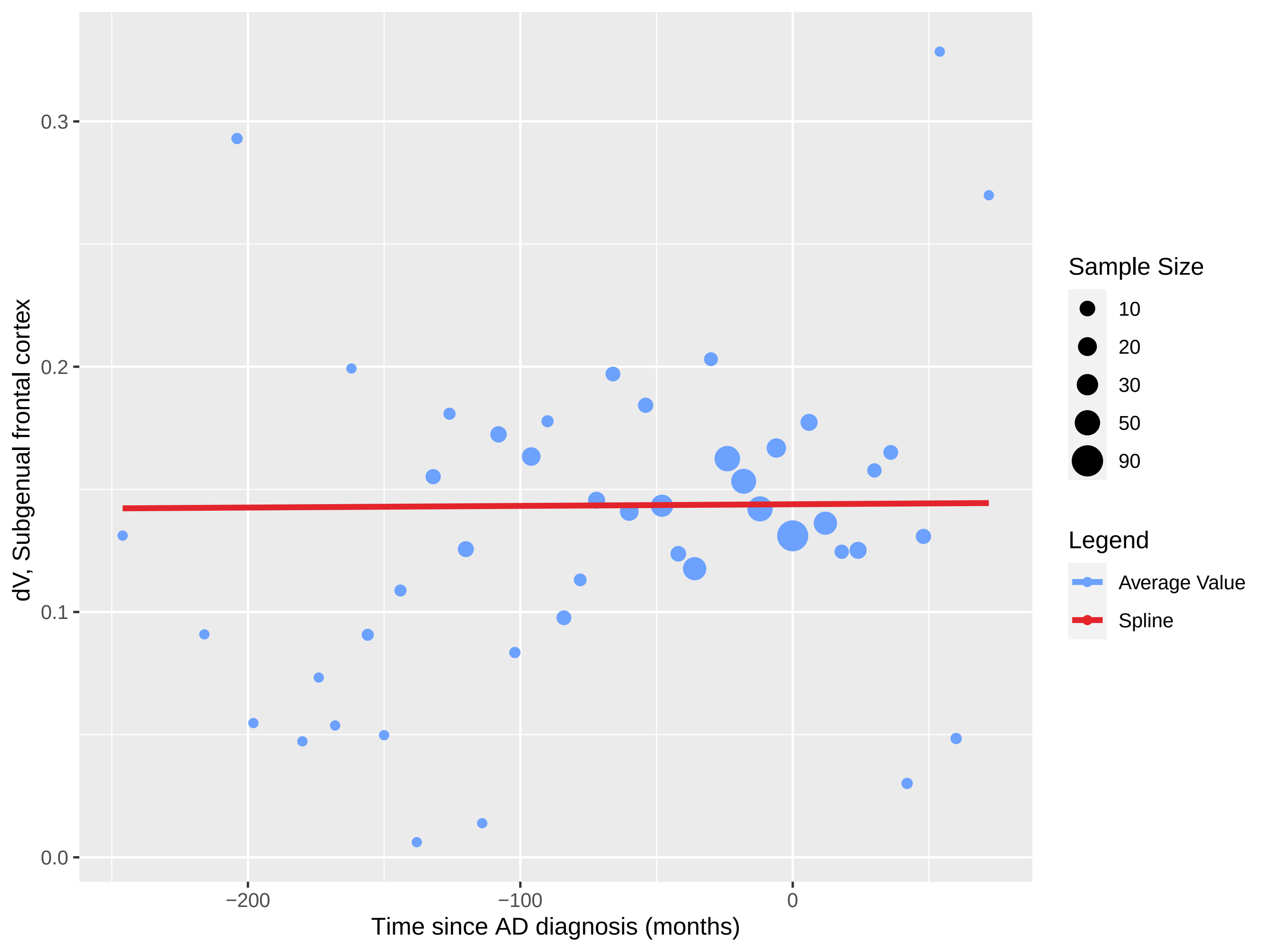

0.3
0.2
0.1
0.0
dV, Subgenual frontal cortex
−200
−100
0
Time since AD diagnosis (months)
Sample Size
10
20
30
50
90
Legend
Average Value
Spline

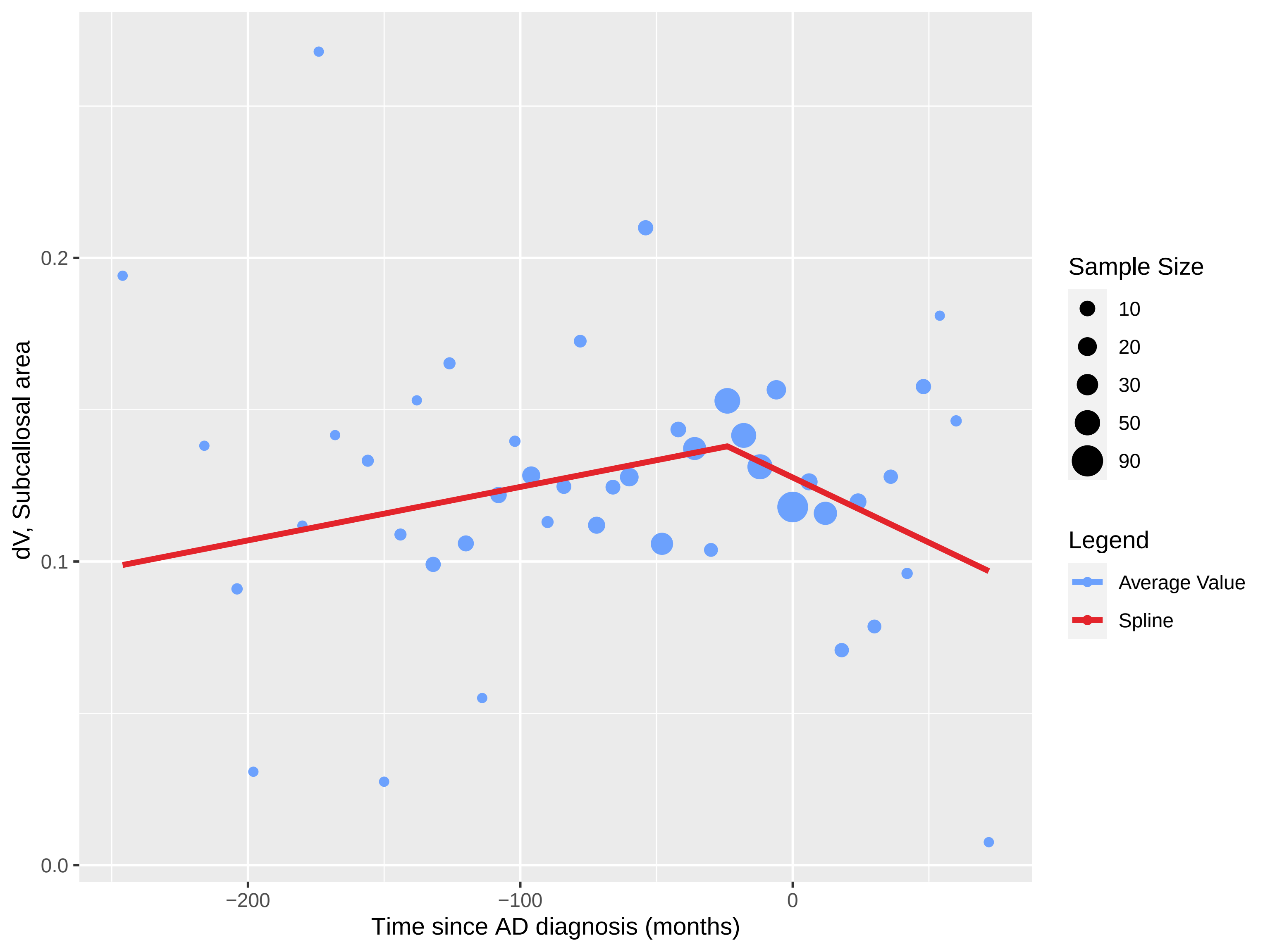

dV, Subcallosal area
0.0
0.1
0.2
−200
−100
0
Time since AD diagnosis (months)
Sample Size
10
20
30
50
90
Legend
Average Value
Spline

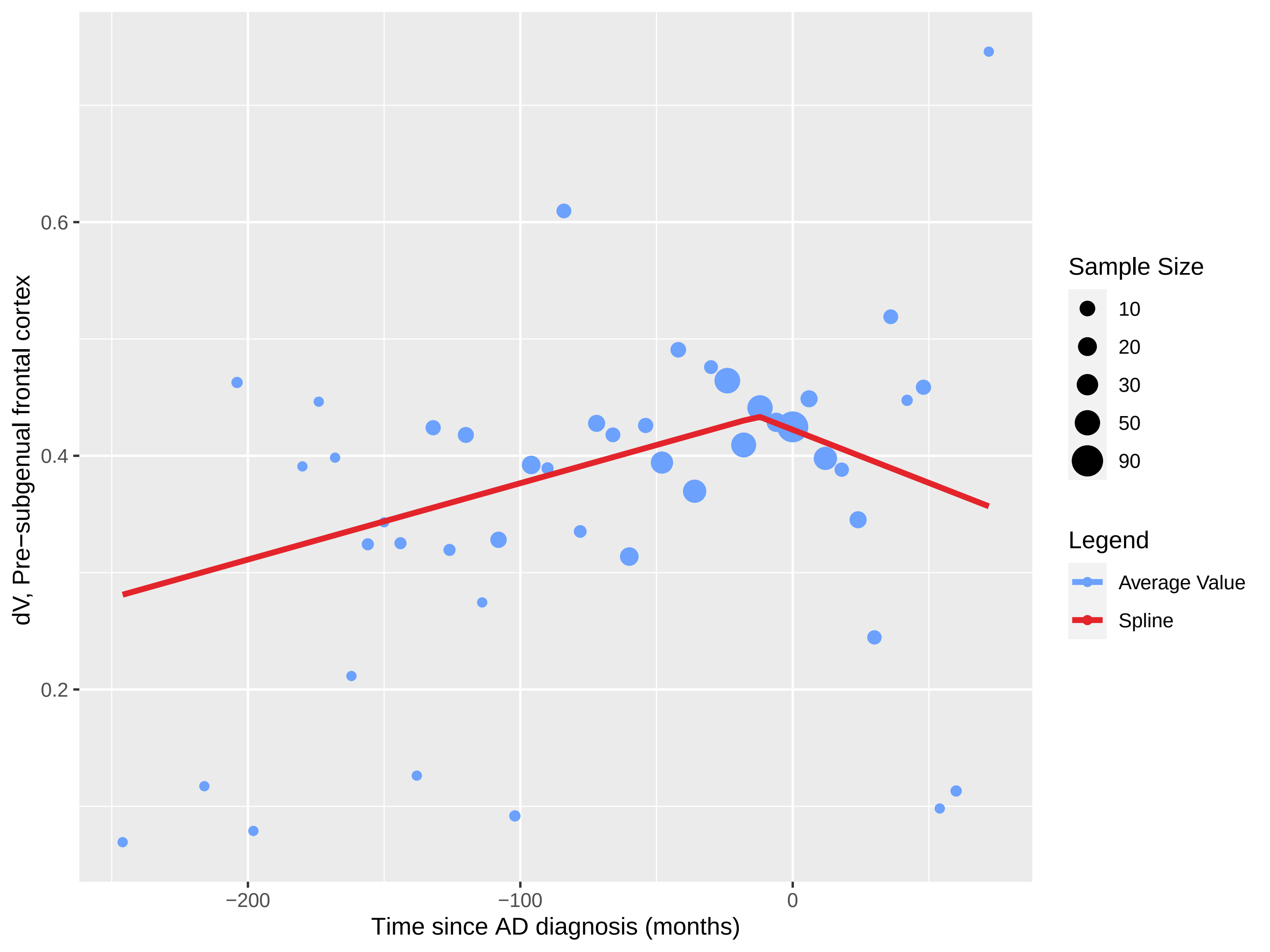

dV, Pre−subgenual frontal cortex
0.6
0.4
0.2
−200
−100
0
Time since AD diagnosis (months)
Sample Size
10
20
30
50
90
Legend
Average Value
Spline

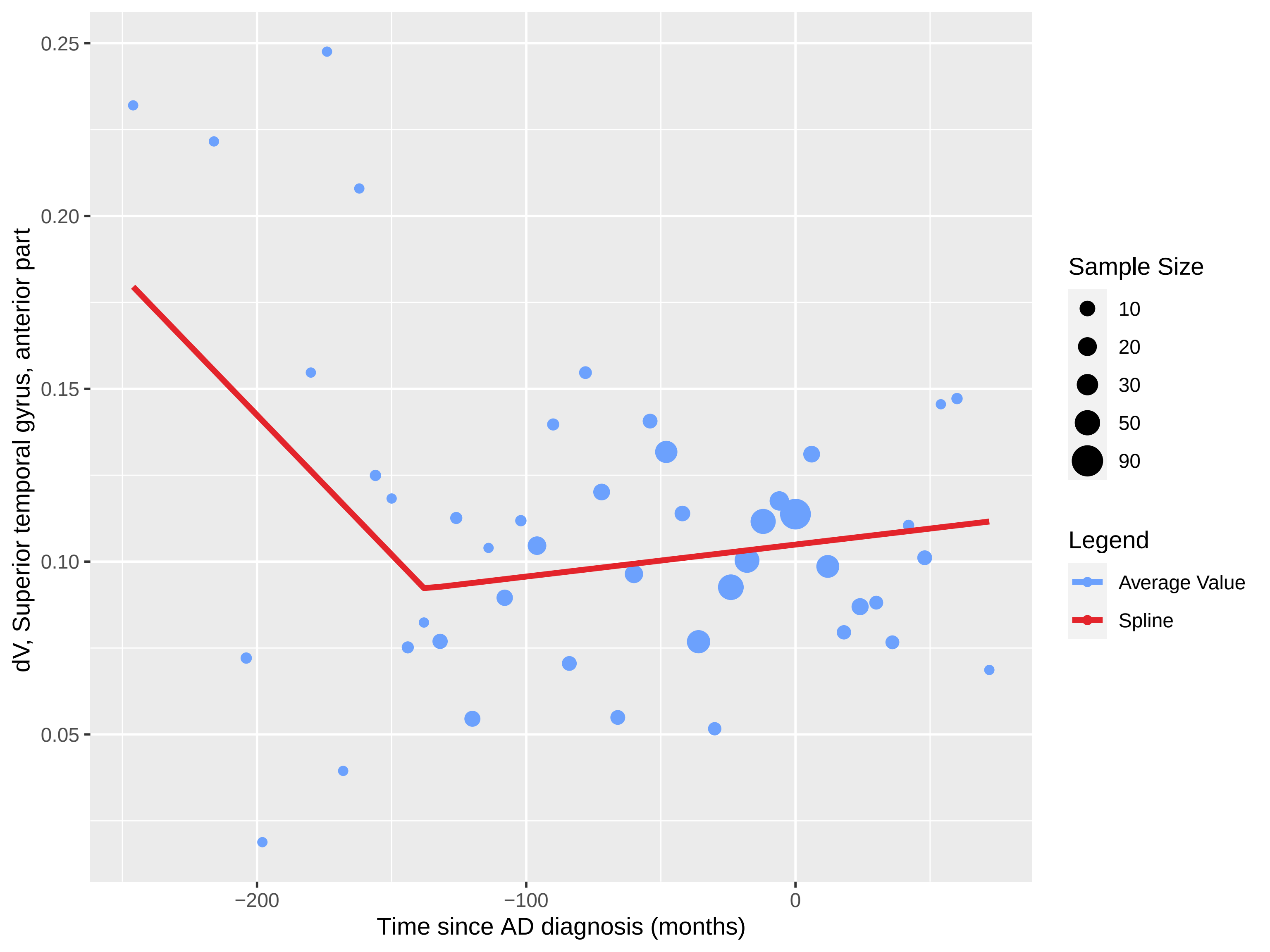

0.25
0.20
0.15
0.10
0.05
dV, Superior temporal gyrus, anterior part
−200
−100
0
Time since AD diagnosis (months)
Sample Size
10
20
30
50
90
Legend
Average Value
Spline

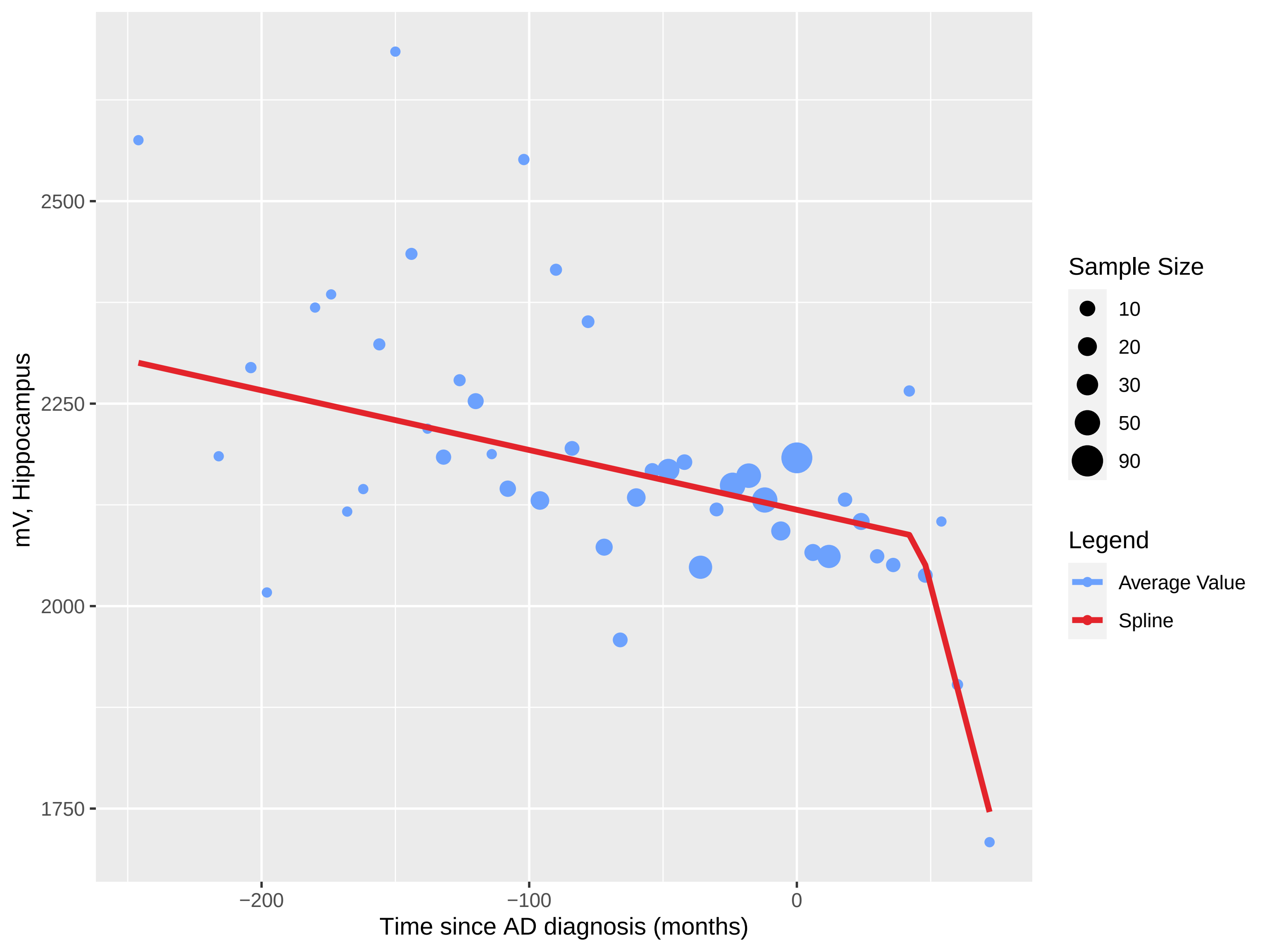

mV, Hippocampus
2500
2250
2000
1750
−200
−100
0
Time since AD diagnosis (months)
Sample Size
10
20
30
50
90
Legend
Average Value
Spline

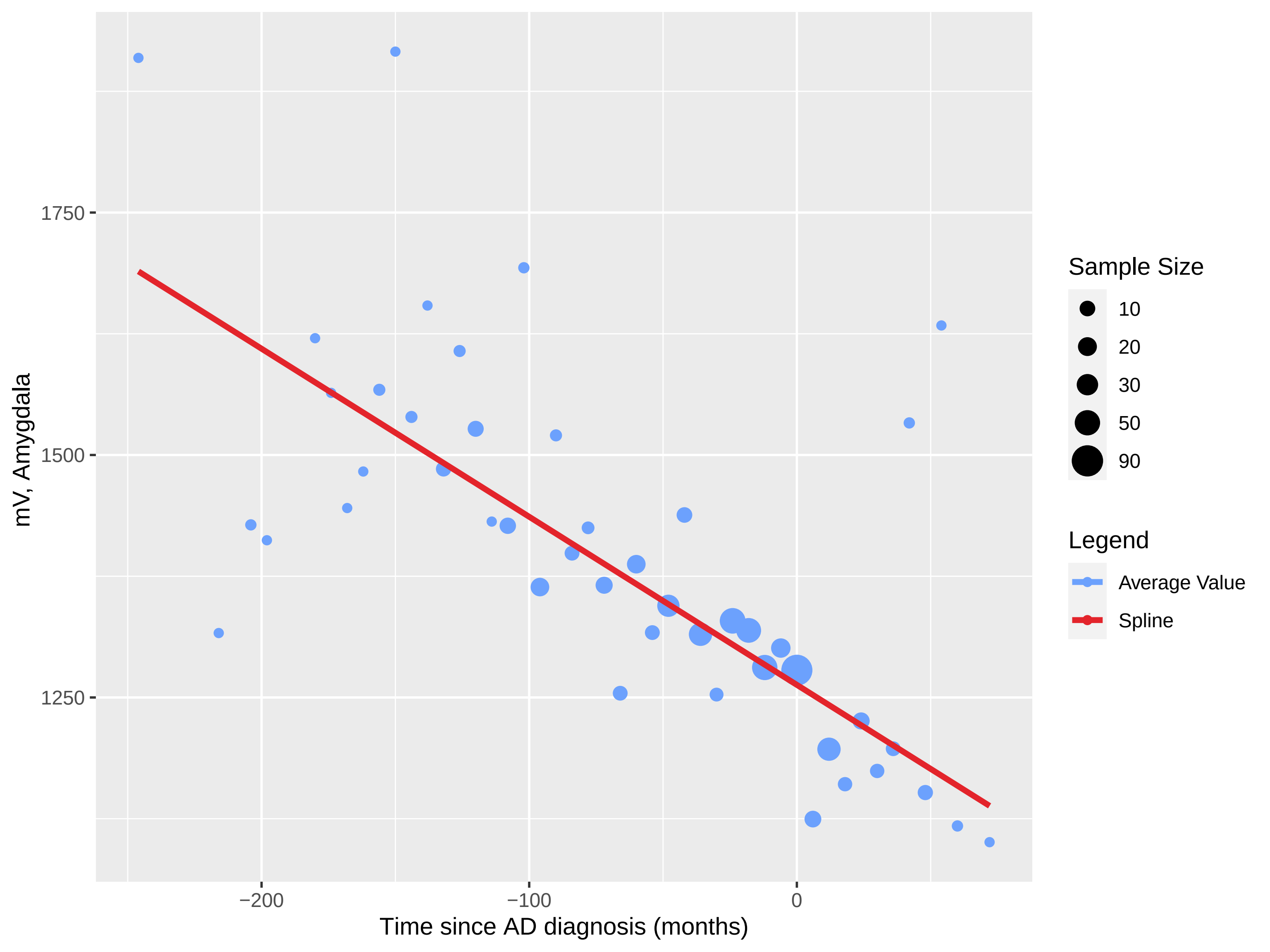

1750
1500
1250
−200
−100
0
mV, Amygdala
Time since AD diagnosis (months)
Sample Size
10
20
30
50
90
Legend
Average Value
Spline

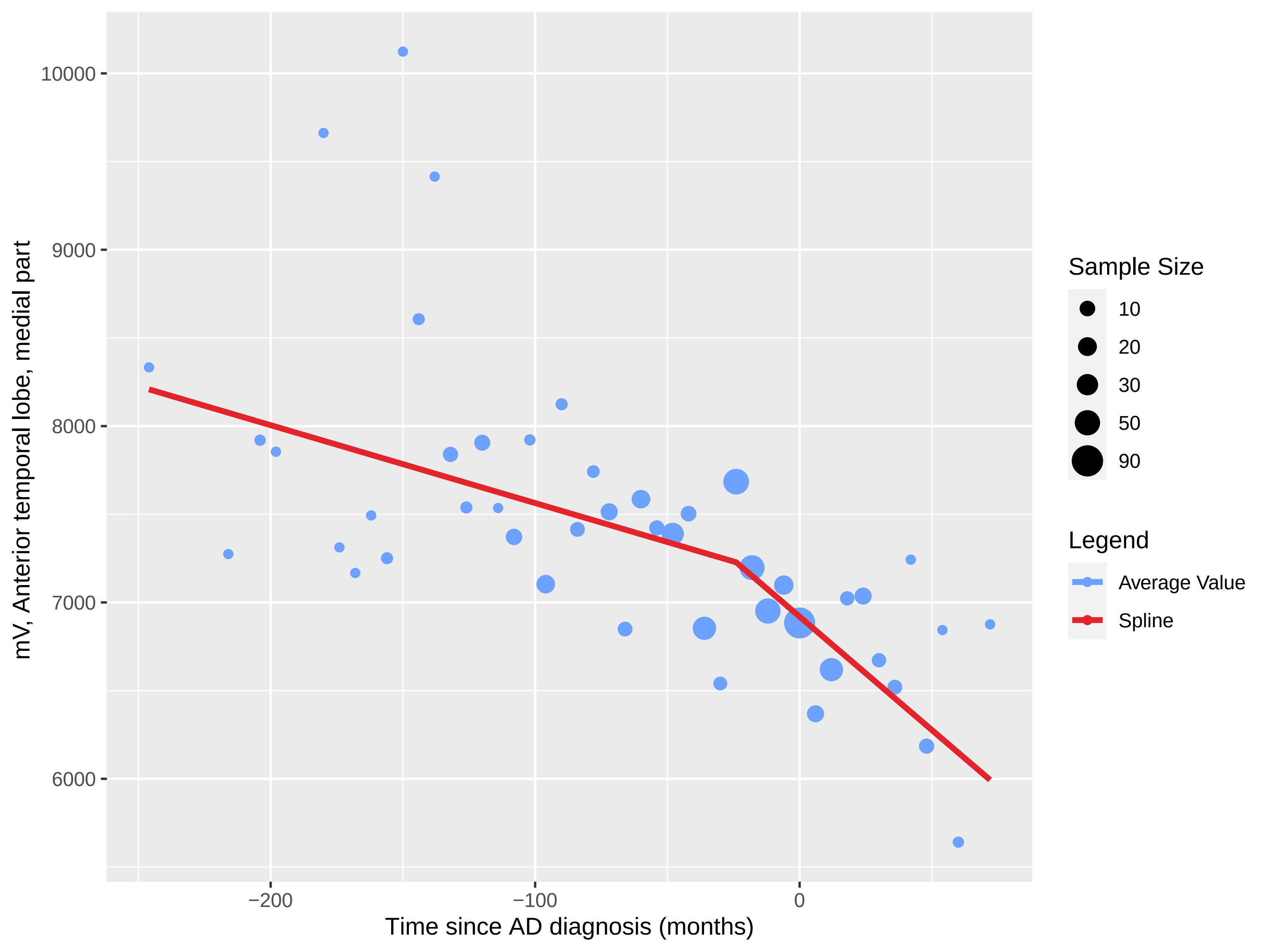

10000
9000
8000
7000
6000
−200
−100
0
mV, Anterior temporal lobe, medial part
Time since AD diagnosis (months)
Sample Size
10
20
30
50
90
Legend
Average Value
Spline

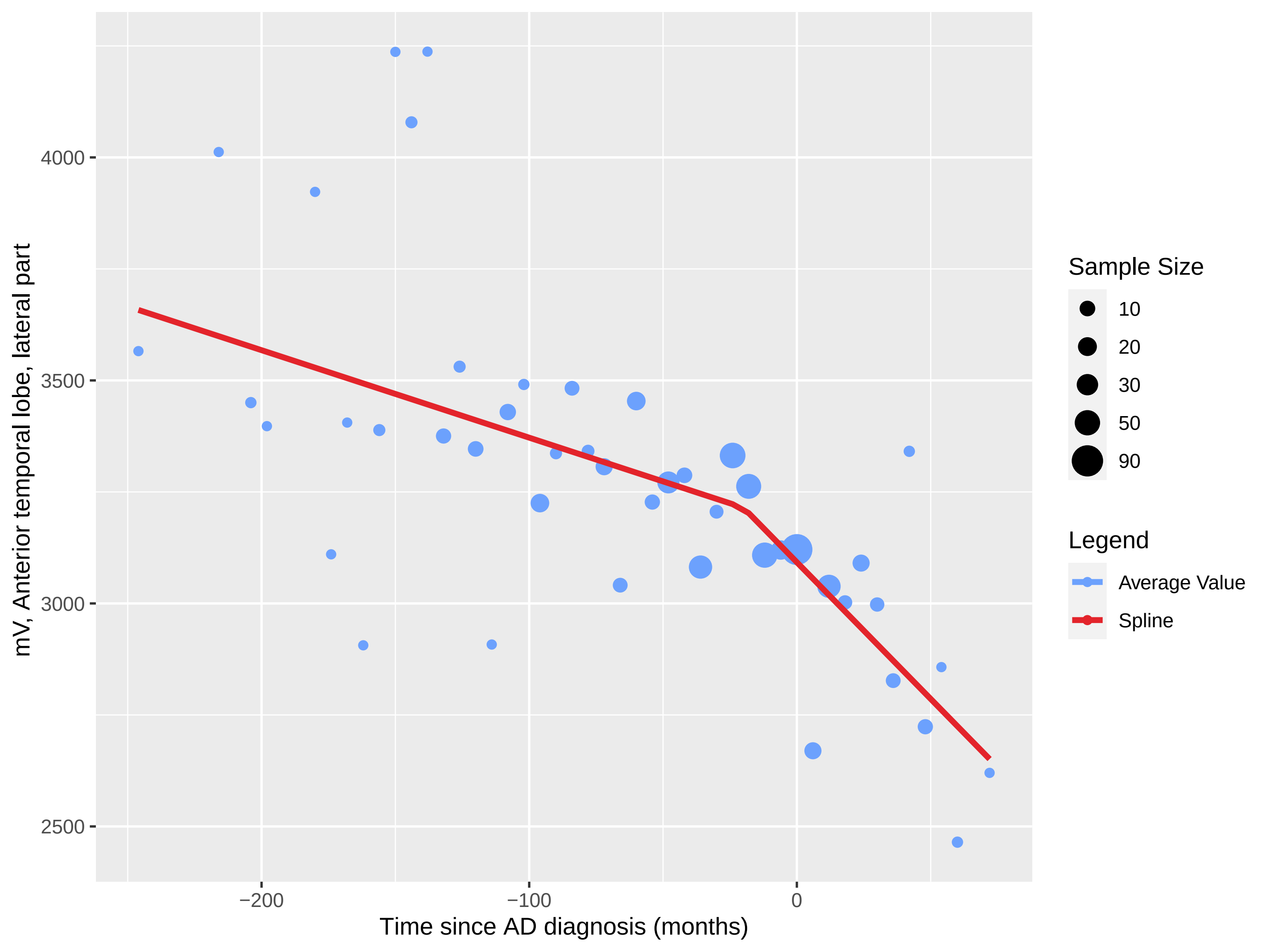

mV, Anterior temporal lobe, lateral part
4000
3500
3000
2500
−200
−100
0
Time since AD diagnosis (months)
Sample Size
10
20
30
50
90
Legend
Average Value
Spline

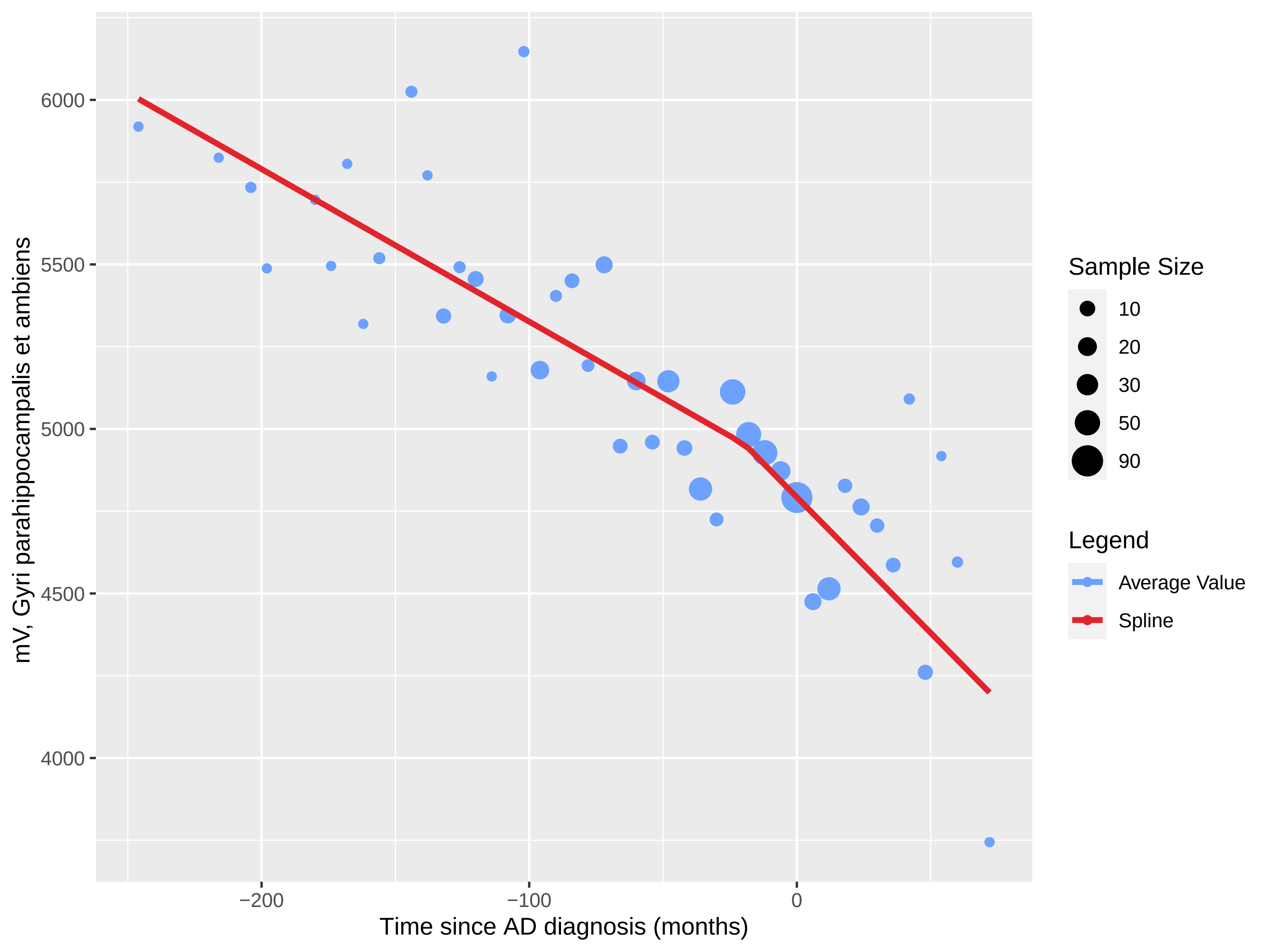

mV, Gyri parahippocampalis et ambiens
6000
5500
5000
4500
4000
−200
−100
0
Time since AD diagnosis (months)
Sample Size
10
20
30
50
90
Legend
Average Value
Spline

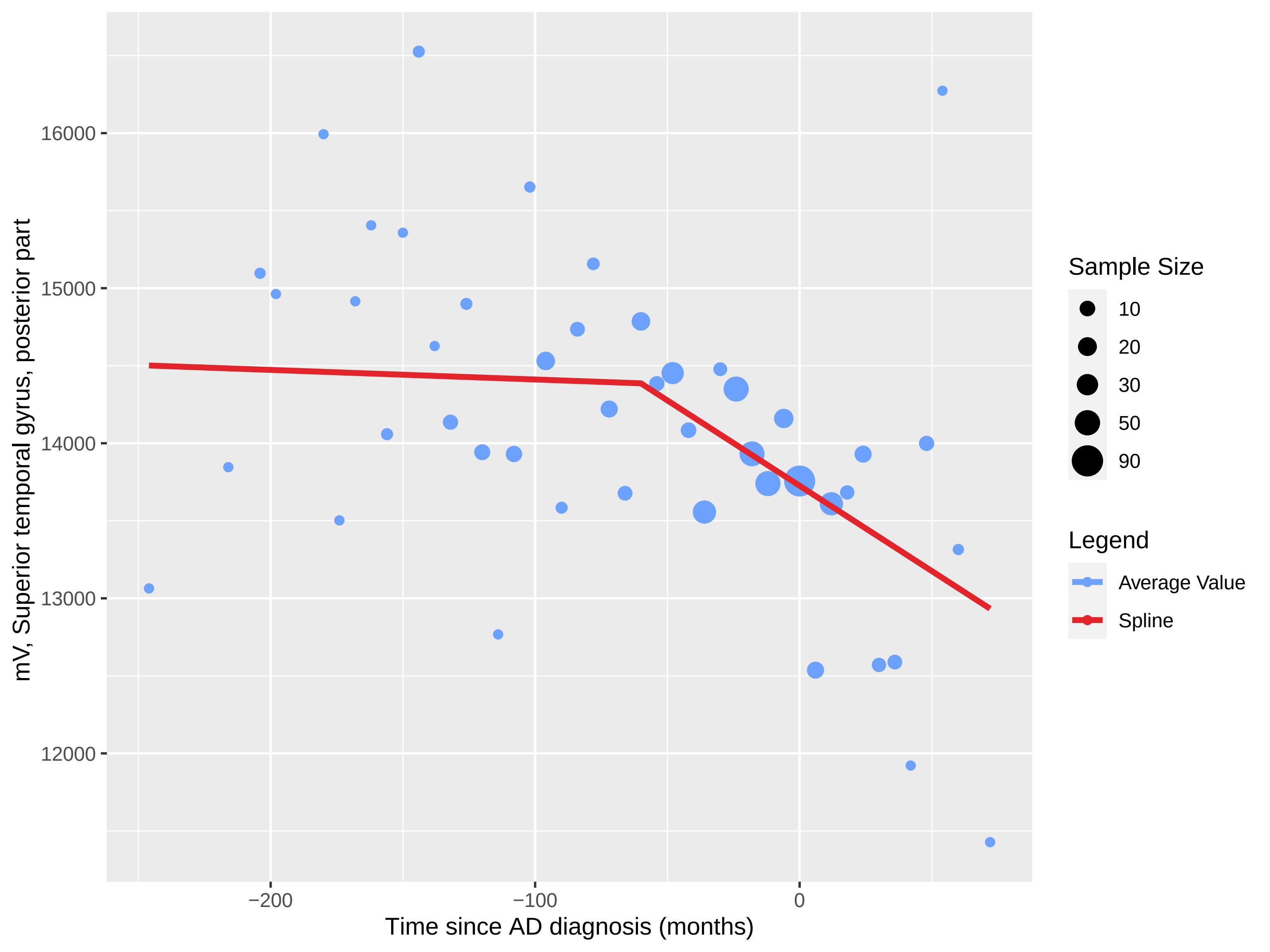

mV, Superior temporal gyrus, posterior part
16000
15000
14000
13000
12000
−200
−100
0
Time since AD diagnosis (months)
Sample Size
10
20
30
50
90
Legend
Average Value
Spline

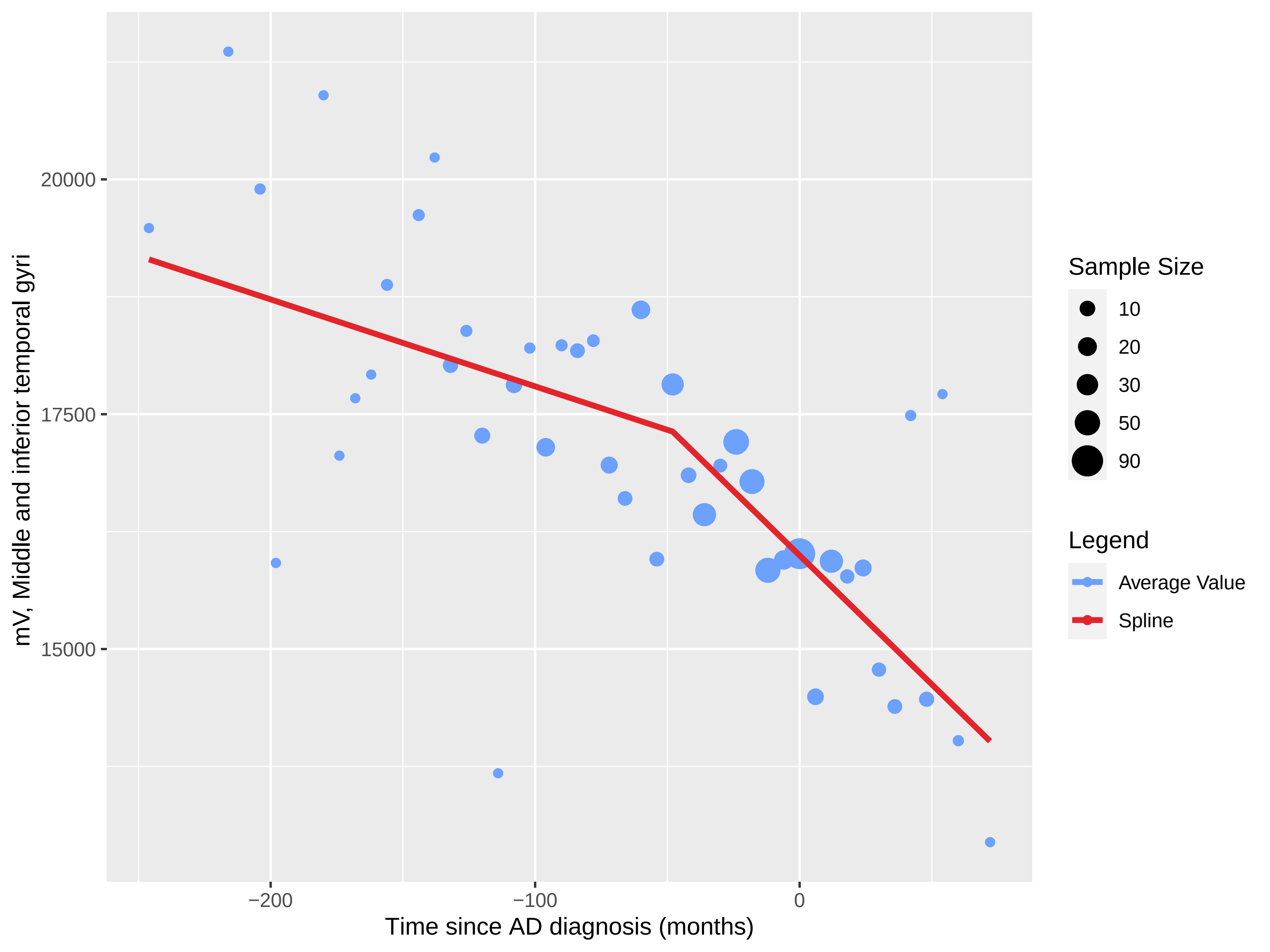

20000
17500
15000
−200
−100
0
mV, Middle and inferior temporal gyri
Time since AD diagnosis (months)
Sample Size
10
20
30
50
90
Legend
Average Value
Spline

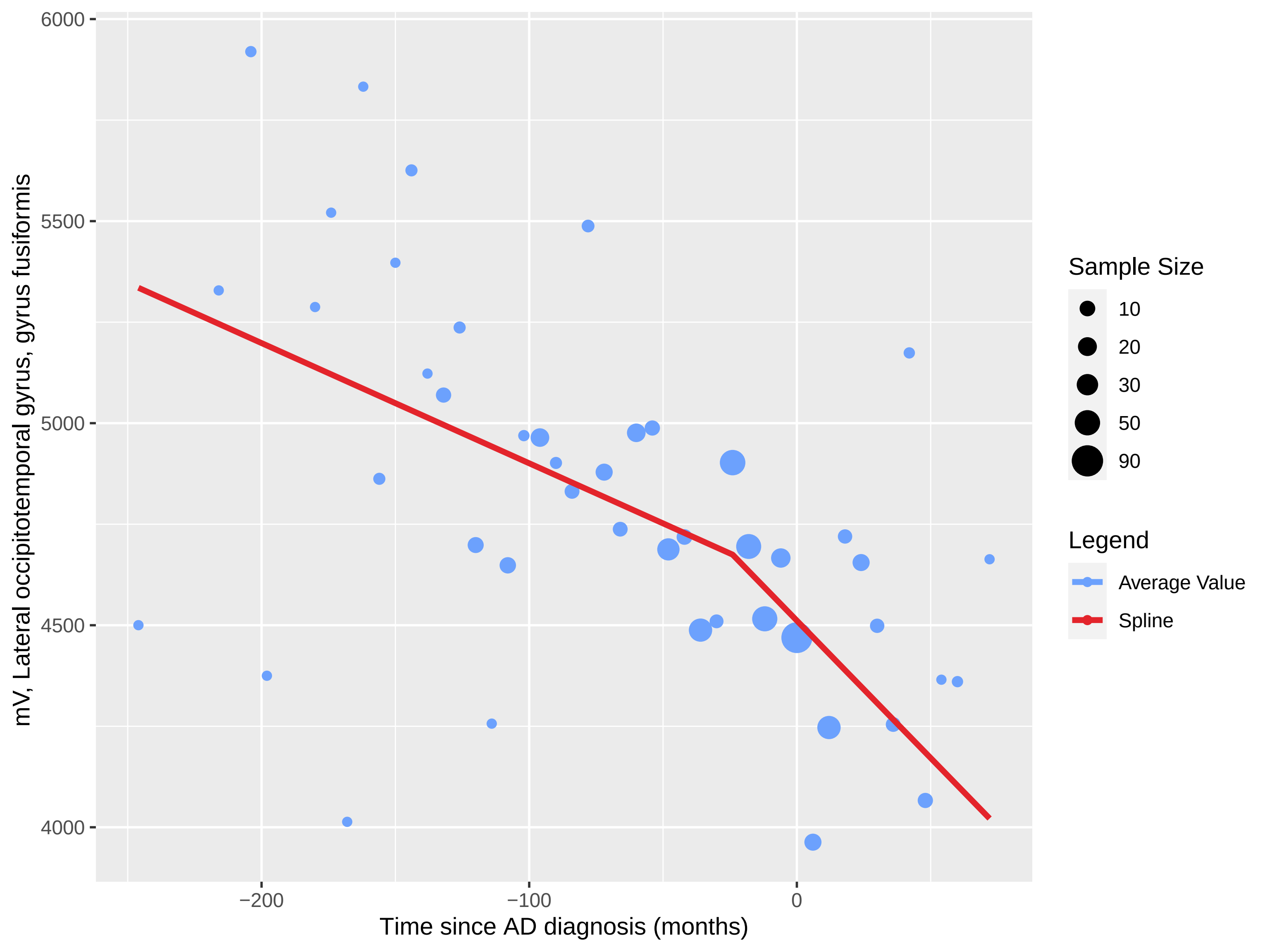

6000
5500
5000
4500
4000
mV, Lateral occipitotemporal gyrus, gyrus fusiformis
−200
−100
0
Time since AD diagnosis (months)
Sample Size
10
20
30
50
90
Legend
Average Value
Spline

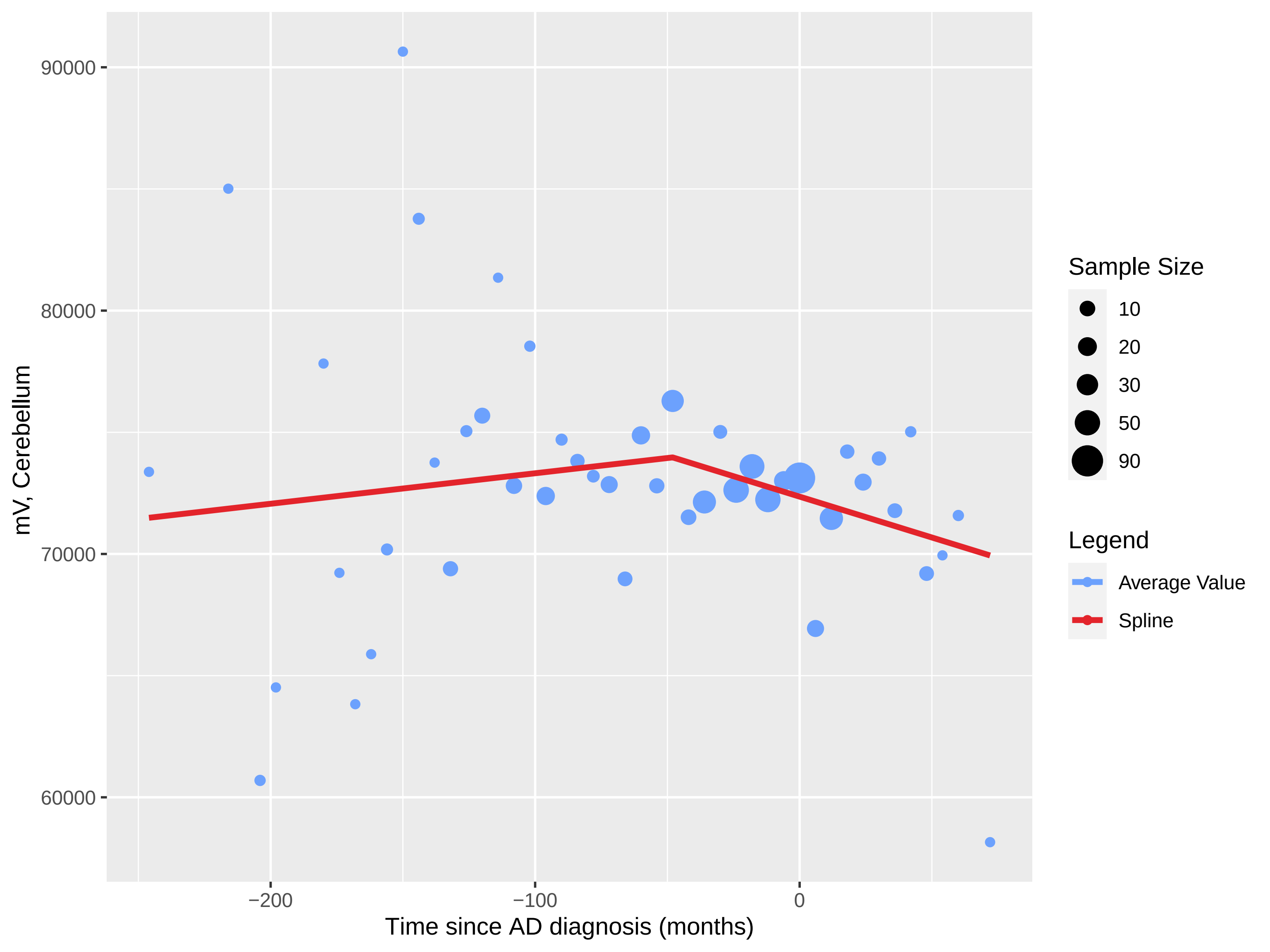

90000
80000
70000
60000
mV, Cerebellum
−200
−100
0
Time since AD diagnosis (months)
Sample Size
10
20
30
50
90
Legend
Average Value
Spline

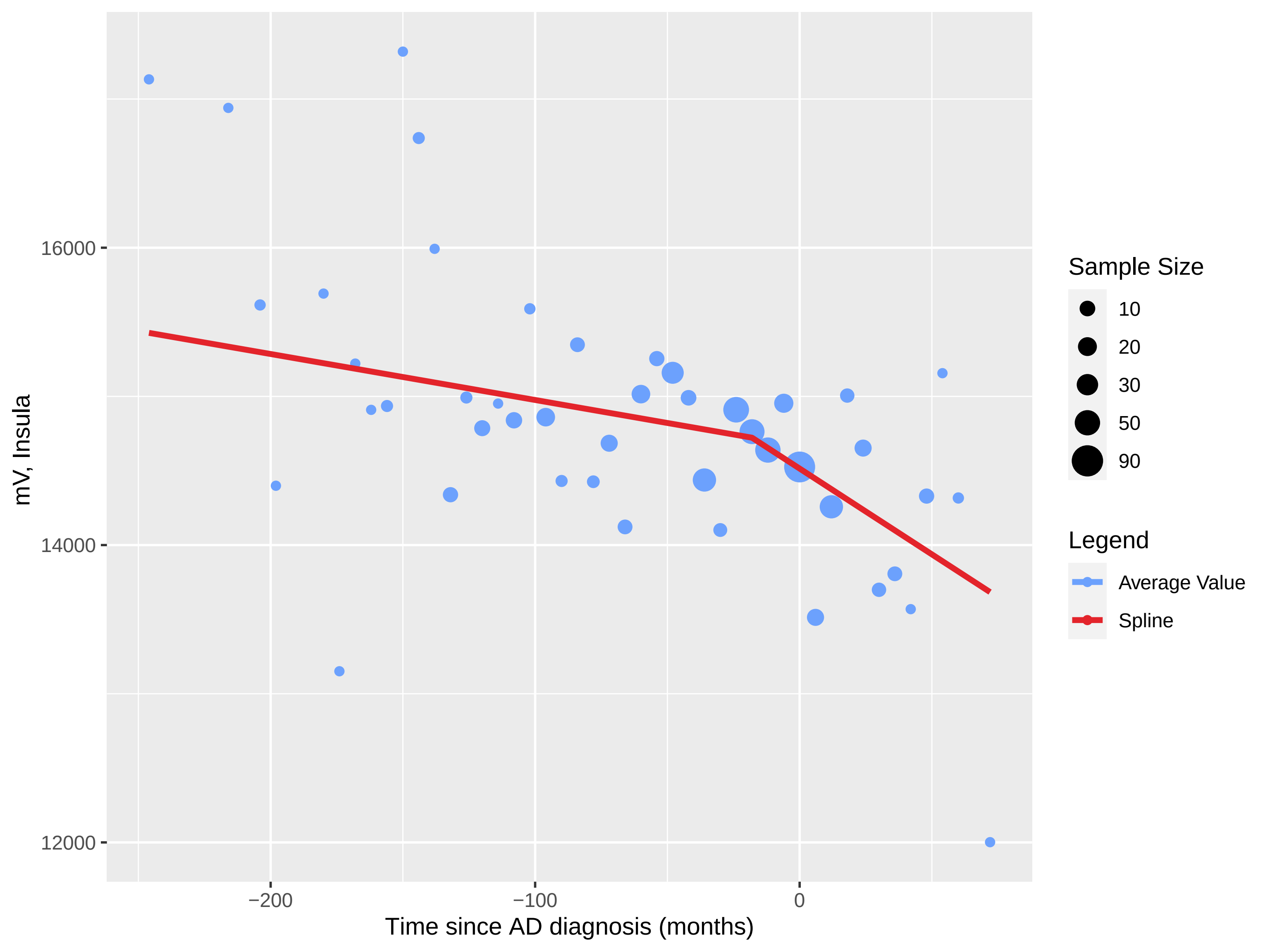

16000
14000
12000
−200
−100
0
mV, Insula
Time since AD diagnosis (months)
Sample Size
10
20
30
50
90
Legend
Average Value
Spline

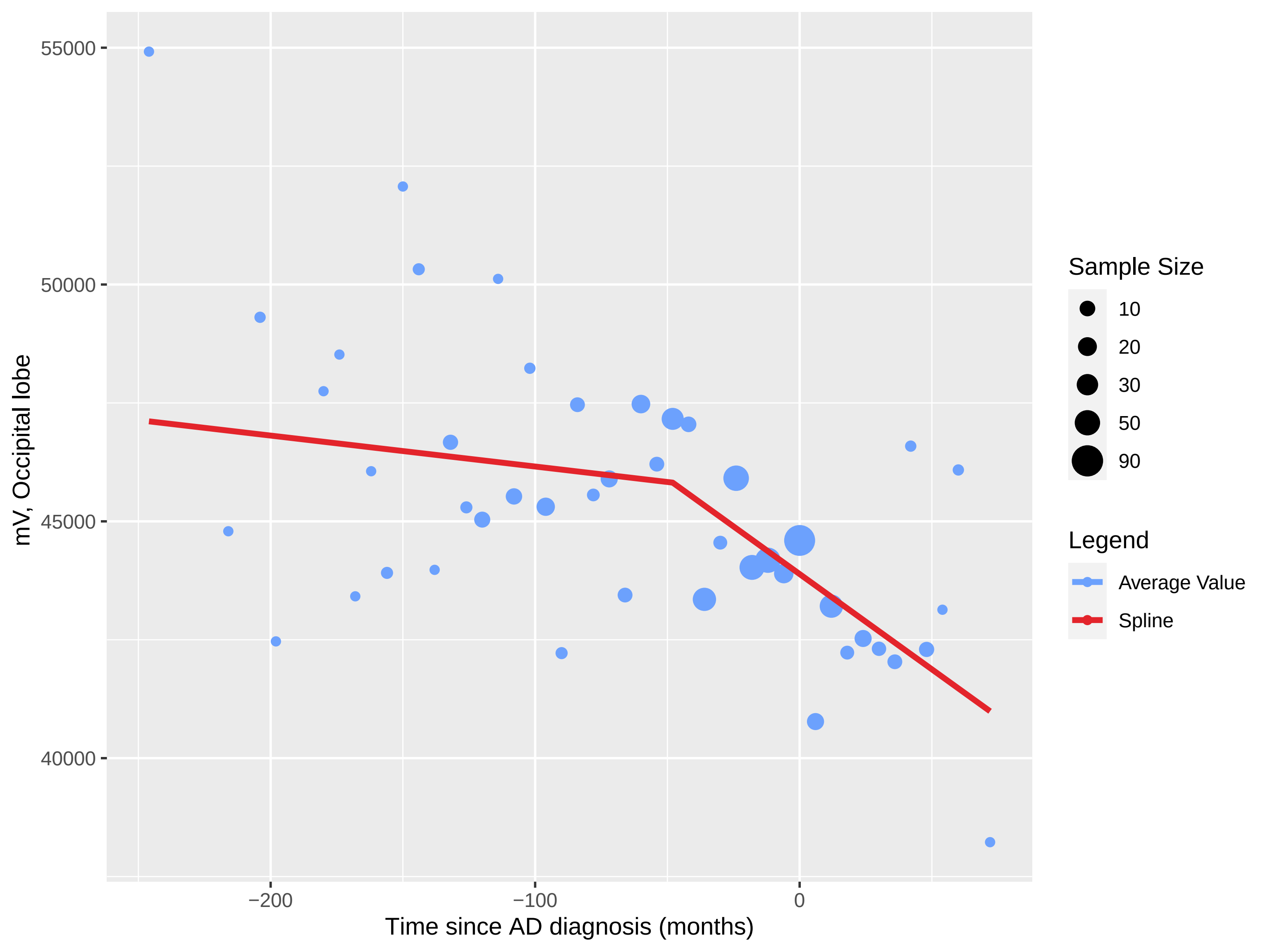

55000
50000
45000
40000
mV, Occipital lobe
−200
−100
0
Time since AD diagnosis (months)
Sample Size
10
20
30
50
90
Legend
Average Value
Spline

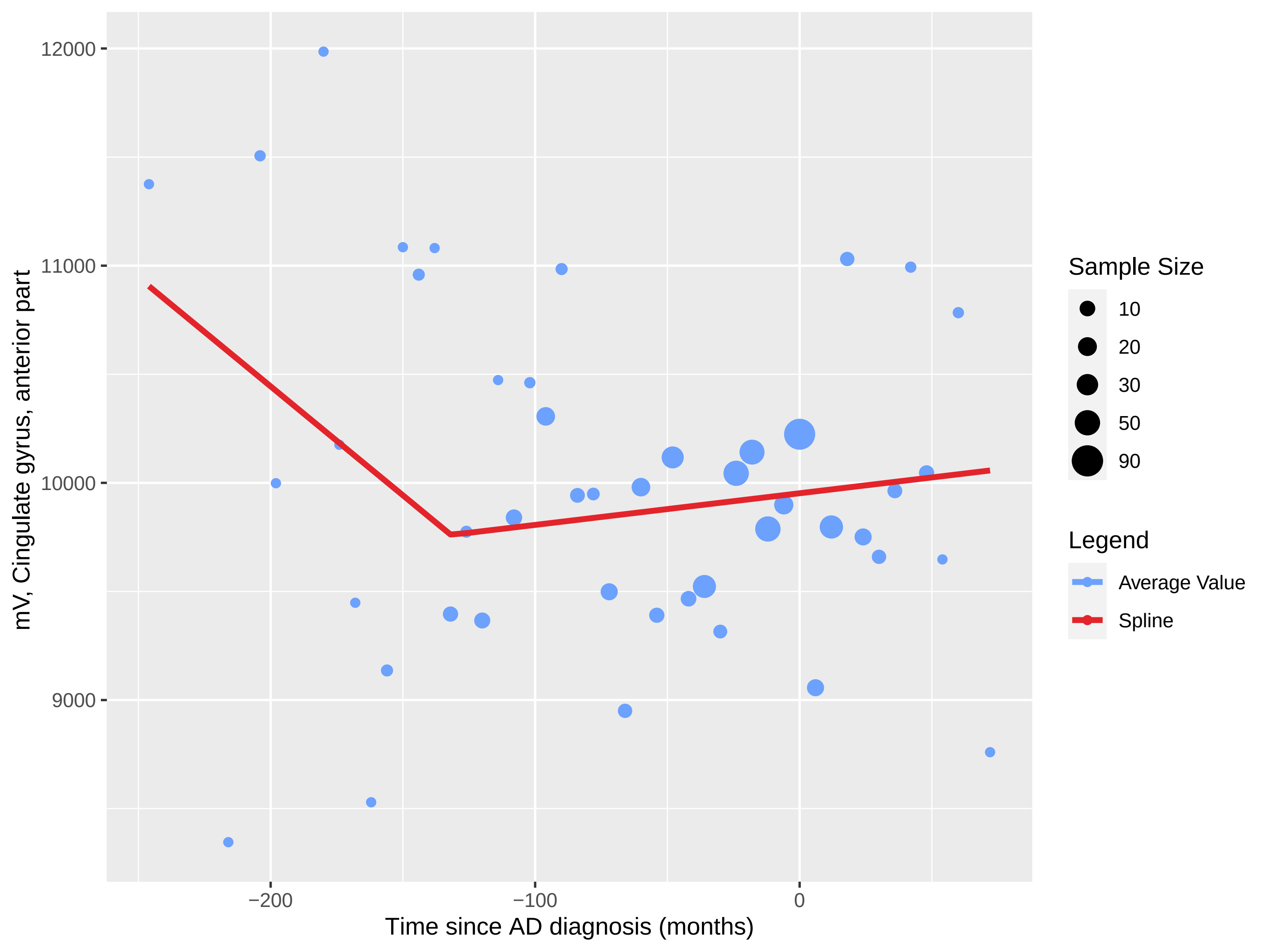

12000
11000
10000
9000
mV, Cingulate gyrus, anterior part
−200
−100
0
Time since AD diagnosis (months)
Sample Size
10
20
30
50
90
Legend
Average Value
Spline

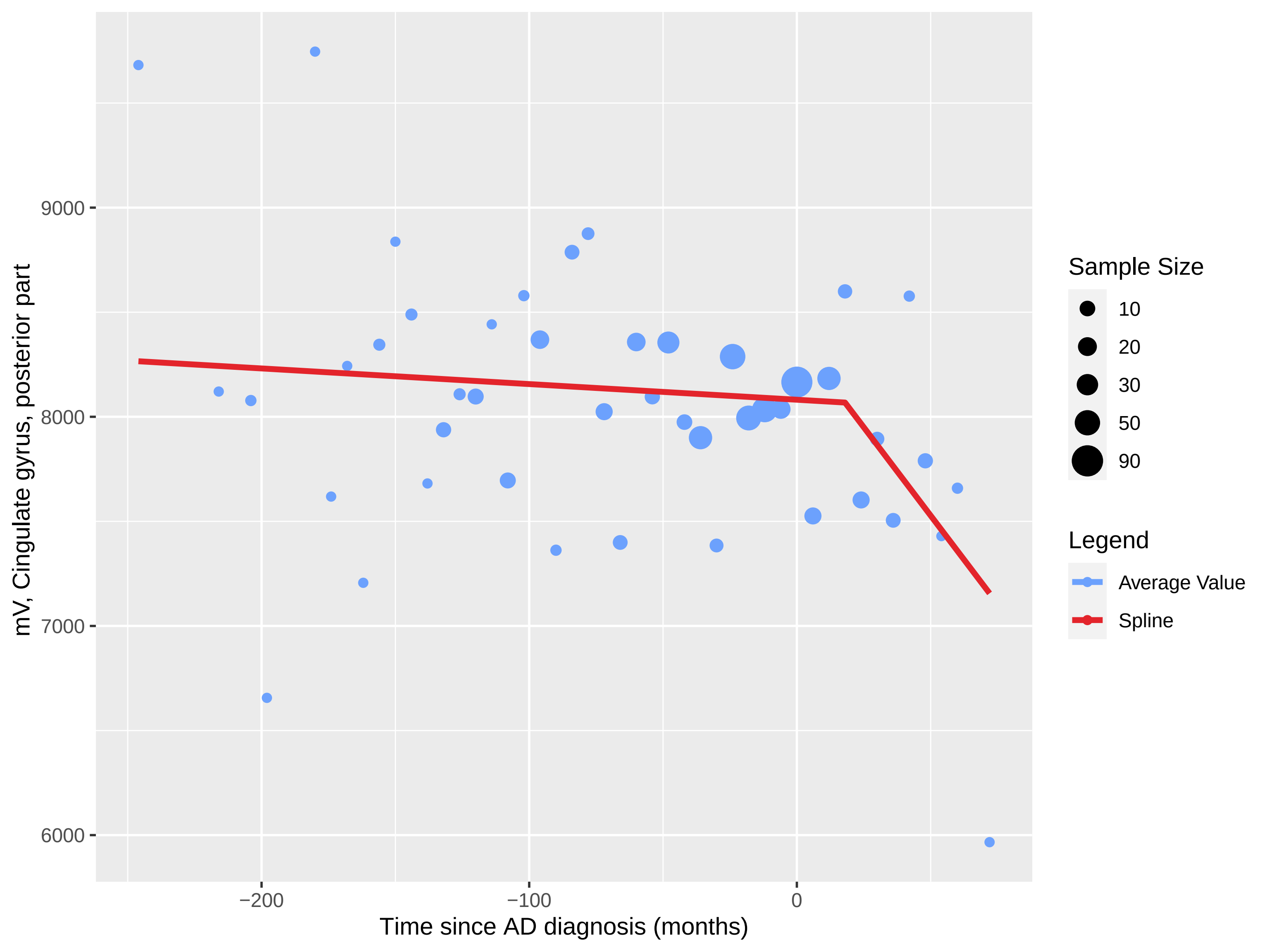

mV, Cingulate gyrus, posterior part
9000
8000
7000
6000
−200
−100
0
Time since AD diagnosis (months)
Sample Size
10
20
30
50
90
Legend
Average Value
Spline

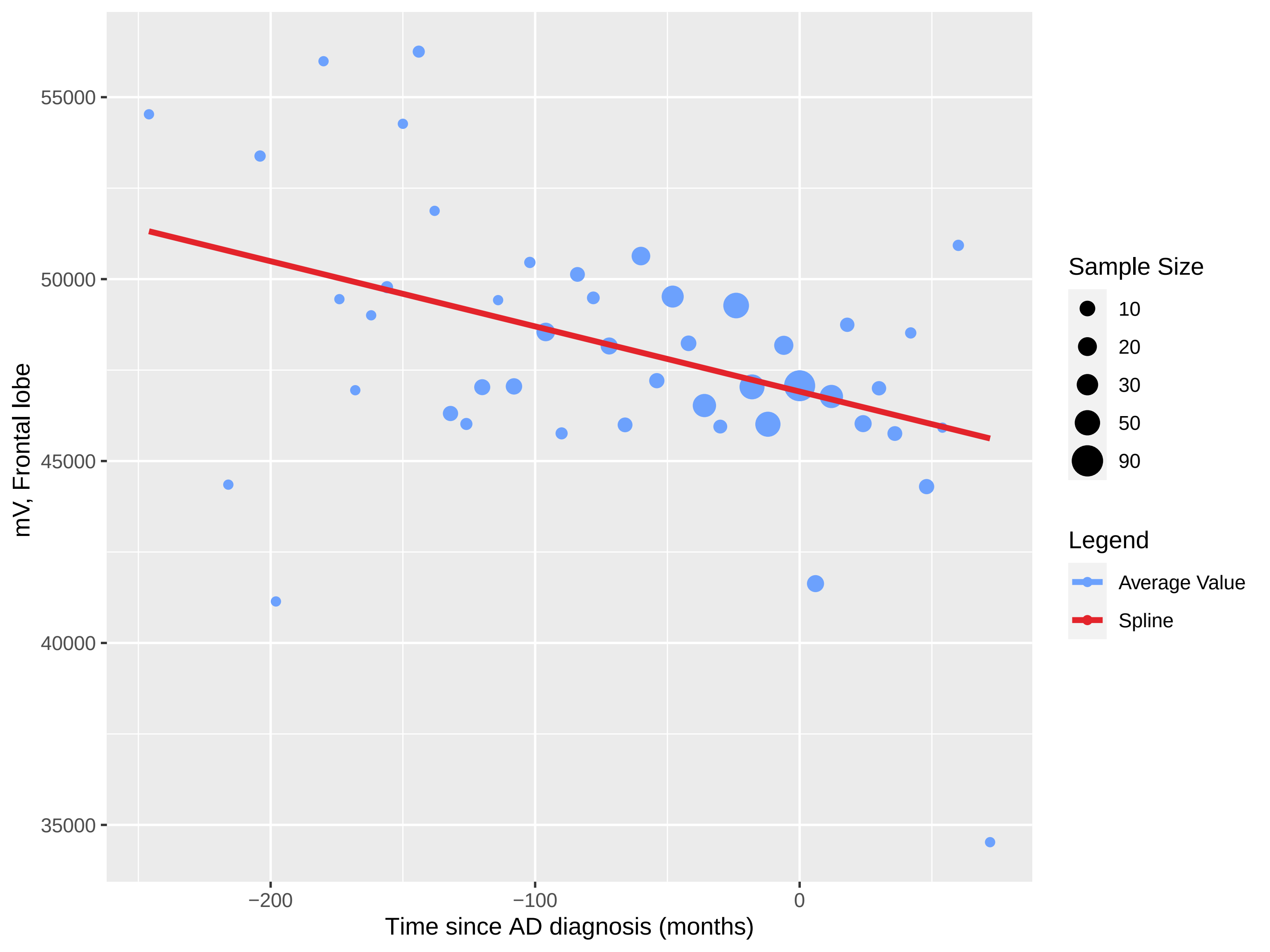

55000
50000
45000
40000
35000
mV, Frontal lobe
−200
−100
0
Time since AD diagnosis (months)
Sample Size
10
20
30
50
90
Legend
Average Value
Spline

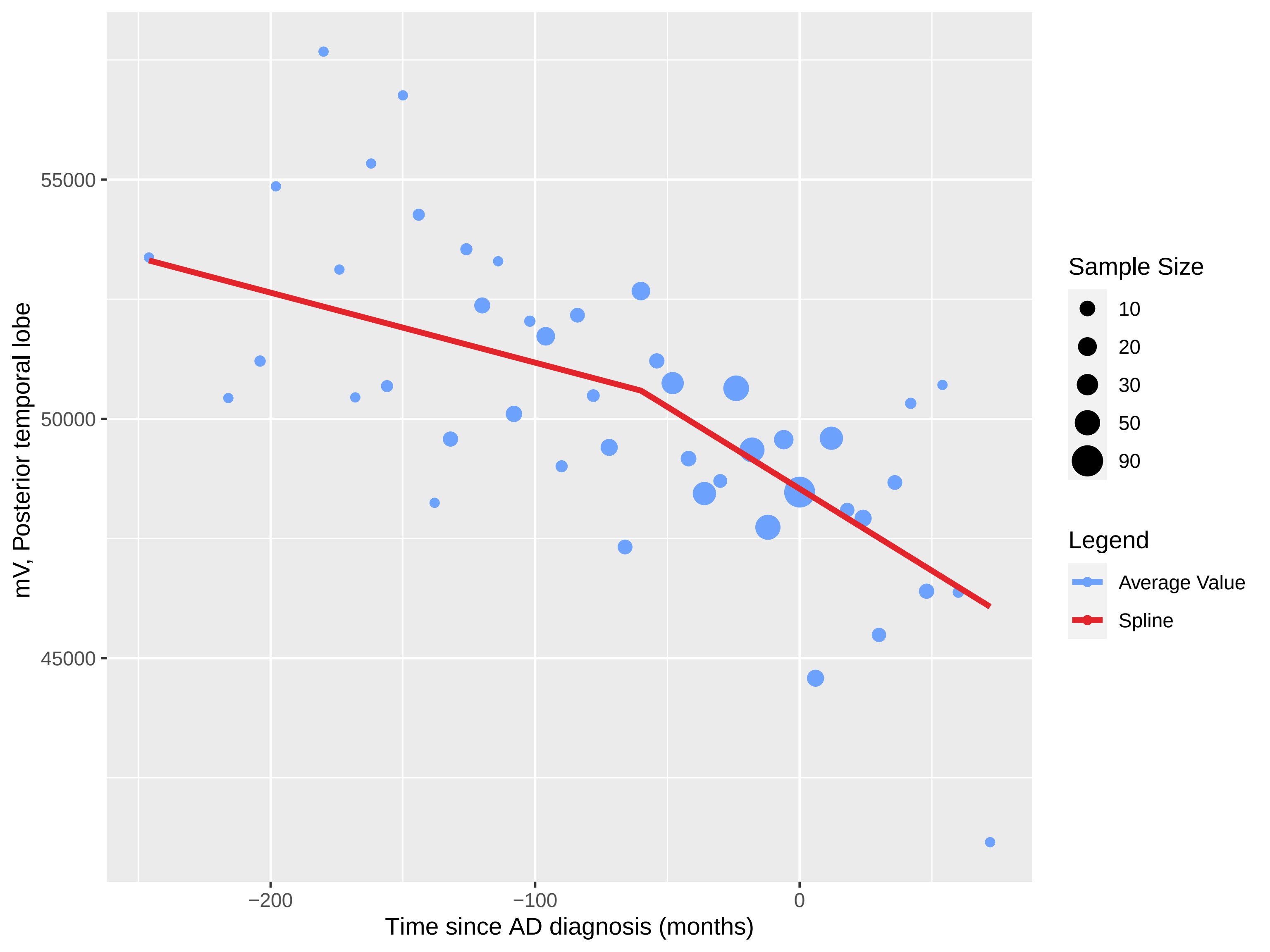

55000
50000
45000
mV, Posterior temporal lobe
−200
−100
0
Time since AD diagnosis (months)
Sample Size
10
20
30
50
90
Legend
Average Value
Spline

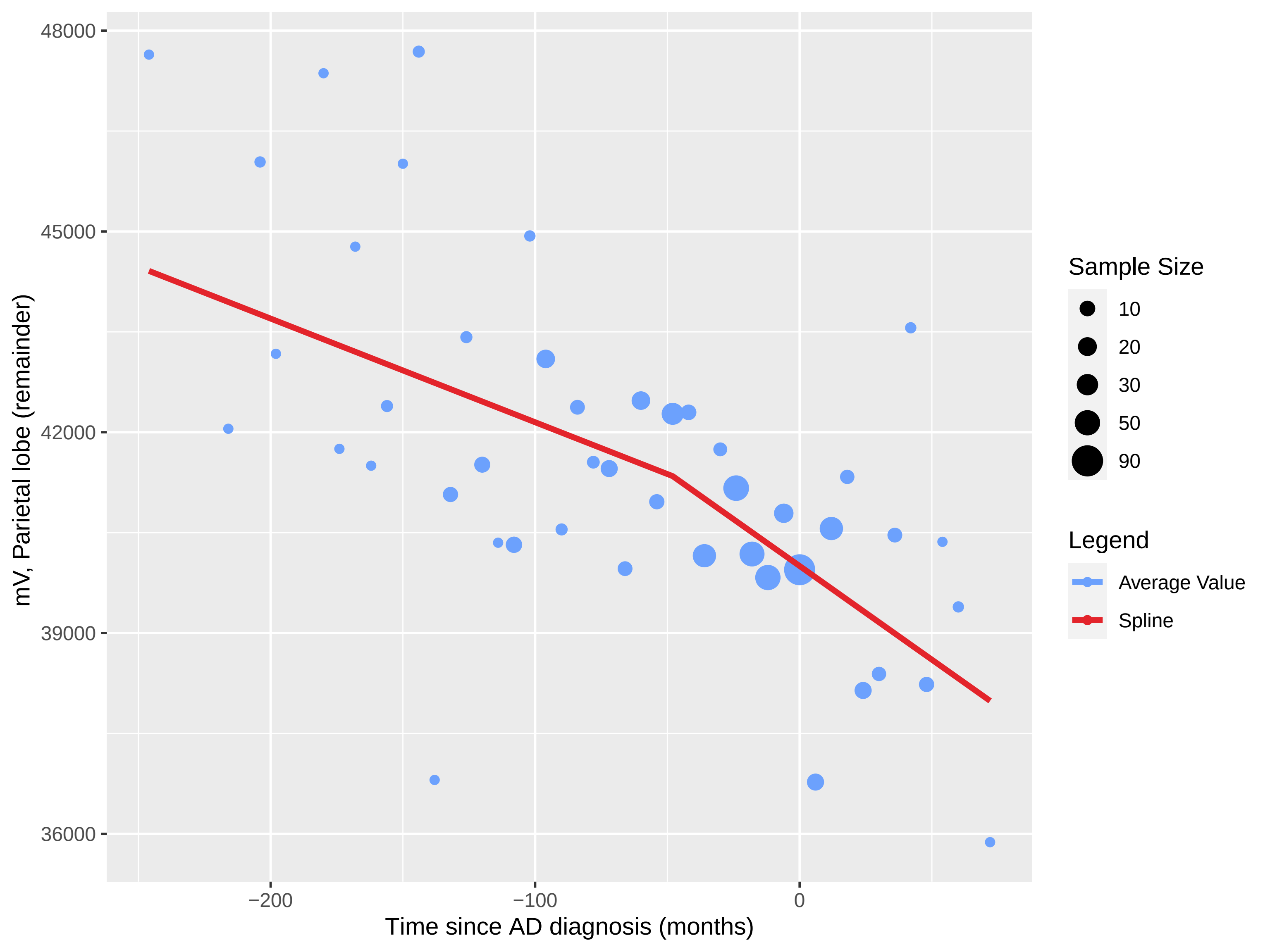

48000
45000
42000
39000
36000
mV, Parietal lobe (remainder)
−200
−100
0
Time since AD diagnosis (months)
Sample Size
10
20
30
50
90
Legend
Average Value
Spline

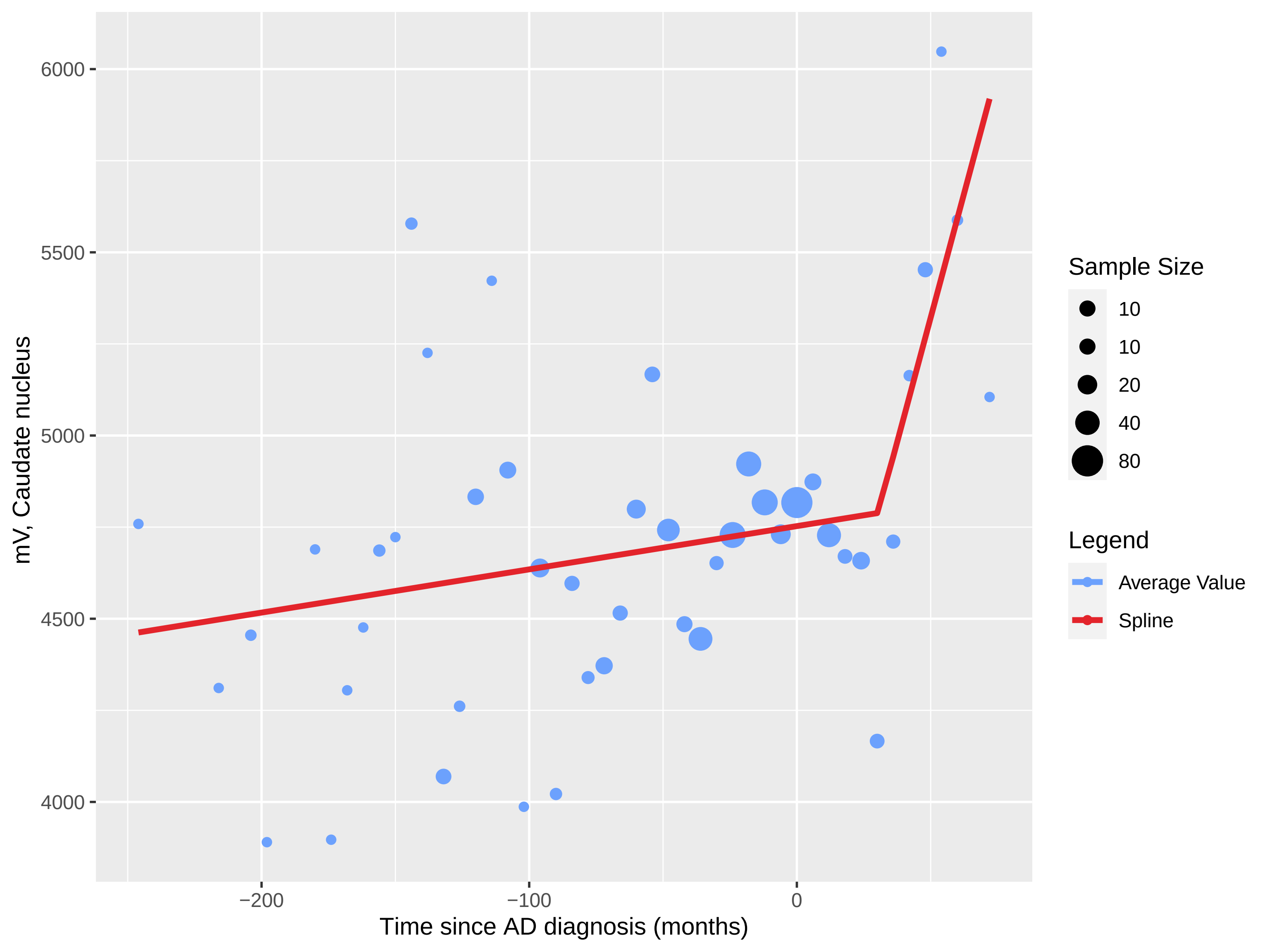

6000
5500
5000
4500
4000
mV, Caudate nucleus
−200
−100
0
Time since AD diagnosis (months)
Sample Size
10
10
20
40
80
Legend
Average Value
Spline

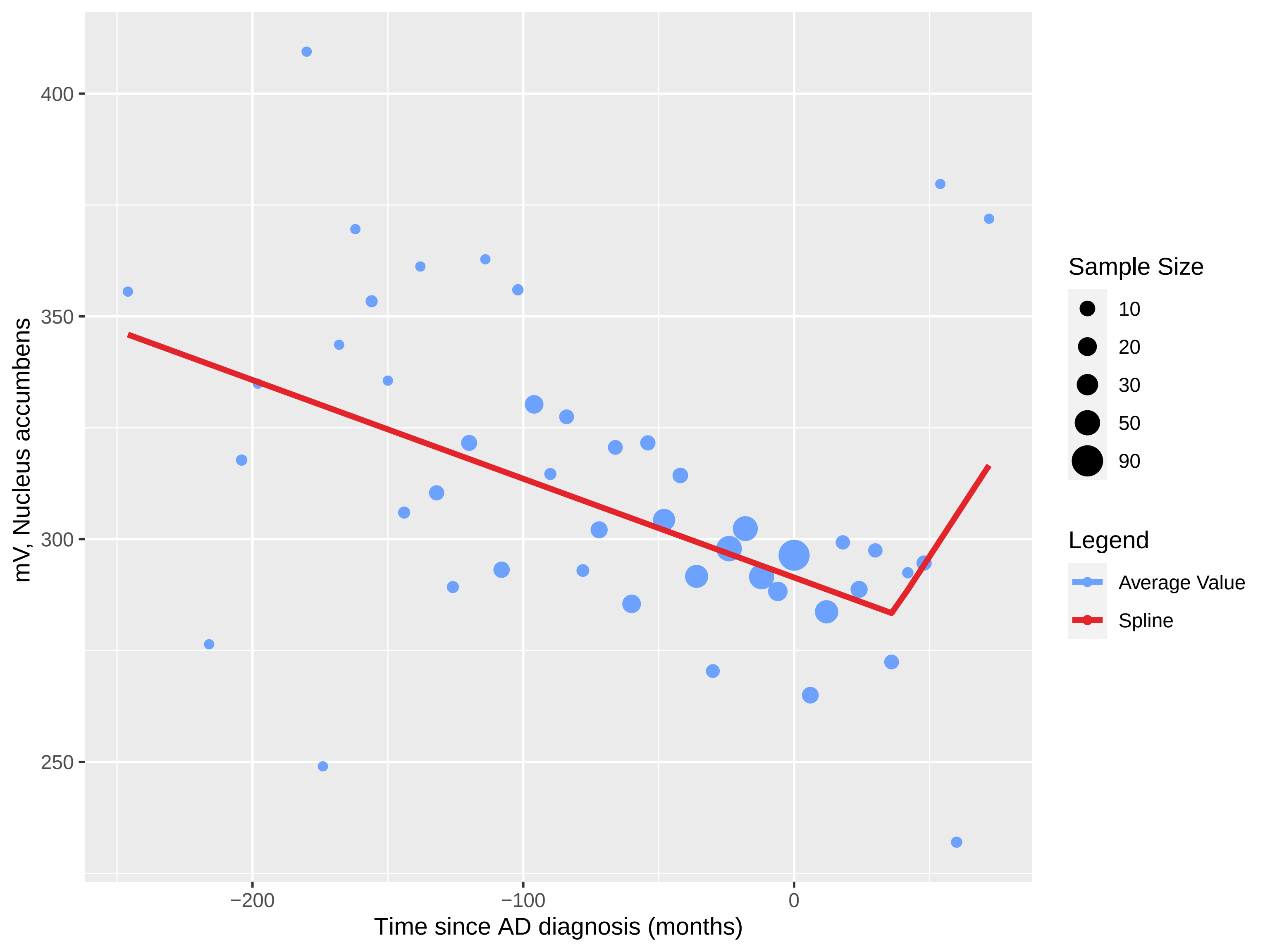

400
350
300
250
mV, Nucleus accumbens
−200
−100
0
Time since AD diagnosis (months)
Sample Size
10
20
30
50
90
Legend
Average Value
Spline

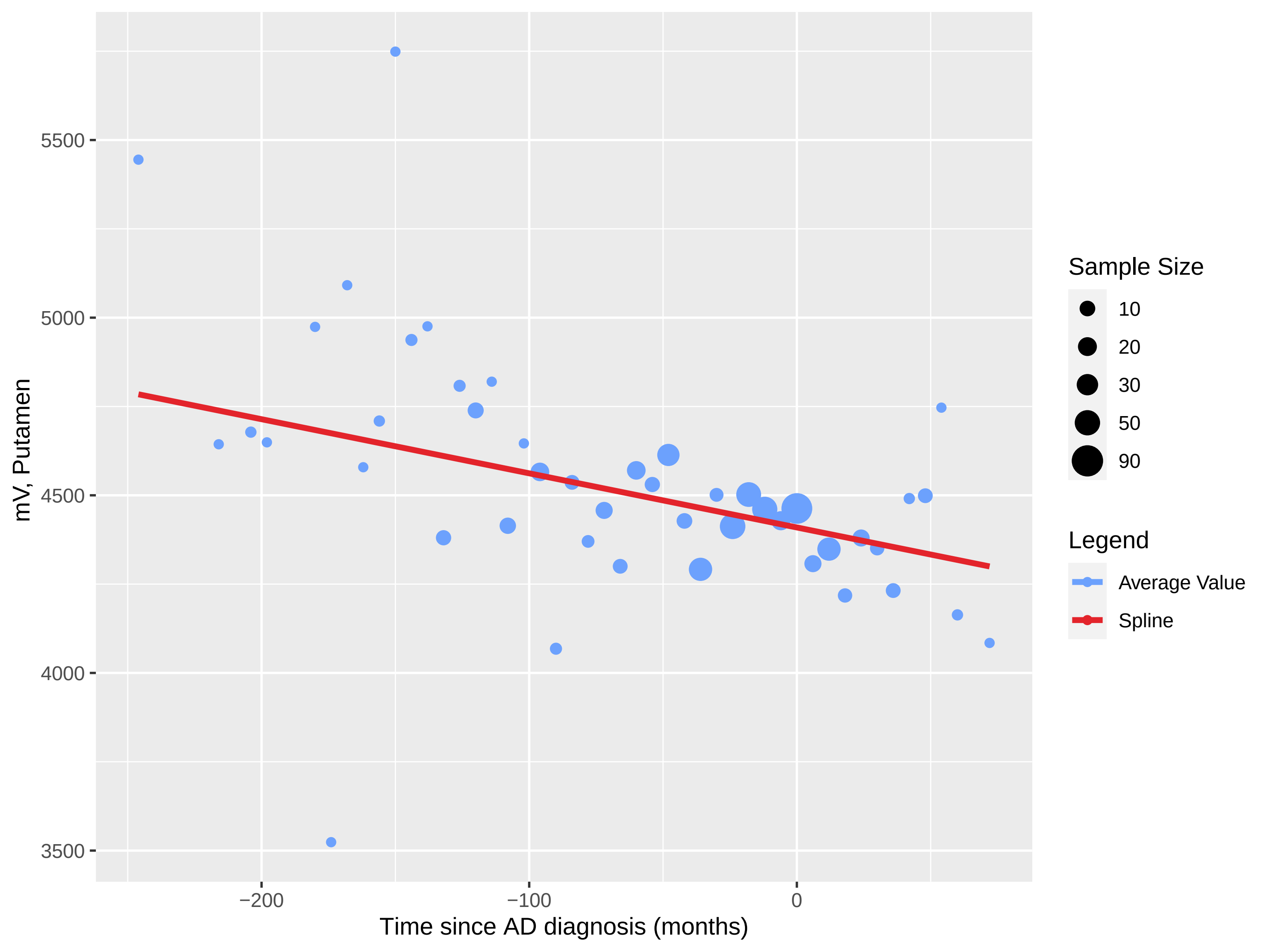

mV, Putamen
5500
5000
4500
4000
3500
−200
−100
0
Time since AD diagnosis (months)
Sample Size
10
20
30
50
90
Legend
Average Value
Spline

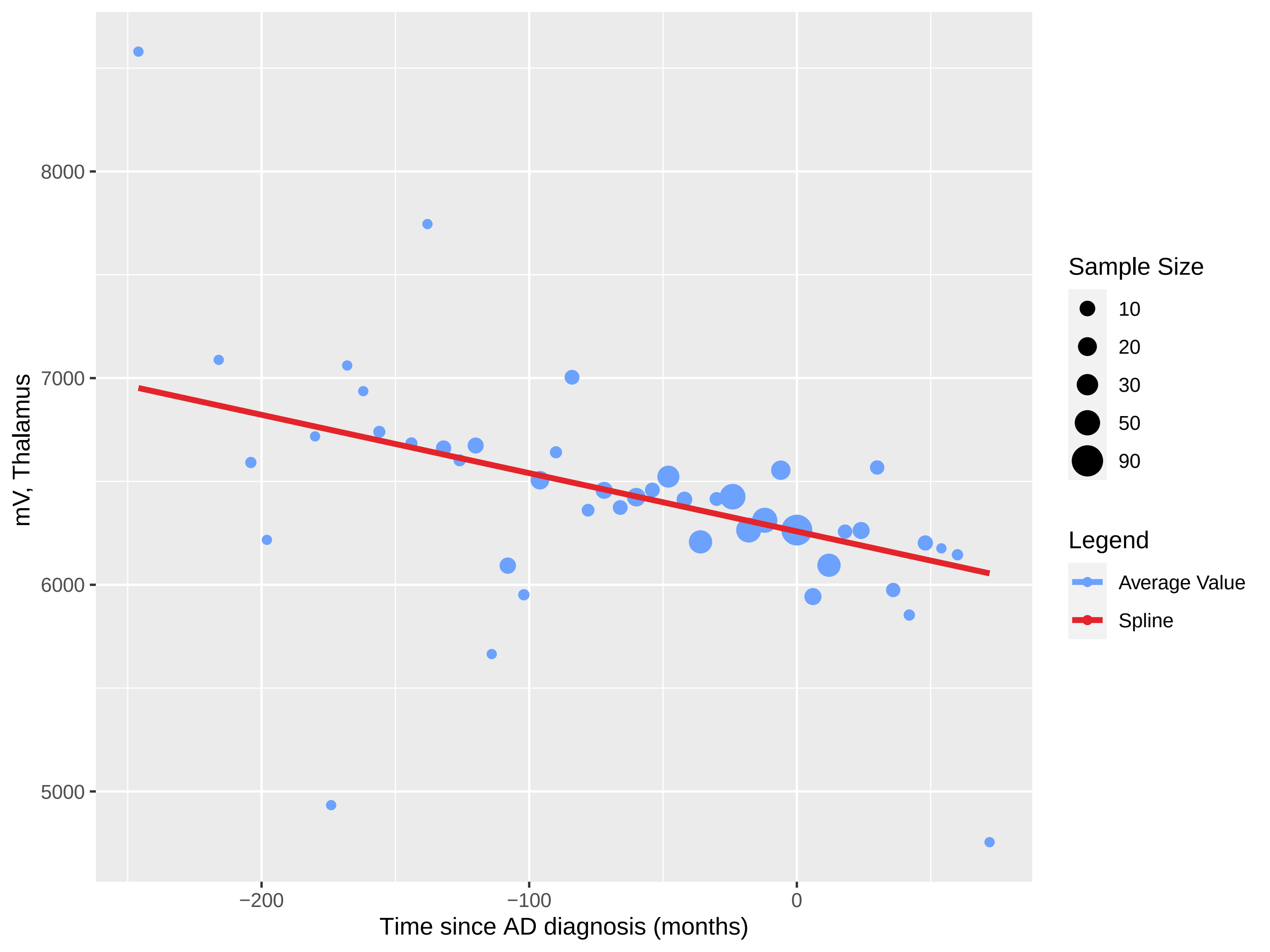

8000
7000
6000
5000
mV, Thalamus
−200
−100
0
Time since AD diagnosis (months)
Sample Size
10
20
30
50
90
Legend
Average Value
Spline

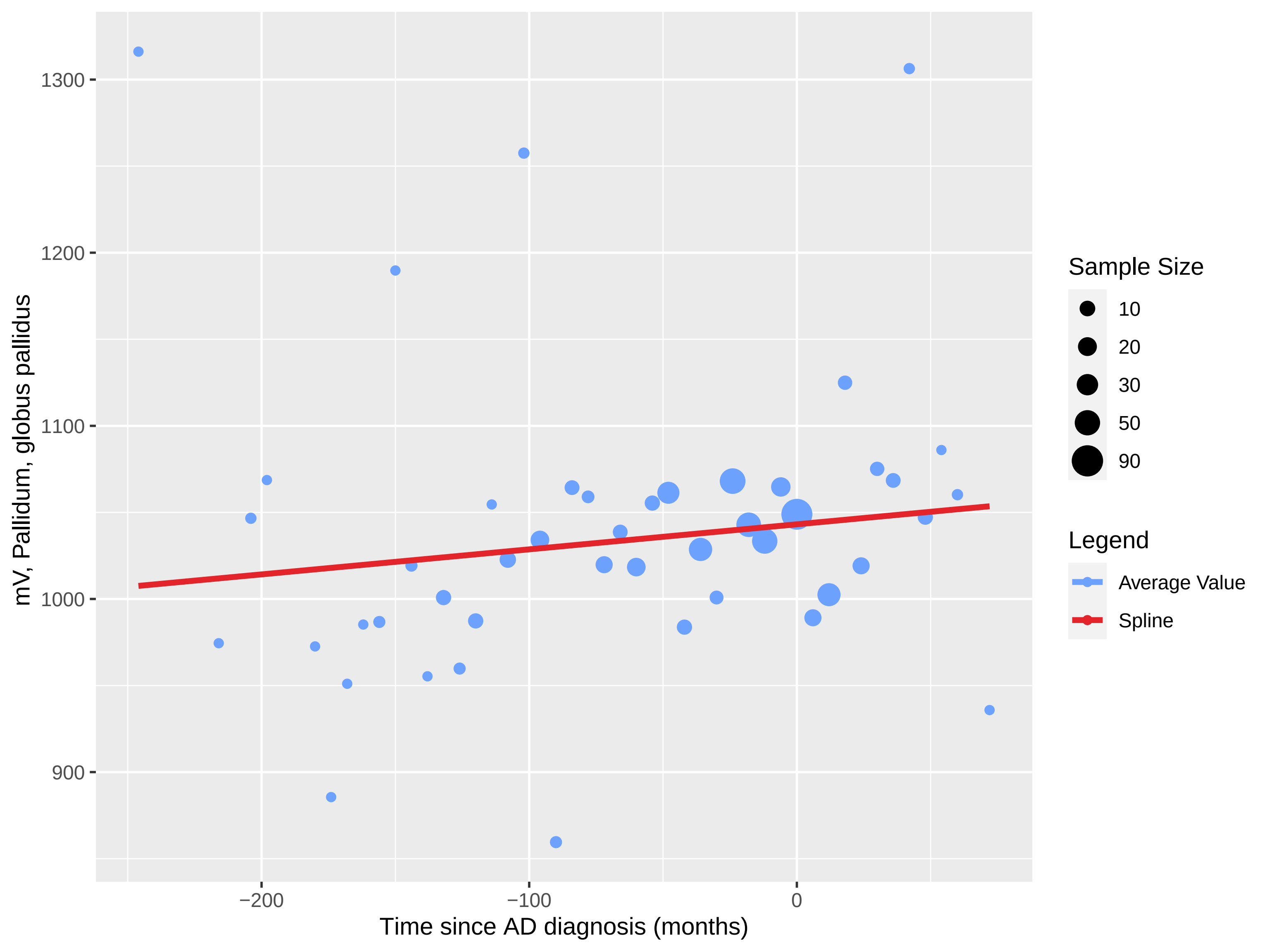

1300
1200
1100
1000
900
−200
−100
0
Time since AD diagnosis (months)
mV, Pallidum, globus pallidus
Sample Size
10
20
30
50
90
Legend
Average Value
Spline

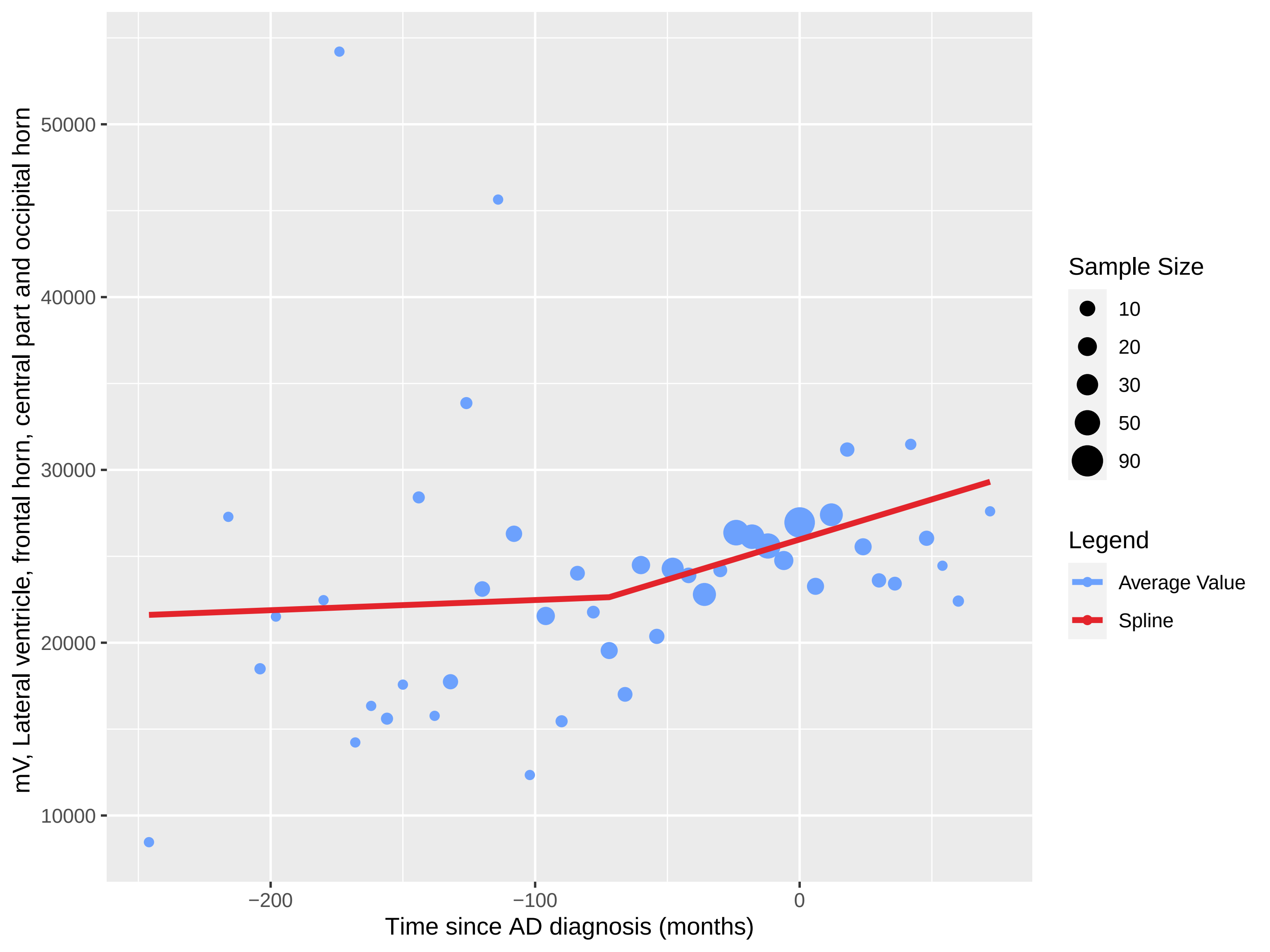

mV, Lateral ventricle, frontal horn, central part and occipital horn
50000
40000
30000
20000
10000
−200
−100
0
Time since AD diagnosis (months)
Sample Size
10
20
30
50
90
Legend
Average Value
Spline

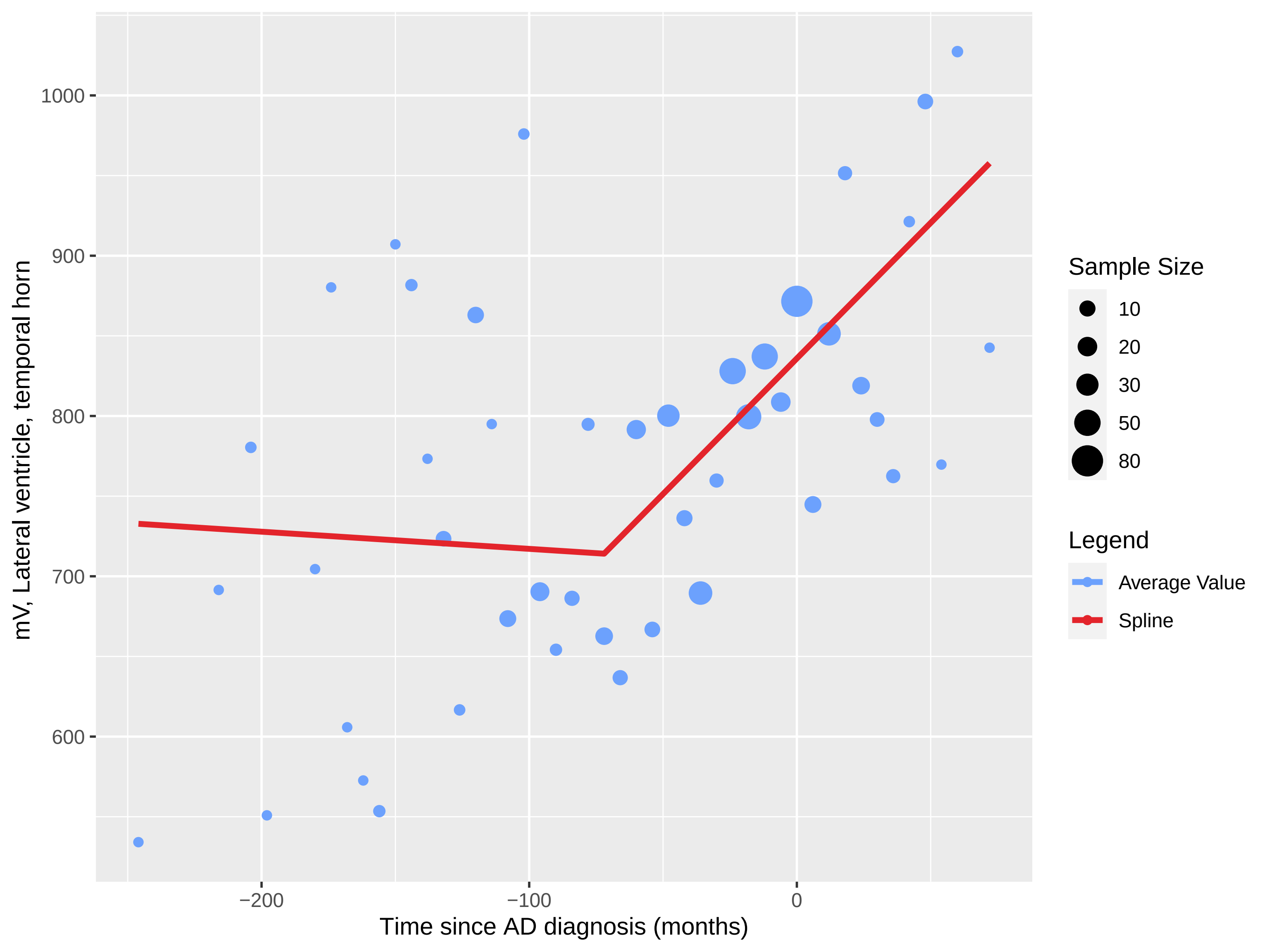

mV, Lateral ventricle, temporal horn
1000
900
800
700
600
−200
−100
0
Time since AD diagnosis (months)
Sample Size
10
20
30
50
80
Legend
Average Value
Spline

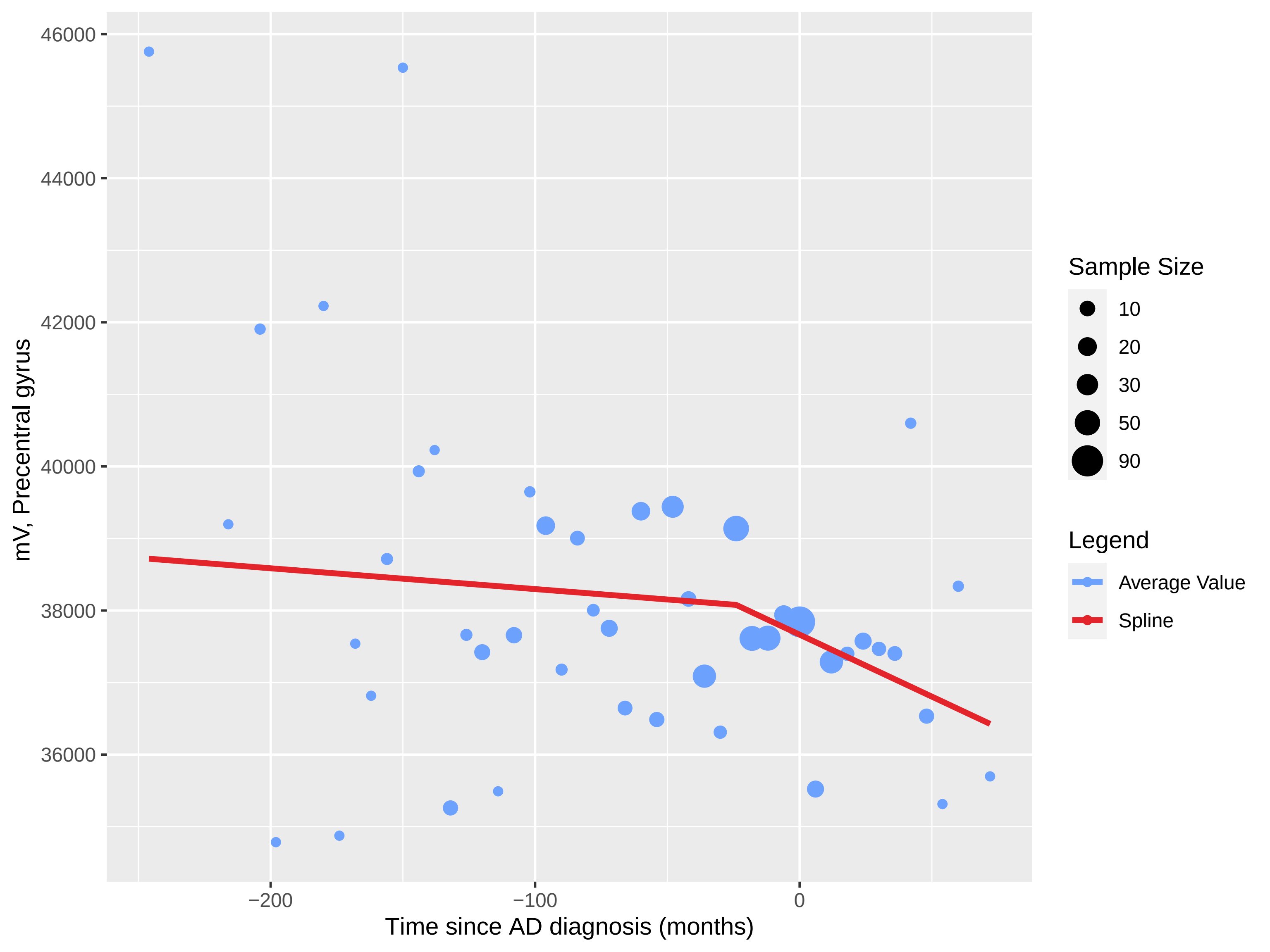
46000
44000
42000
40000
38000
36000
mV, Precentral gyrus
−200
−100
0
Time since AD diagnosis (months)
Sample Size
10
20
30
50
90
Legend
Average Value
Spline

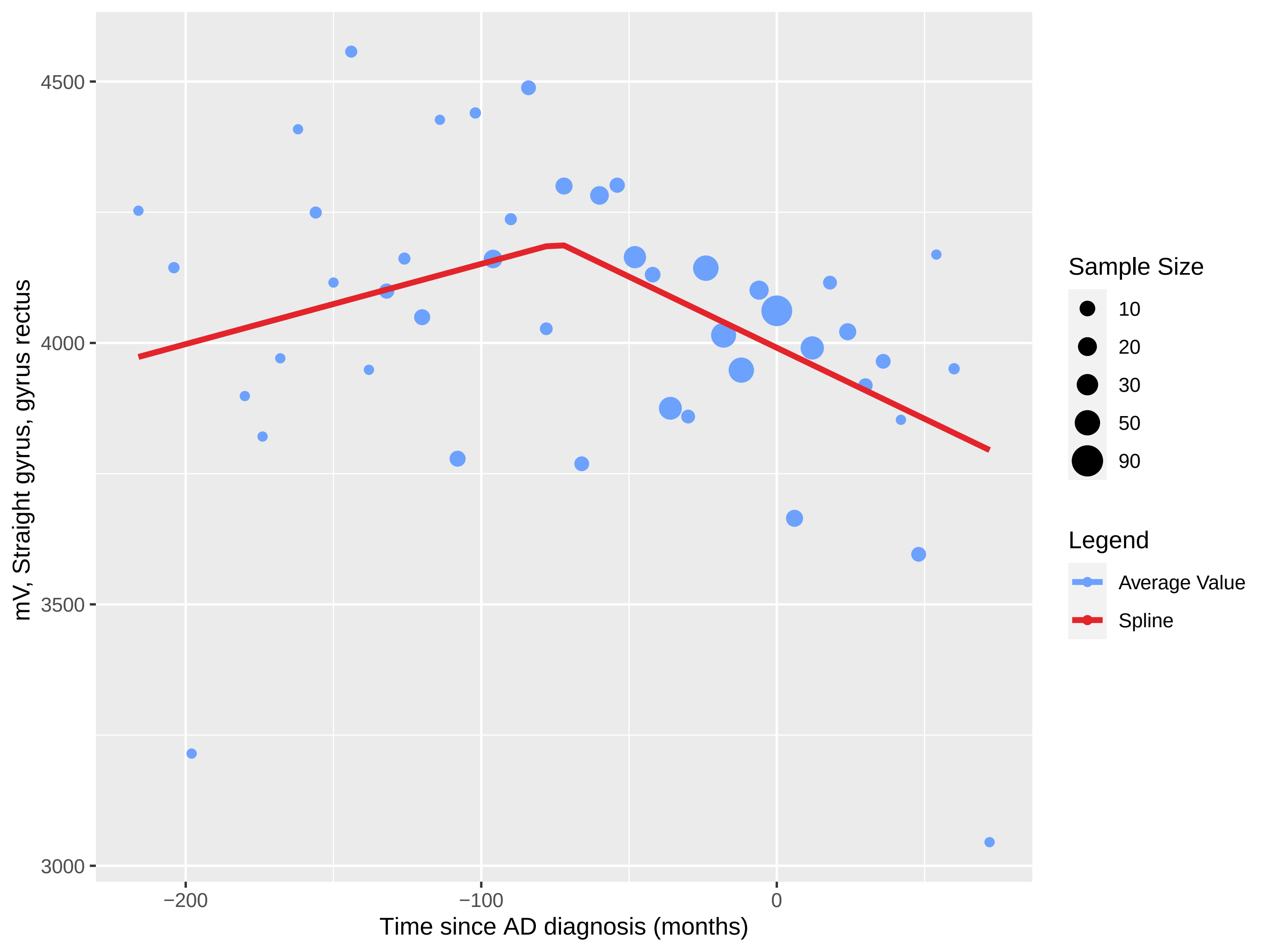

4500
4000
3500
3000
−200
−100
0
mV, Straight gyrus, gyrus rectus
Time since AD diagnosis (months)
Sample Size
10
20
30
50
90
Legend
Average Value
Spline

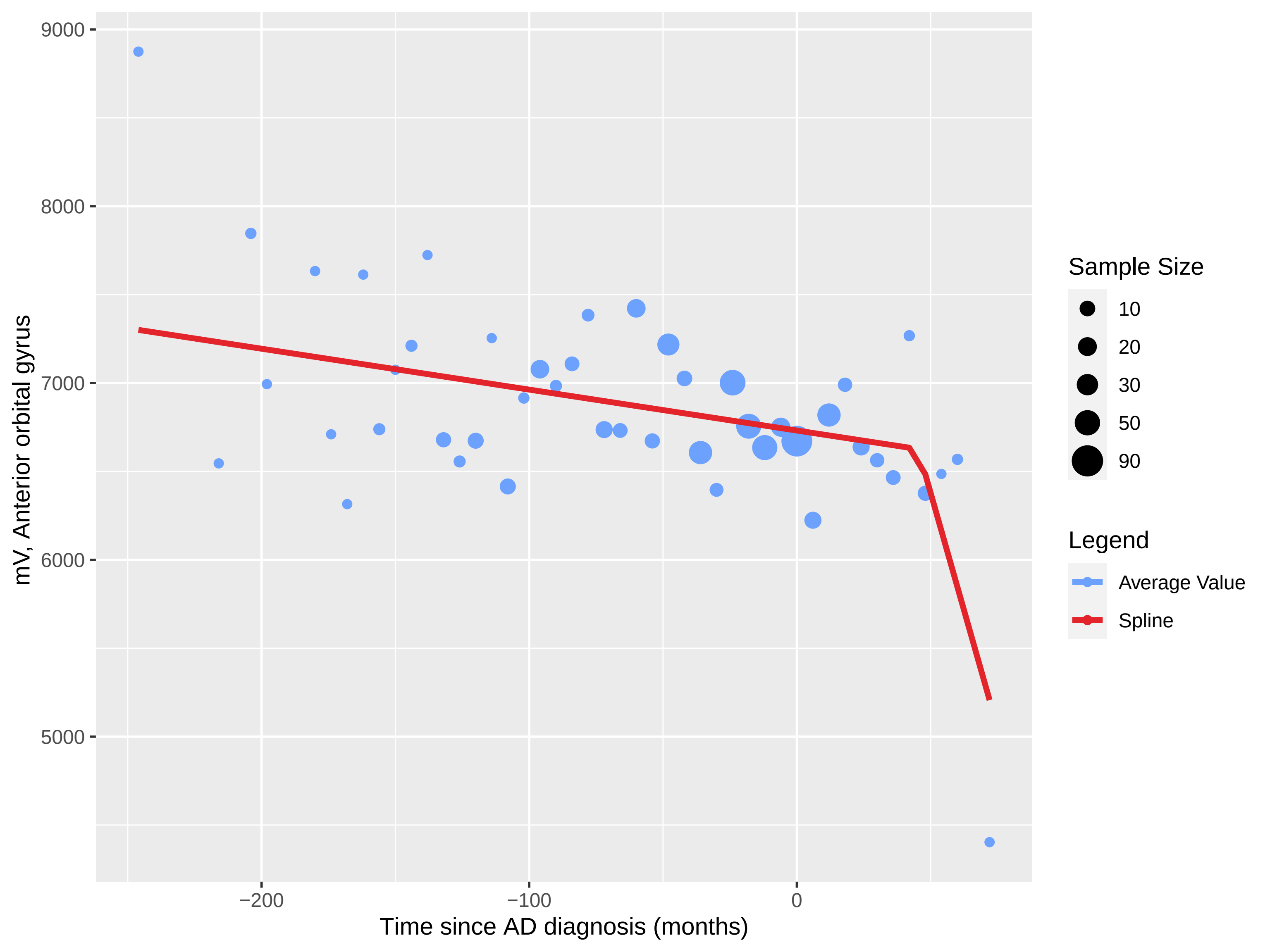

9000
8000
7000
6000
5000
−200
−100
0
mV, Anterior orbital gyrus
Time since AD diagnosis (months)
Sample Size
10
20
30
50
90
Legend
Average Value
Spline

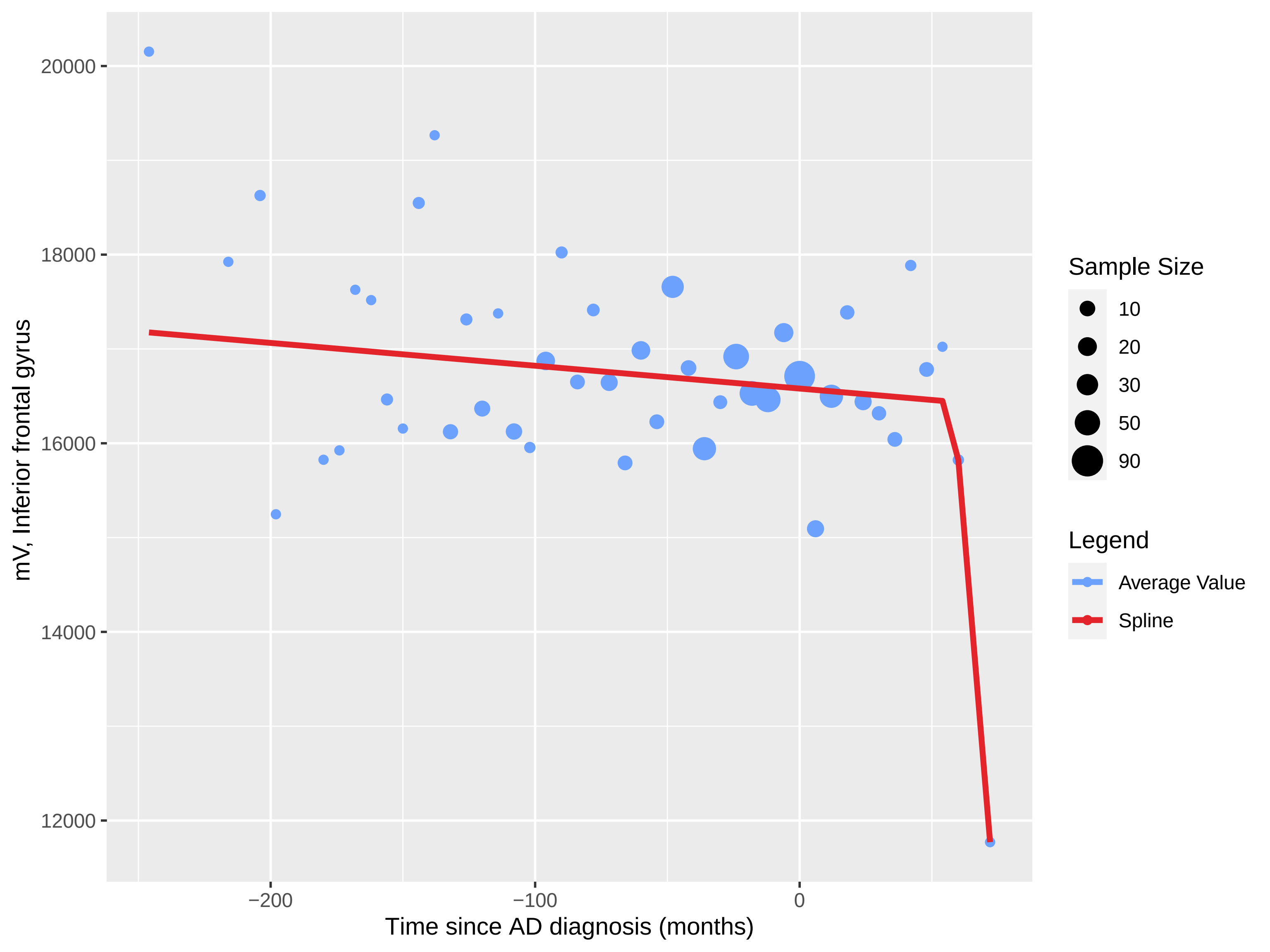

20000
18000
16000
14000
12000
−200
−100
0
Time since AD diagnosis (months)
mV, Inferior frontal gyrus
Sample Size
10
20
30
50
90
Legend
Average Value
Spline

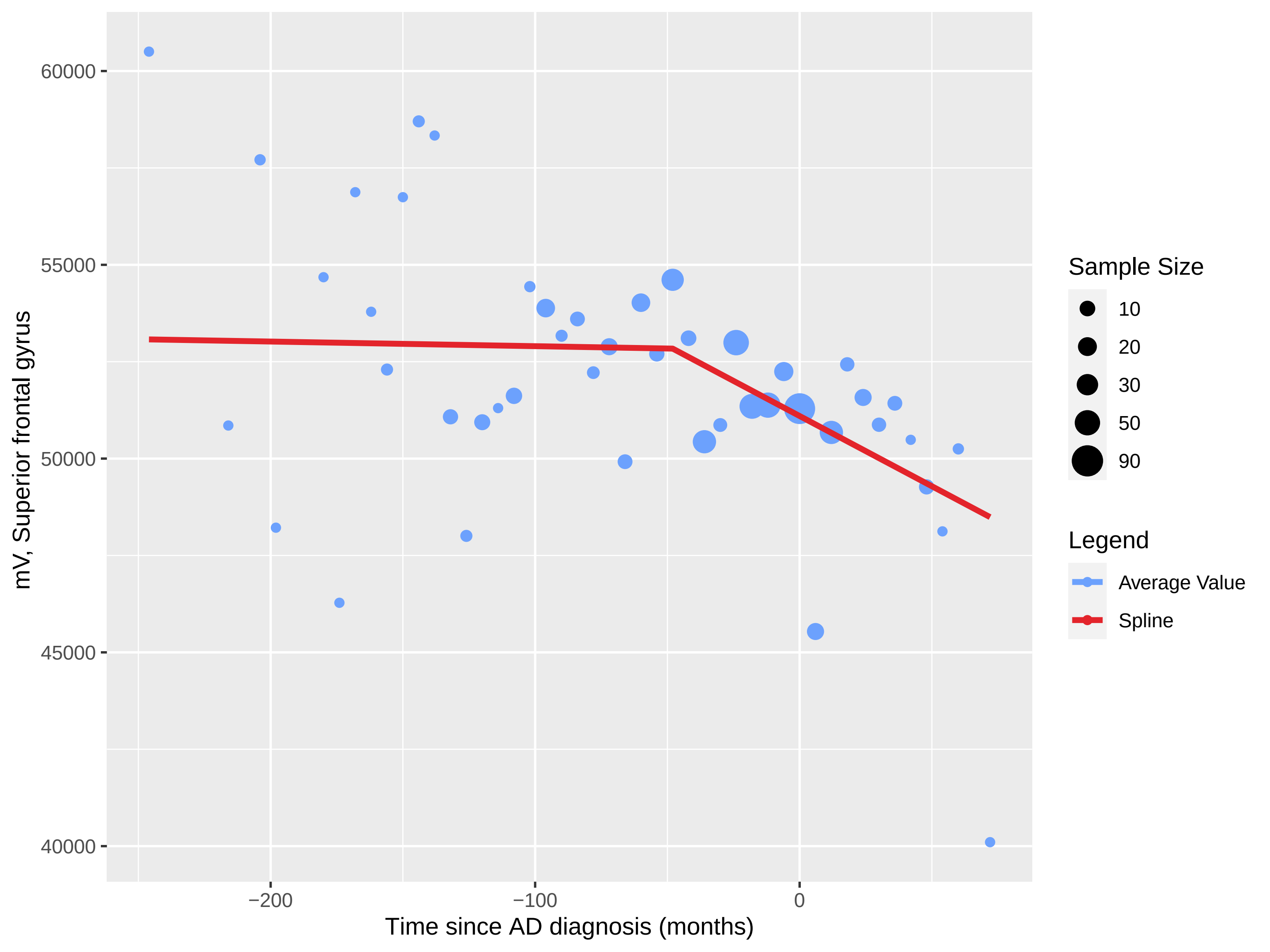

60000
55000
50000
45000
40000
mV, Superior frontal gyrus
−200
−100
0
Time since AD diagnosis (months)
Sample Size
10
20
30
50
90
Legend
Average Value
Spline

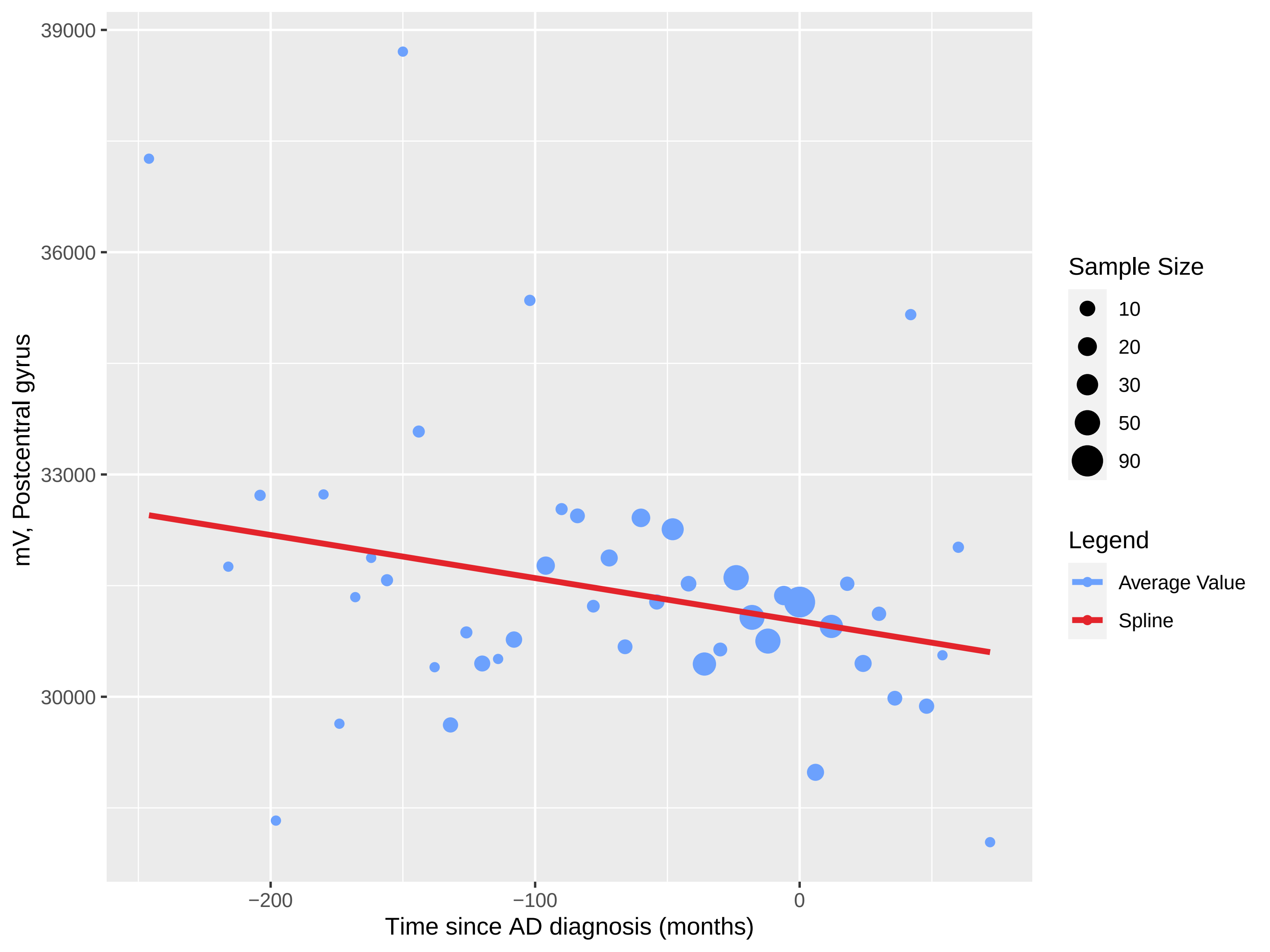

39000
36000
33000
30000
mV, Postcentral gyrus
−200
−100
0
Time since AD diagnosis (months)
Sample Size
10
20
30
50
90
Legend
Average Value
Spline

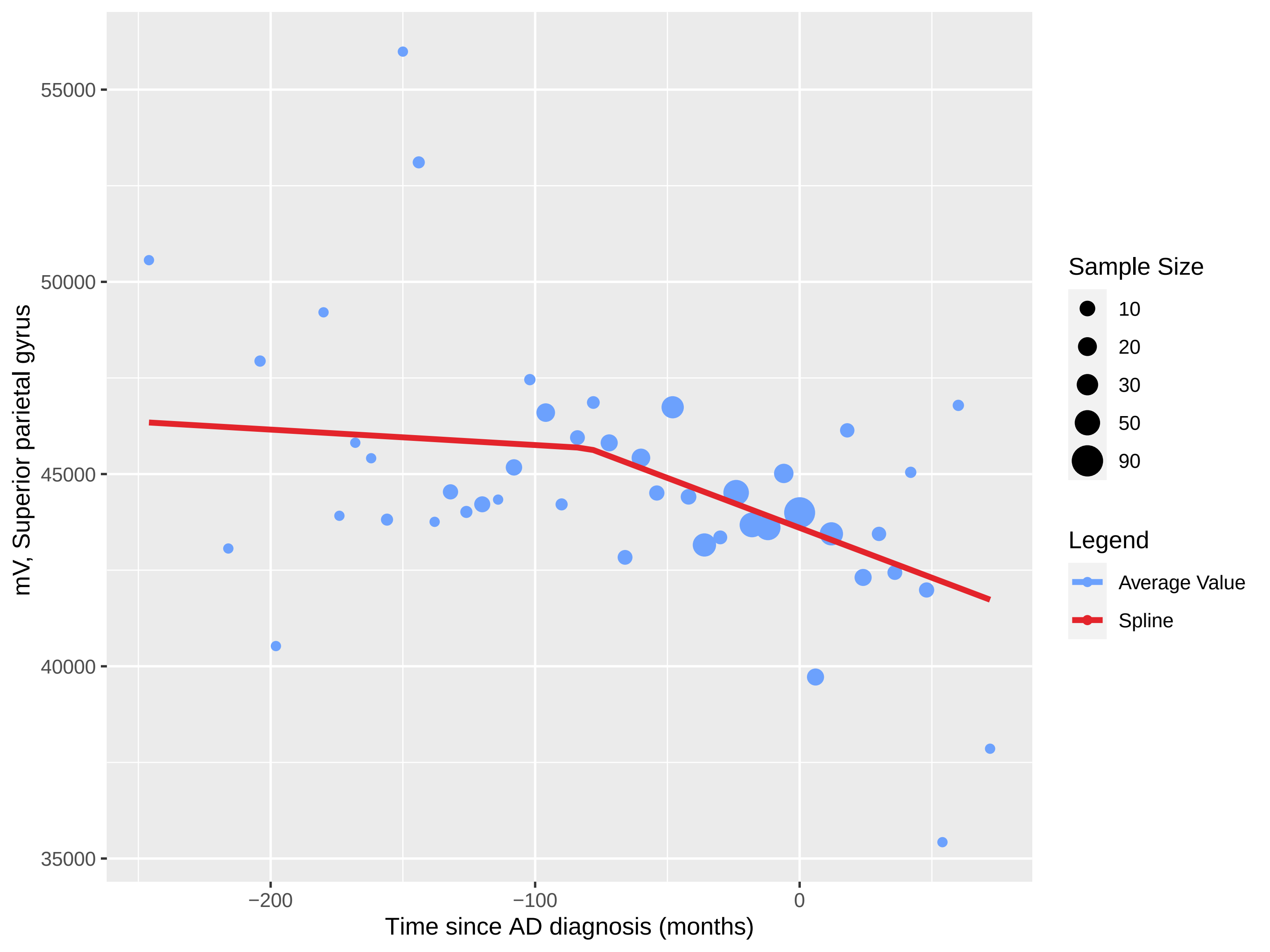

55000
50000
45000
40000
35000
mV, Superior parietal gyrus
−200
−100
0
Time since AD diagnosis (months)
Sample Size
10
20
30
50
90
Legend
Average Value
Spline

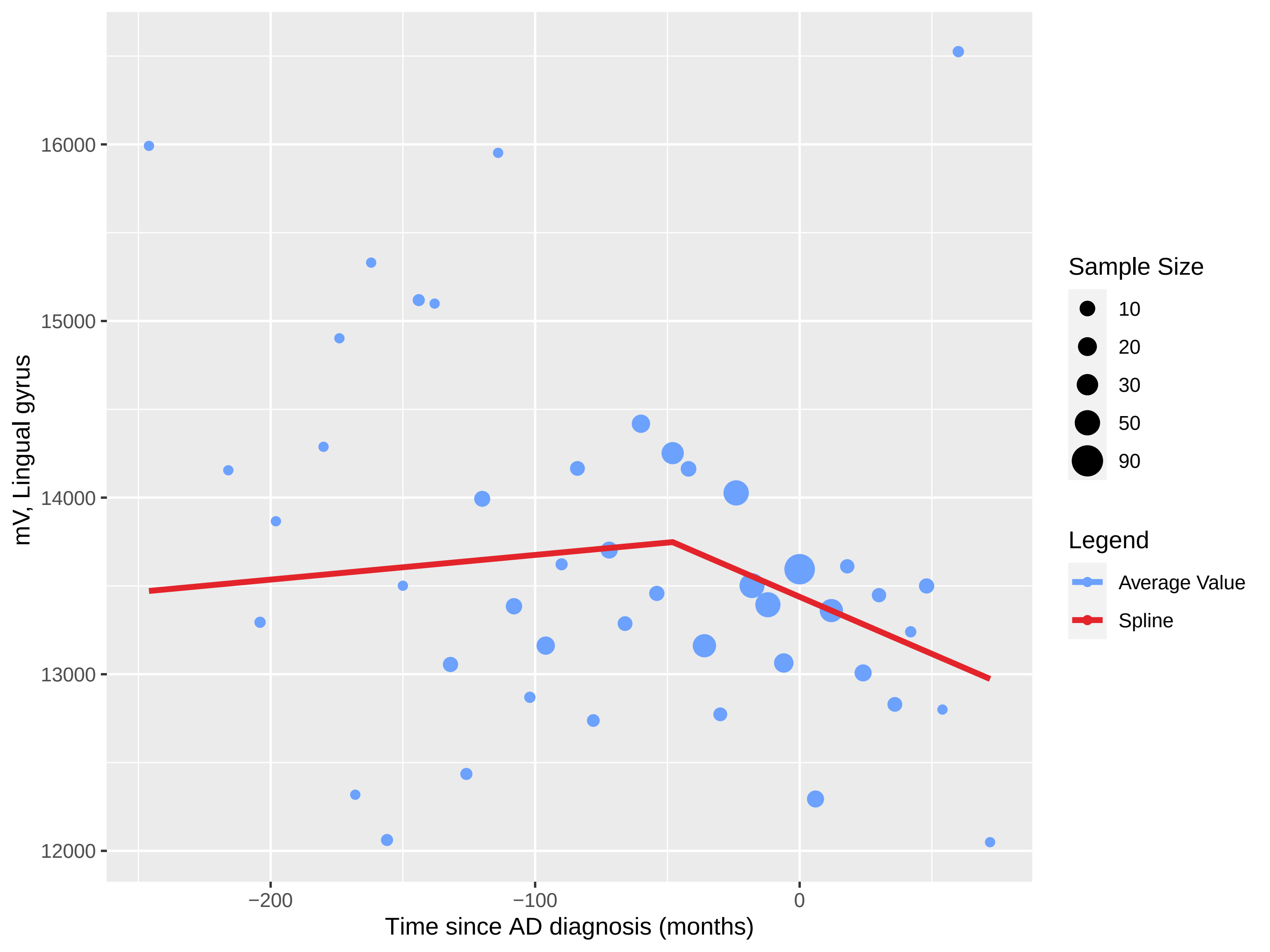

mV, Lingual gyrus
16000
15000
14000
13000
12000
−200
−100
0
Time since AD diagnosis (months)
Sample Size
10
20
30
50
90
Legend
Average Value
Spline

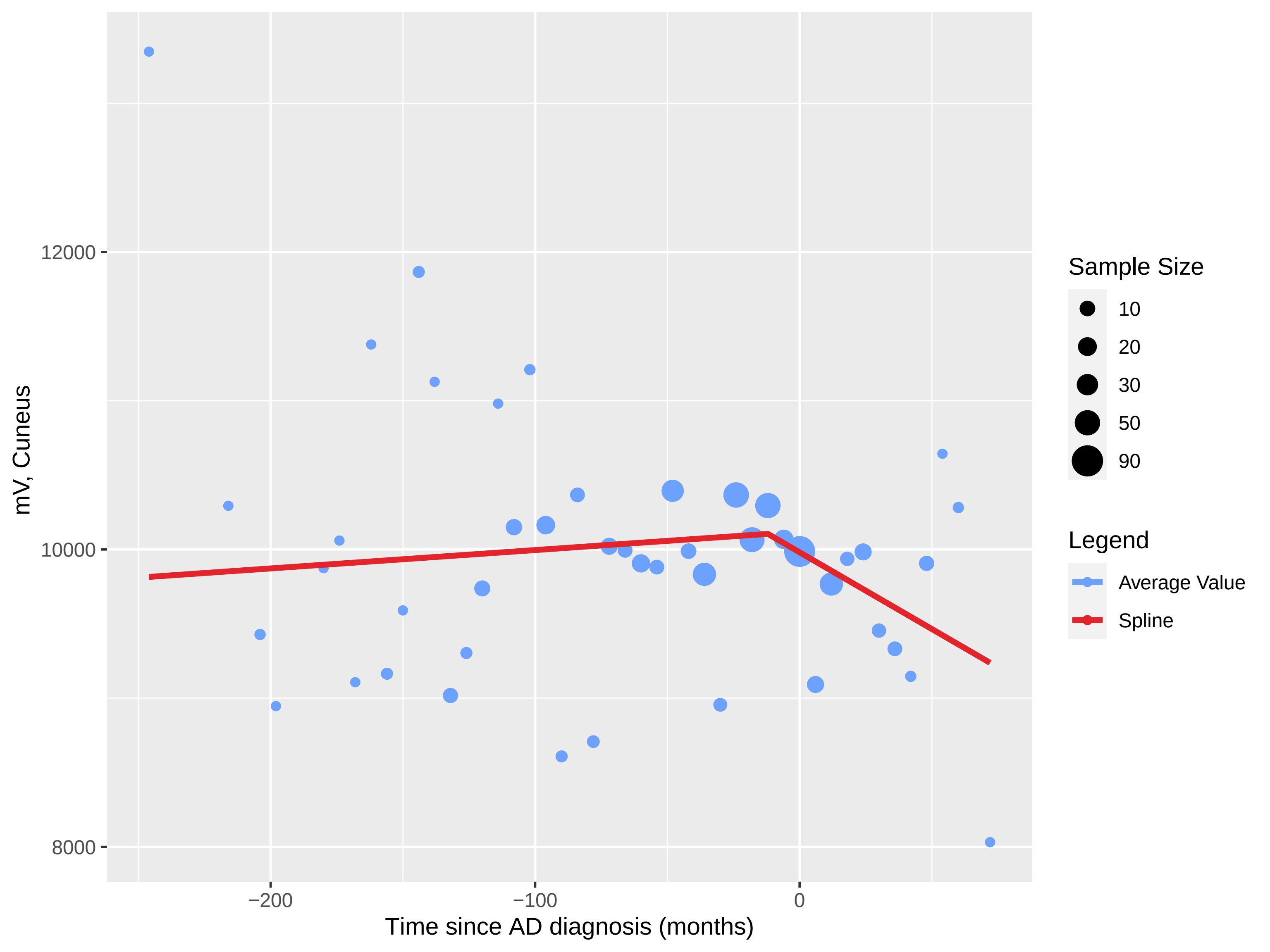

12000
10000
8000
−200
−100
0
mV, Cuneus
Time since AD diagnosis (months)
Sample Size
10
20
30
50
90
Legend
Average Value
Spline

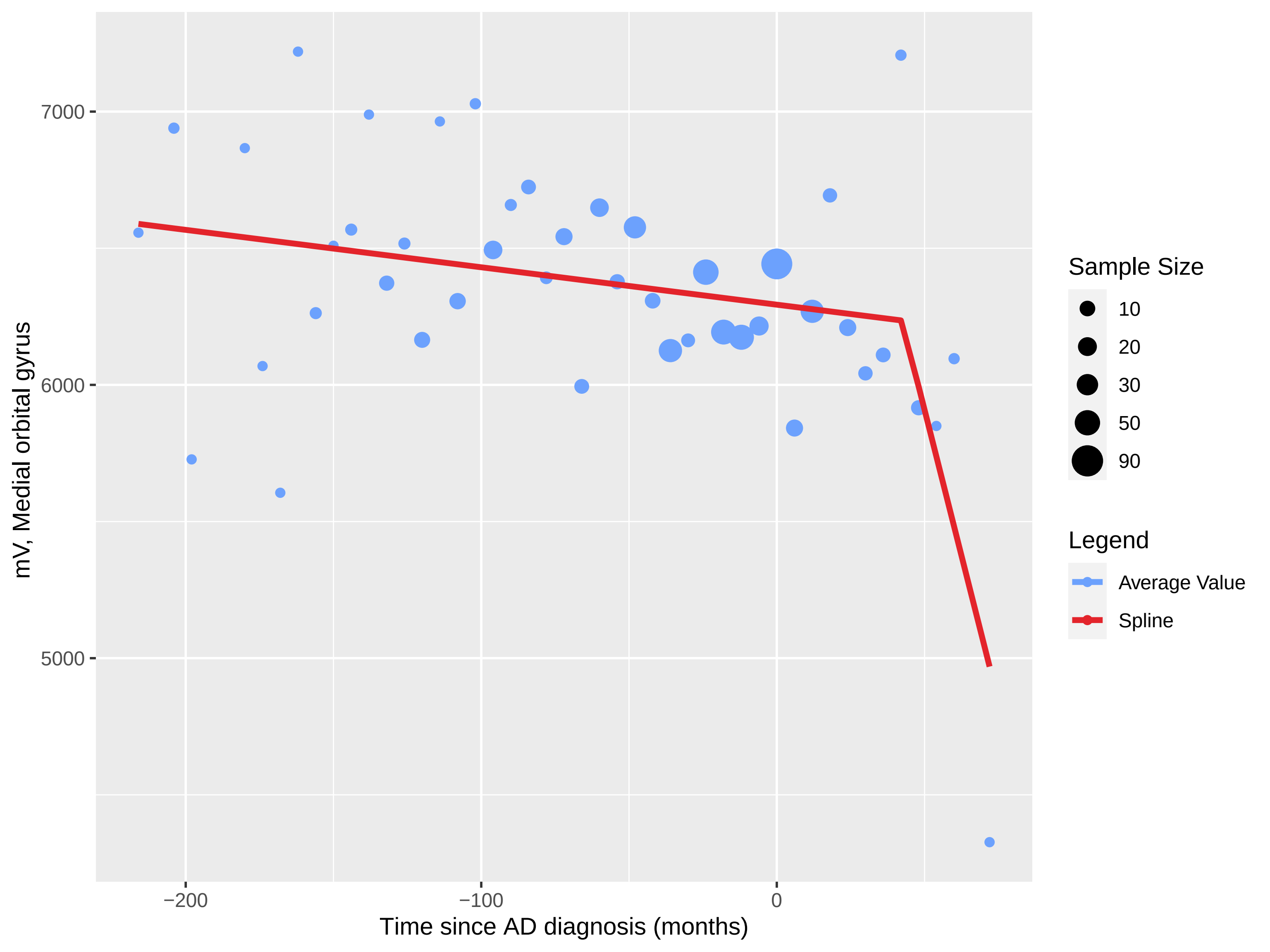

7000
6000
5000
mV, Medial orbital gyrus
−200
−100
0
Time since AD diagnosis (months)
Sample Size
10
20
30
50
90
Legend
Average Value
Spline

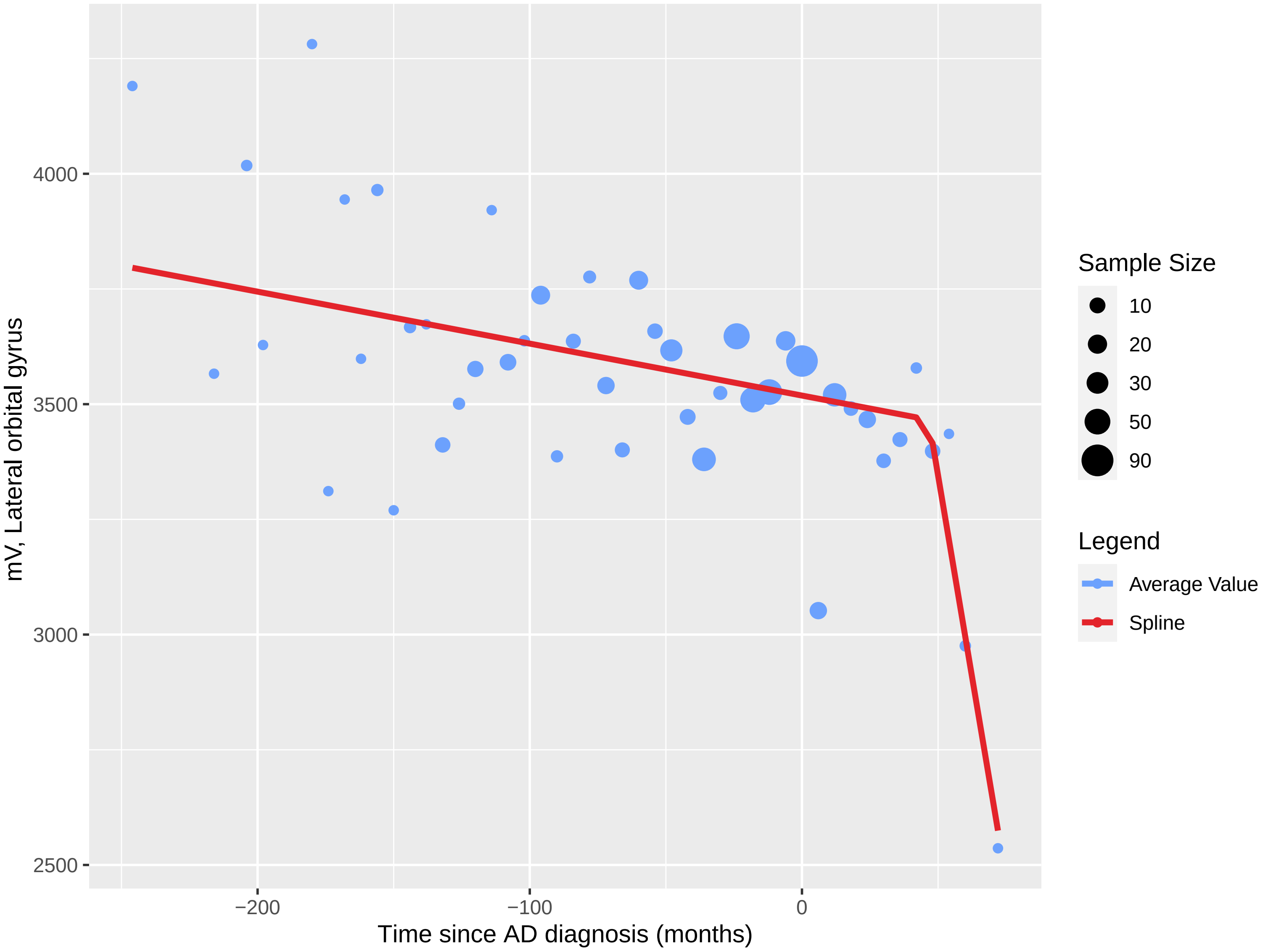

mV, Lateral orbital gyrus
4000
3500
3000
2500
−200
−100
0
Time since AD diagnosis (months)
Sample Size
10
20
30
50
90
Legend
Average Value
Spline

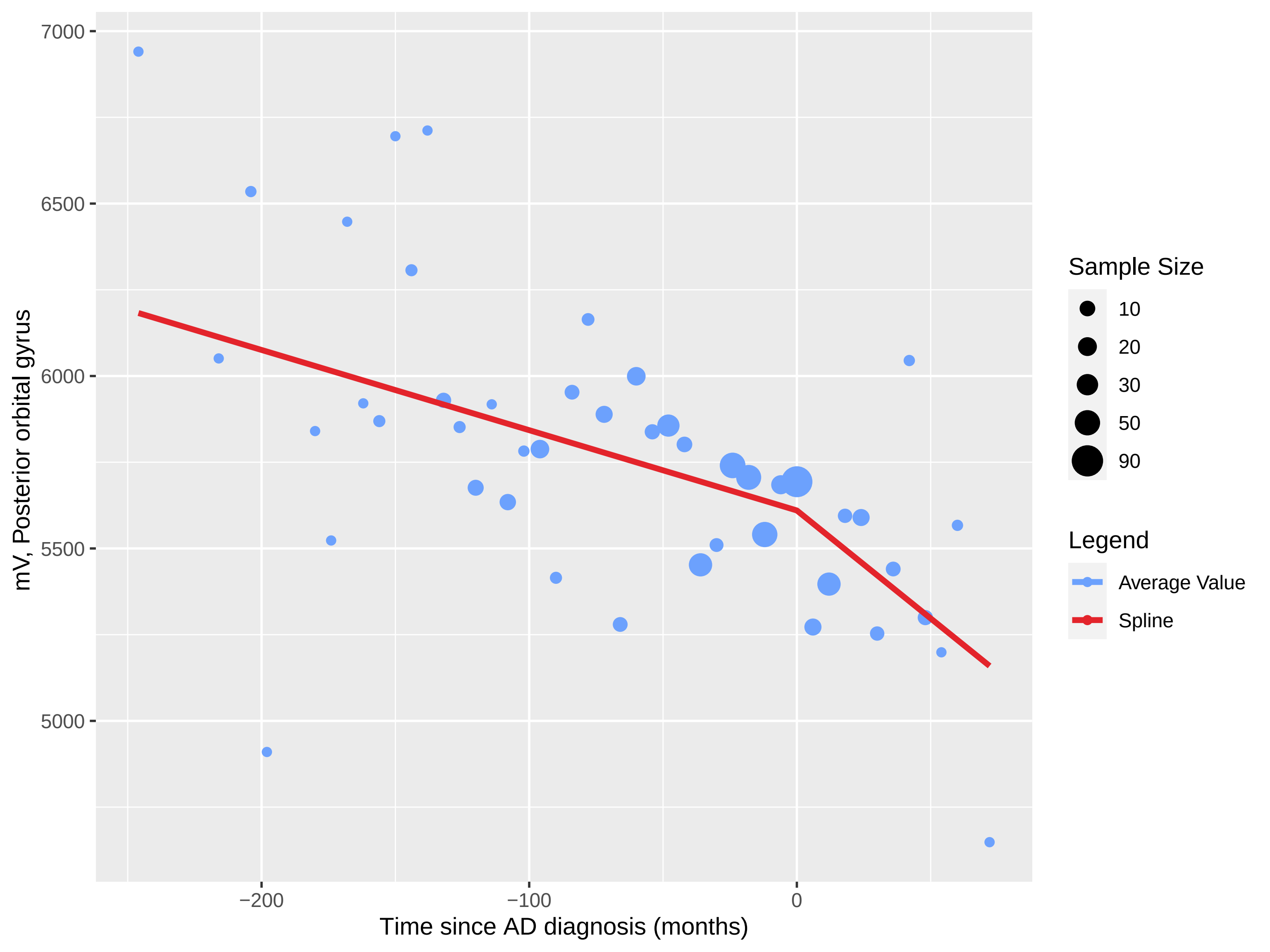

7000
6500
6000
5500
5000
−200
−100
0
mV, Posterior orbital gyrus
Time since AD diagnosis (months)
Sample Size
10
20
30
50
90
Legend
Average Value
Spline

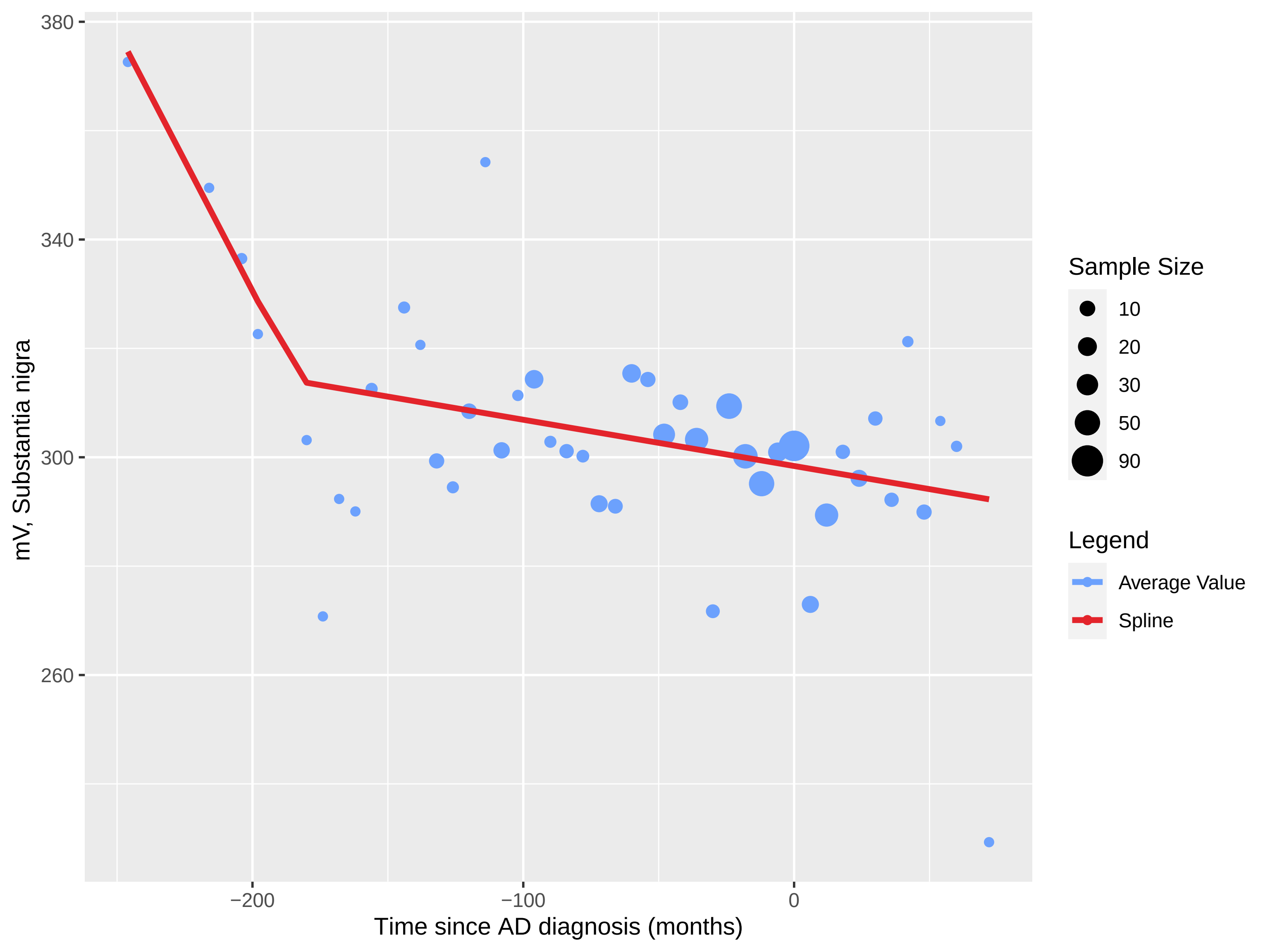

380
340
300
260
−200
−100
0
Time since AD diagnosis (months)
mV, Substantia nigra
Sample Size
10
20
30
50
90
Legend
Average Value
Spline

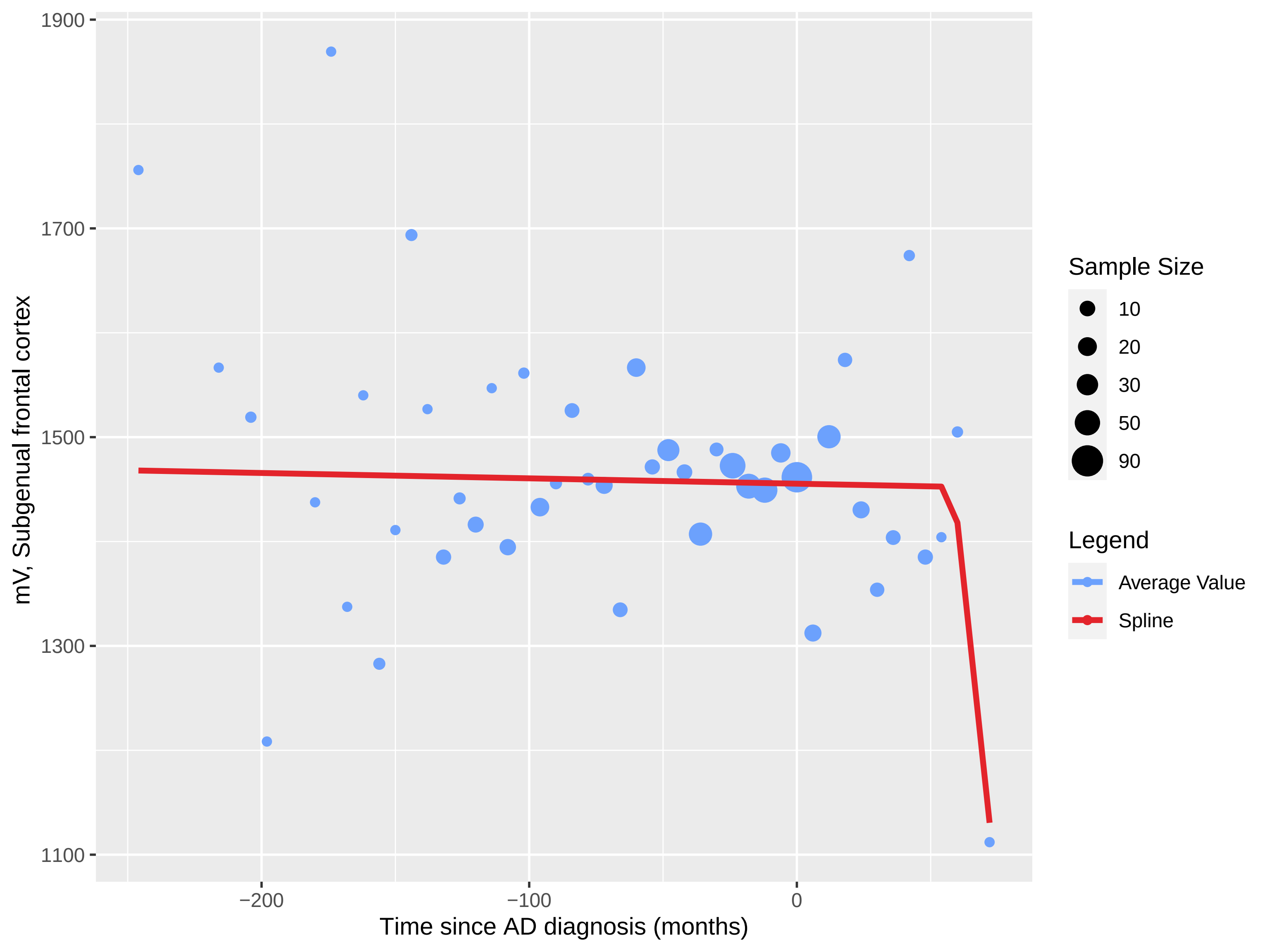

mV, Subgenual frontal cortex
1900
1700
1500
1300
1100
−200
−100
0
Time since AD diagnosis (months)
Sample Size
10
20
30
50
90
Legend
Average Value
Spline

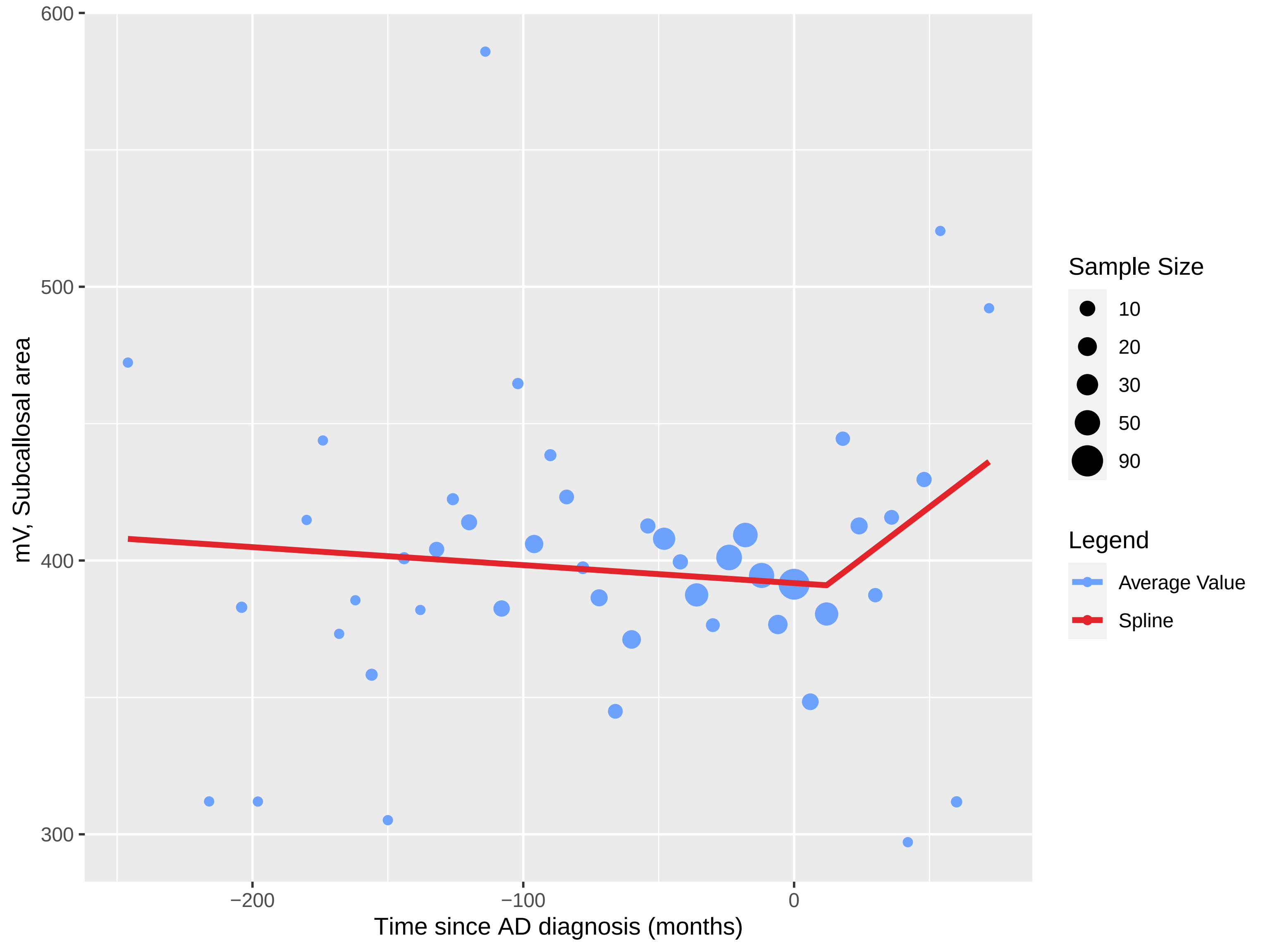
600
500
400
300
mV, Subcallosal area
−200
−100
0
Time since AD diagnosis (months)
Sample Size
10
20
30
50
90
Legend
Average Value
Spline

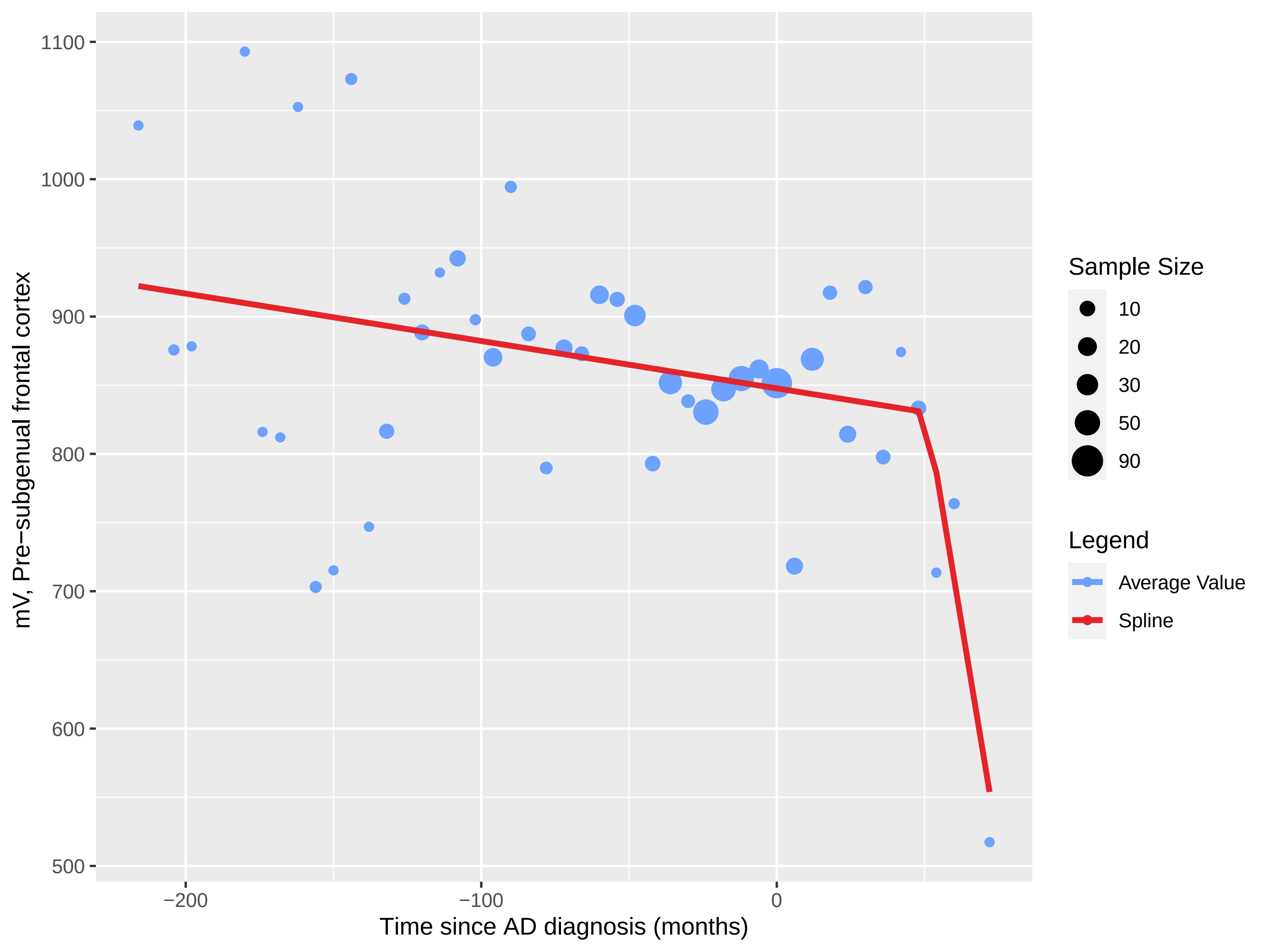

1100
1000
900
800
700
600
500
−200
−100
0
Time since AD diagnosis (months)
mV, Pre−subgenual frontal cortex
Sample Size
10
20
30
50
90
Legend
Average Value
Spline

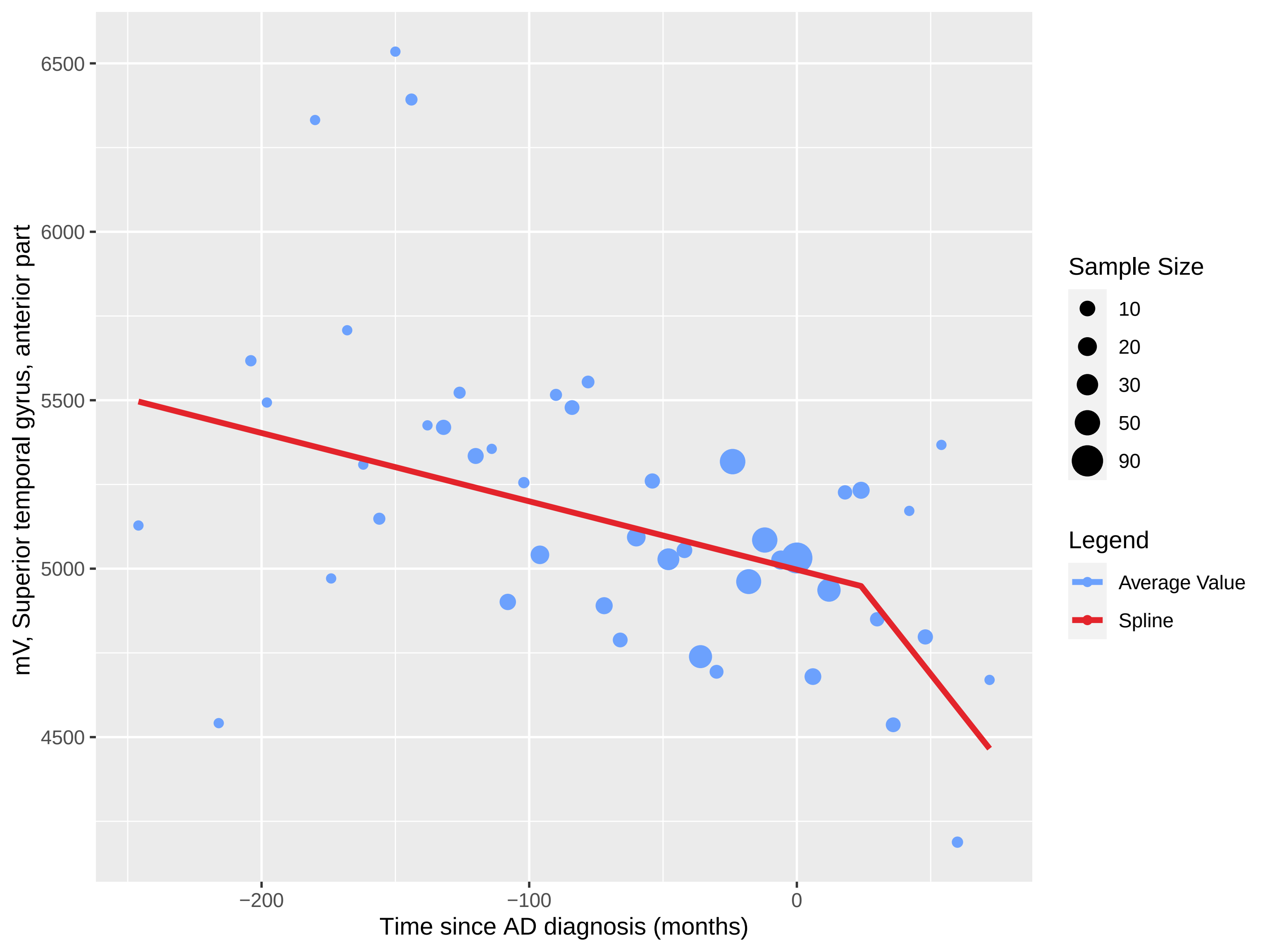

6500
6000
5500
5000
4500
mV, Superior temporal gyrus, anterior part
−200
−100
0
Time since AD diagnosis (months)
Sample Size
10
20
30
50
90
Legend
Average Value
Spline

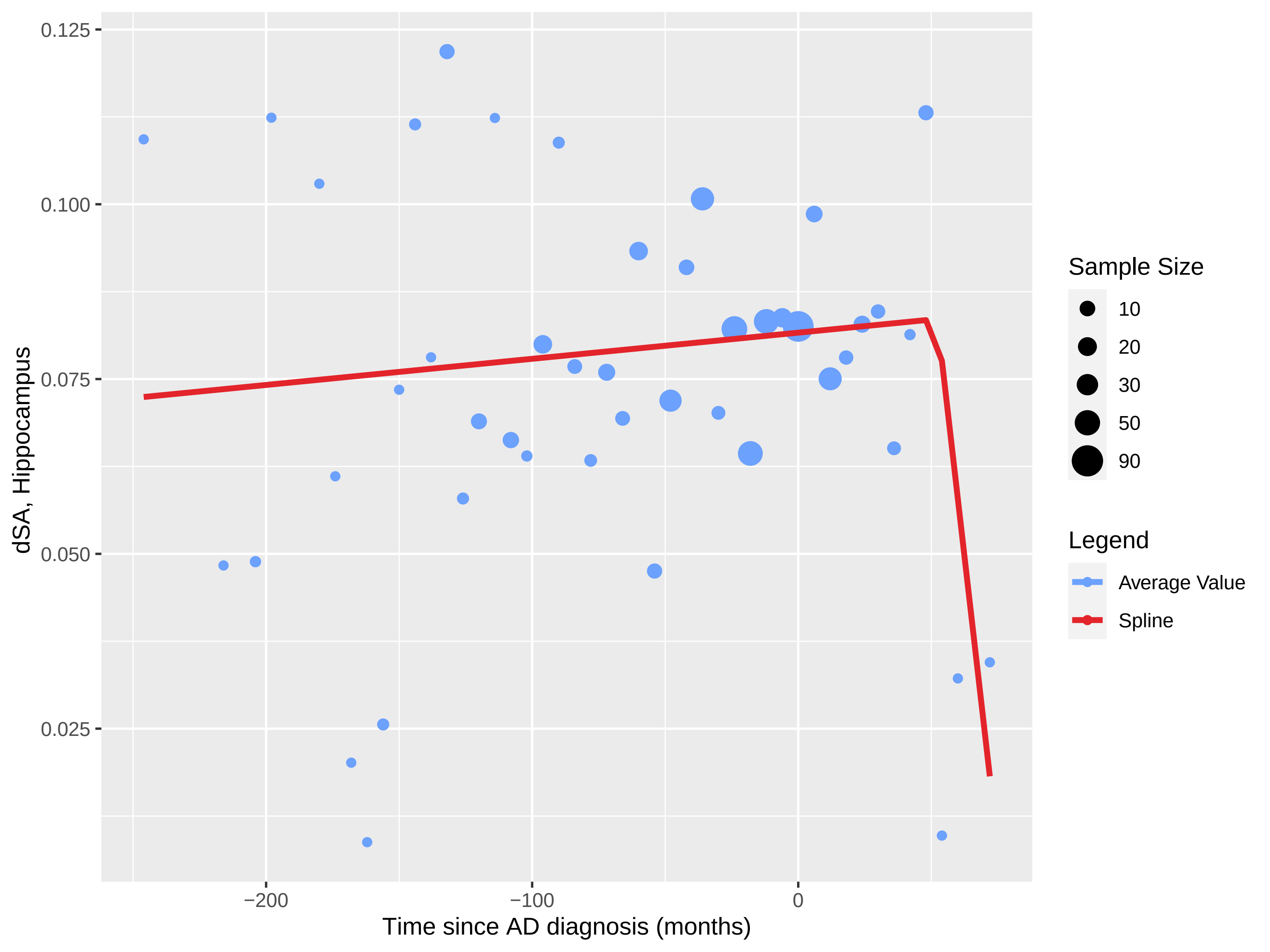
0.125
0.100
0.075
0.050
0.025
dSA, Hippocampus
−200
−100
0
Time since AD diagnosis (months)
Sample Size
10
20
30
50
90
Legend
Average Value
Spline

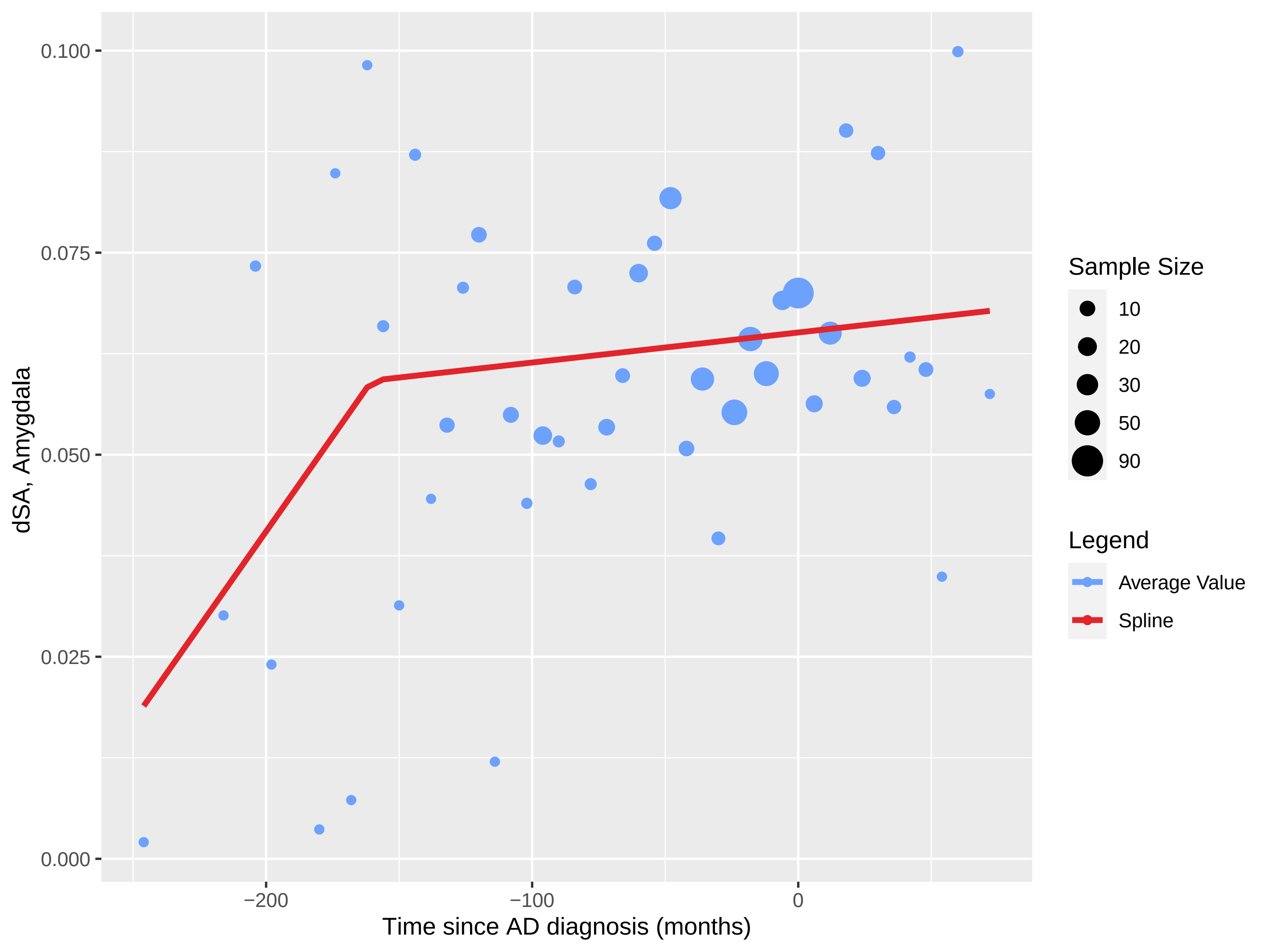

0.100
0.075
0.050
0.025
0.000
dSA, Amygdala
−200
−100
0
Time since AD diagnosis (months)
Sample Size
10
20
30
50
90
Legend
Average Value
Spline

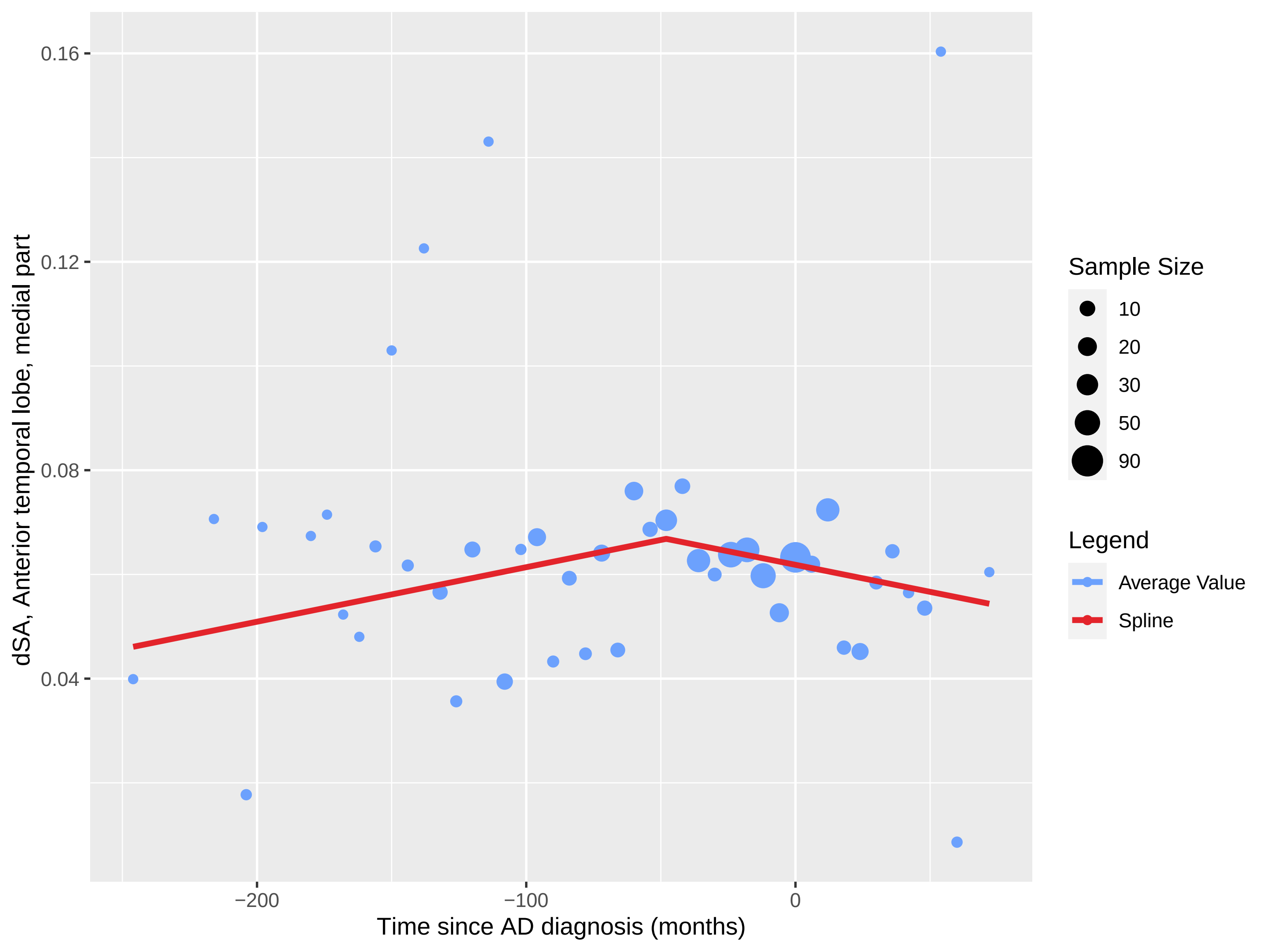

dSA, Anterior temporal lobe, medial part
0.16
0.12
0.08
0.04
−200
−100
0
Time since AD diagnosis (months)
Sample Size
10
20
30
50
90
Legend
Average Value
Spline

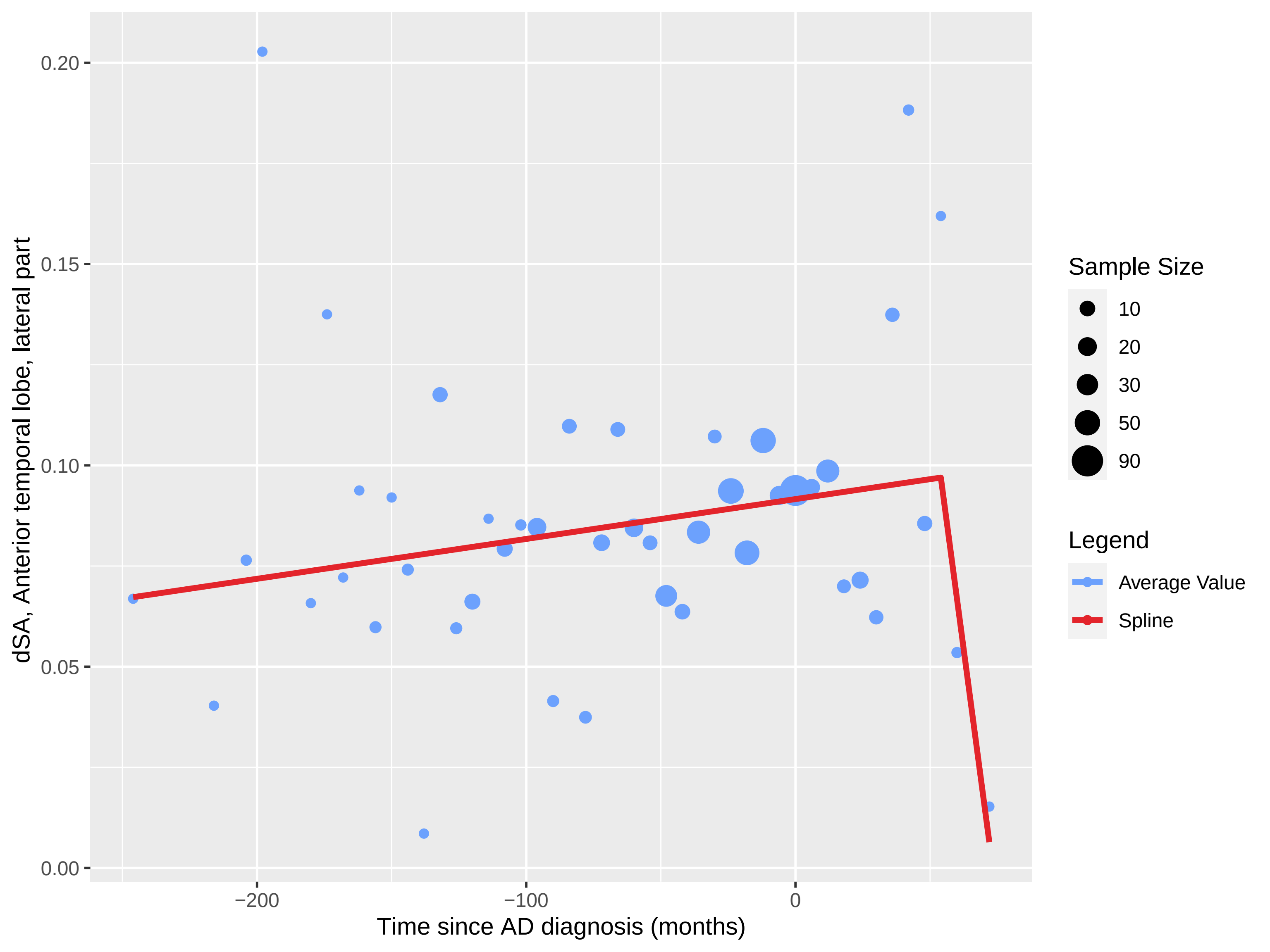

dSA, Anterior temporal lobe, lateral part
0.20
0.15
0.10
0.05
0.00
−200
−100
0
Time since AD diagnosis (months)
Sample Size
10
20
30
50
90
Legend
Average Value
Spline

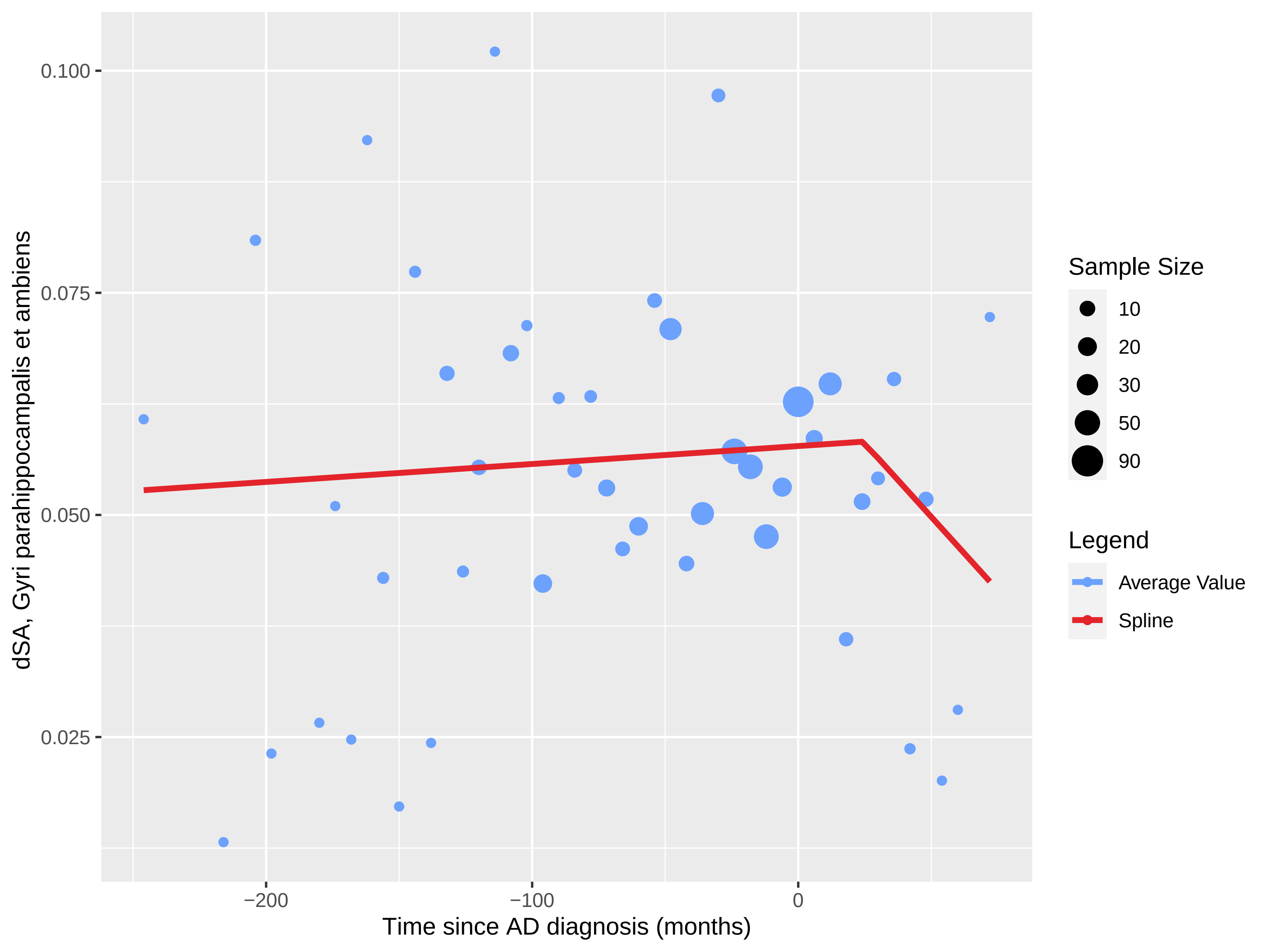

0.100
0.075
0.050
0.025
−200
−100
0
Time since AD diagnosis (months)
dSA, Gyri parahippocampalis et ambiens
Sample Size
10
20
30
50
90
Legend
Average Value
Spline

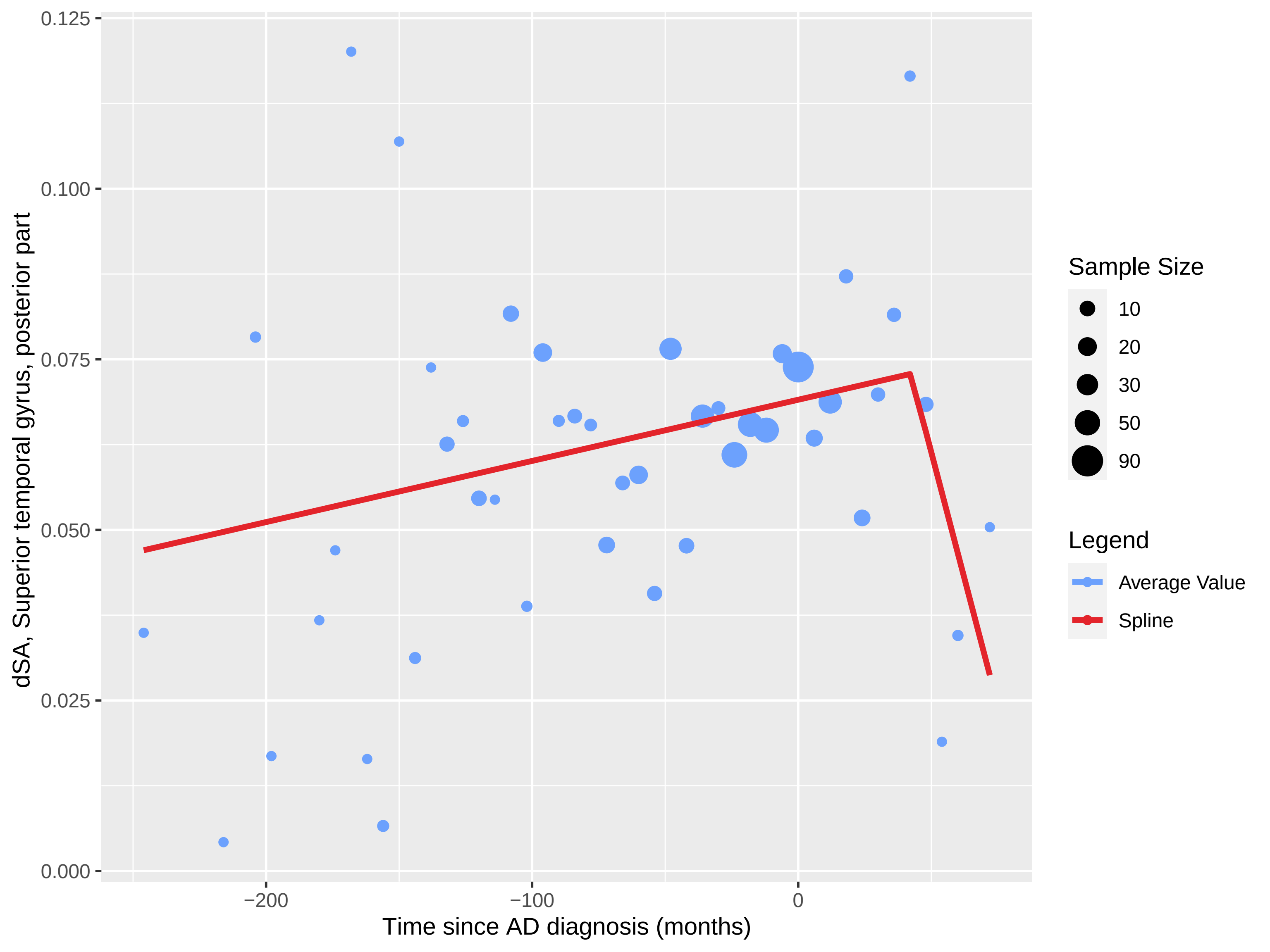
dSA, Superior temporal gyrus, posterior part
0.125
0.100
0.075
0.050
0.025
0.000
−200
−100
0
Time since AD diagnosis (months)
Sample Size
10
20
30
50
90
Legend
Average Value
Spline

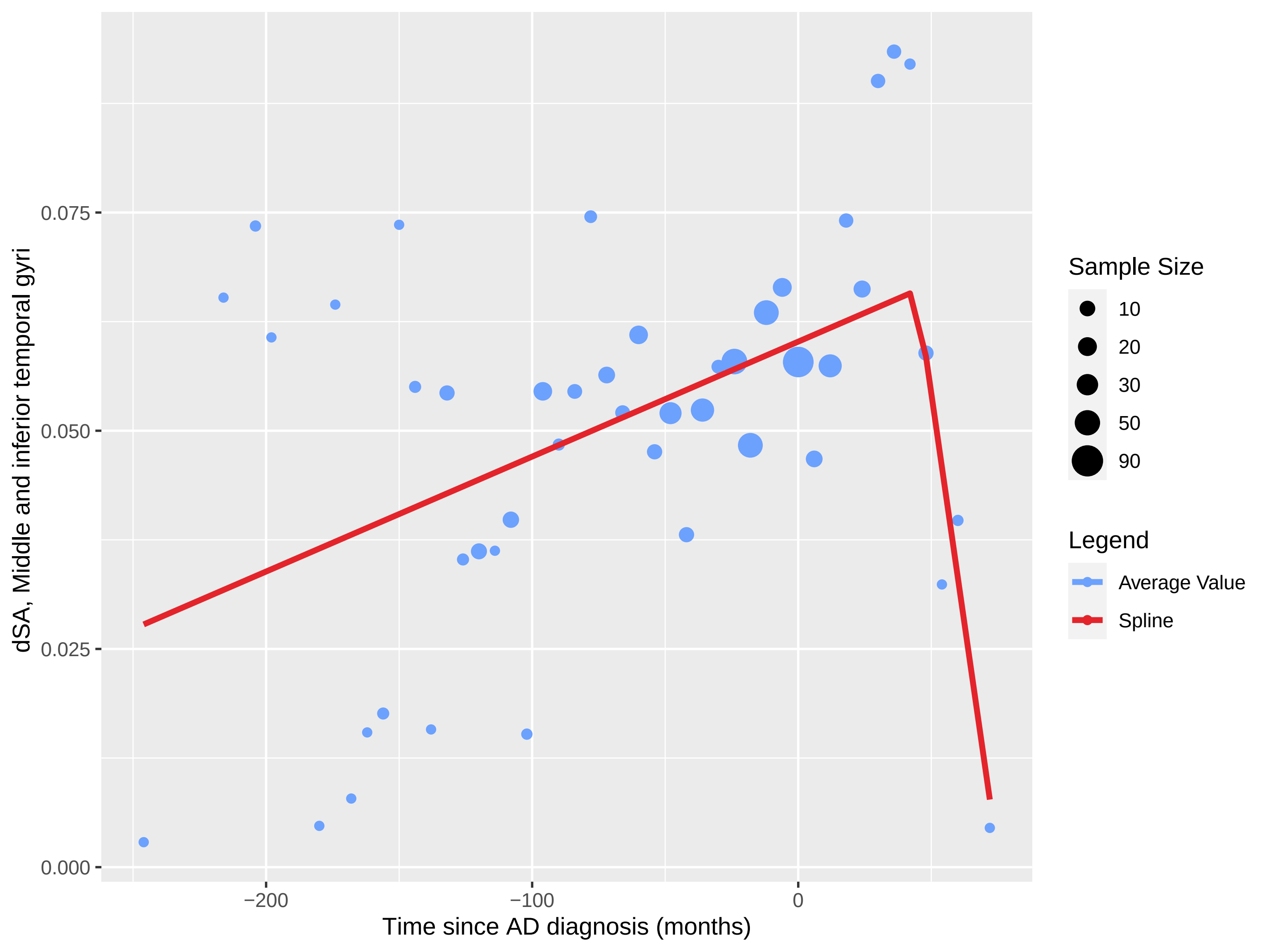

dSA, Middle and inferior temporal gyri
0.075
0.050
0.025
0.000
−200
−100
0
Time since AD diagnosis (months)
Sample Size
10
20
30
50
90
Legend
Average Value
Spline

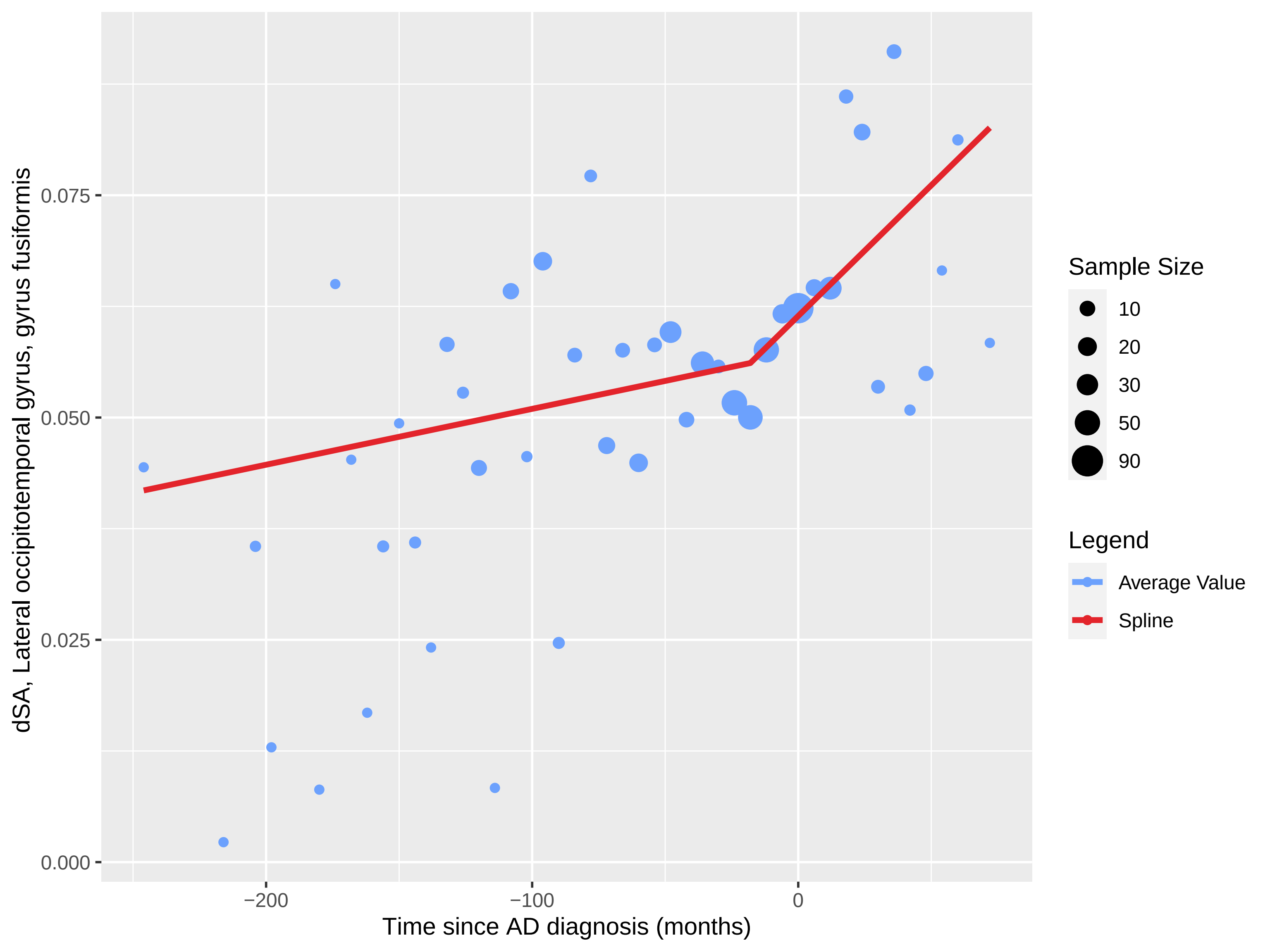

dSA, Lateral occipitotemporal gyrus, gyrus fusiformis
0.075
0.050
0.025
0.000
−200
−100
0
Time since AD diagnosis (months)
Sample Size
10
20
30
50
90
Legend
Average Value
Spline

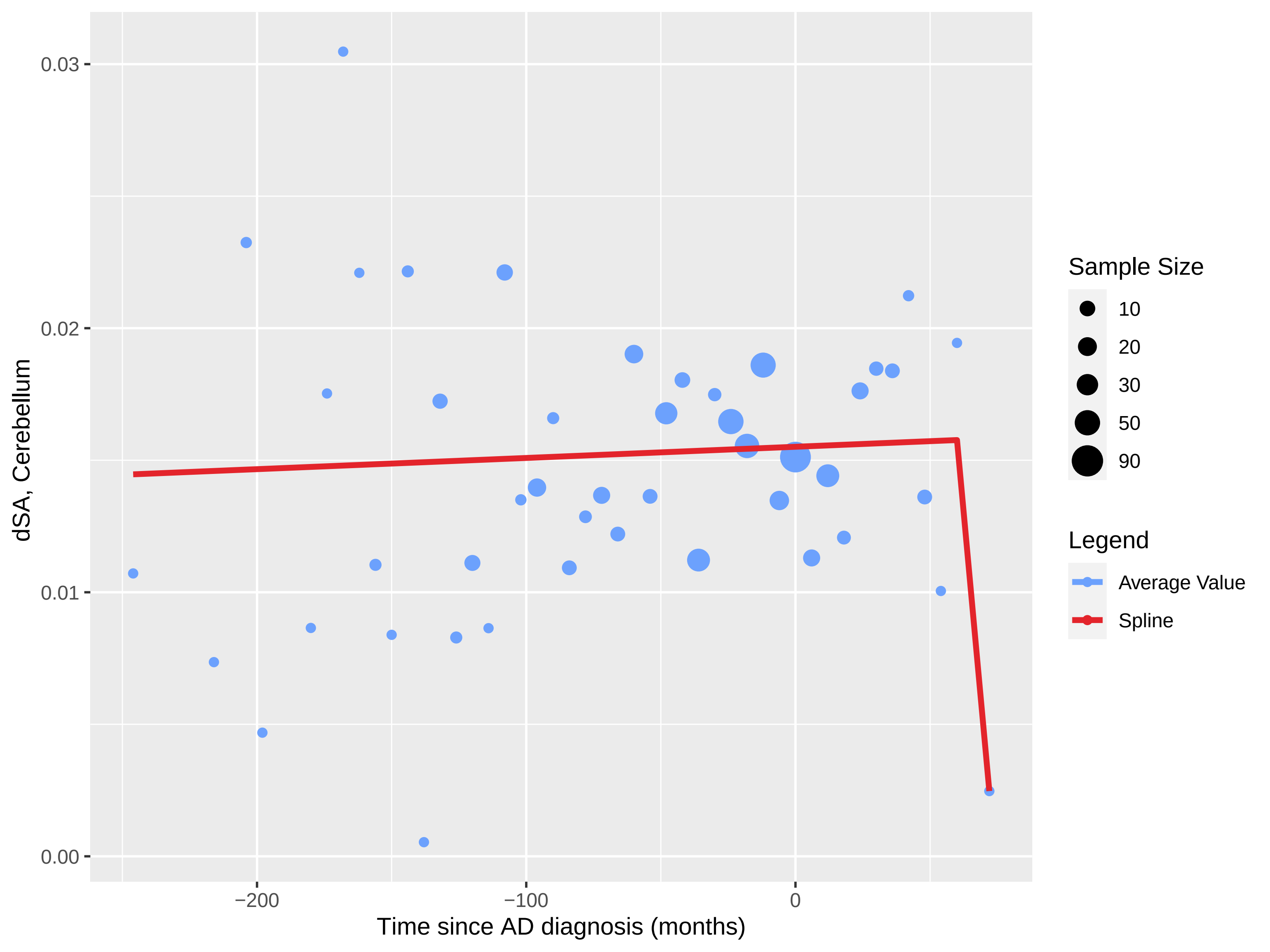

0.03
0.02
0.01
0.00
dSA, Cerebellum
−200
−100
0
Time since AD diagnosis (months)
Sample Size
10
20
30
50
90
Legend
Average Value
Spline

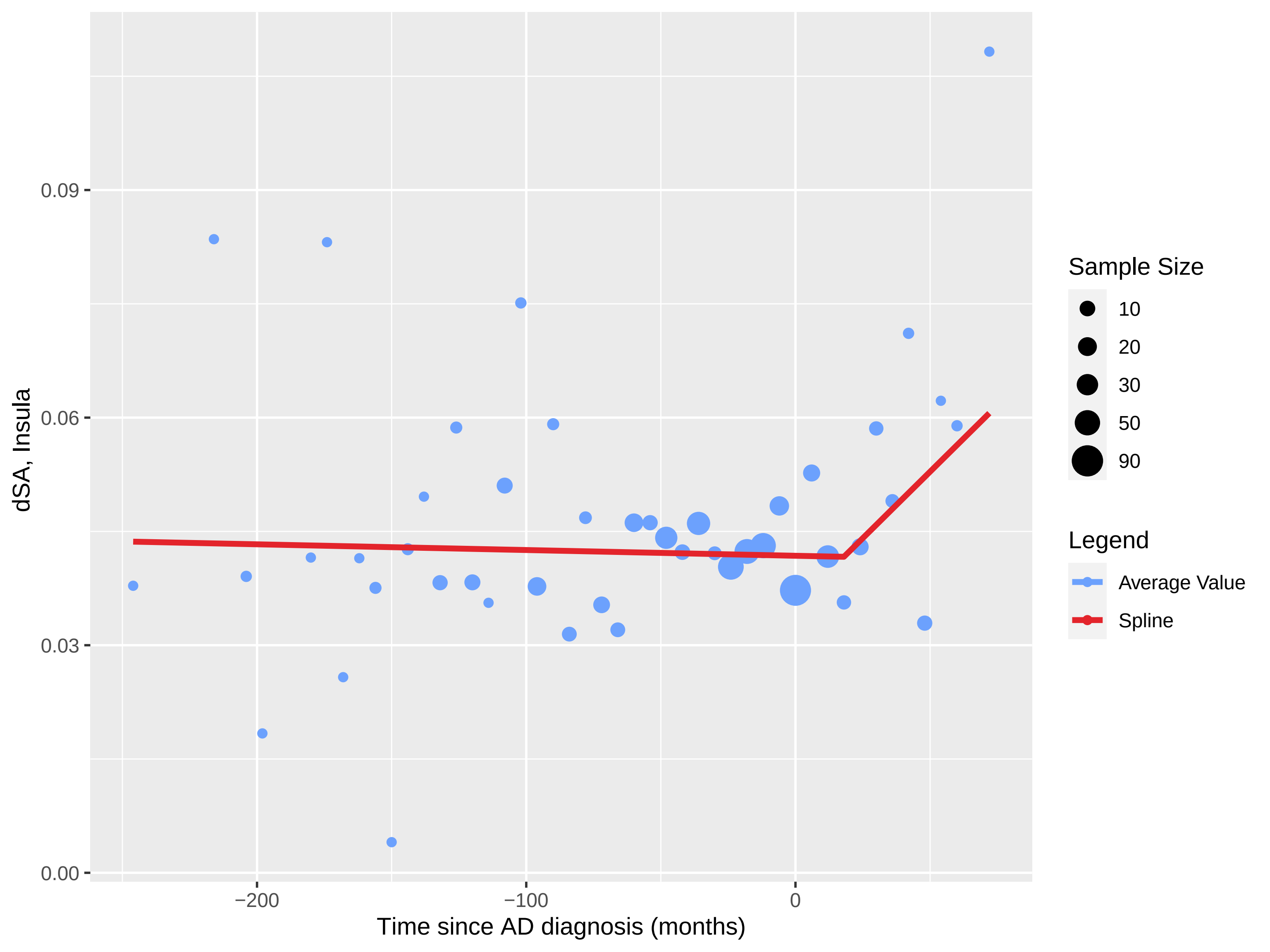

0.09
0.06
0.03
0.00
−200
−100
0
dSA, Insula
Time since AD diagnosis (months)
Sample Size
10
20
30
50
90
Legend
Average Value
Spline

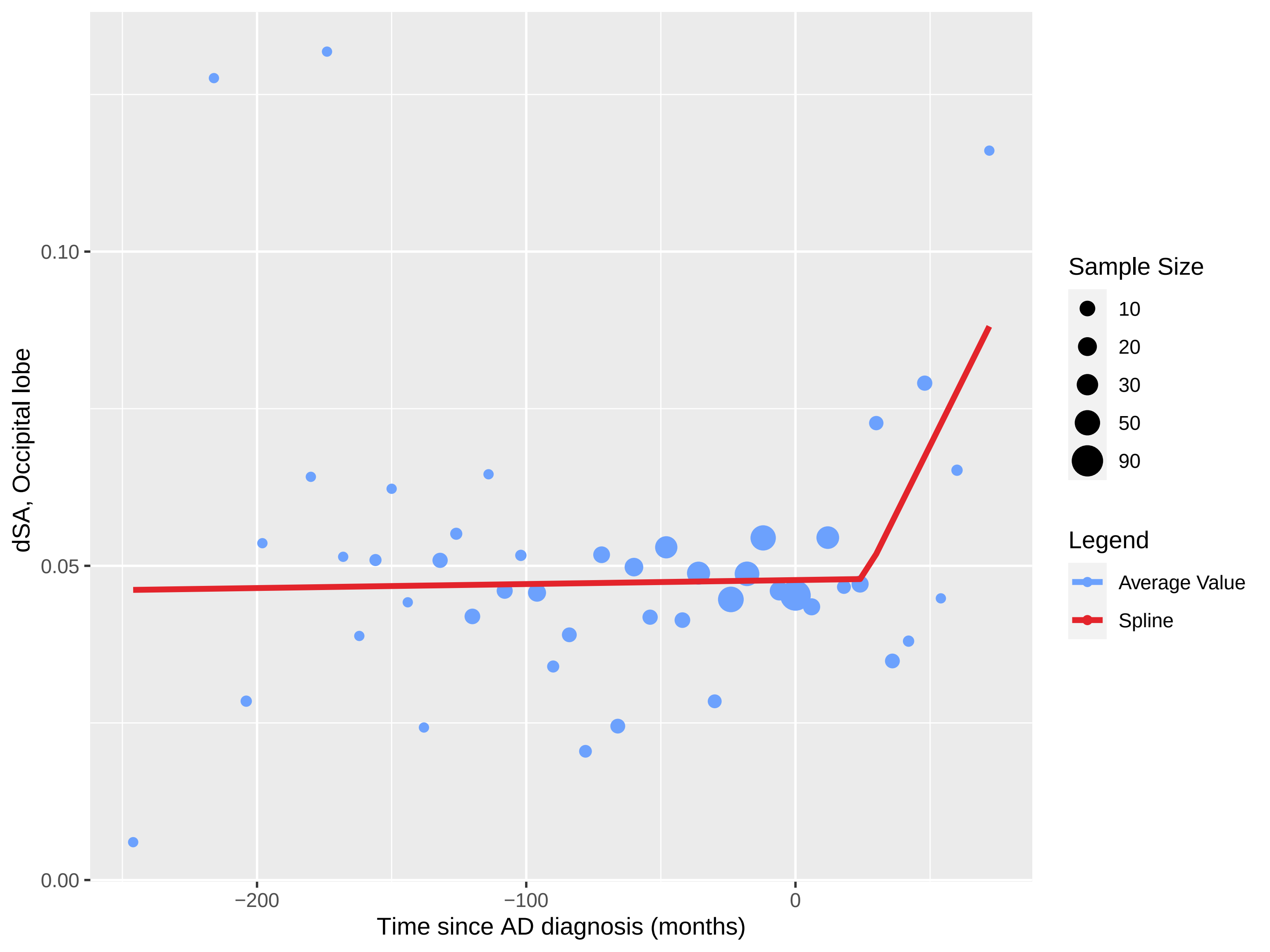

dSA, Occipital lobe
0.10
0.05
0.00
−200
−100
0
Time since AD diagnosis (months)
Sample Size
10
20
30
50
90
Legend
Average Value
Spline

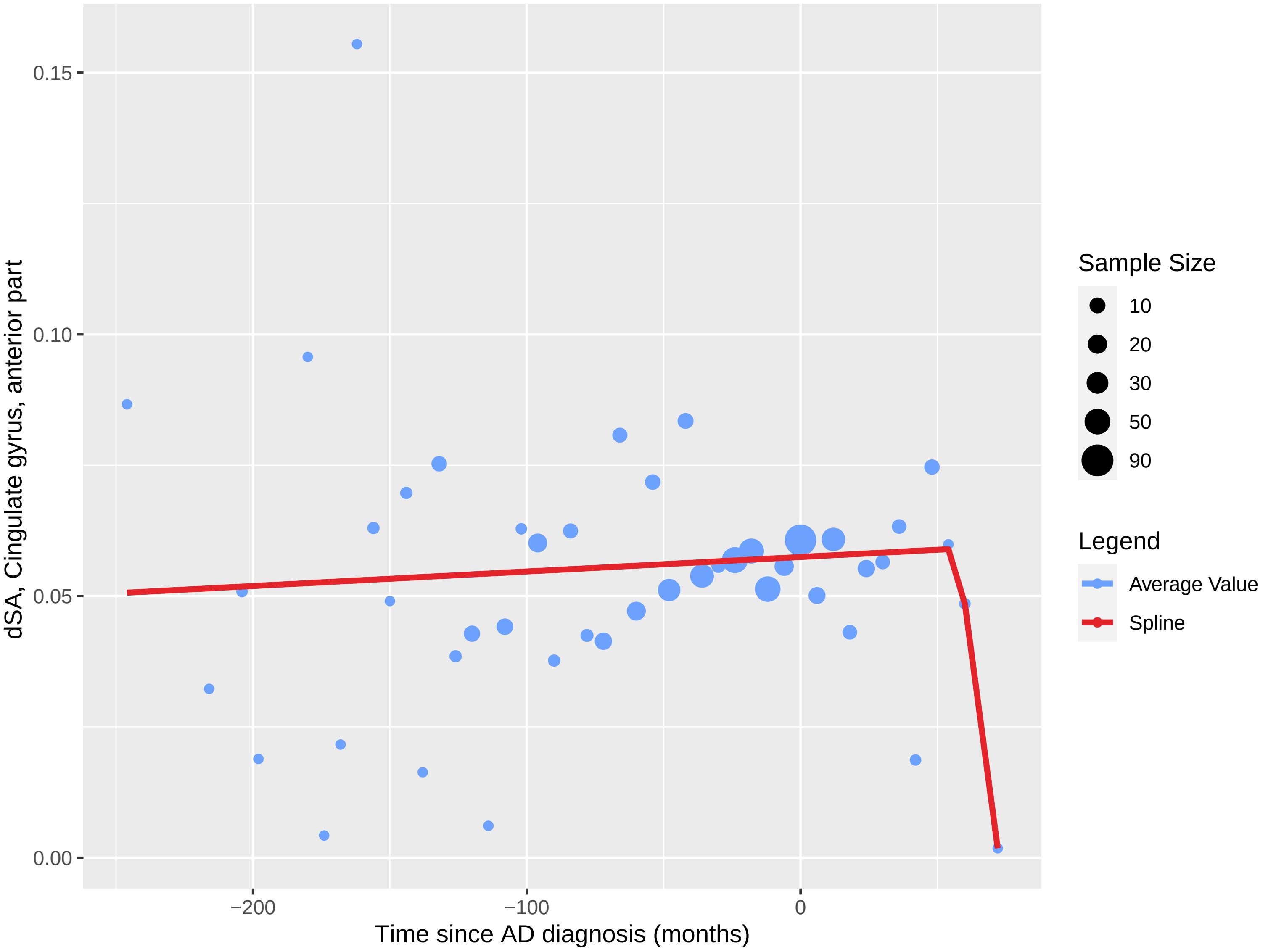

0.15
0.10
0.05
0.00
−200
−100
0
Time since AD diagnosis (months)
dSA, Cingulate gyrus, anterior part
Sample Size
10
20
30
50
90
Legend
Average Value
Spline

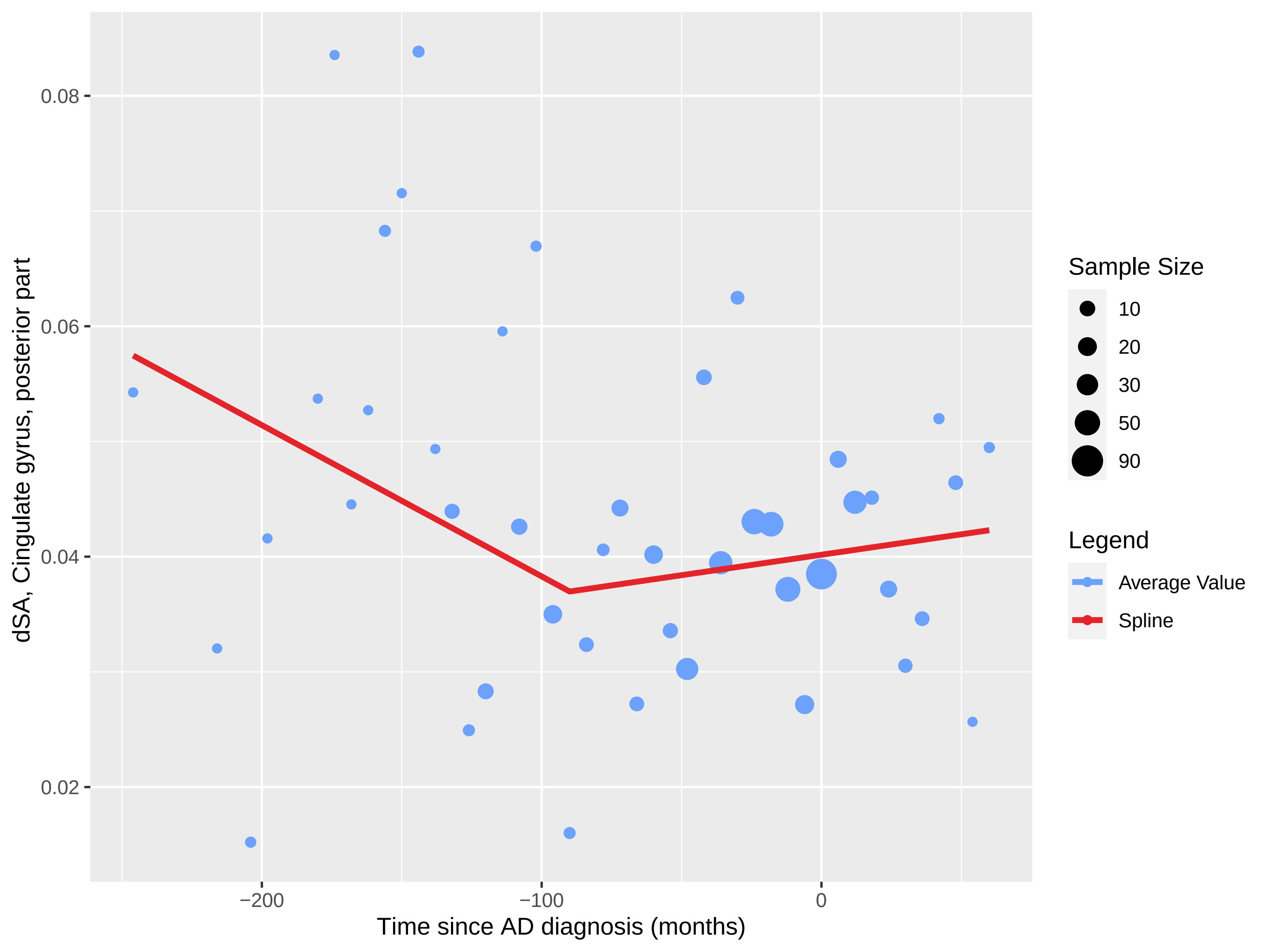

dSA, Cingulate gyrus, posterior part
0.08
0.06
0.04
0.02
−200
−100
0
Time since AD diagnosis (months)
Sample Size
10
20
30
50
90
Legend
Average Value
Spline

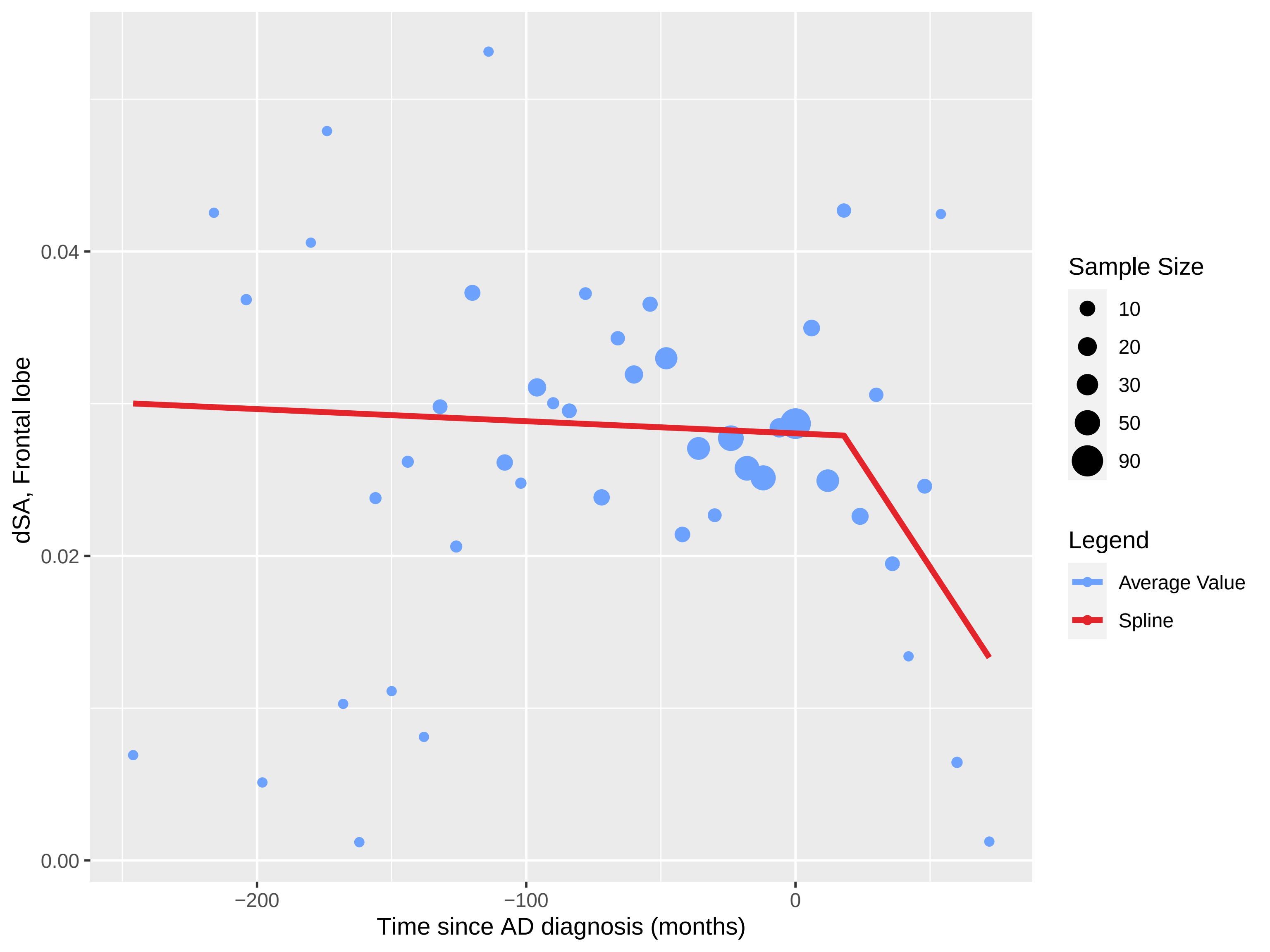

dSA, Frontal lobe
0.04
0.02
0.00
−200
−100
0
Time since AD diagnosis (months)
Sample Size
10
20
30
50
90
Legend
Average Value
Spline

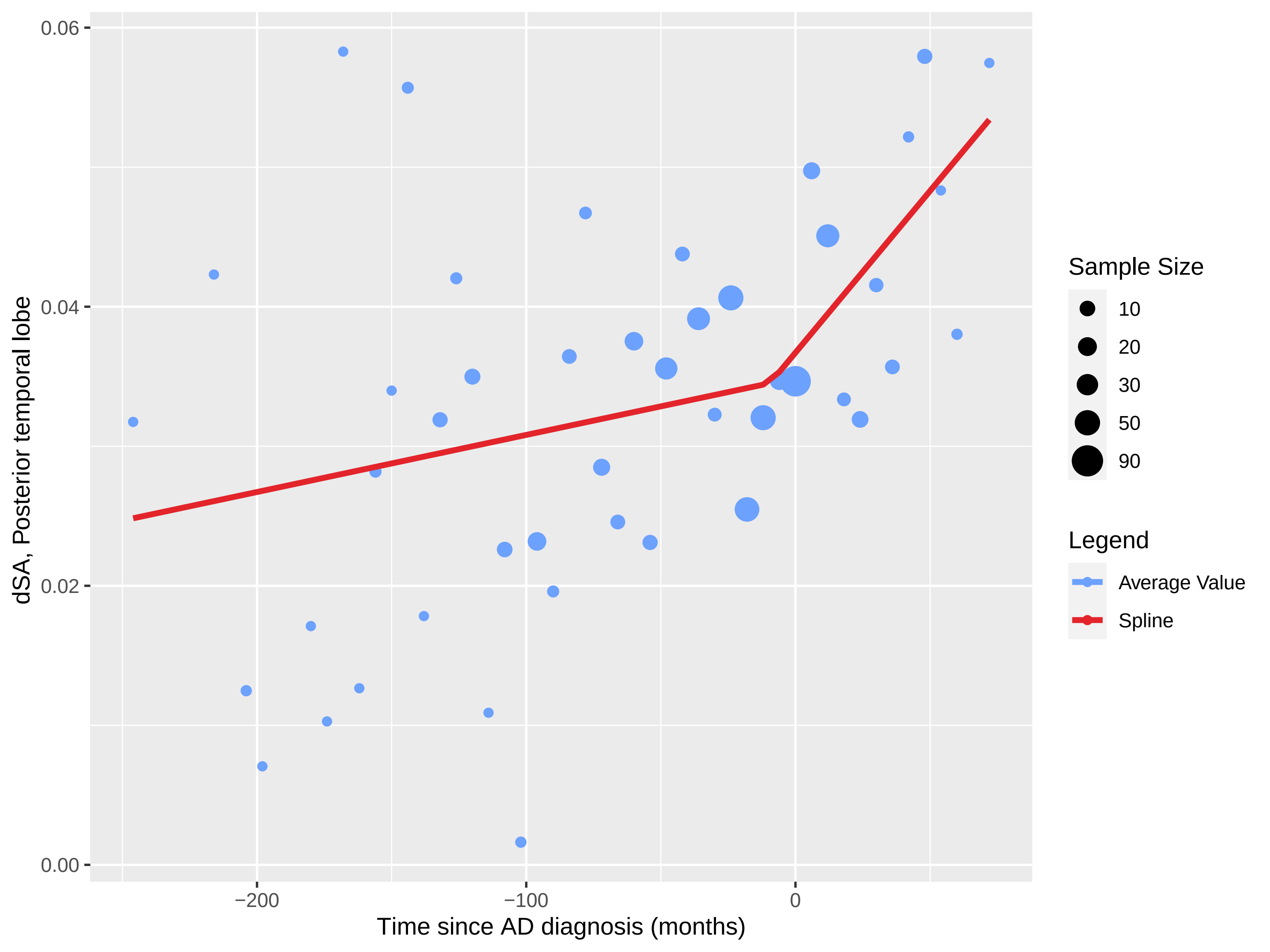

0.06
0.04
0.02
0.00
−200
−100
0
dSA, Posterior temporal lobe
Time since AD diagnosis (months)
Sample Size
10
20
30
50
90
Legend
Average Value
Spline

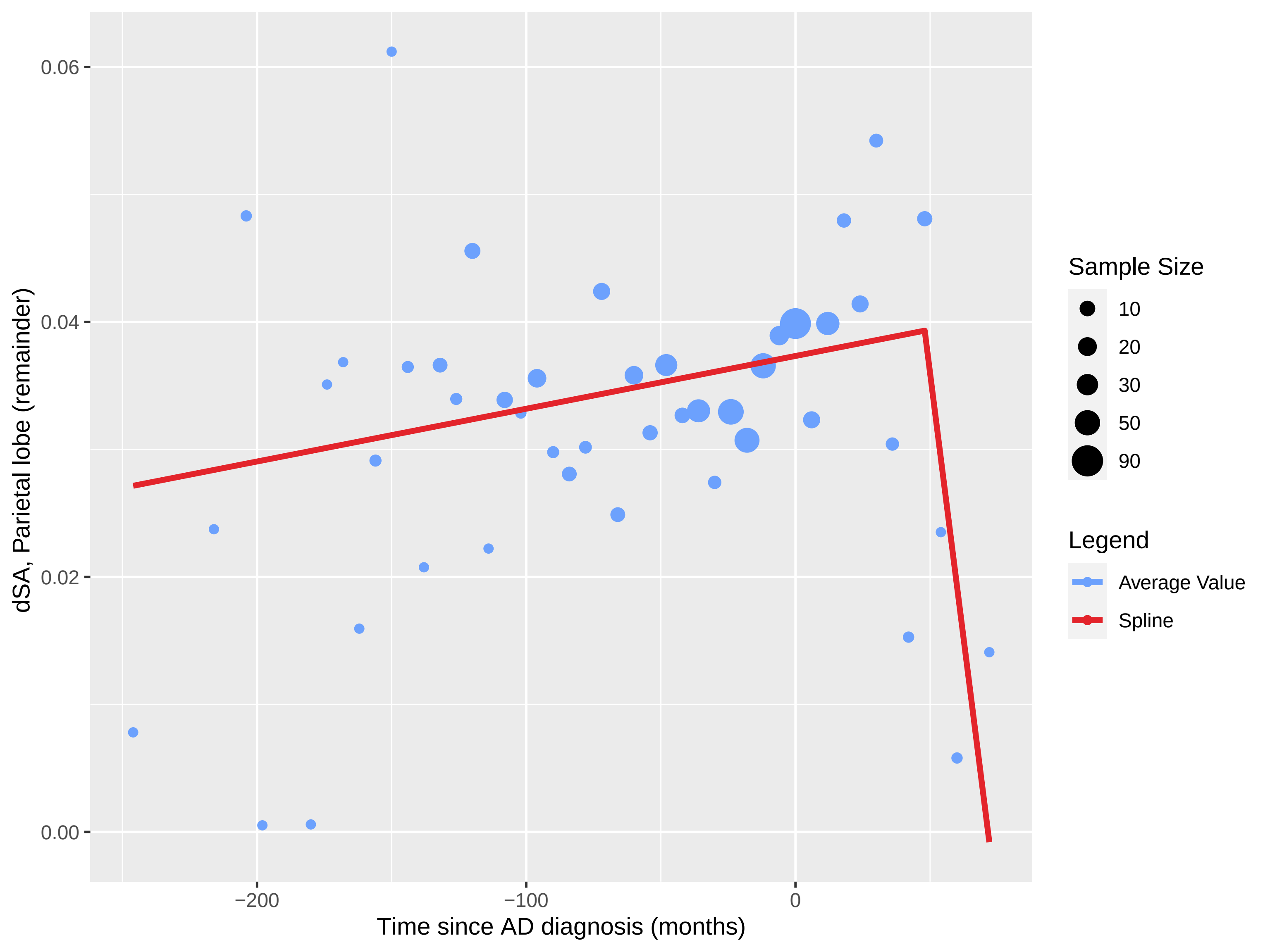

0.06
0.04
0.02
0.00
dSA, Parietal lobe (remainder)
−200
−100
0
Time since AD diagnosis (months)
Sample Size
10
20
30
50
90
Legend
Average Value
Spline

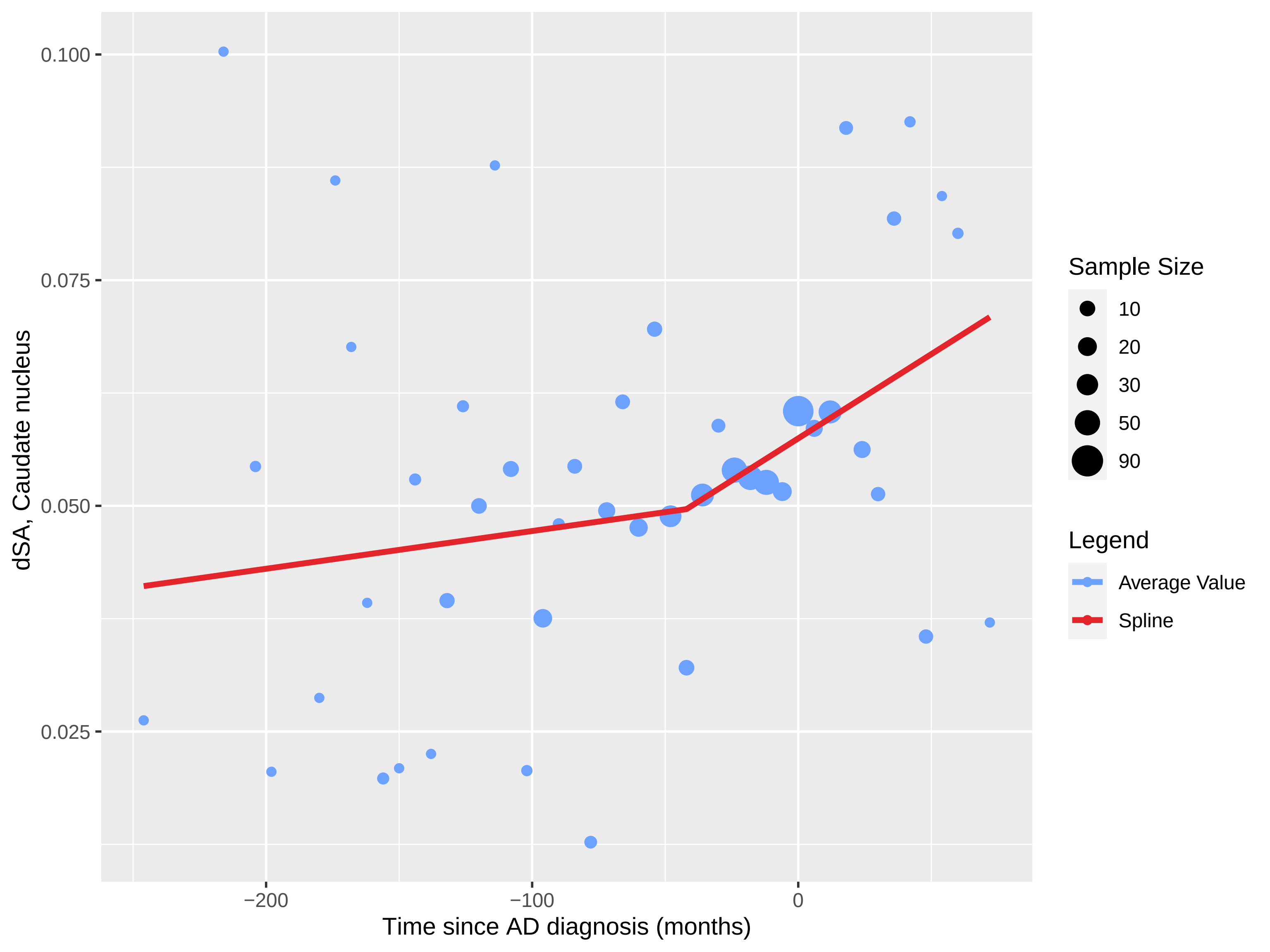

0.100
0.075
0.050
0.025
−200
−100
0
Time since AD diagnosis (months)
dSA, Caudate nucleus
Sample Size
10
20
30
50
90
Legend
Average Value
Spline

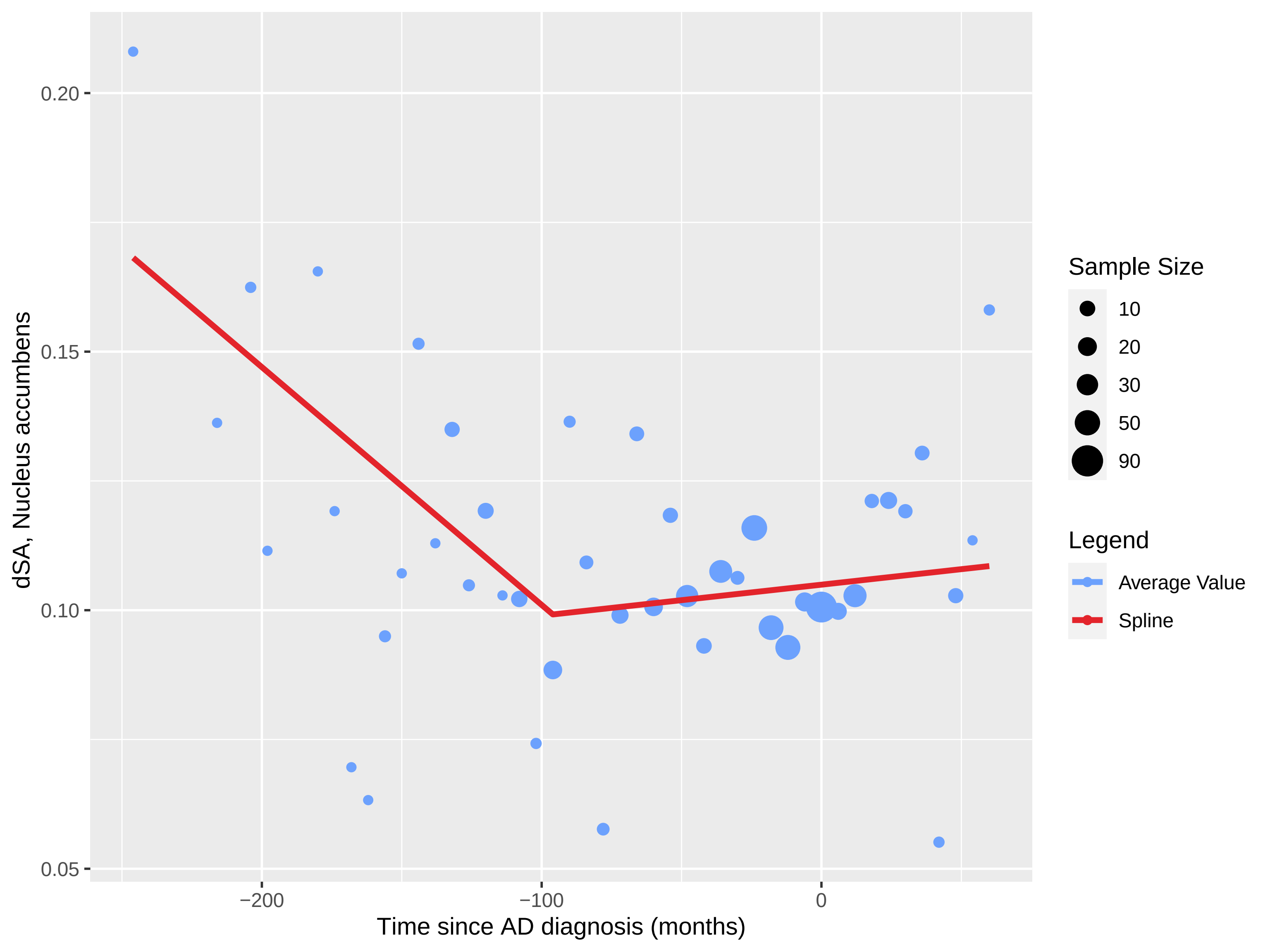

dSA, Nucleus accumbens
0.20
0.15
0.10
0.05
−200
−100
0
Time since AD diagnosis (months)
Sample Size
10
20
30
50
90
Legend
Average Value
Spline

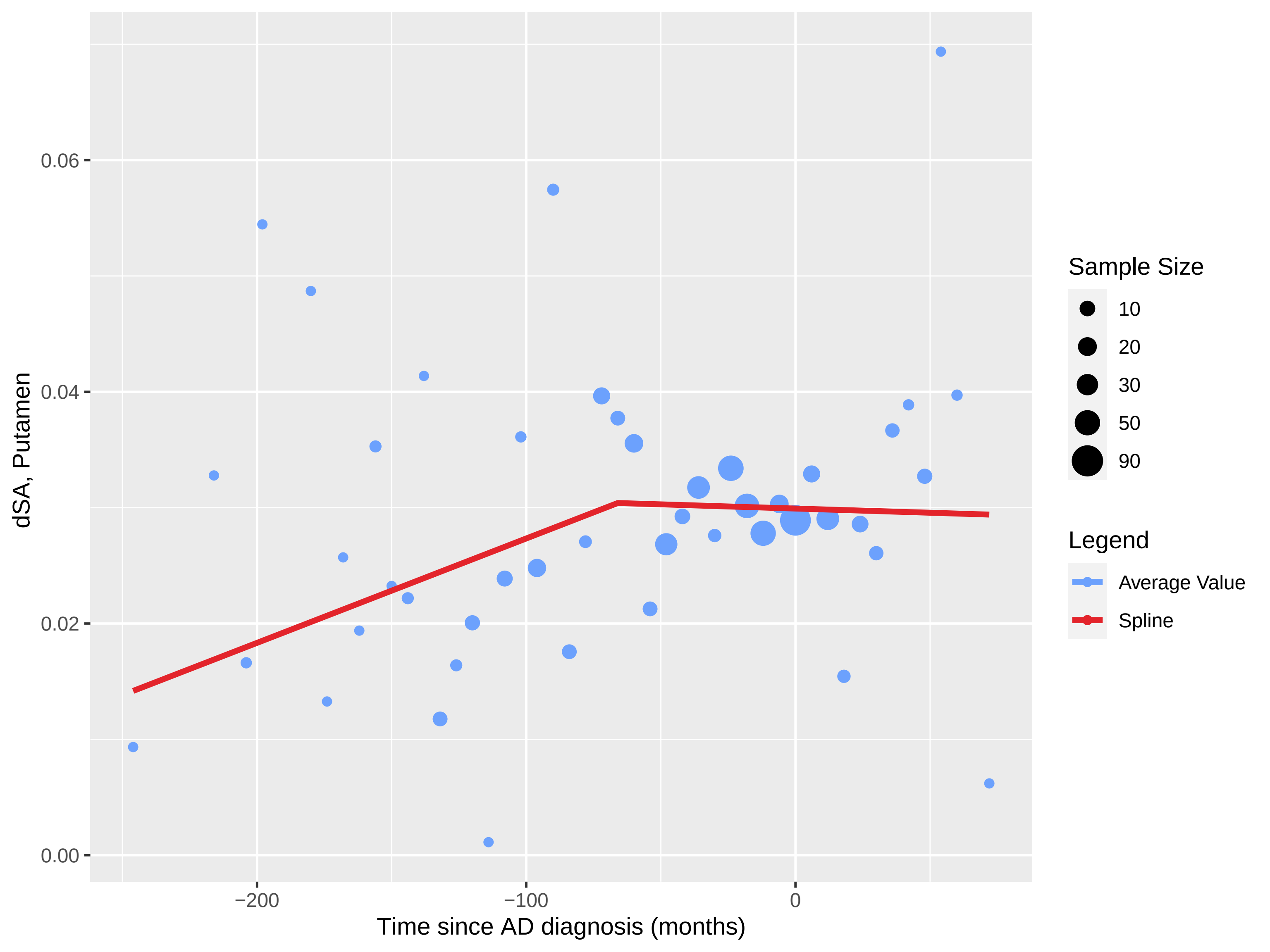

0.06
0.04
0.02
0.00
dSA, Putamen
−200
−100
0
Time since AD diagnosis (months)
Sample Size
10
20
30
50
90
Legend
Average Value
Spline

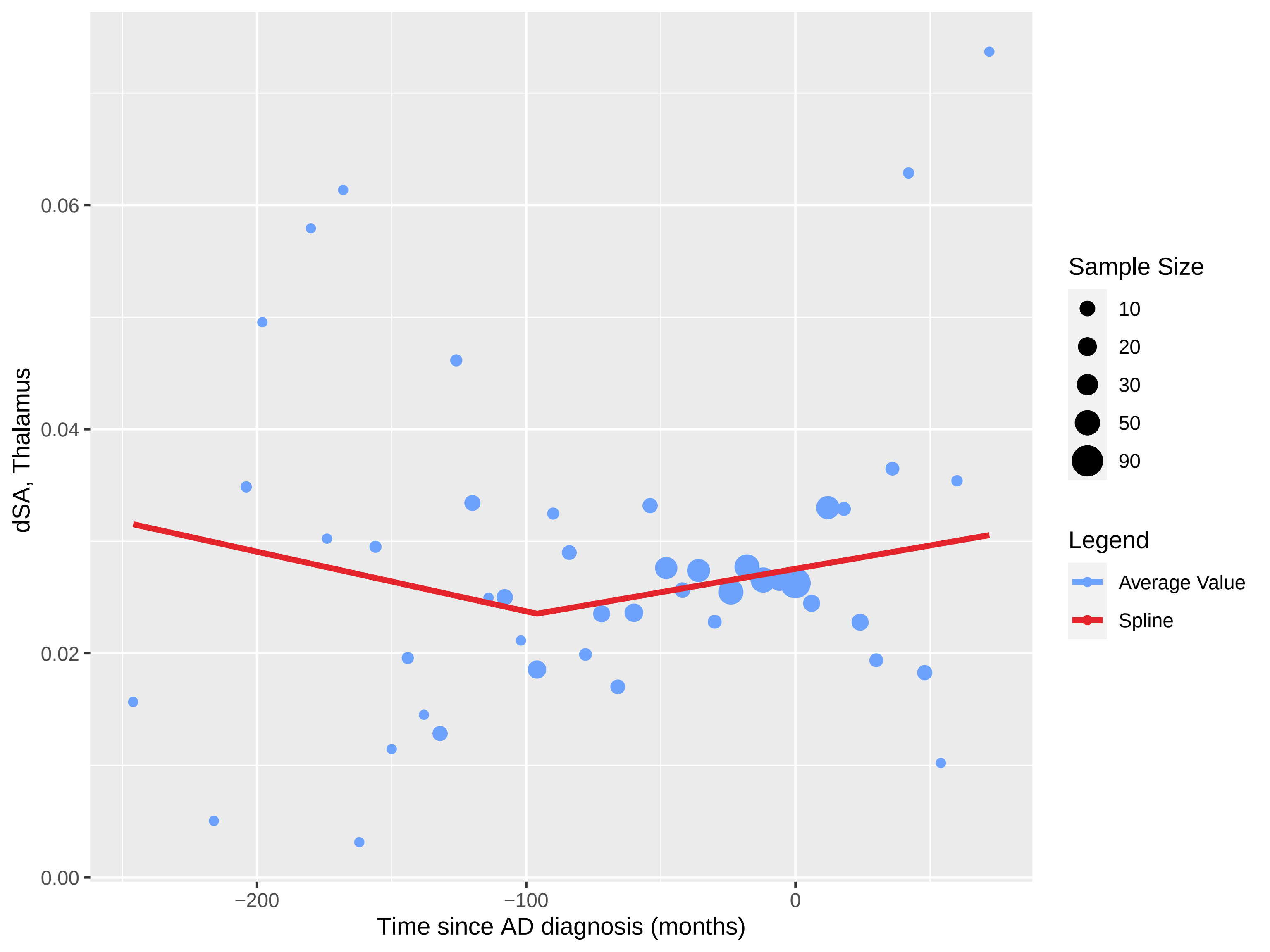

0.06
0.04
0.02
0.00
−200
−100
0
dSA, Thalamus
Time since AD diagnosis (months)
Sample Size
10
20
30
50
90
Legend
Average Value
Spline

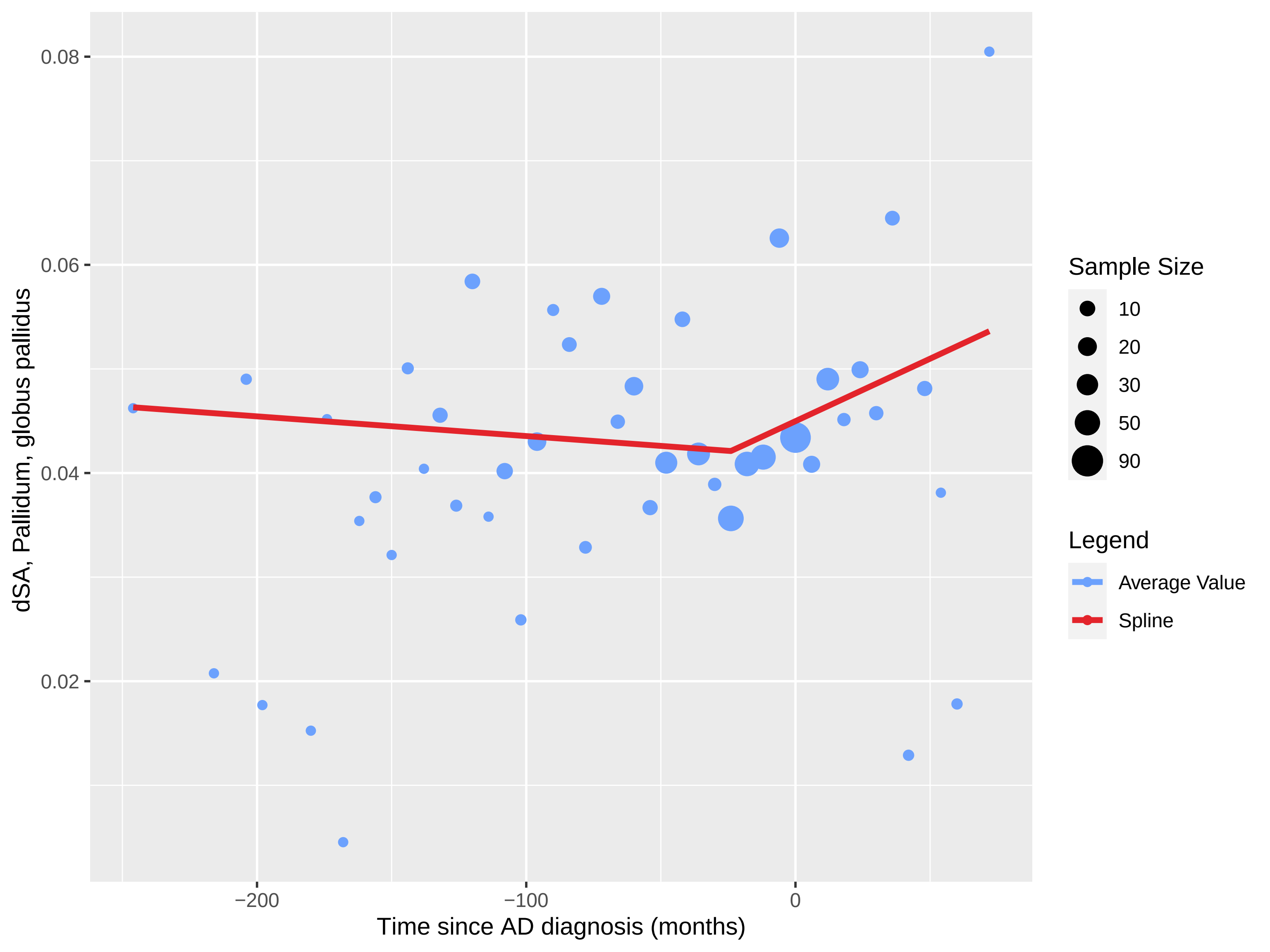

0.08
0.06
0.04
0.02
−200
−100
0
Time since AD diagnosis (months)
dSA, Pallidum, globus pallidus
Sample Size
10
20
30
50
90
Legend
Average Value
Spline

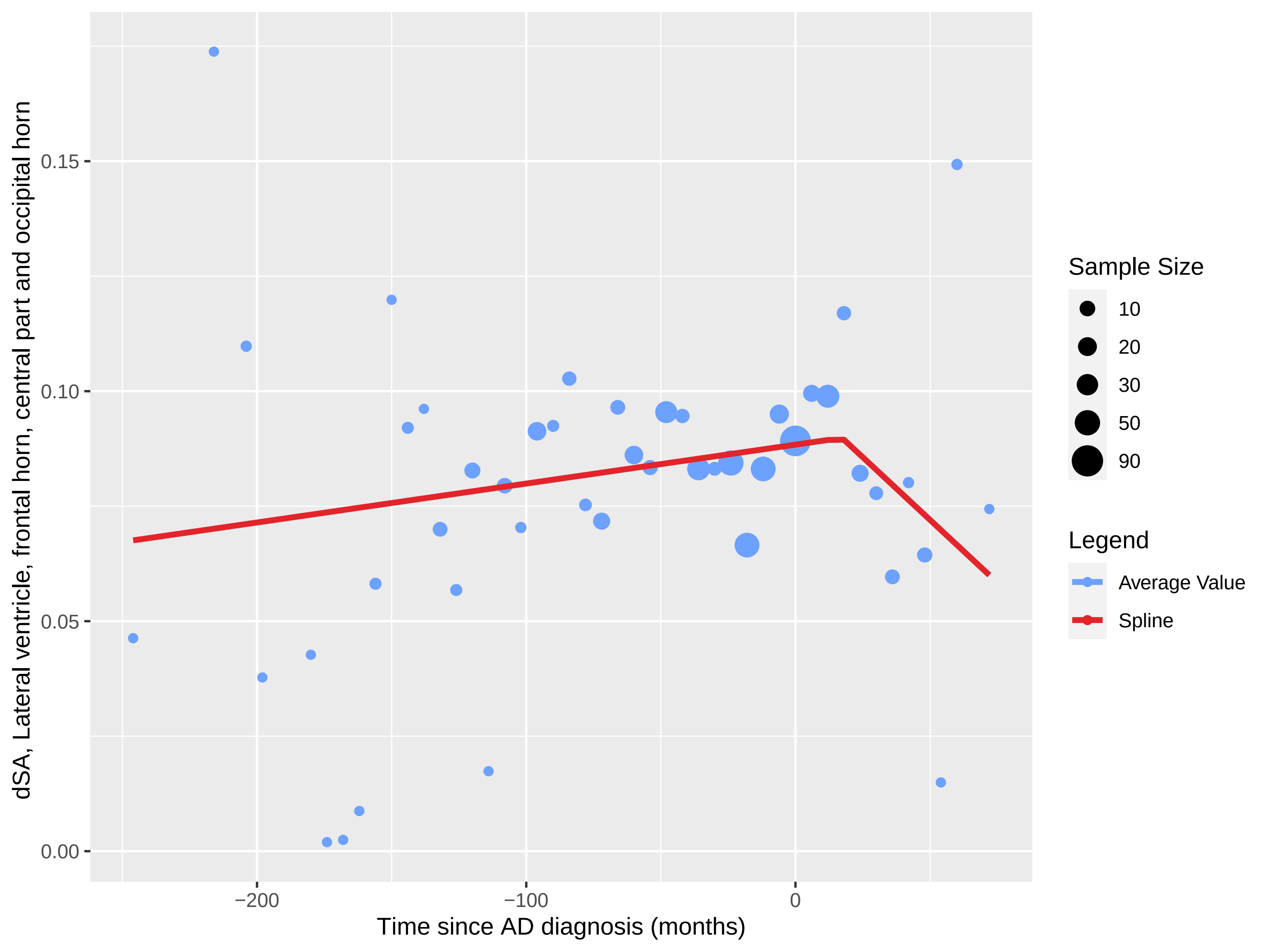
dSA, Lateral ventricle, frontal horn, central part and occipital horn
0.15
0.10
0.05
0.00
−200
−100
0
Time since AD diagnosis (months)
Sample Size
10
20
30
50
90
Legend
Average Value
Spline

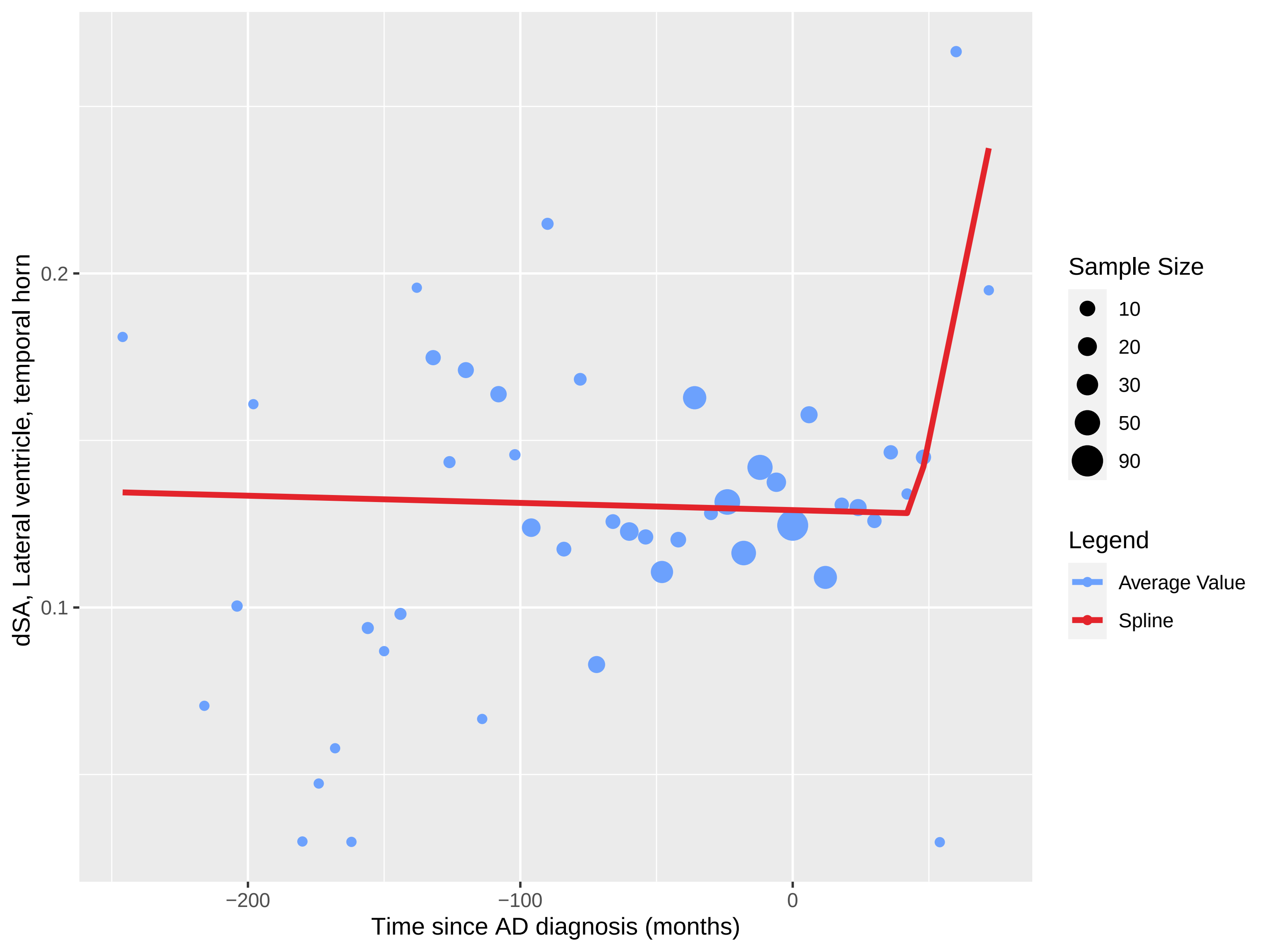

0.2
0.1
−200
−100
0
Time since AD diagnosis (months)
dSA, Lateral ventricle, temporal horn
Sample Size
10
20
30
50
90
Legend
Average Value
Spline

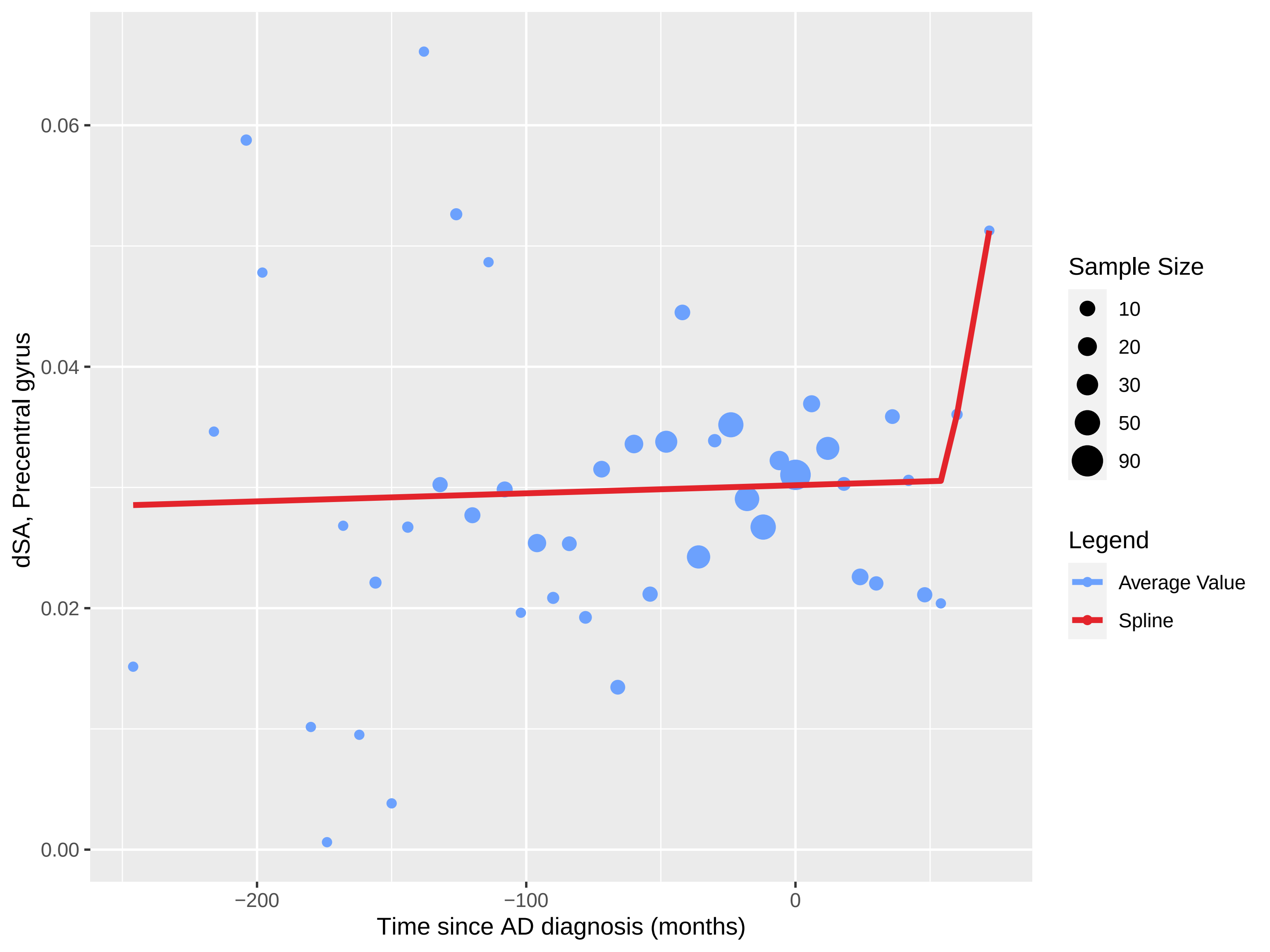
0.06
0.04
0.02
0.00
−200
−100
0
dSA, Precentral gyrus
Time since AD diagnosis (months)
Sample Size
10
20
30
50
90
Legend
Average Value
Spline

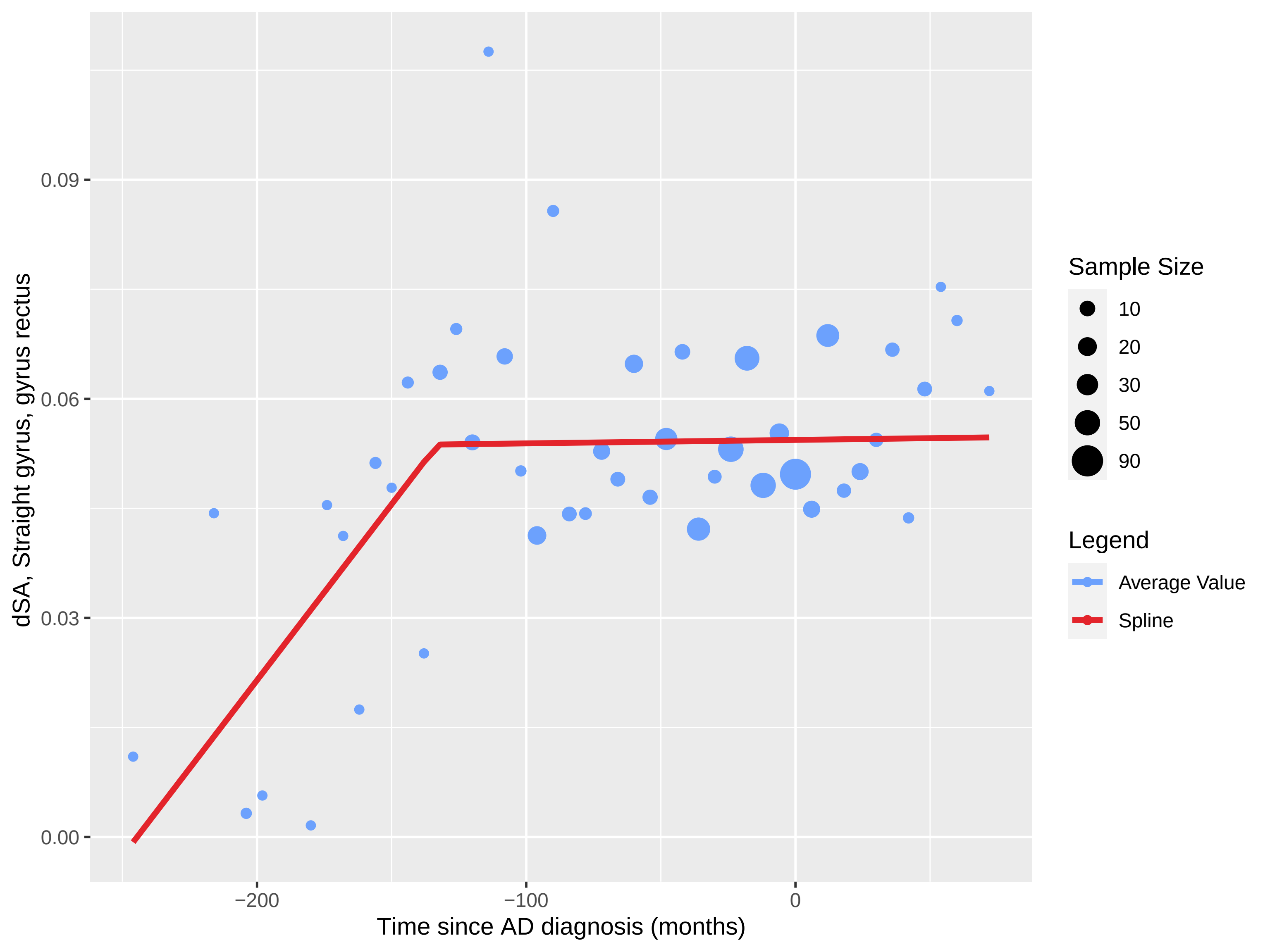

dSA, Straight gyrus, gyrus rectus
0.09
0.06
0.03
0.00
−200
−100
0
Time since AD diagnosis (months)
Sample Size
10
20
30
50
90
Legend
Average Value
Spline

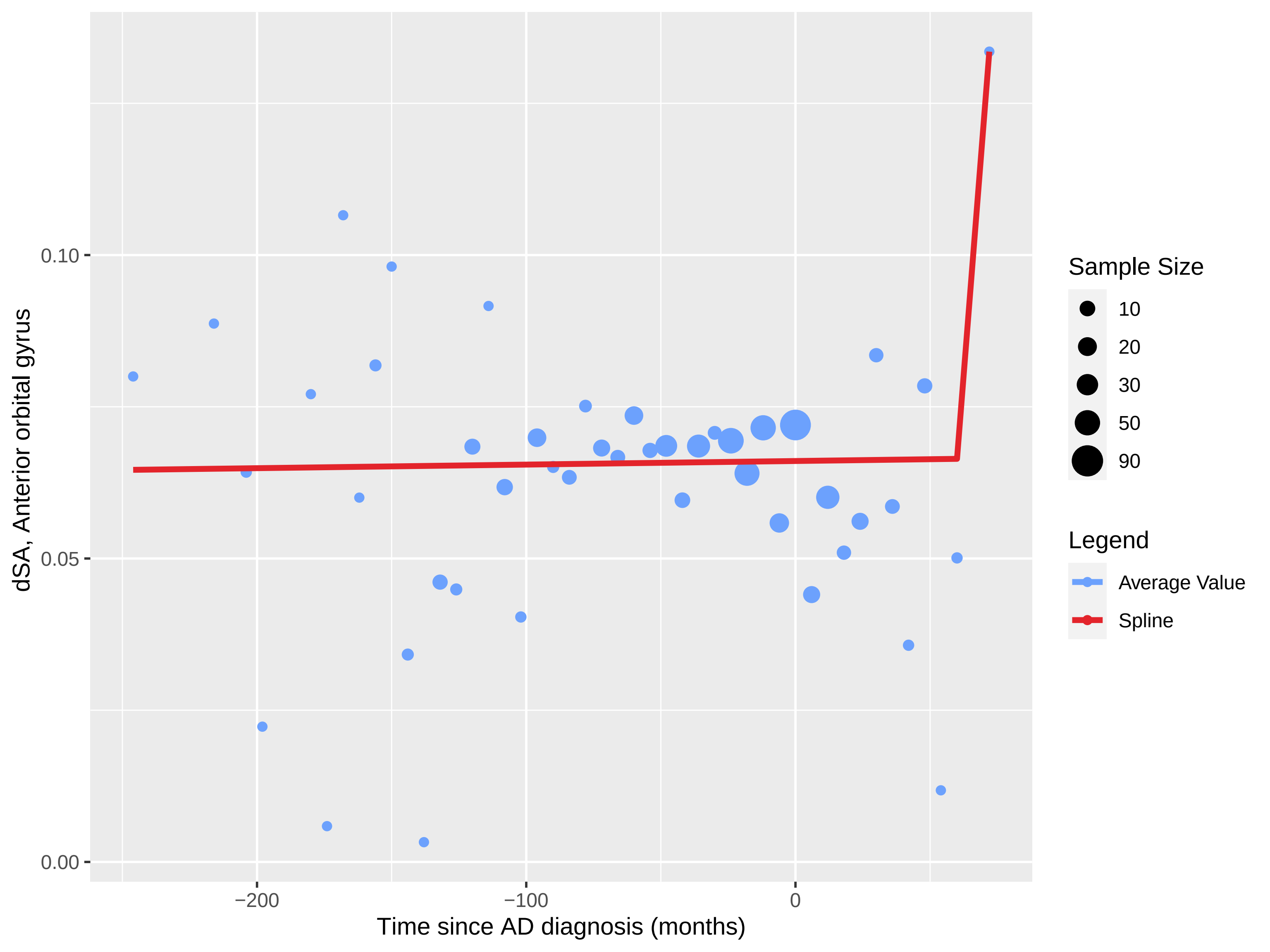

0.10
0.05
0.00
−200
−100
0
dSA, Anterior orbital gyrus
Time since AD diagnosis (months)
Sample Size
10
20
30
50
90
Legend
Average Value
Spline

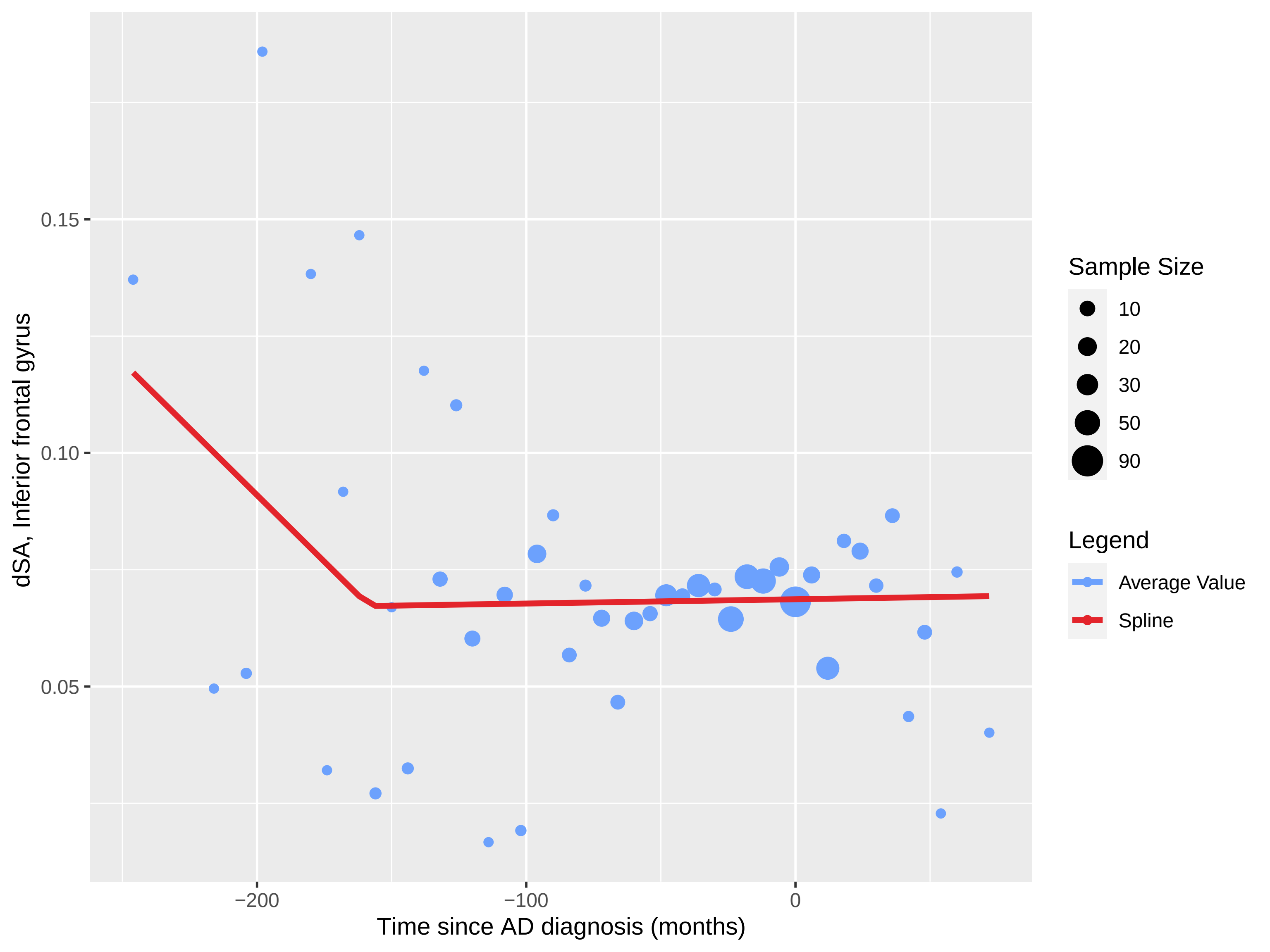

0.15
0.10
0.05
dSA, Inferior frontal gyrus
−200
−100
0
Time since AD diagnosis (months)
Sample Size
10
20
30
50
90
Legend
Average Value
Spline

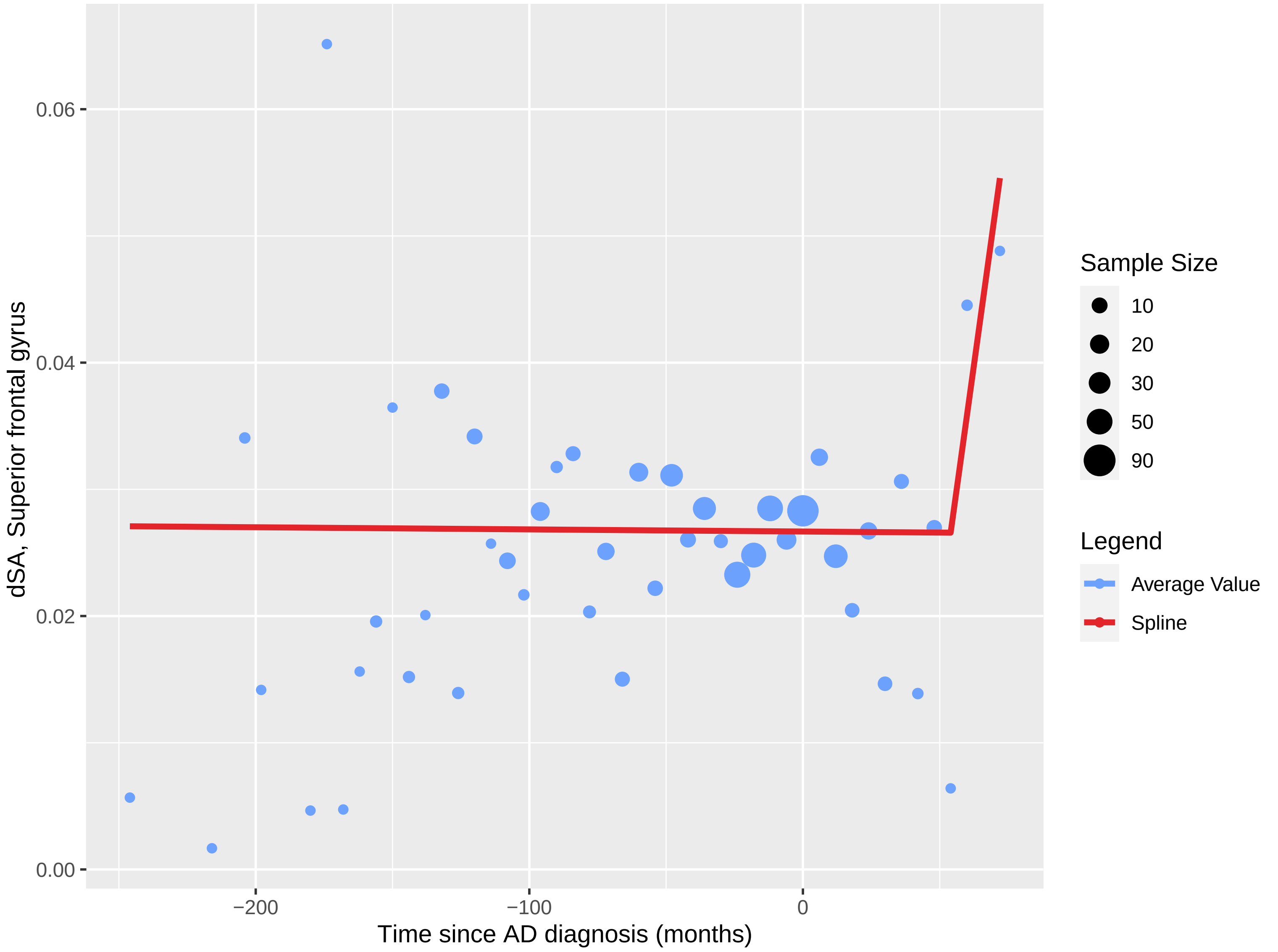

0.06
0.04
0.02
0.00
−200
−100
0
Time since AD diagnosis (months)
dSA, Superior frontal gyrus
Sample Size
10
20
30
50
90
Legend
Average Value
Spline

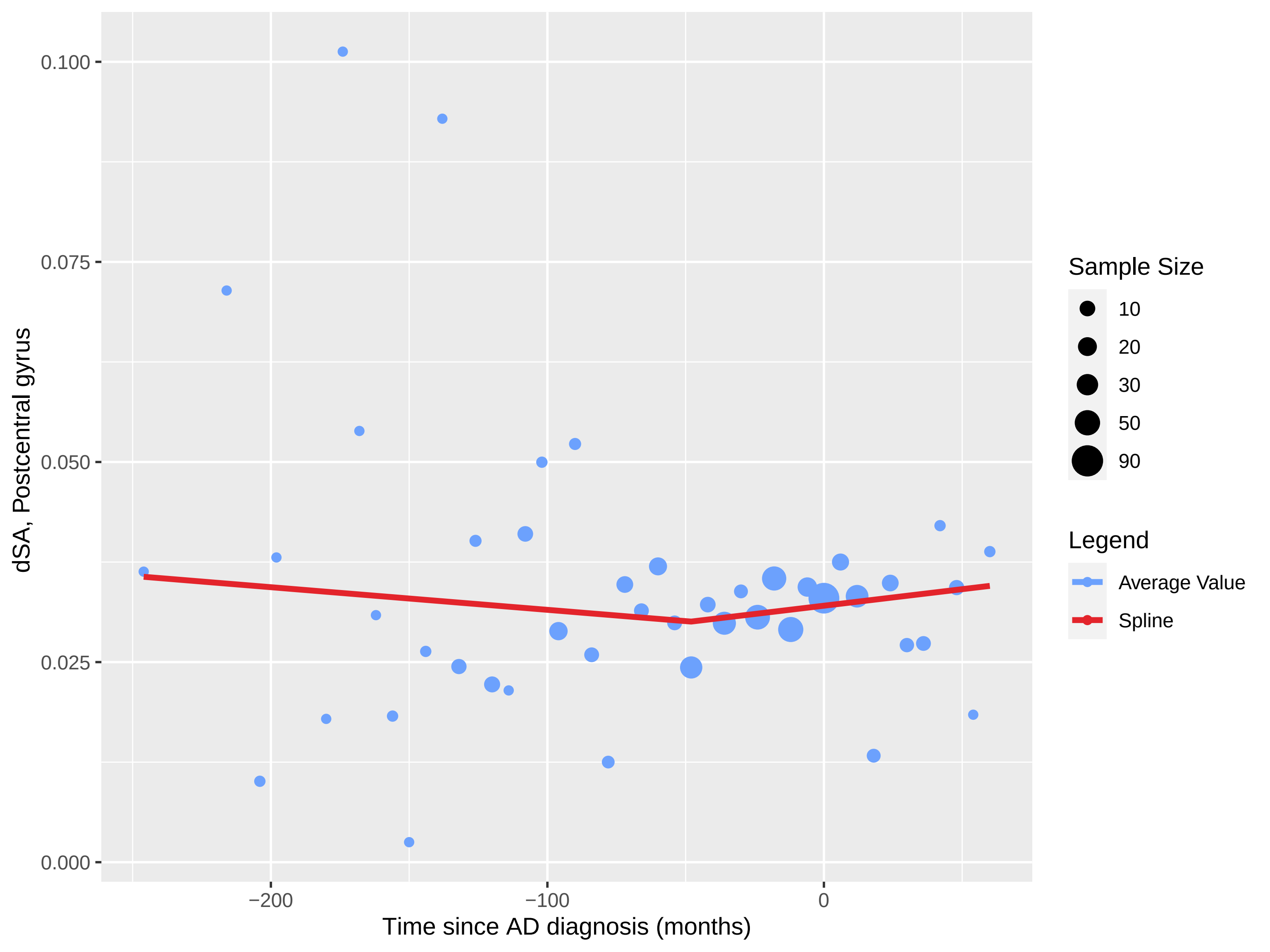

0.100
0.075
0.050
0.025
0.000
dSA, Postcentral gyrus
−200
−100
0
Time since AD diagnosis (months)
Sample Size
10
20
30
50
90
Legend
Average Value
Spline

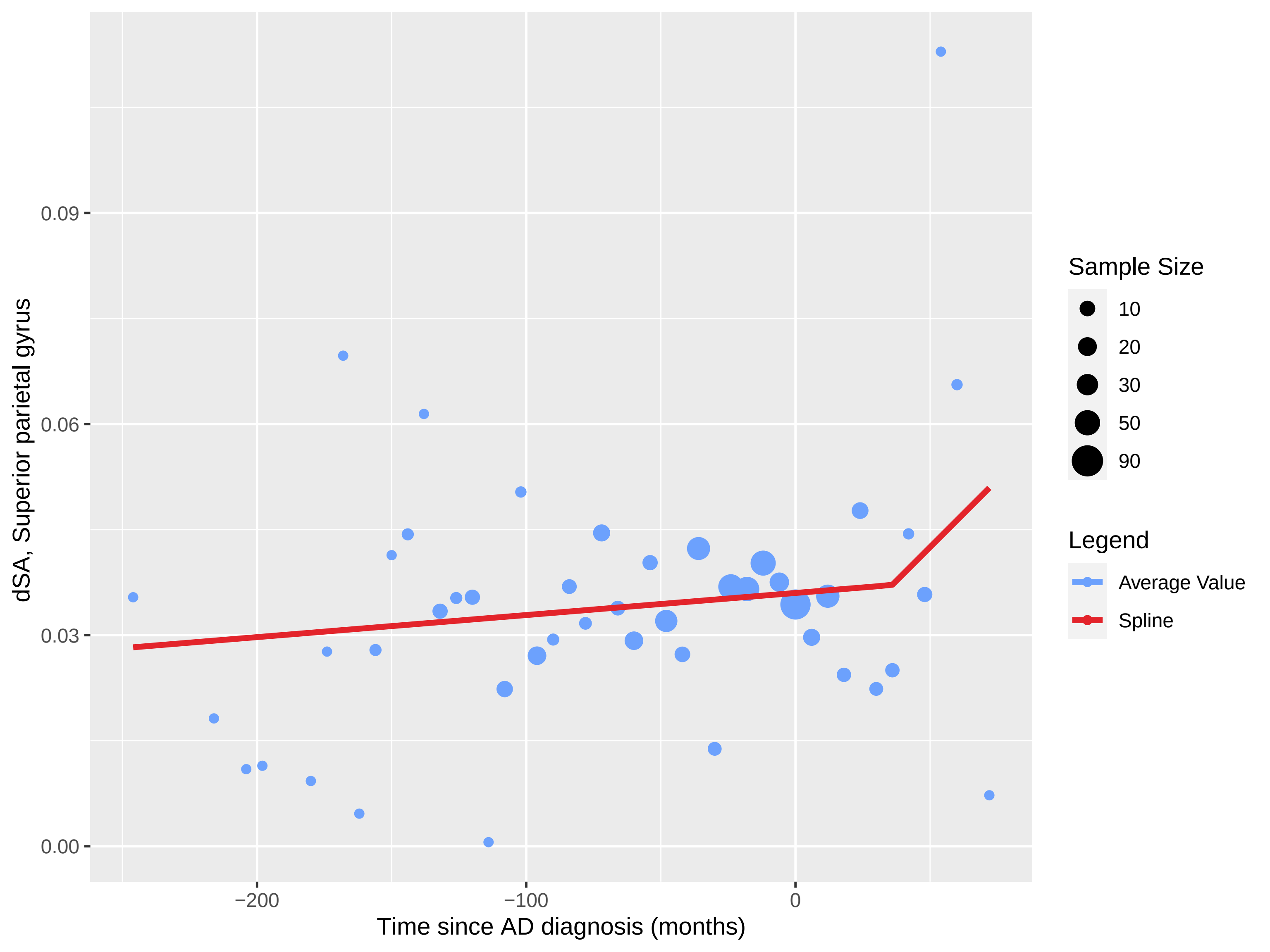

dSA, Superior parietal gyrus
0.00
0.03
0.06
0.09
−200
−100
0
Time since AD diagnosis (months)
Sample Size
10
20
30
50
90
Legend
Average Value
Spline

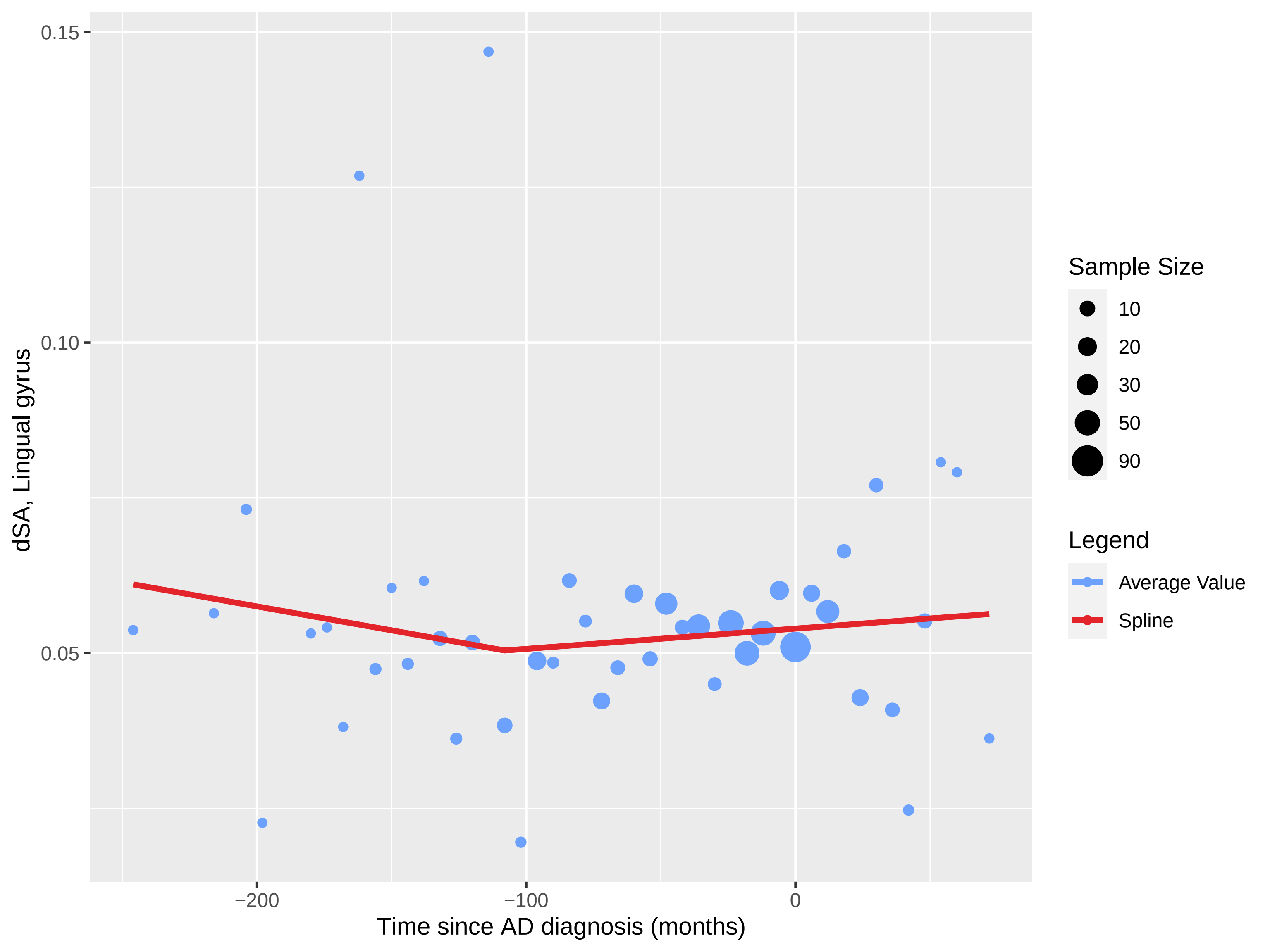

0.15
0.10
0.05
−200
−100
0
Time since AD diagnosis (months)
dSA, Lingual gyrus
Sample Size
10
20
30
50
90
Legend
Average Value
Spline

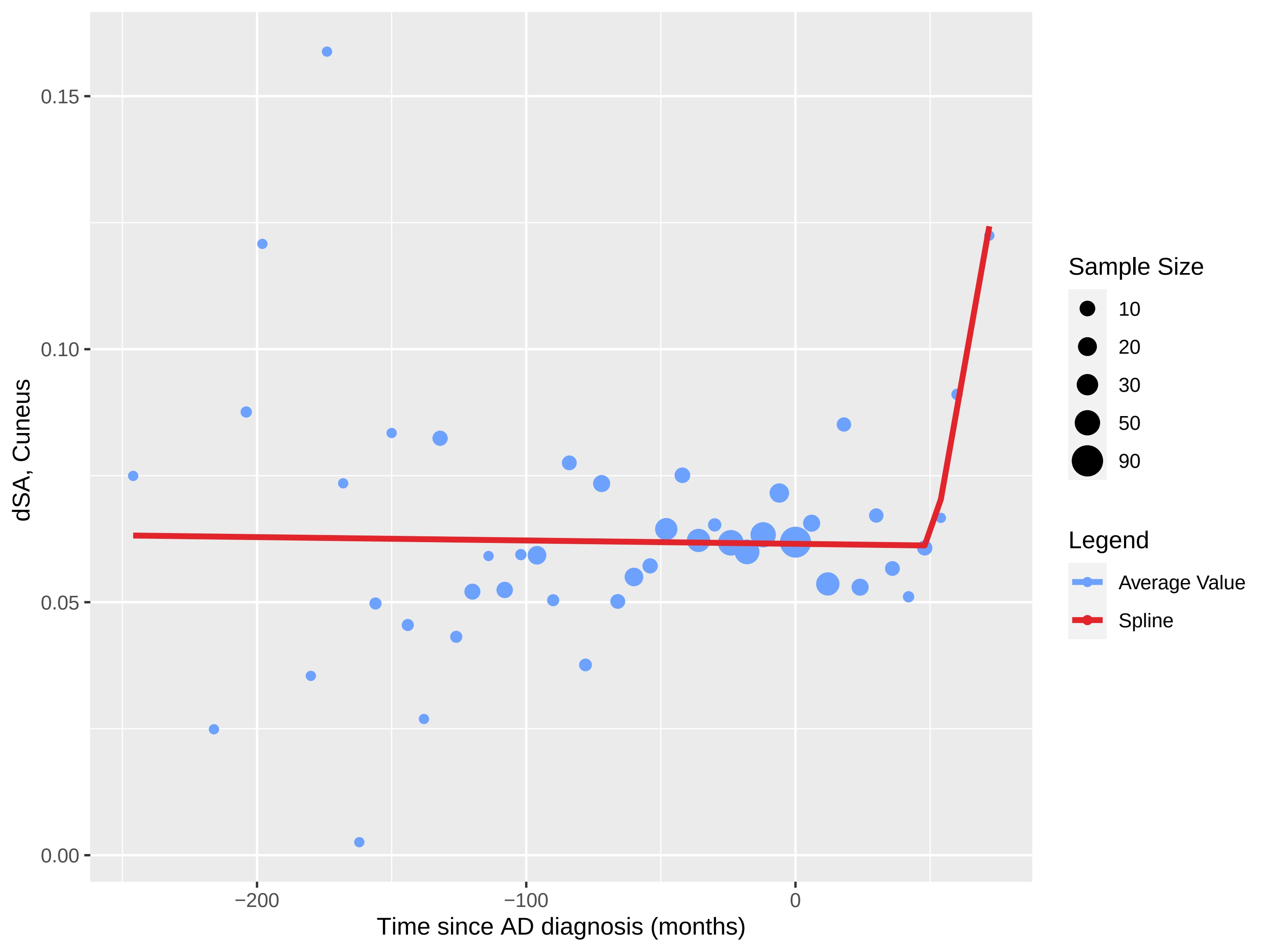

0.15
0.10
0.05
0.00
dSA, Cuneus
−200
−100
0
Time since AD diagnosis (months)
Sample Size
10
20
30
50
90
Legend
Average Value
Spline

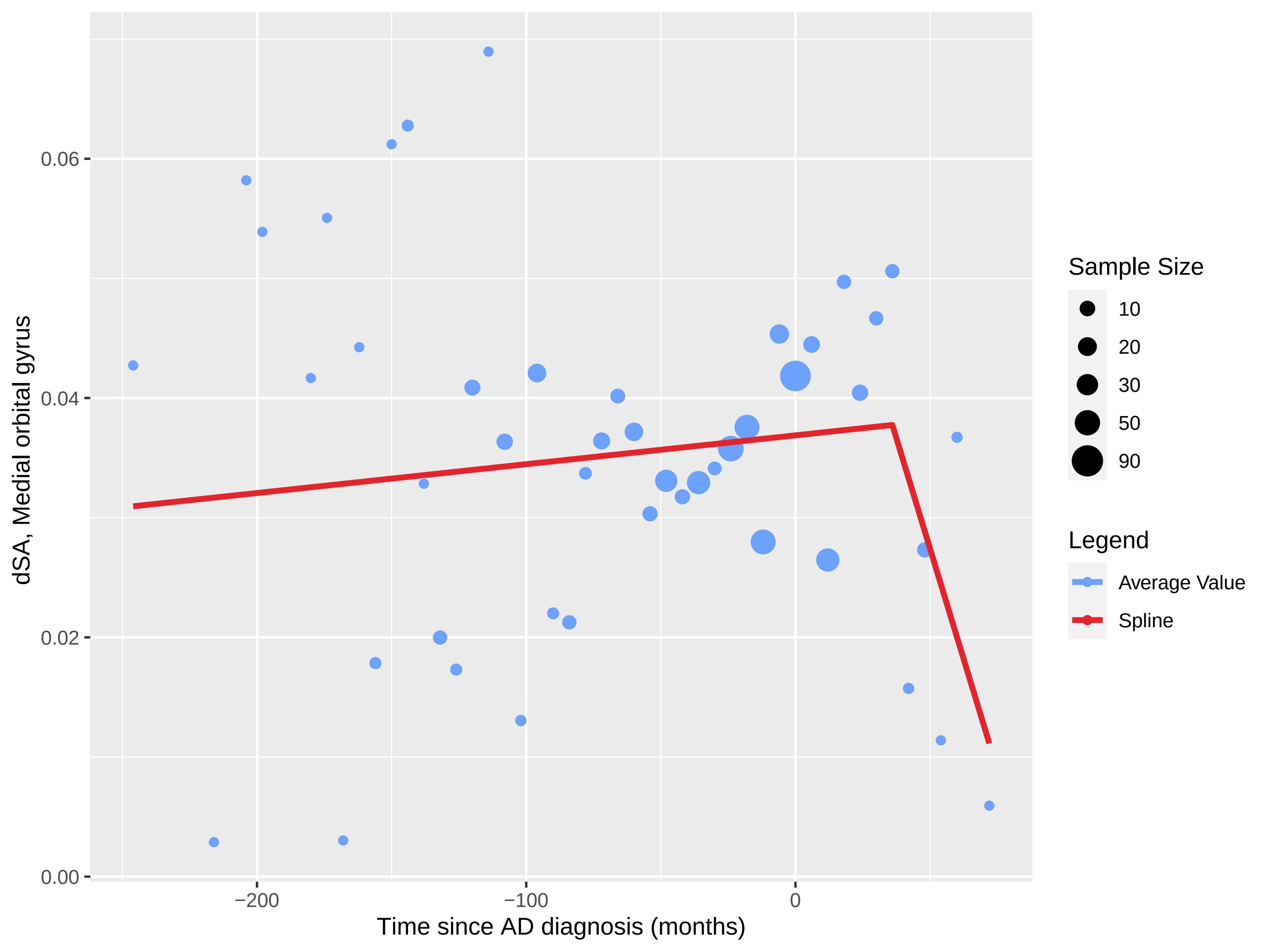

dSA, Medial orbital gyrus
0.06
0.04
0.02
0.00
−200
−100
0
Time since AD diagnosis (months)
Sample Size
10
20
30
50
90
Legend
Average Value
Spline

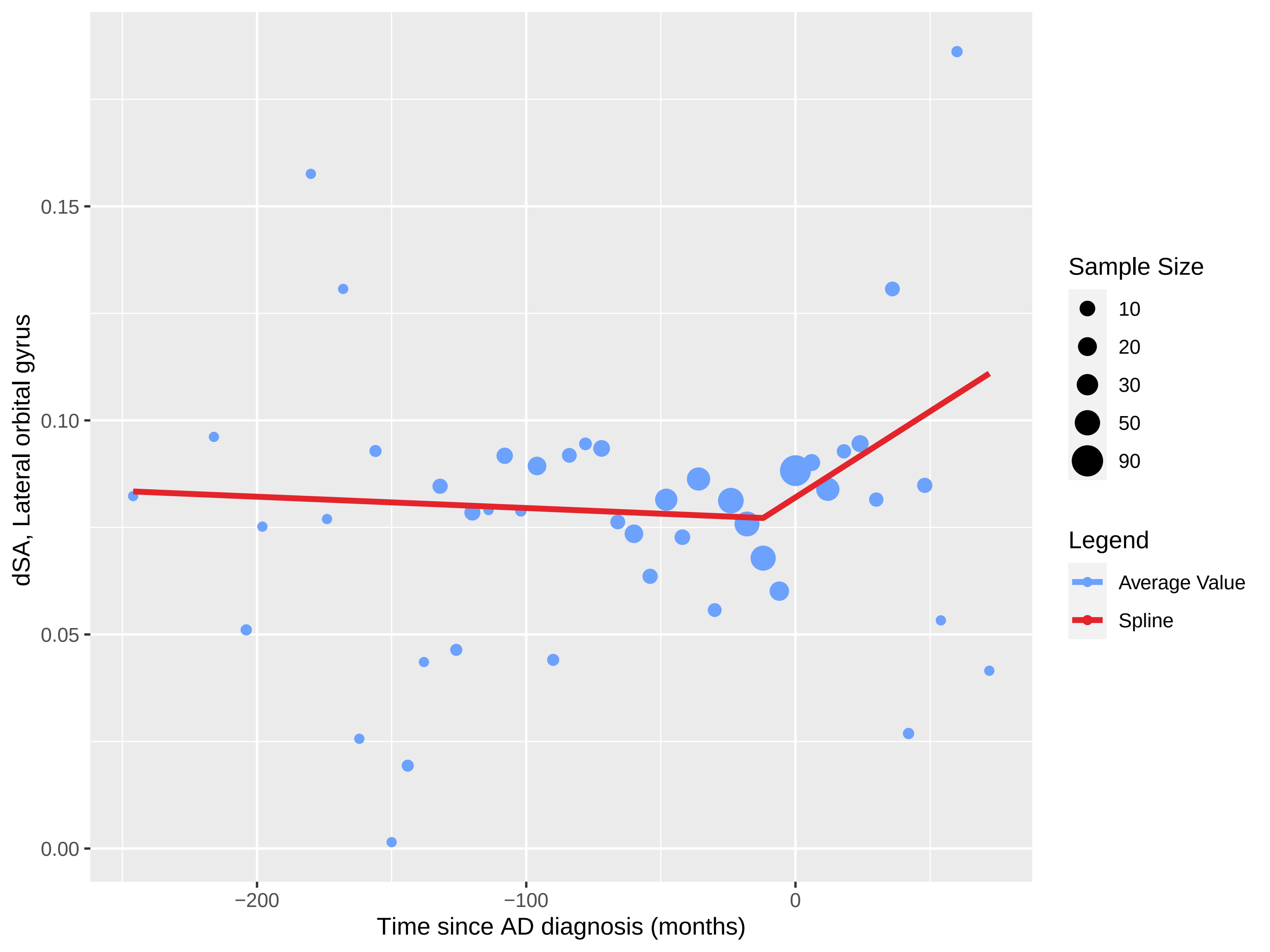

dSA, Lateral orbital gyrus
0.15
0.10
0.05
0.00
−200
−100
0
Time since AD diagnosis (months)
Sample Size
10
20
30
50
90
Legend
Average Value
Spline

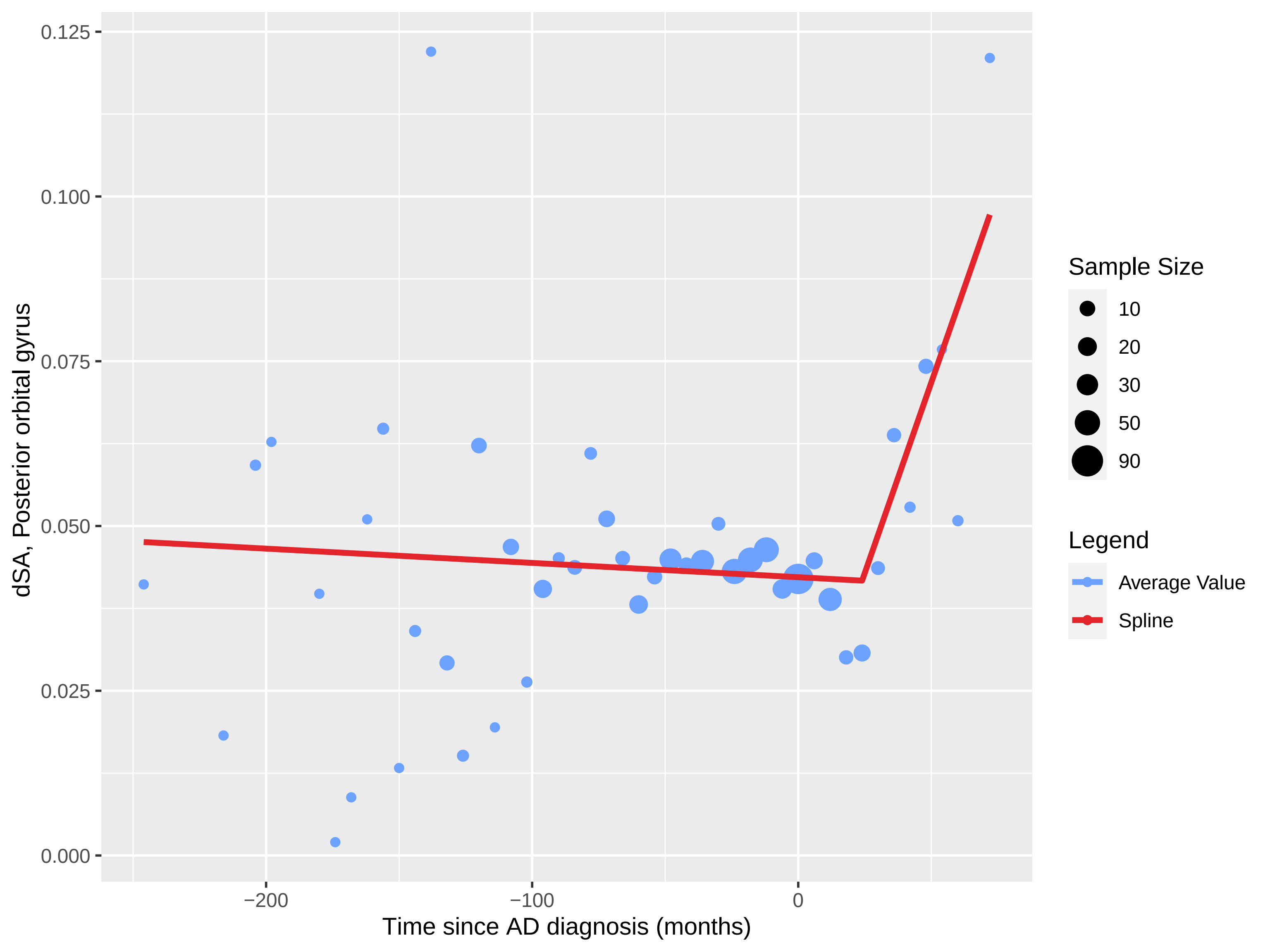

dSA, Posterior orbital gyrus
0.125
0.100
0.075
0.050
0.025
0.000
−200
−100
0
Time since AD diagnosis (months)
Sample Size
10
20
30
50
90
Legend
Average Value
Spline

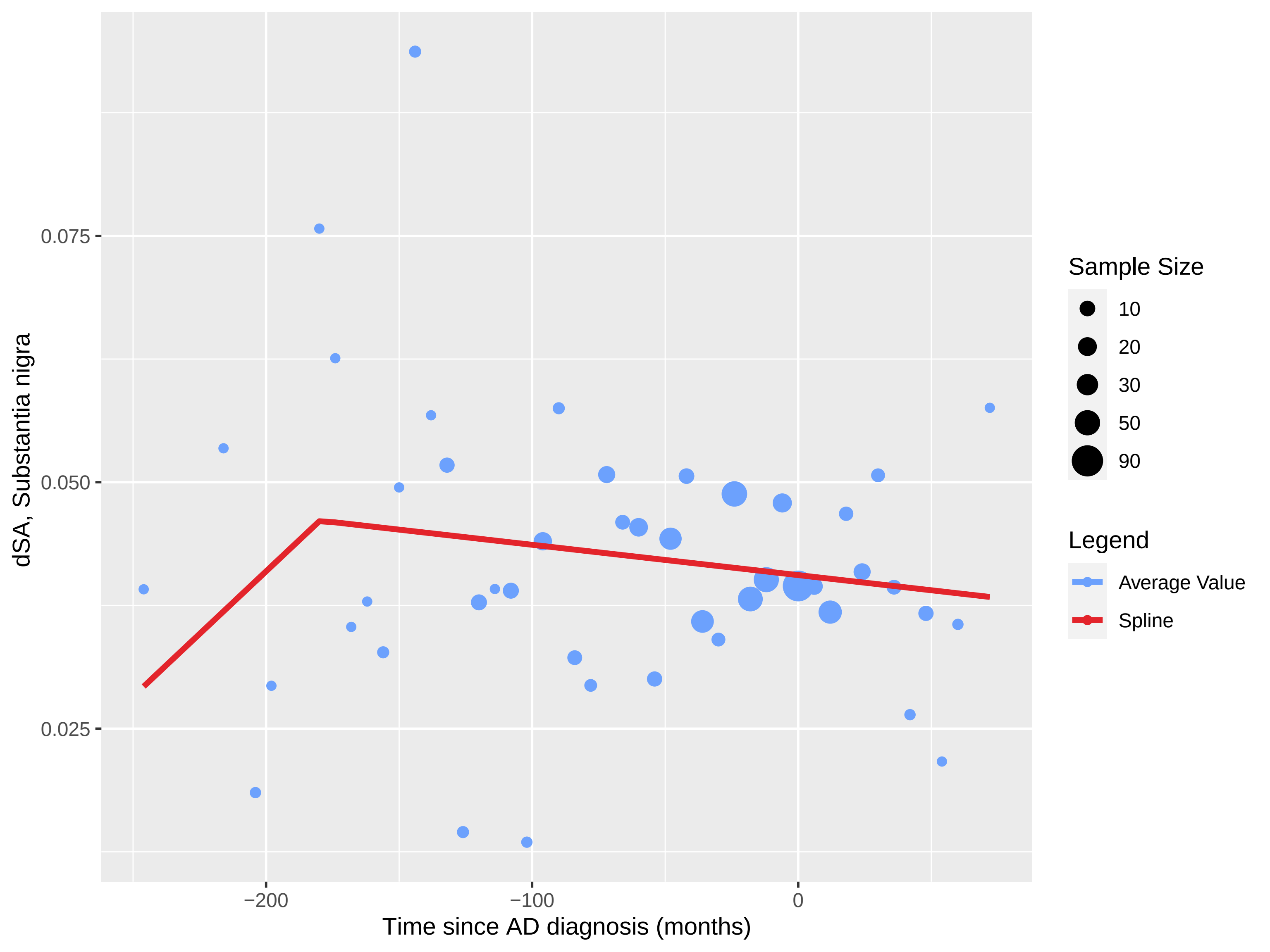

dSA, Substantia nigra
0.075
0.050
0.025
−200
−100
0
Time since AD diagnosis (months)
Sample Size
10
20
30
50
90
Legend
Average Value
Spline

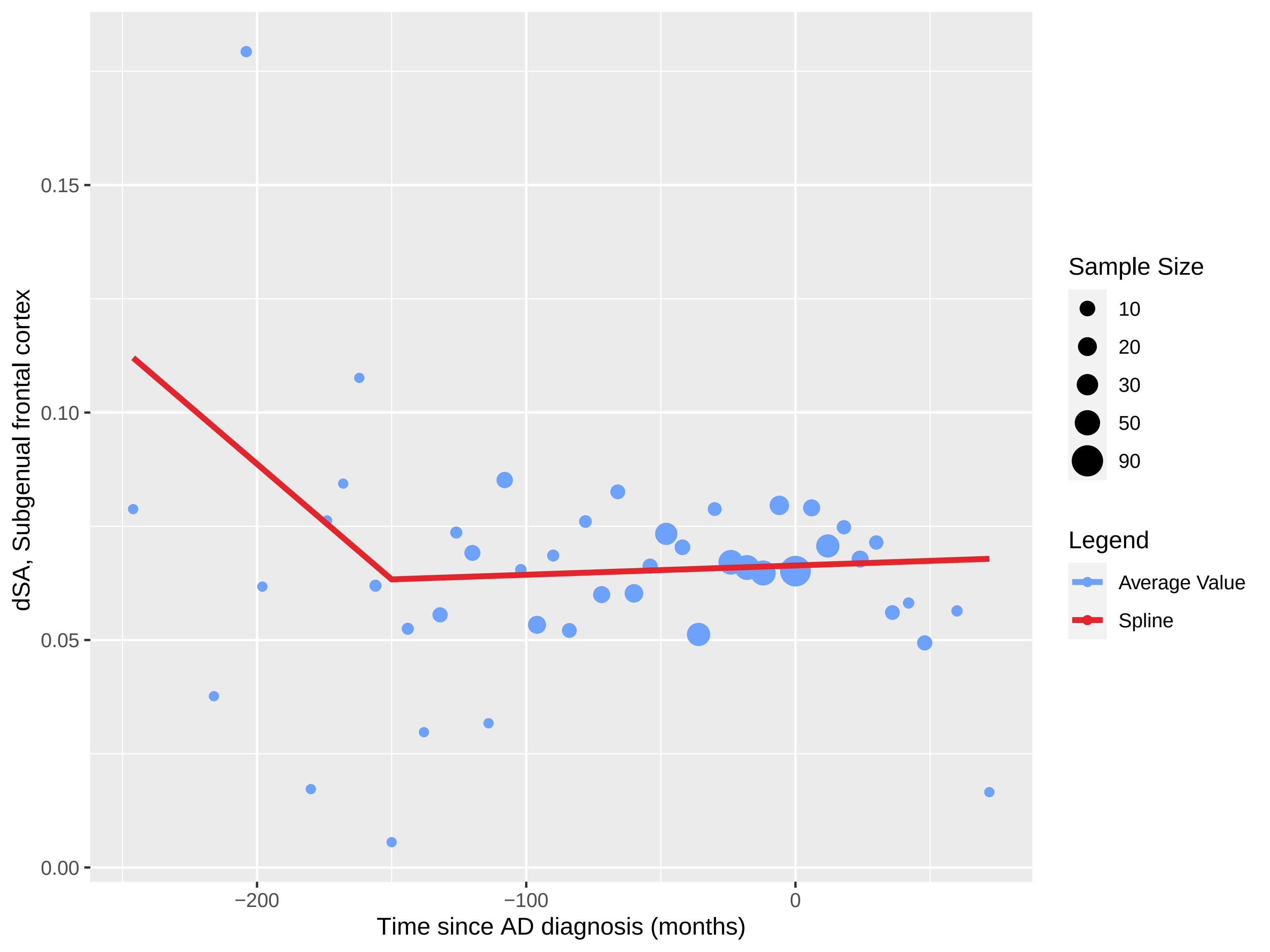

0.15
0.10
0.05
0.00
dSA, Subgenual frontal cortex
−200
−100
0
Time since AD diagnosis (months)
Sample Size
10
20
30
50
90
Legend
Average Value
Spline

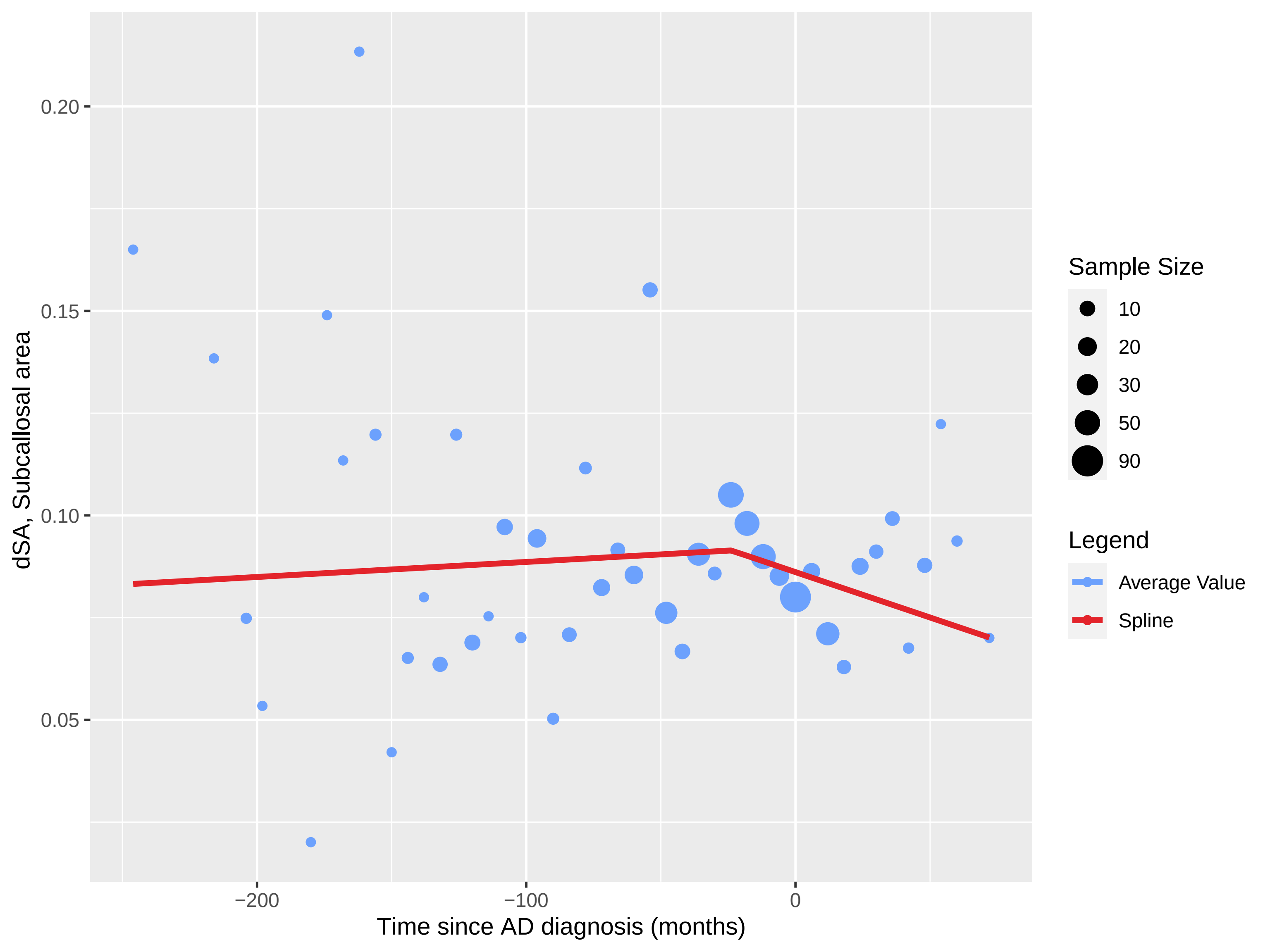

dSA, Subcallosal area
0.20
0.15
0.10
0.05
−200
−100
0
Time since AD diagnosis (months)
Sample Size
10
20
30
50
90
Legend
Average Value
Spline

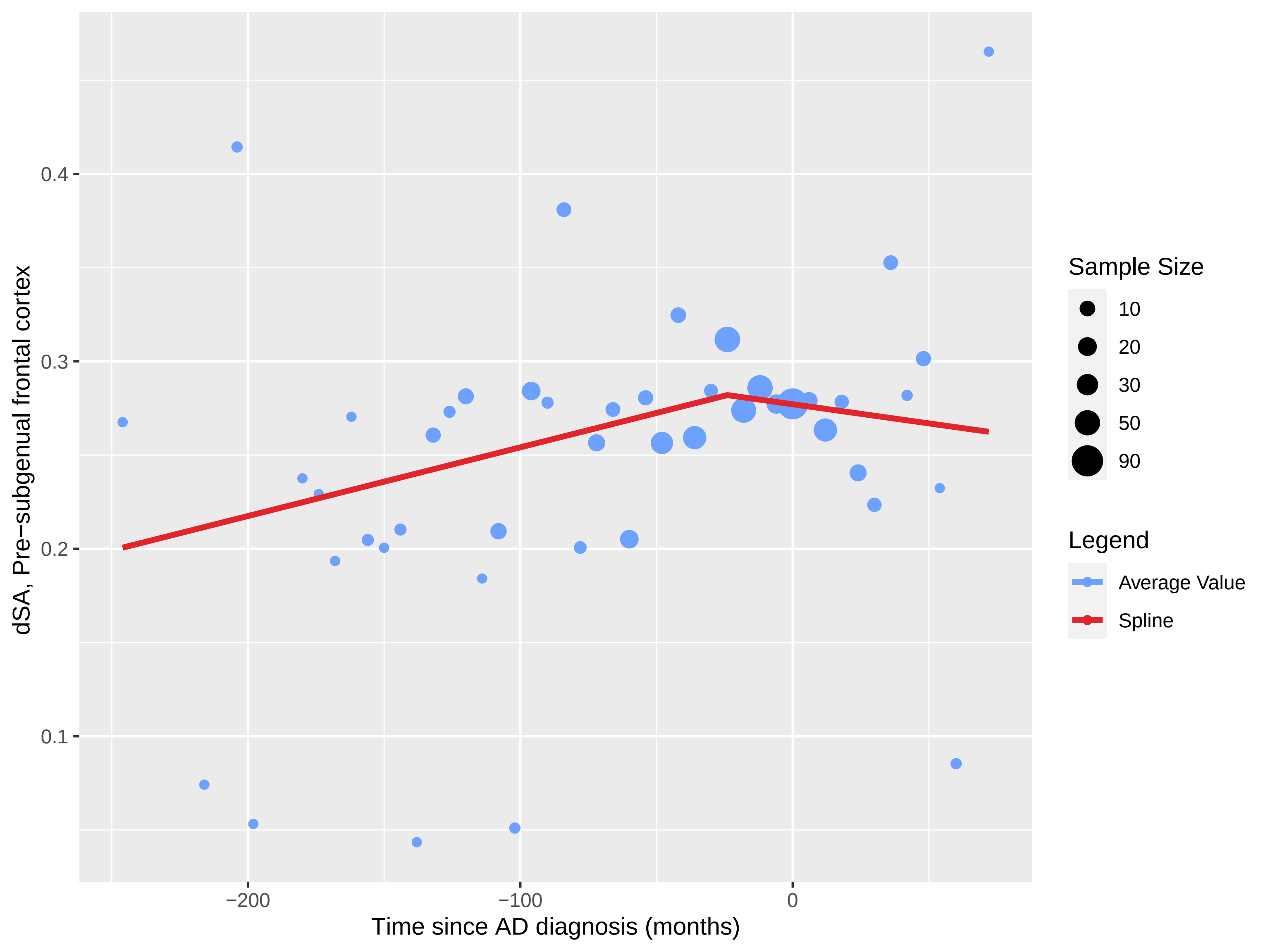

dSA, Pre−subgenual frontal cortex
0.4
0.3
0.2
0.1
−200
−100
0
Time since AD diagnosis (months)
Sample Size
10
20
30
50
90
Legend
Average Value
Spline

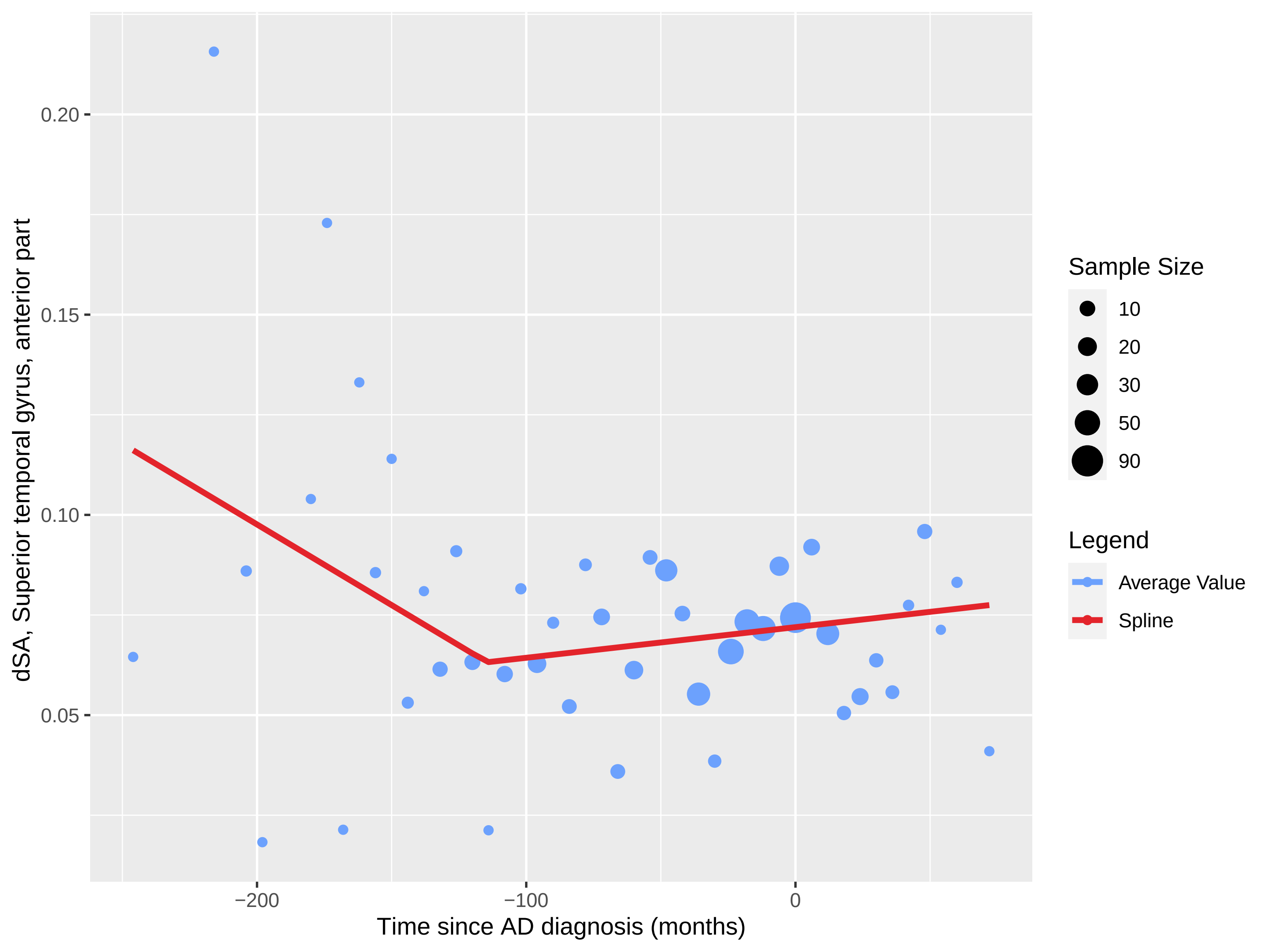

dSA, Superior temporal gyrus, anterior part
0.20
0.15
0.10
0.05
−200
−100
0
Time since AD diagnosis (months)
Sample Size
10
20
30
50
90
Legend
Average Value
Spline

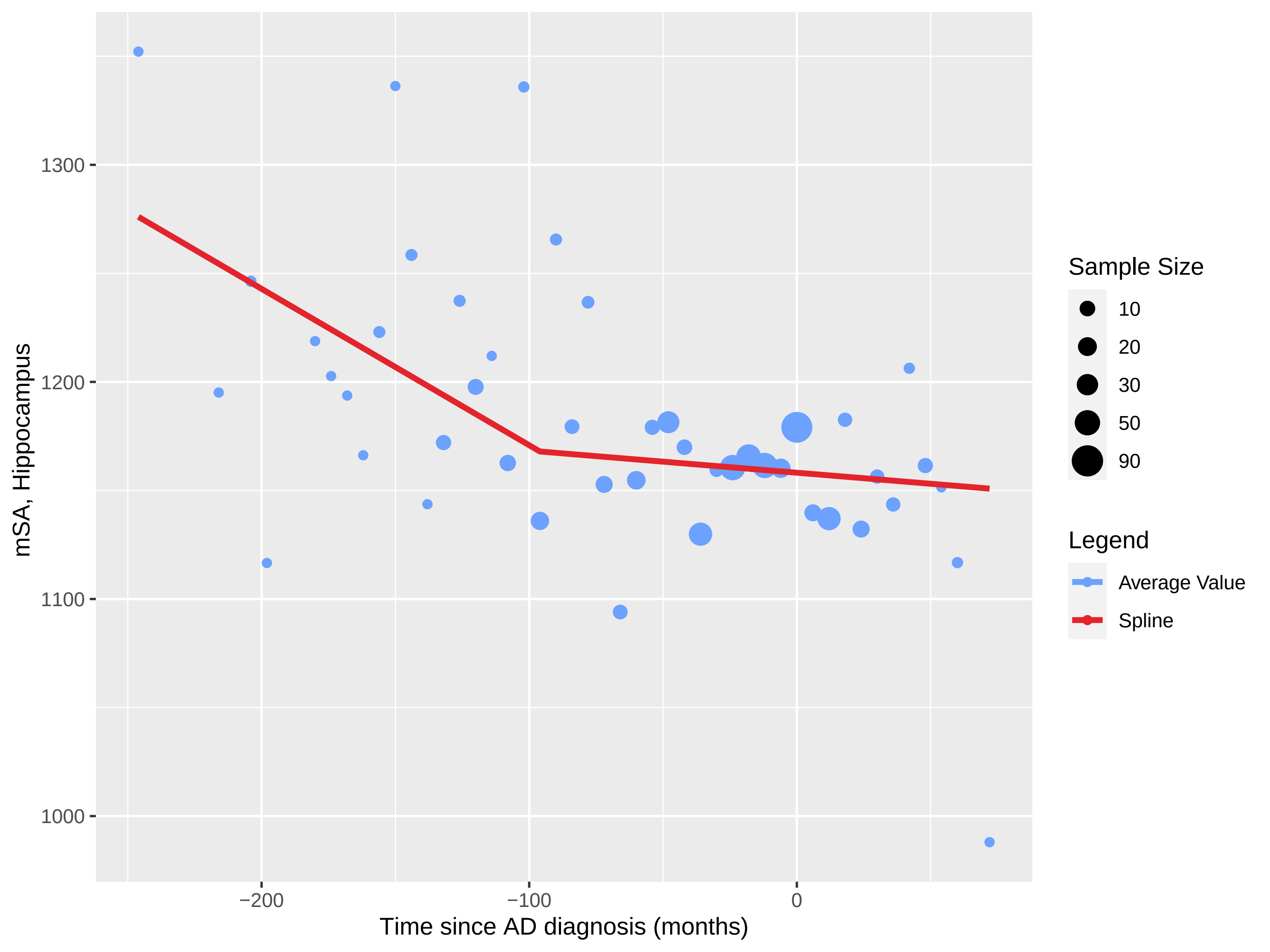

1300
1200
1100
1000
−200
−100
0
Time since AD diagnosis (months)
mSA, Hippocampus
Sample Size
10
20
30
50
90
Legend
Average Value
Spline

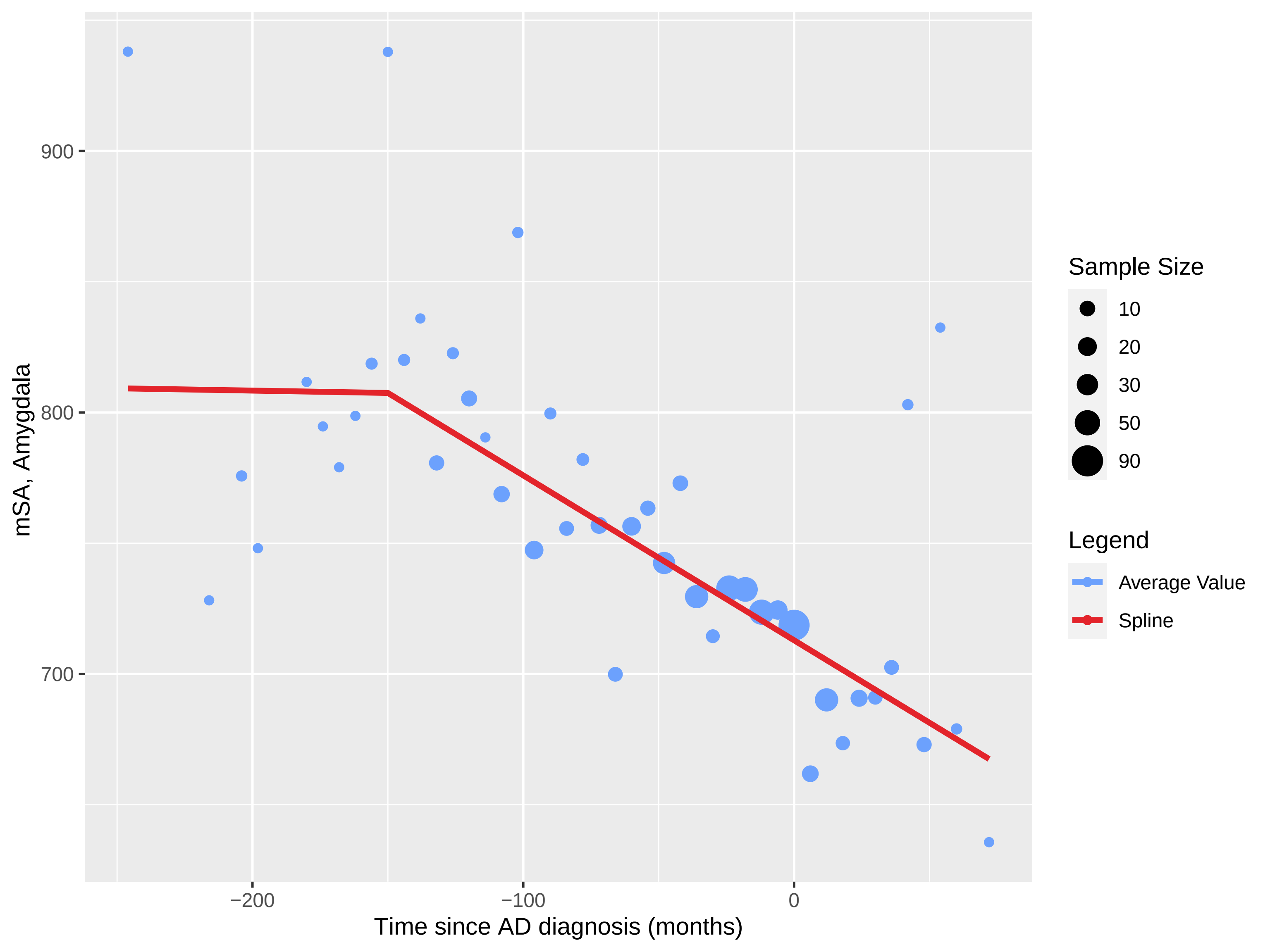

mSA, Amygdala
900
800
700
−200
−100
0
Time since AD diagnosis (months)
Sample Size
10
20
30
50
90
Legend
Average Value
Spline

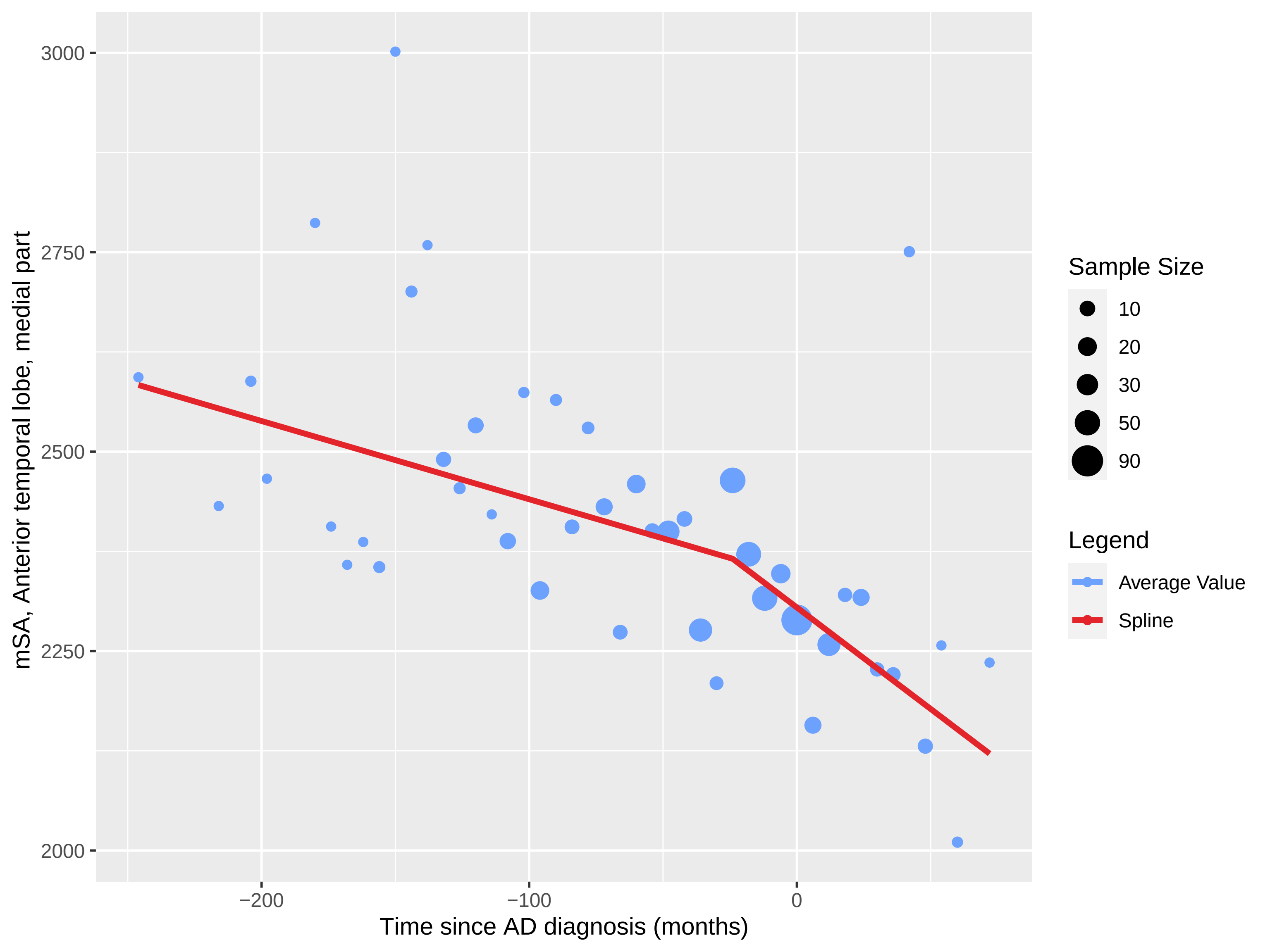

mSA, Anterior temporal lobe, medial part
3000
2750
2500
2250
2000
−200
−100
0
Time since AD diagnosis (months)
Sample Size
10
20
30
50
90
Legend
Average Value
Spline

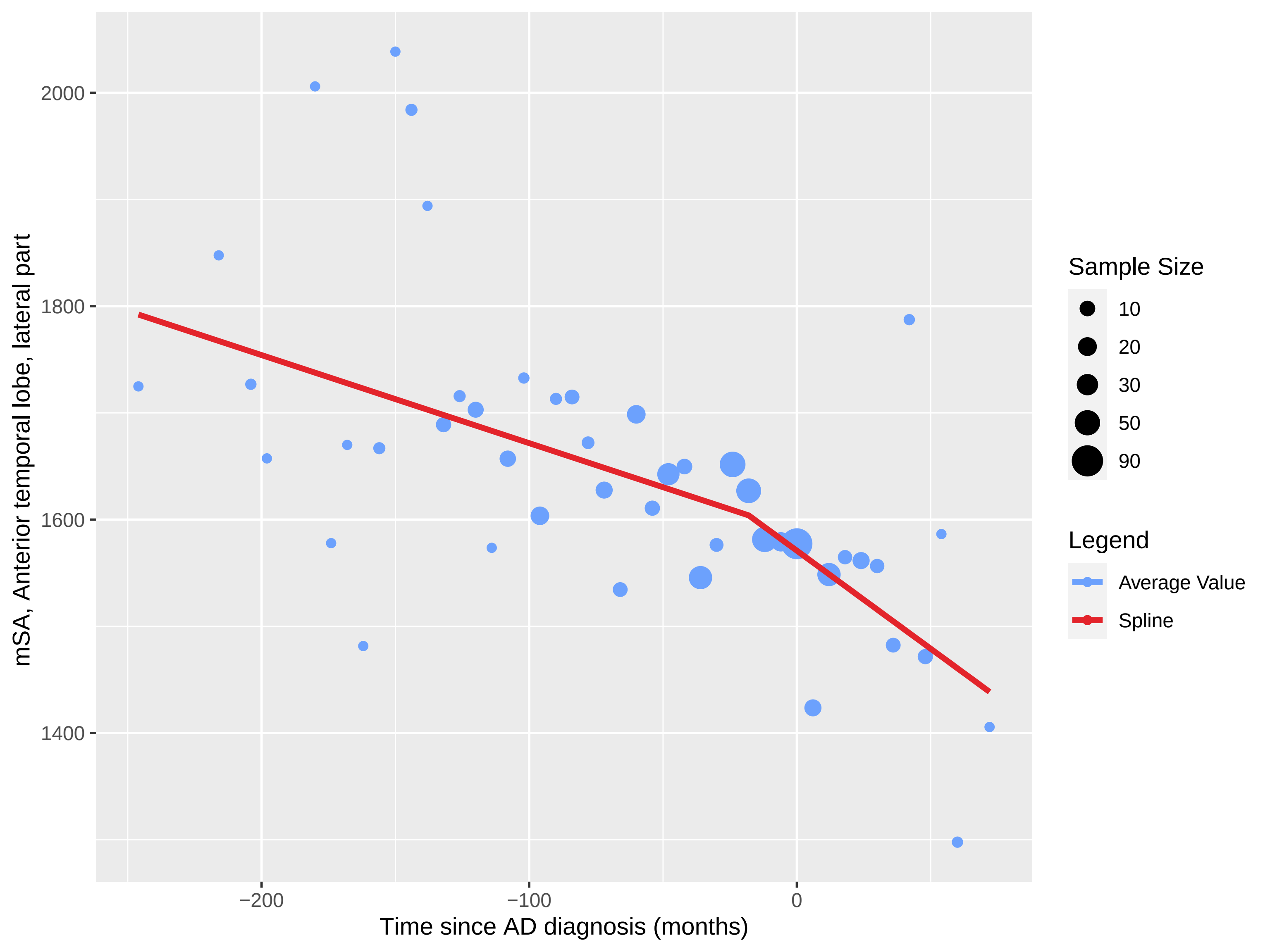

2000
1800
1600
1400
−200
−100
0
mSA, Anterior temporal lobe, lateral part
Time since AD diagnosis (months)
Sample Size
10
20
30
50
90
Legend
Average Value
Spline

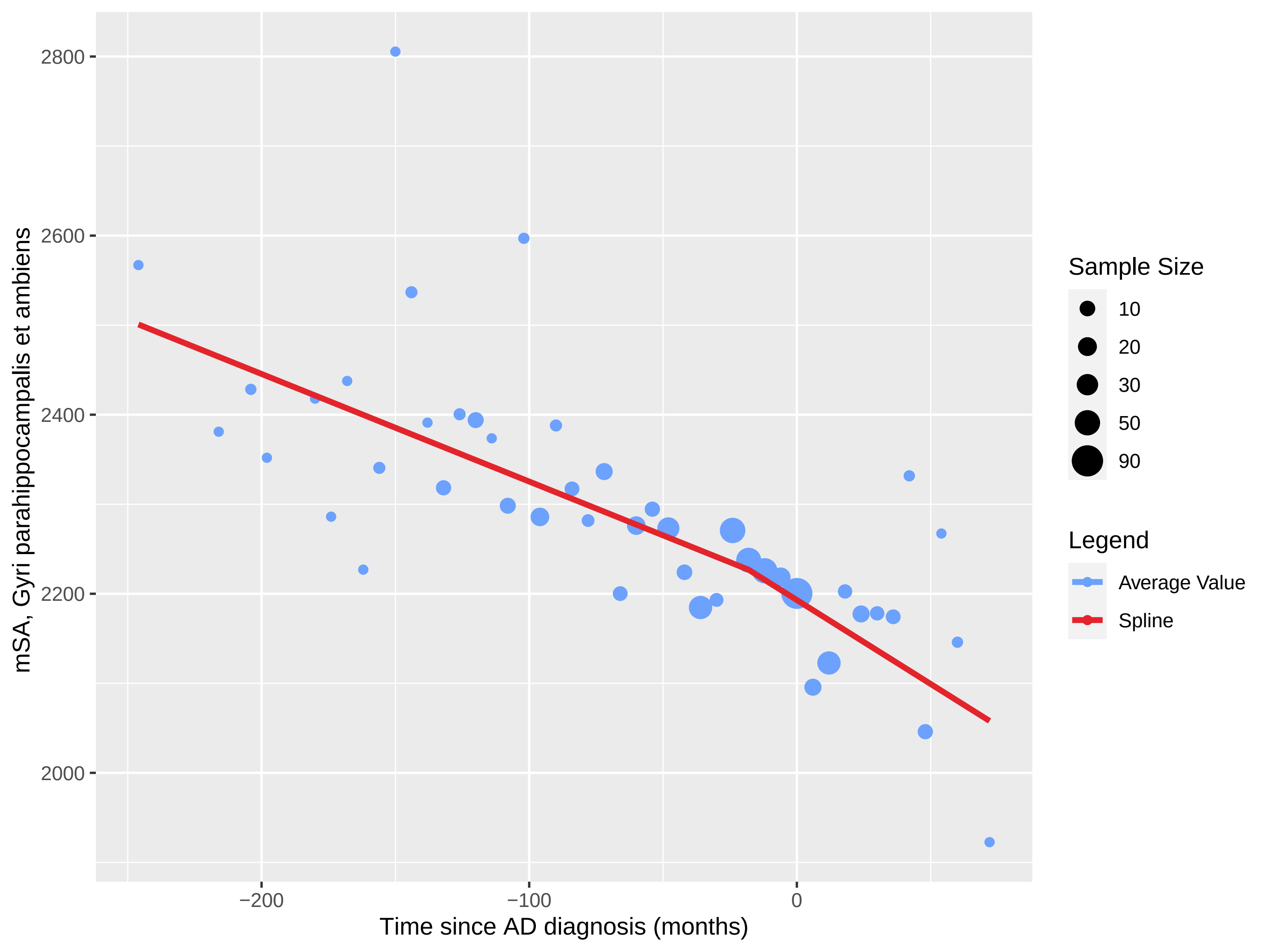

mSA, Gyri parahippocampalis et ambiens
2800
2600
2400
2200
2000
−200
−100
0
Time since AD diagnosis (months)
Sample Size
10
20
30
50
90
Legend
Average Value
Spline

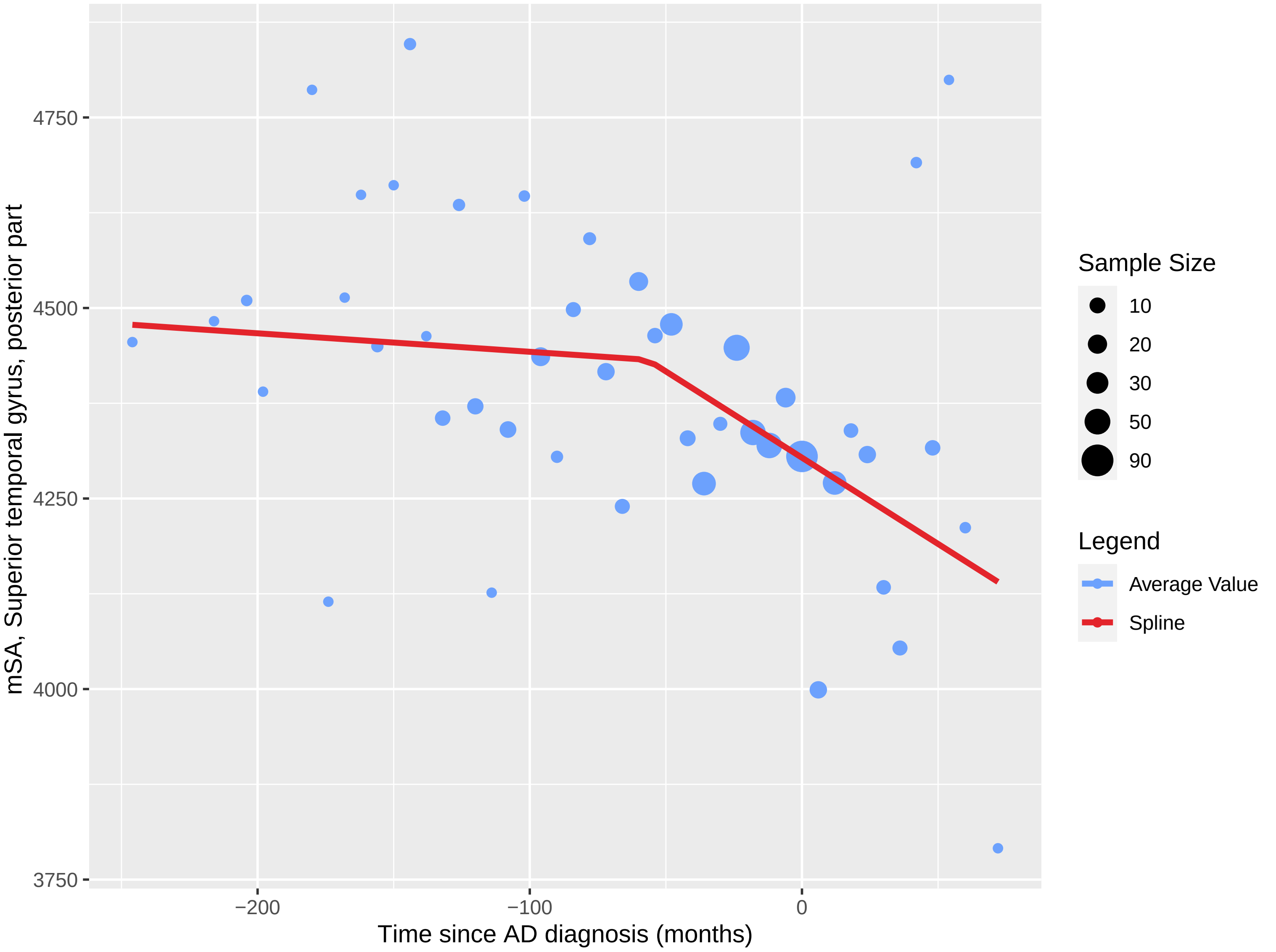

mSA, Superior temporal gyrus, posterior part
4750
4500
4250
4000
3750
−200
−100
0
Time since AD diagnosis (months)
Sample Size
10
20
30
50
90
Legend
Average Value
Spline

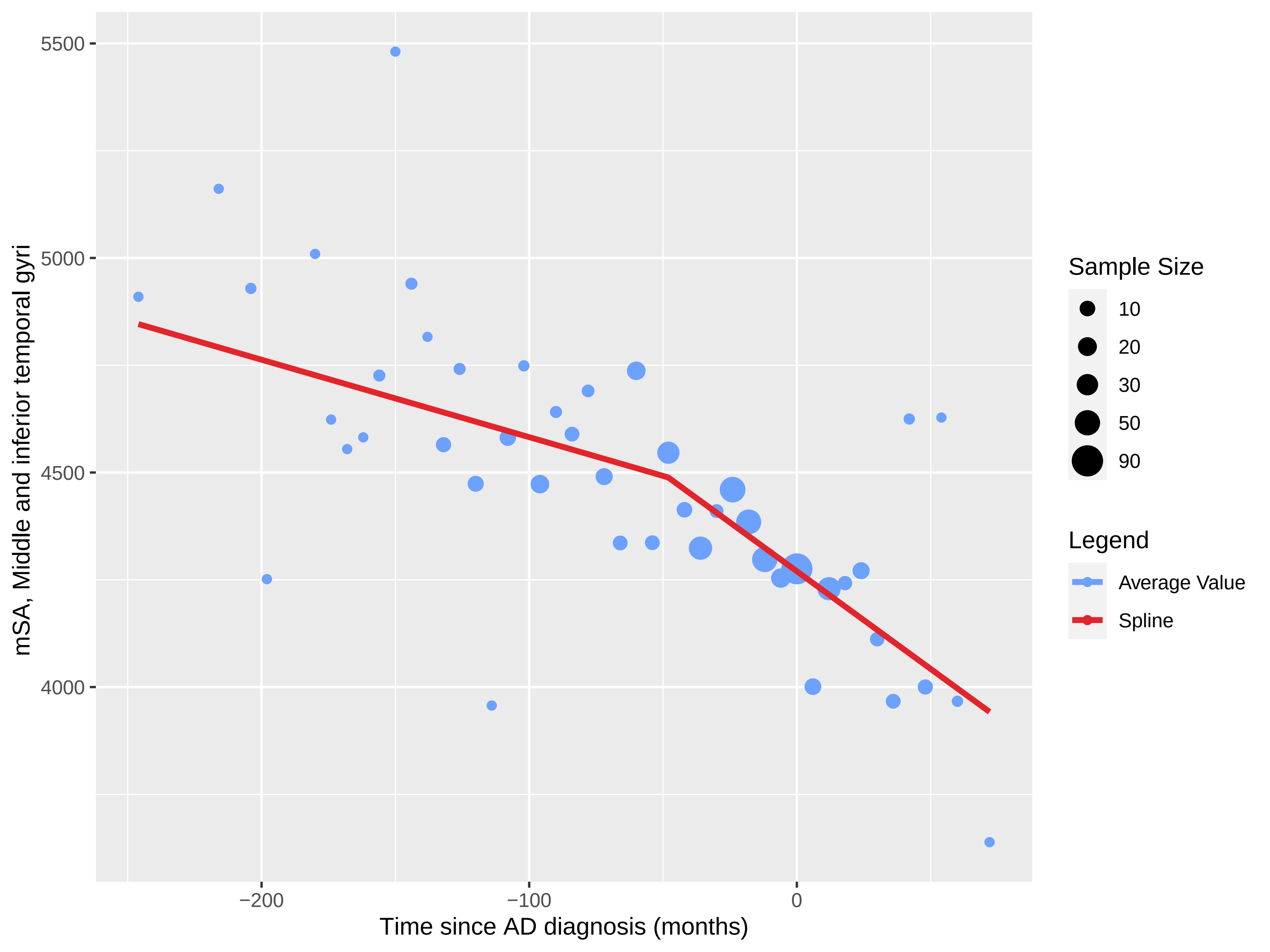

mSA, Middle and inferior temporal gyri
5500
5000
4500
4000
−200
−100
0
Time since AD diagnosis (months)
Sample Size
10
20
30
50
90
Legend
Average Value
Spline

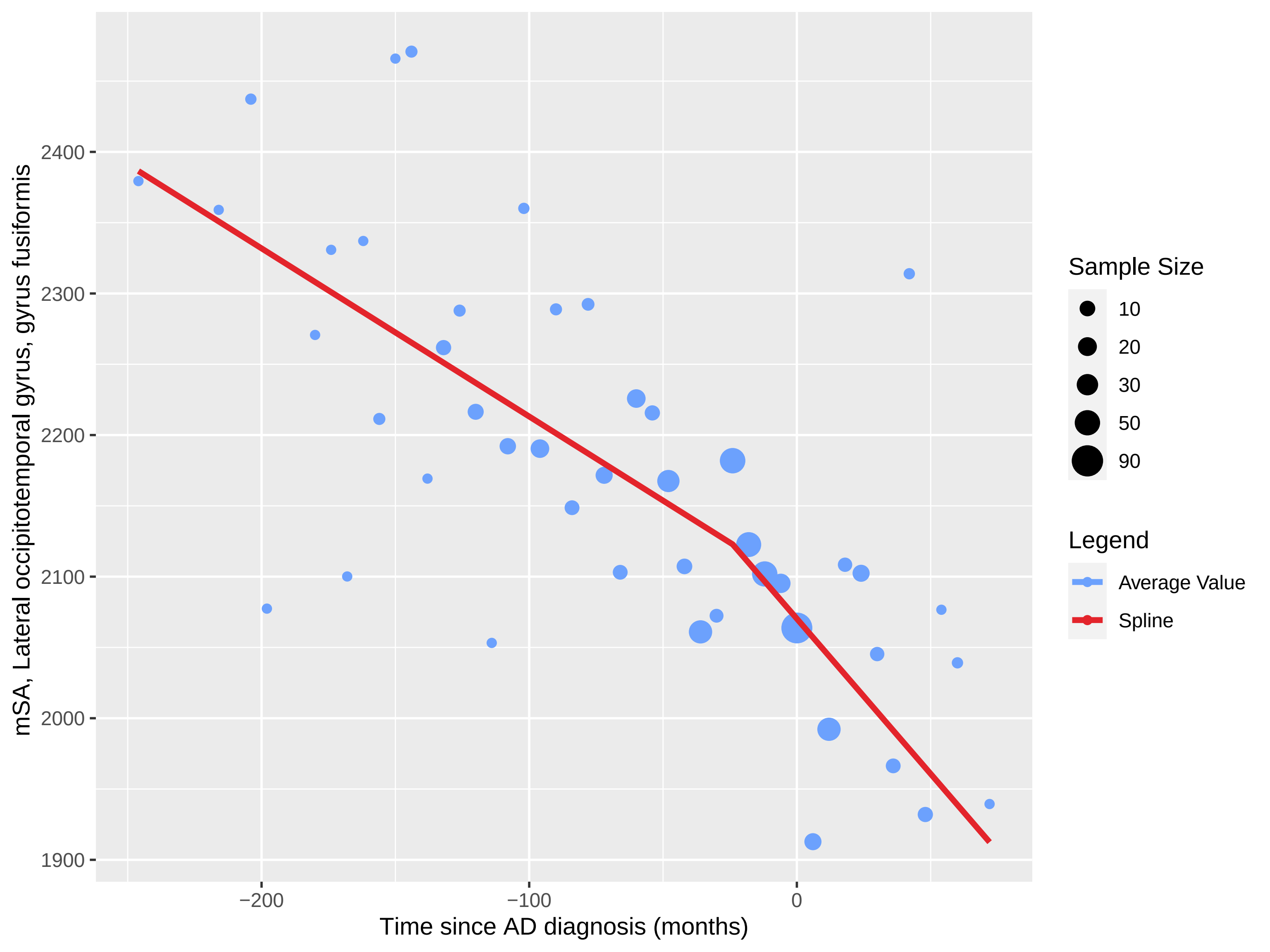

mSA, Lateral occipitotemporal gyrus, gyrus fusiformis
2400
2300
2200
2100
2000
1900
−200
−100
0
Time since AD diagnosis (months)
Sample Size
10
20
30
50
90
Legend
Average Value
Spline

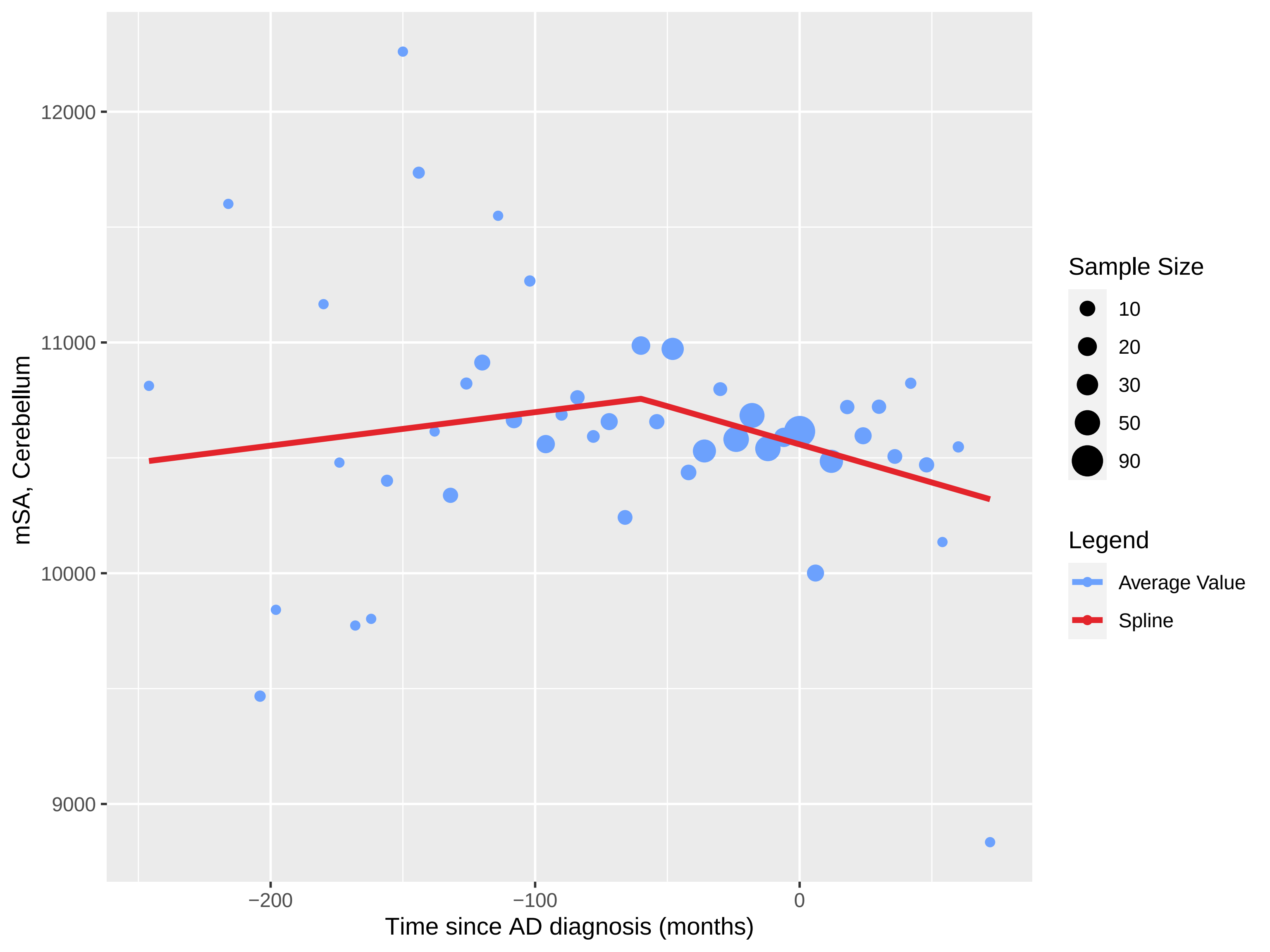

12000
11000
10000
9000
mSA, Cerebellum
−200
−100
0
Time since AD diagnosis (months)
Sample Size
10
20
30
50
90
Legend
Average Value
Spline

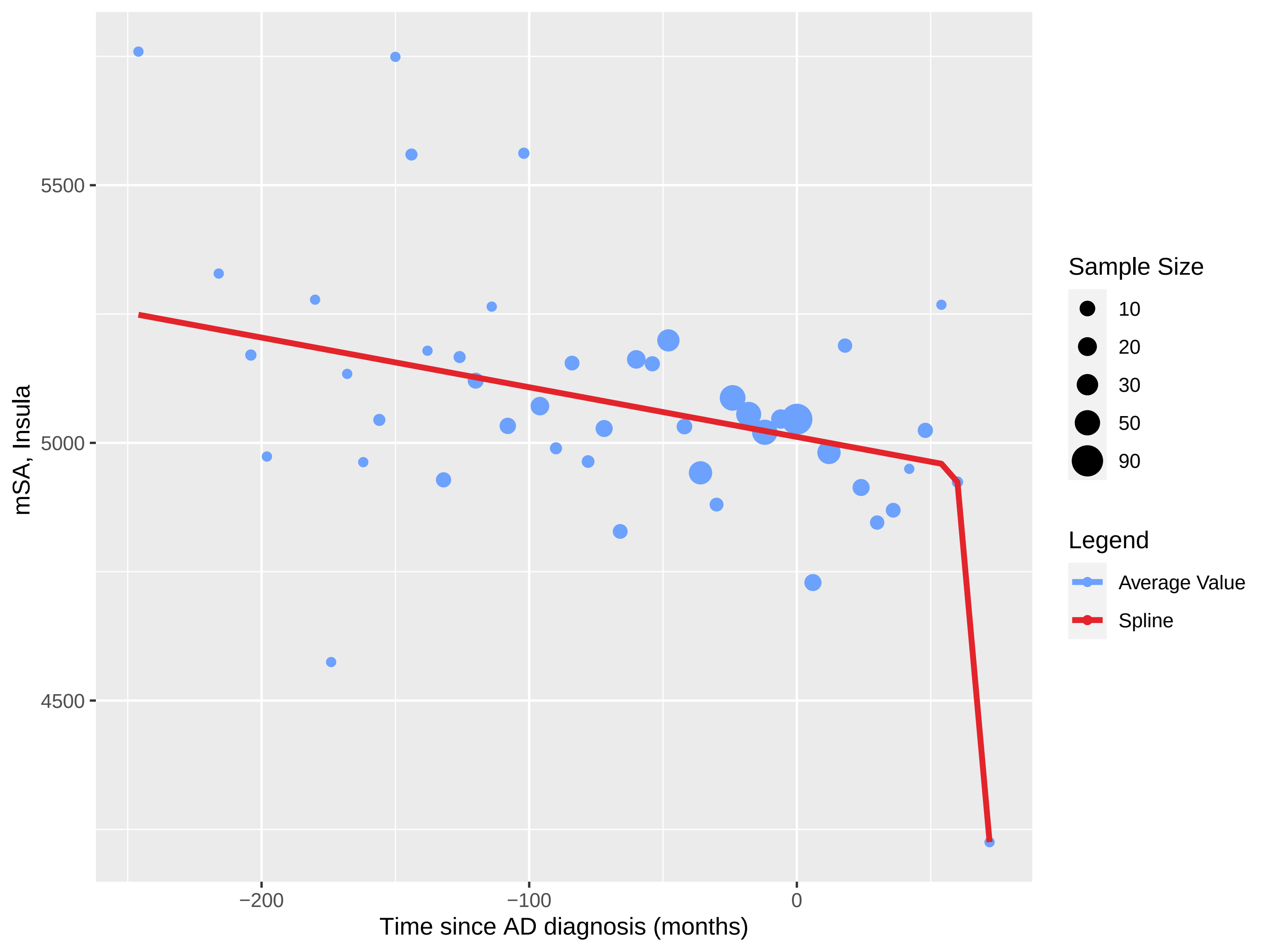

5500
5000
4500
−200
−100
0
mSA, Insula
Time since AD diagnosis (months)
Sample Size
10
20
30
50
90
Legend
Average Value
Spline

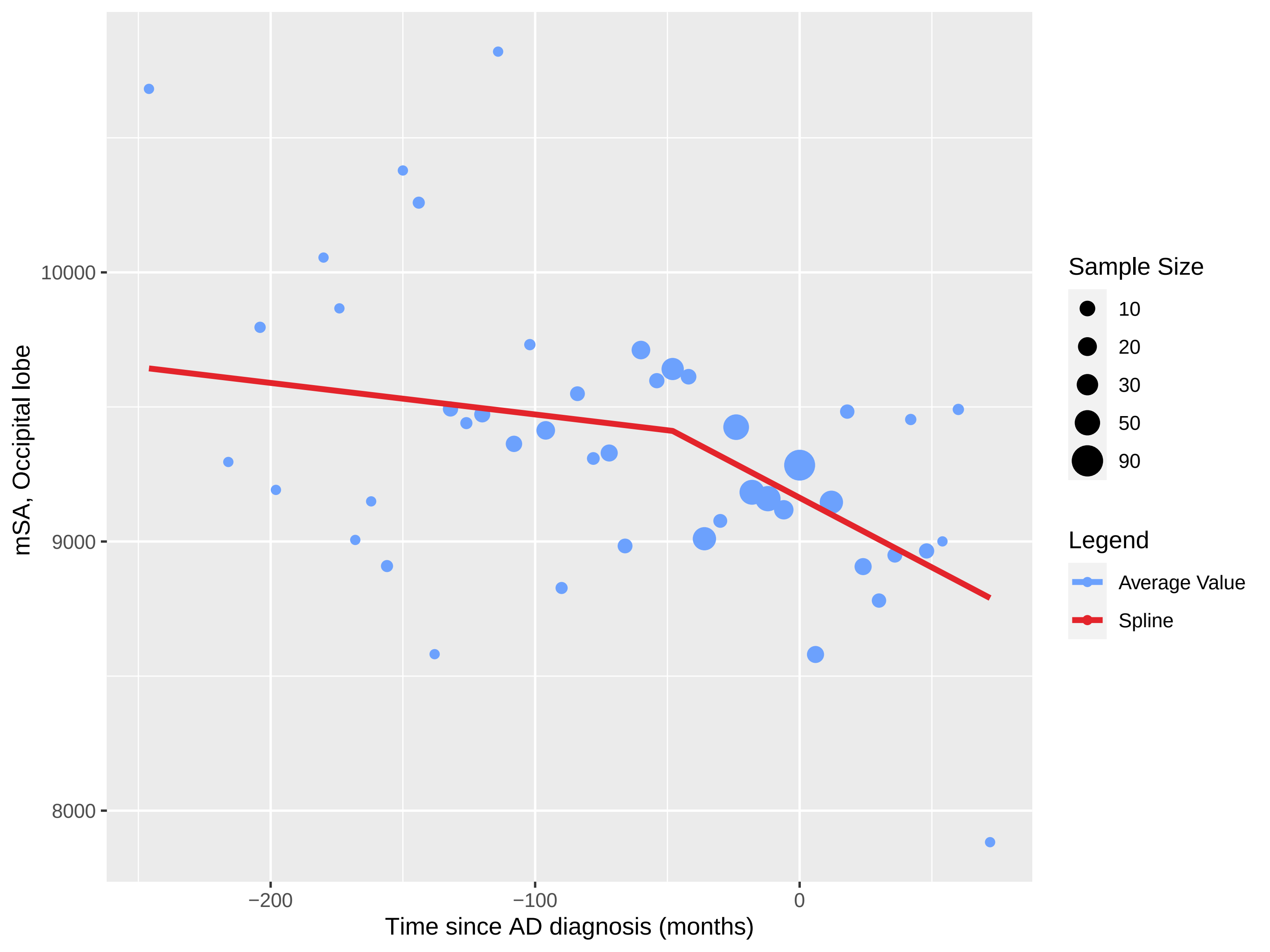

mSA, Occipital lobe
10000
9000
8000
−200
−100
0
Time since AD diagnosis (months)
Sample Size
10
20
30
50
90
Legend
Average Value
Spline

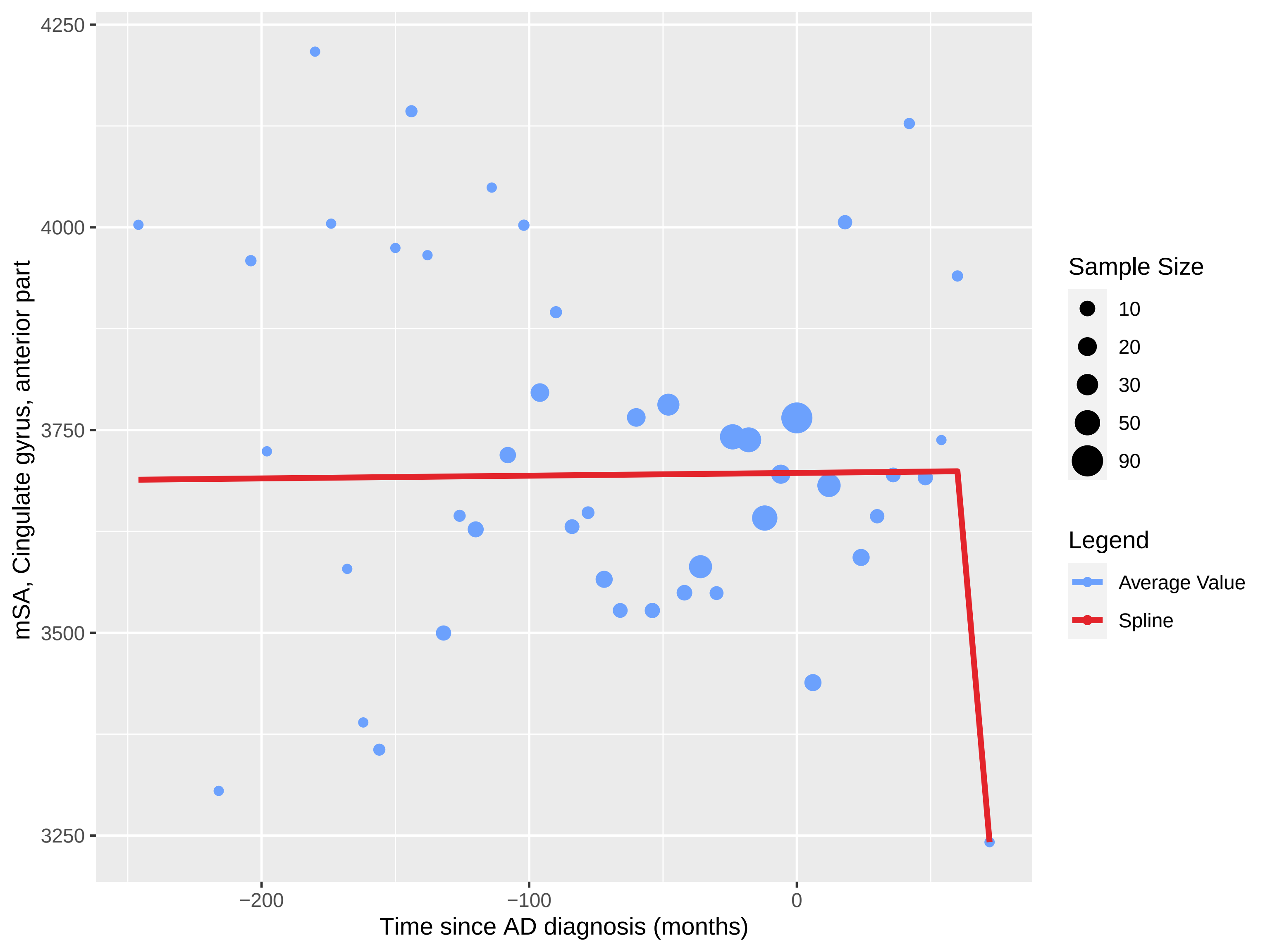

4250
4000
3750
3500
3250
mSA, Cingulate gyrus, anterior part
−200
−100
0
Time since AD diagnosis (months)
Sample Size
10
20
30
50
90
Legend
Average Value
Spline

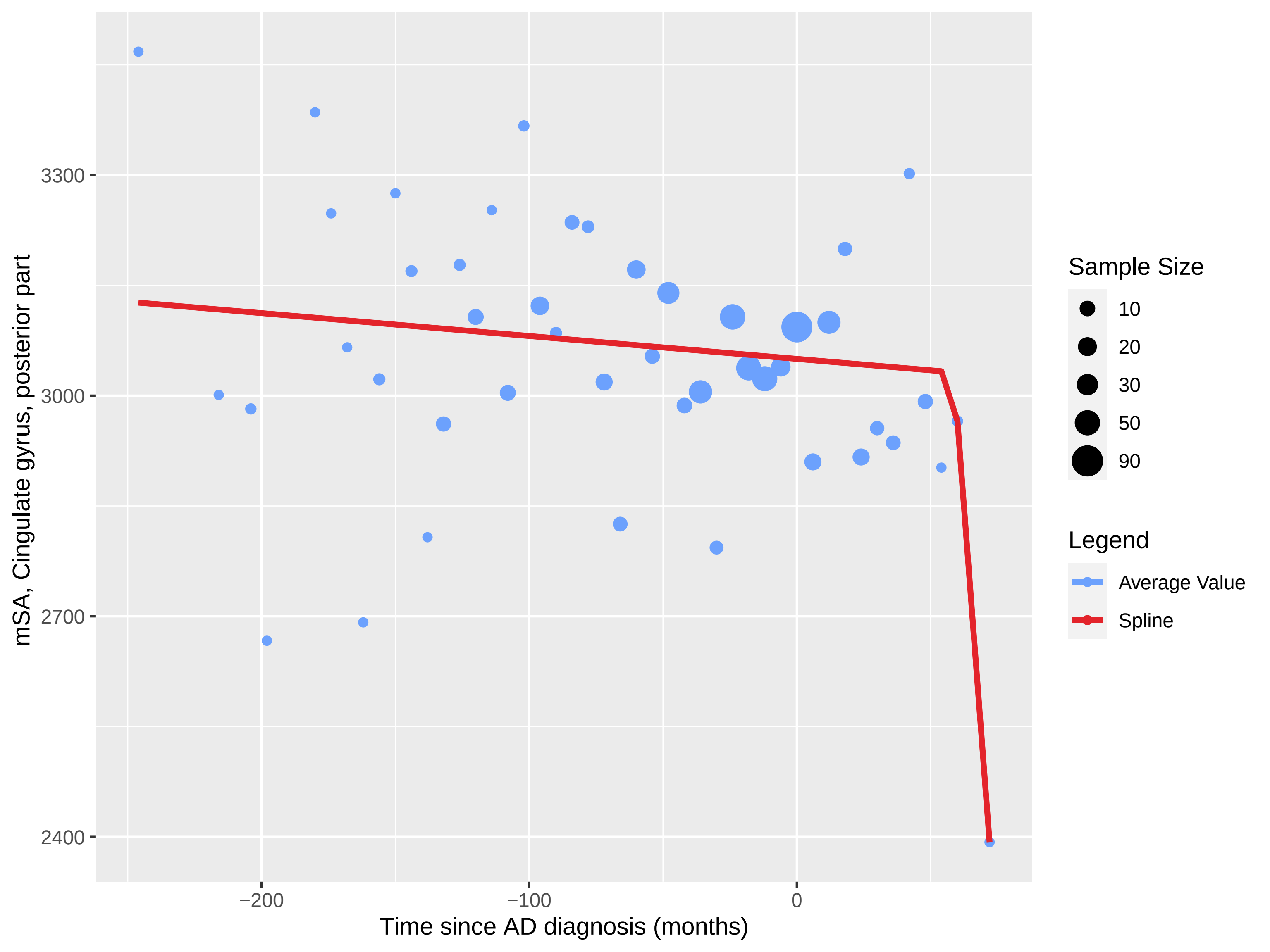

mSA, Cingulate gyrus, posterior part
3300
3000
2700
2400
−200
−100
0
Time since AD diagnosis (months)
Sample Size
10
20
30
50
90
Legend
Average Value
Spline

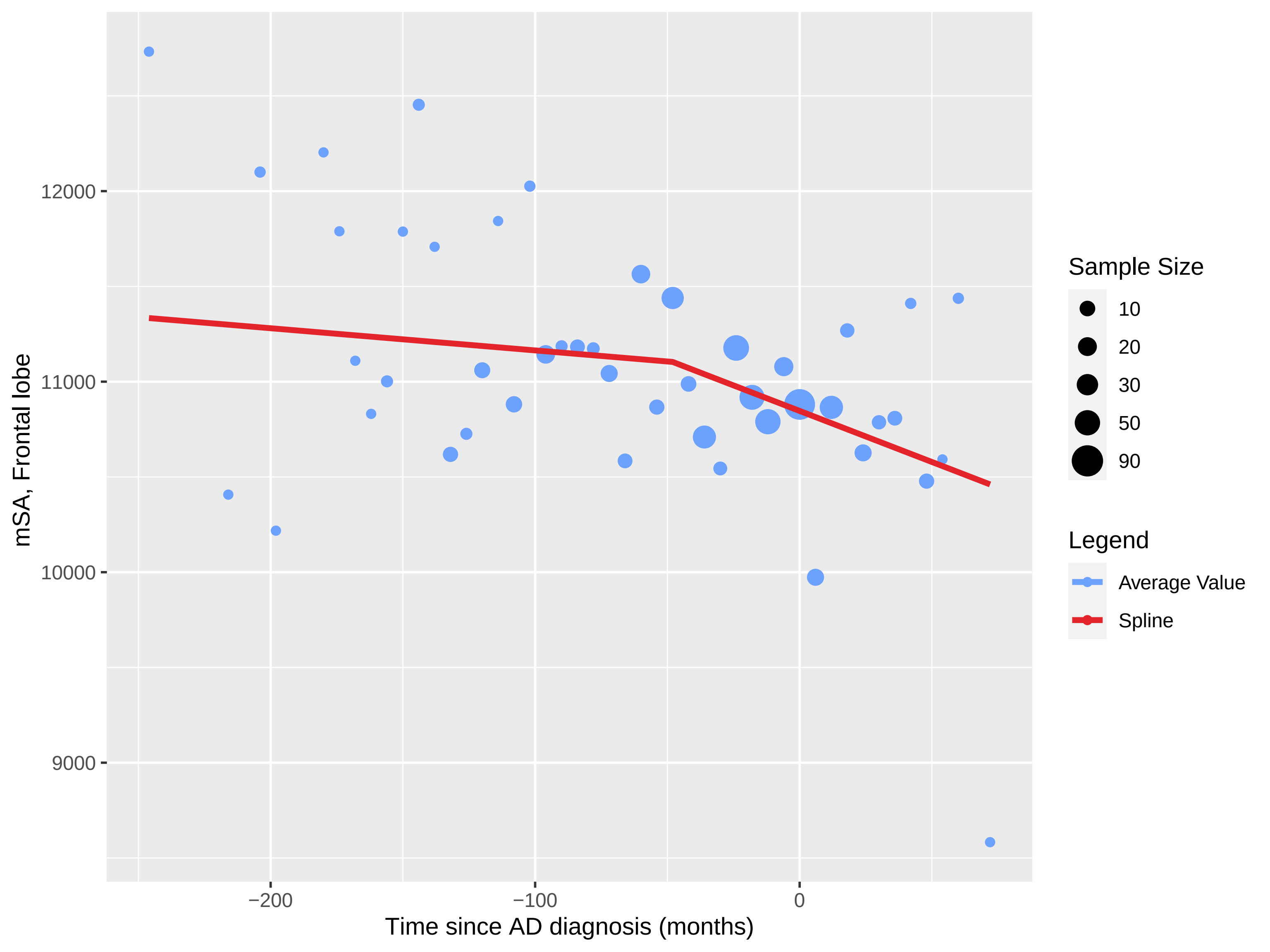

mSA, Frontal lobe
12000
11000
10000
9000
−200
−100
0
Time since AD diagnosis (months)
Sample Size
10
20
30
50
90
Legend
Average Value
Spline

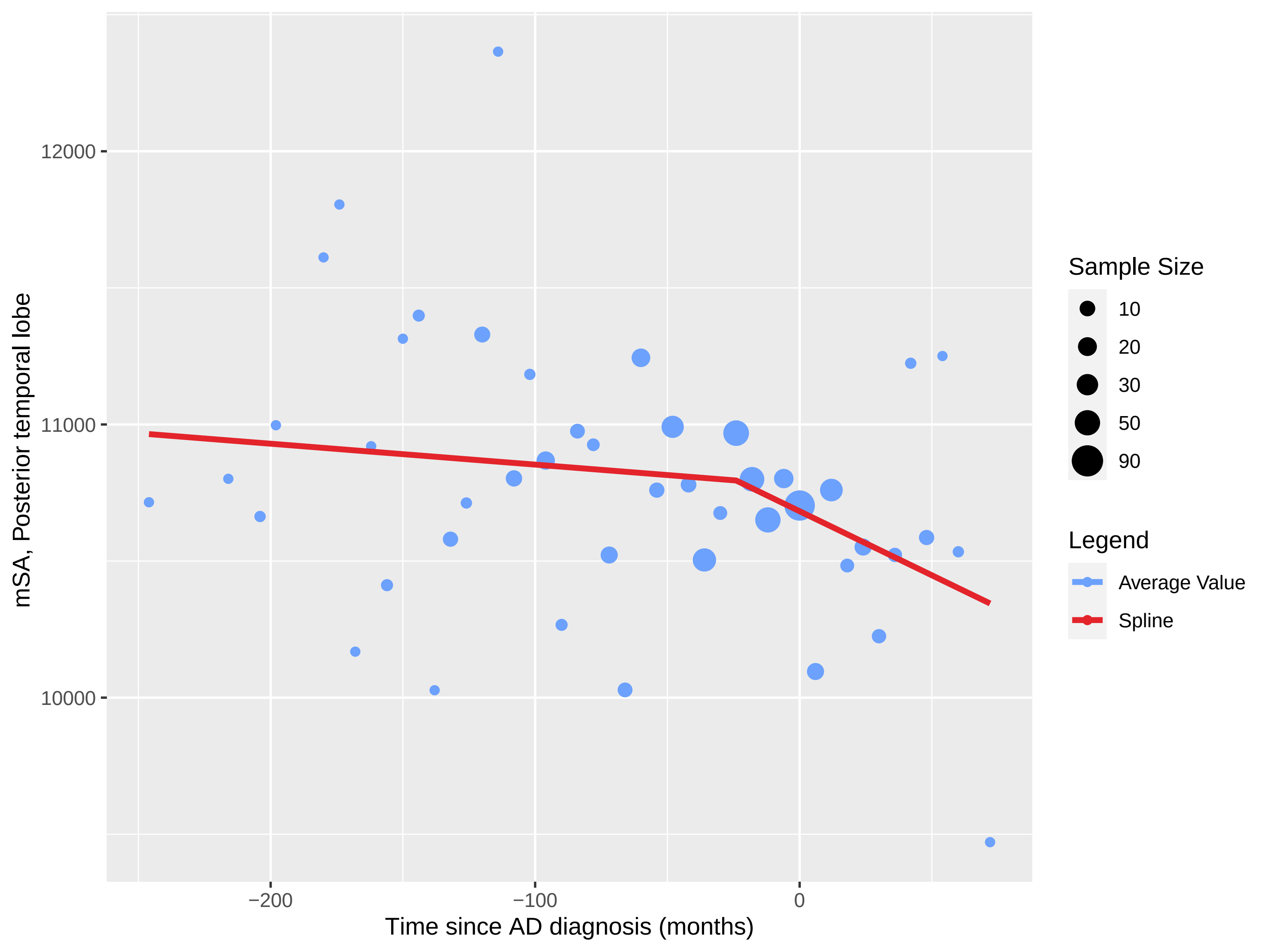

12000
11000
10000
mSA, Posterior temporal lobe
−200
−100
0
Time since AD diagnosis (months)
Sample Size
10
20
30
50
90
Legend
Average Value
Spline

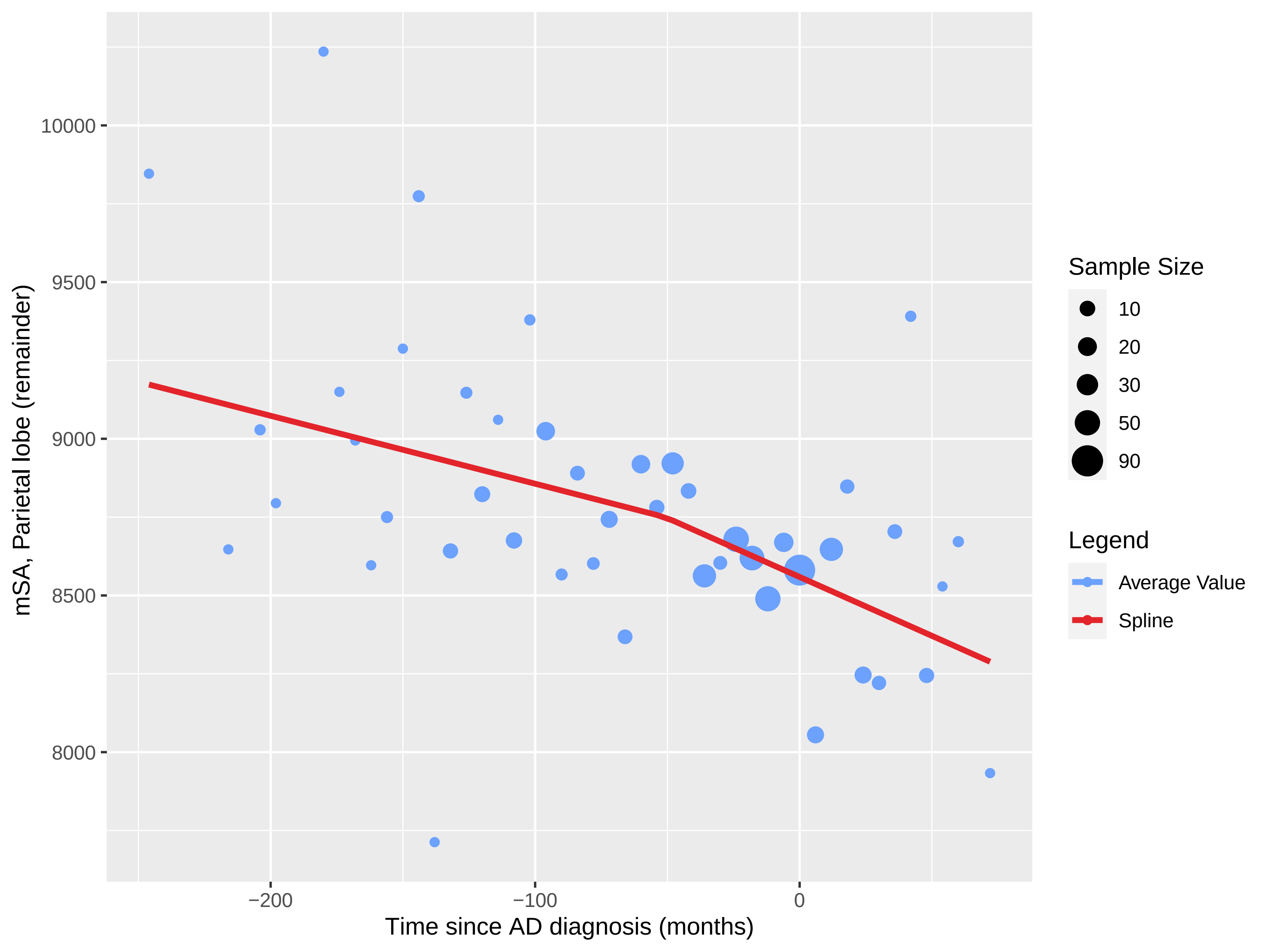

mSA, Parietal lobe (remainder)
10000
9500
9000
8500
8000
−200
−100
0
Time since AD diagnosis (months)
Sample Size
10
20
30
50
90
Legend
Average Value
Spline

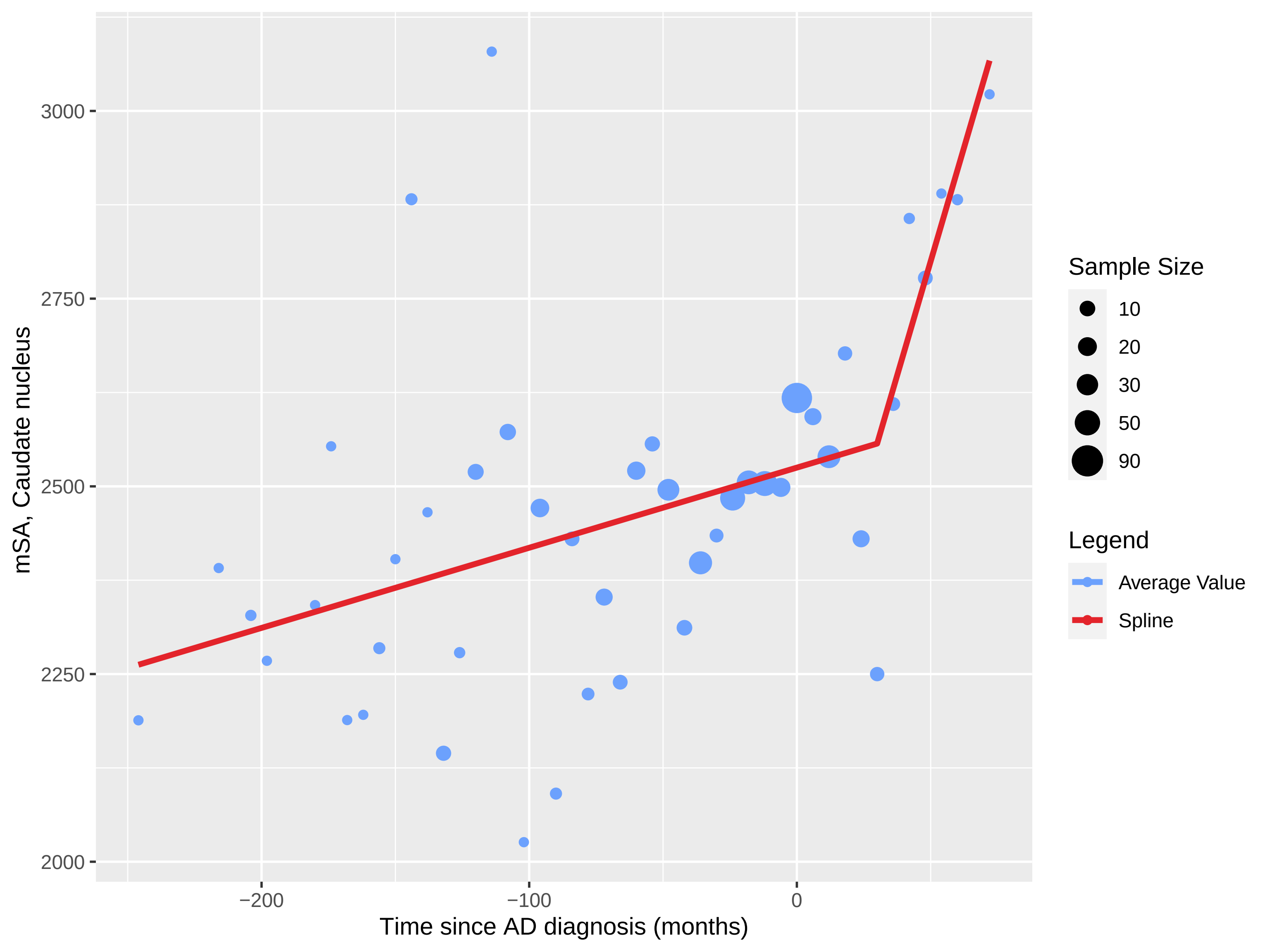

mSA, Caudate nucleus
3000
2750
2500
2250
2000
−200
−100
0
Time since AD diagnosis (months)
Sample Size
10
20
30
50
90
Legend
Average Value
Spline

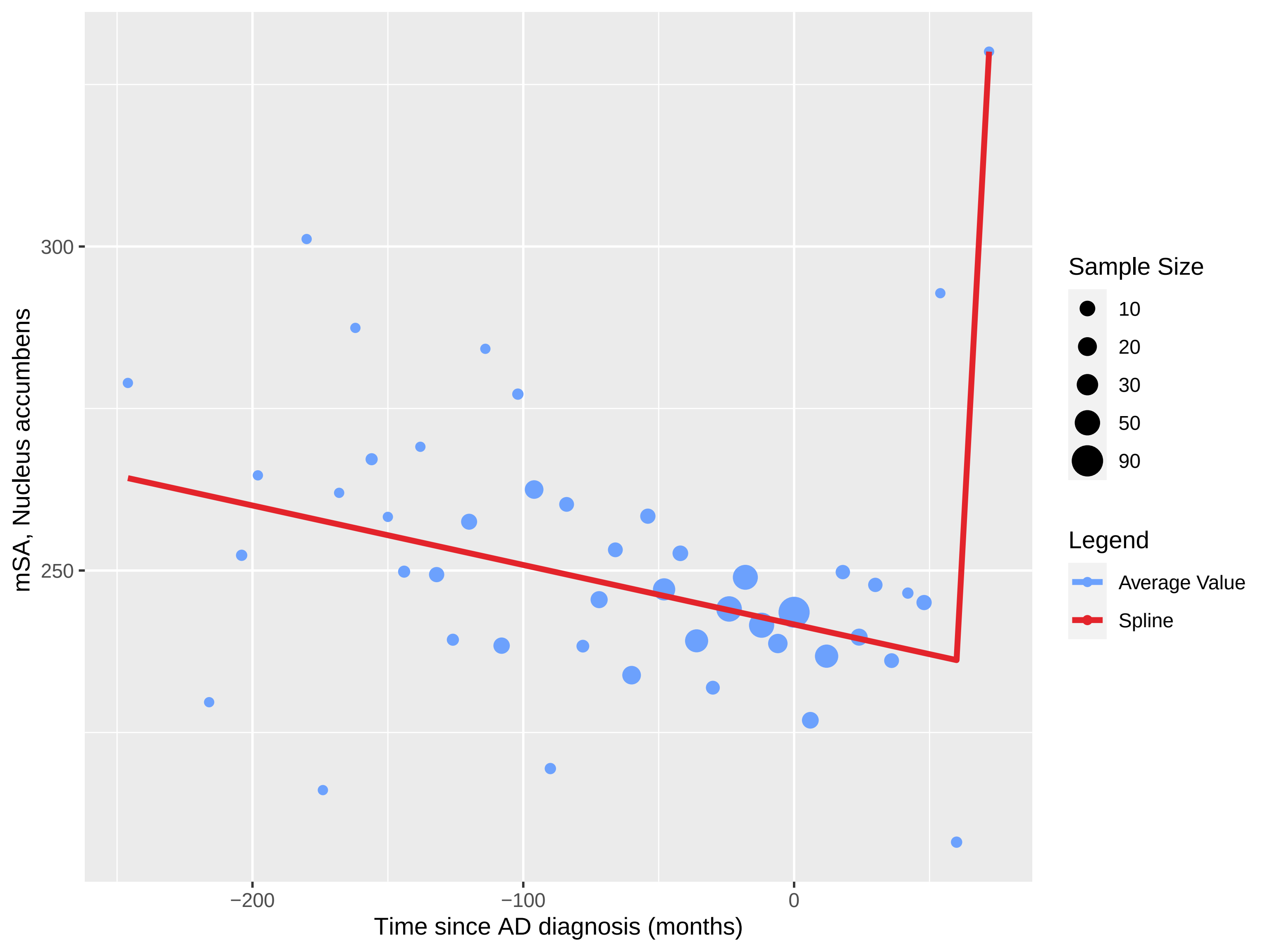

300
250
−200
−100
0
Time since AD diagnosis (months)
mSA, Nucleus accumbens
Sample Size
10
20
30
50
90
Legend
Average Value
Spline

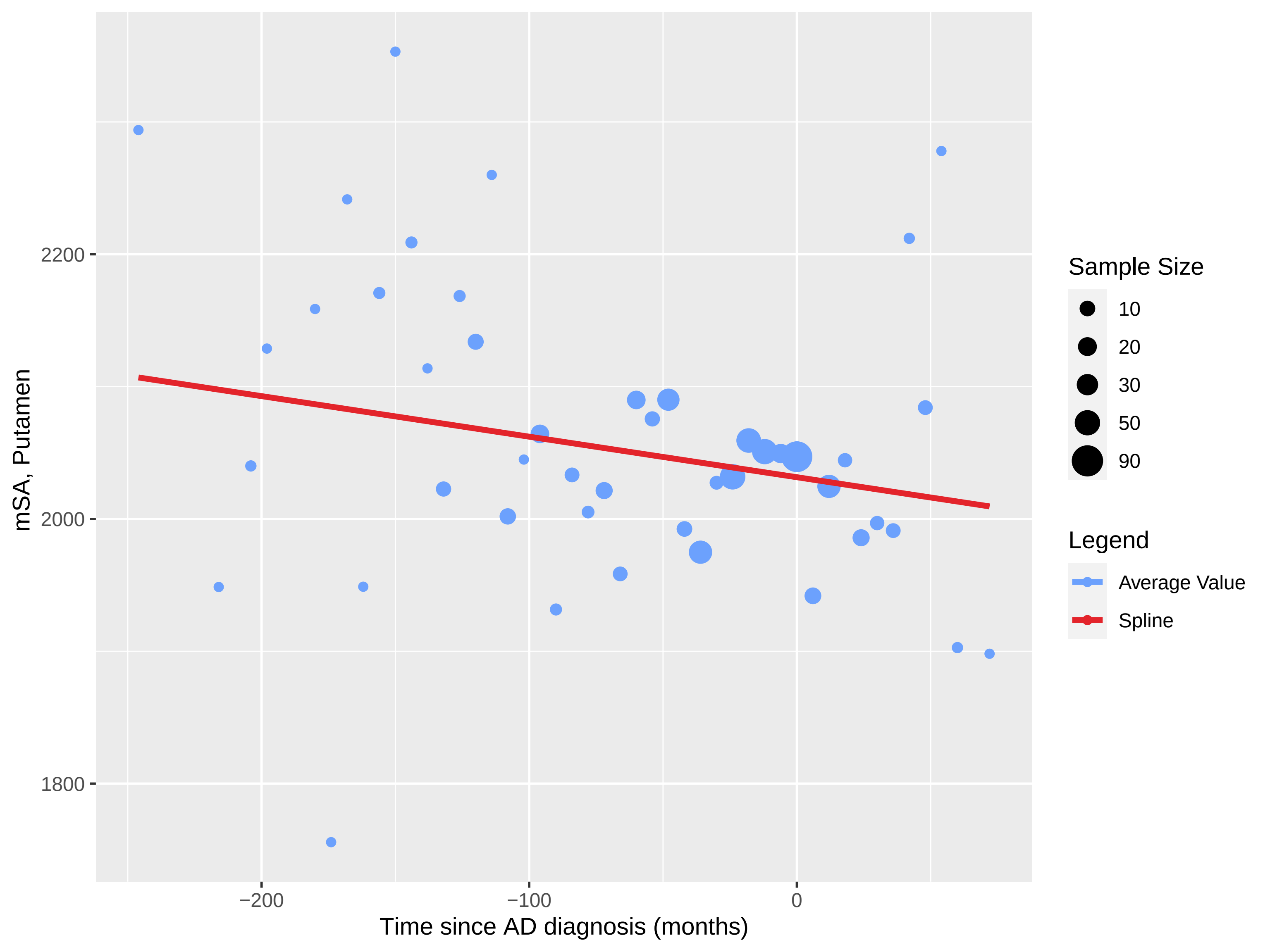

2200
2000
1800
mSA, Putamen
−200
−100
0
Time since AD diagnosis (months)
Sample Size
10
20
30
50
90
Legend
Average Value
Spline

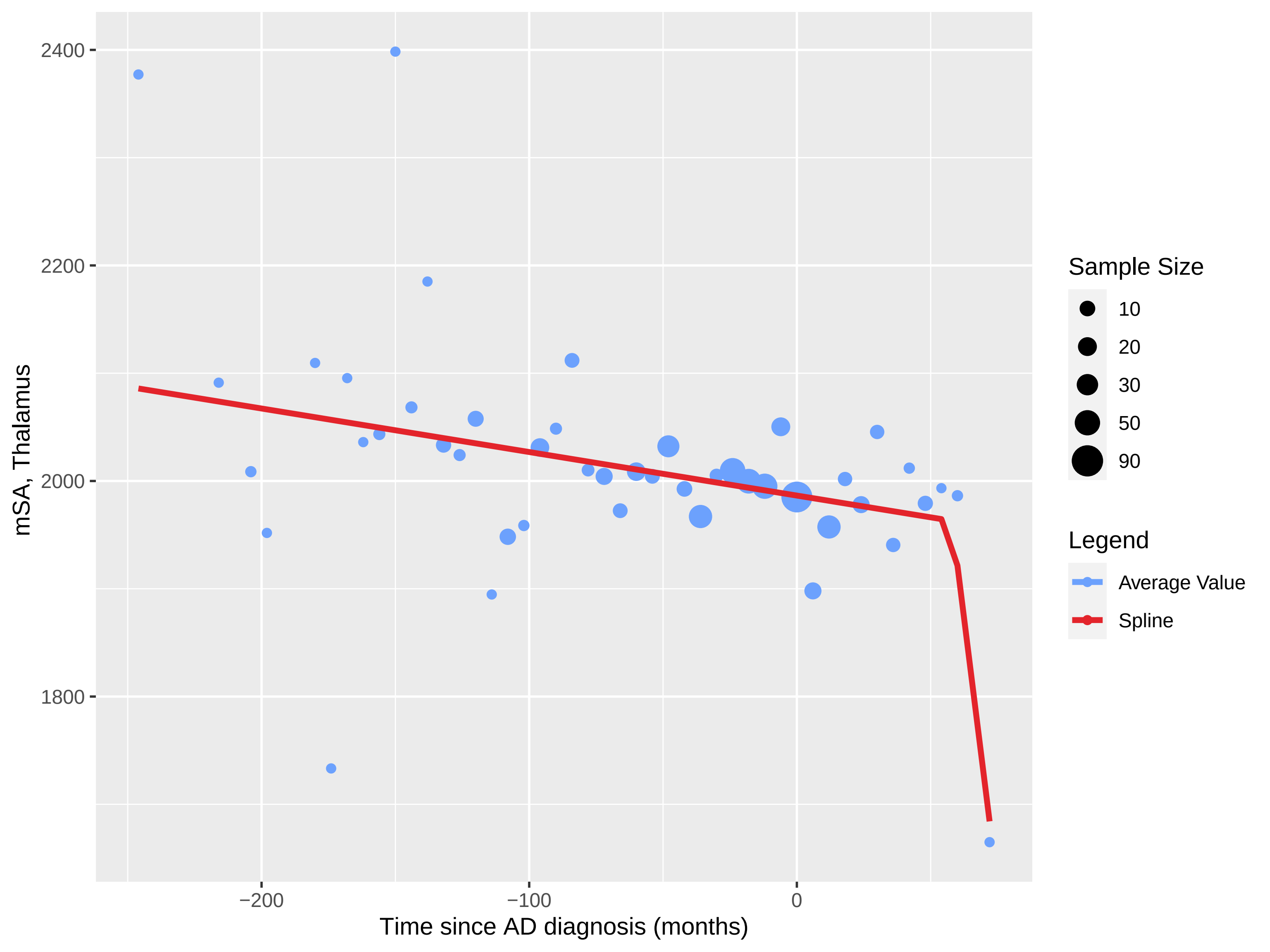

mSA, Thalamus
2400
2200
2000
1800
−200
−100
0
Time since AD diagnosis (months)
Sample Size
10
20
30
50
90
Legend
Average Value
Spline

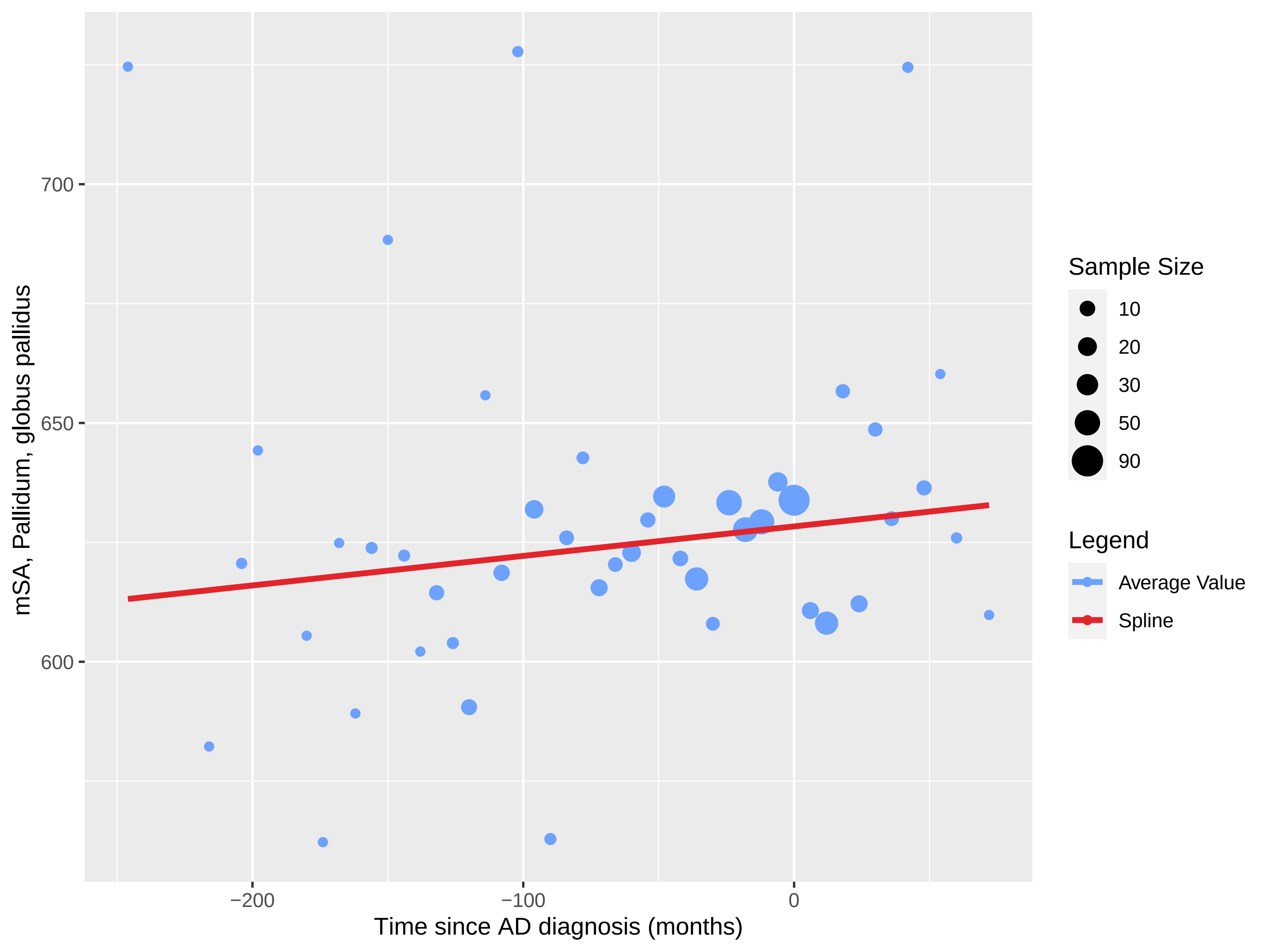

mSA, Pallidum, globus pallidus
700
650
600
−200
−100
0
Time since AD diagnosis (months)
Sample Size
10
20
30
50
90
Legend
Average Value
Spline

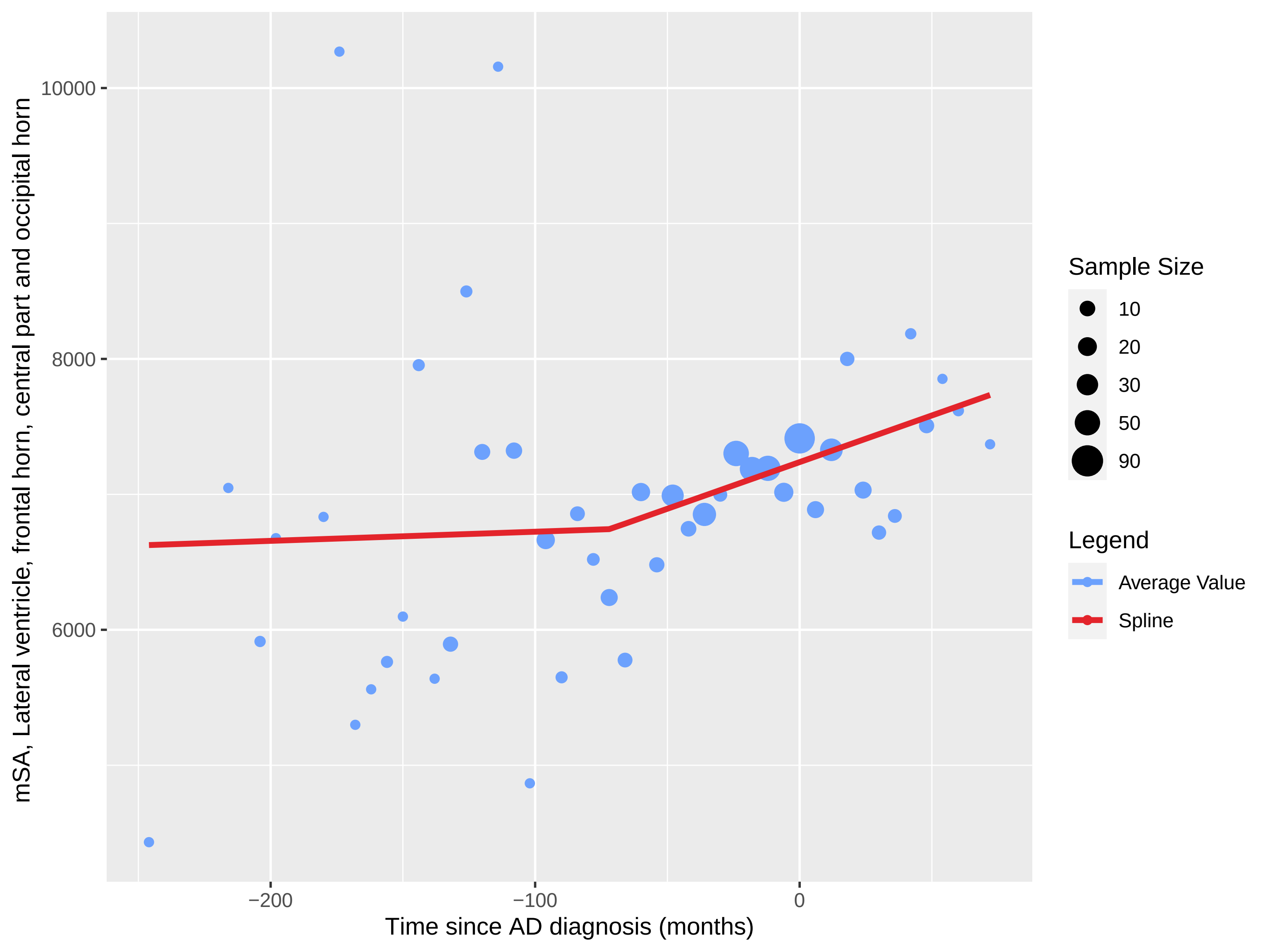

mSA, Lateral ventricle, frontal horn, central part and occipital horn
10000
8000
6000
−200
−100
0
Time since AD diagnosis (months)
Sample Size
10
20
30
50
90
Legend
Average Value
Spline

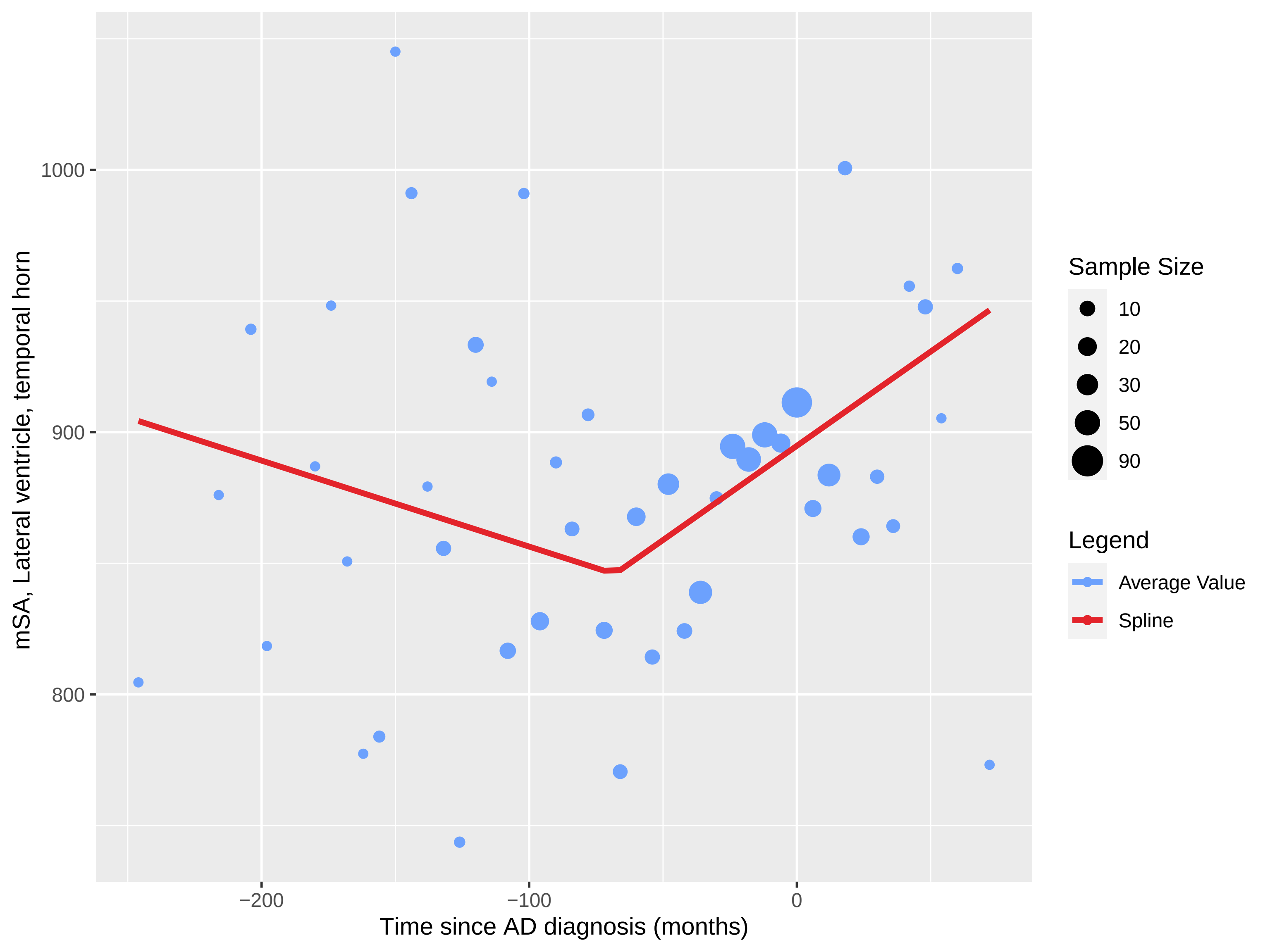

mSA, Lateral ventricle, temporal horn
1000
900
800
−200
−100
0
Time since AD diagnosis (months)
Sample Size
10
20
30
50
90
Legend
Average Value
Spline

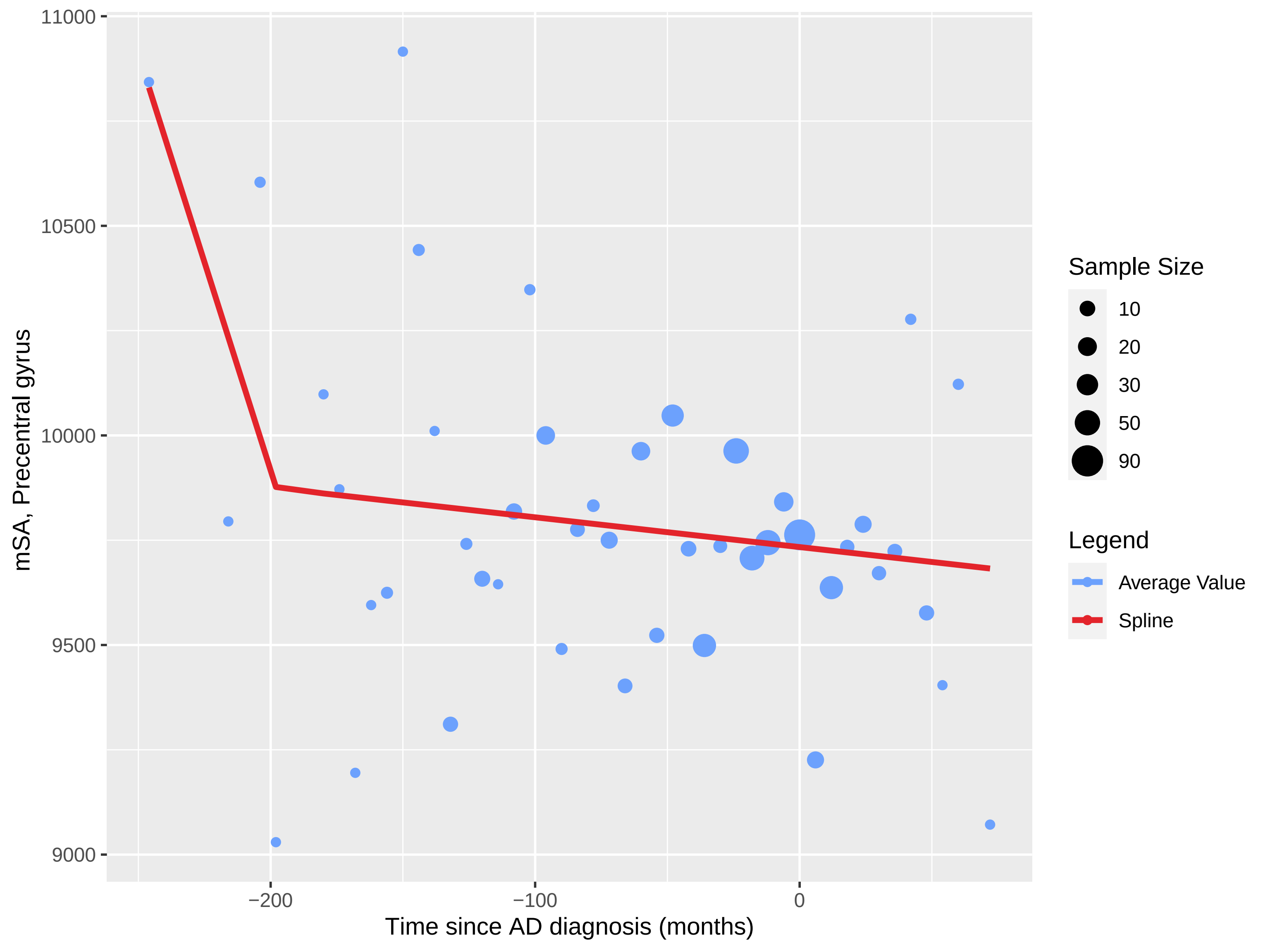

11000
10500
10000
9500
9000
−200
−100
0
Time since AD diagnosis (months)
mSA, Precentral gyrus
Sample Size
10
20
30
50
90
Legend
Average Value
Spline

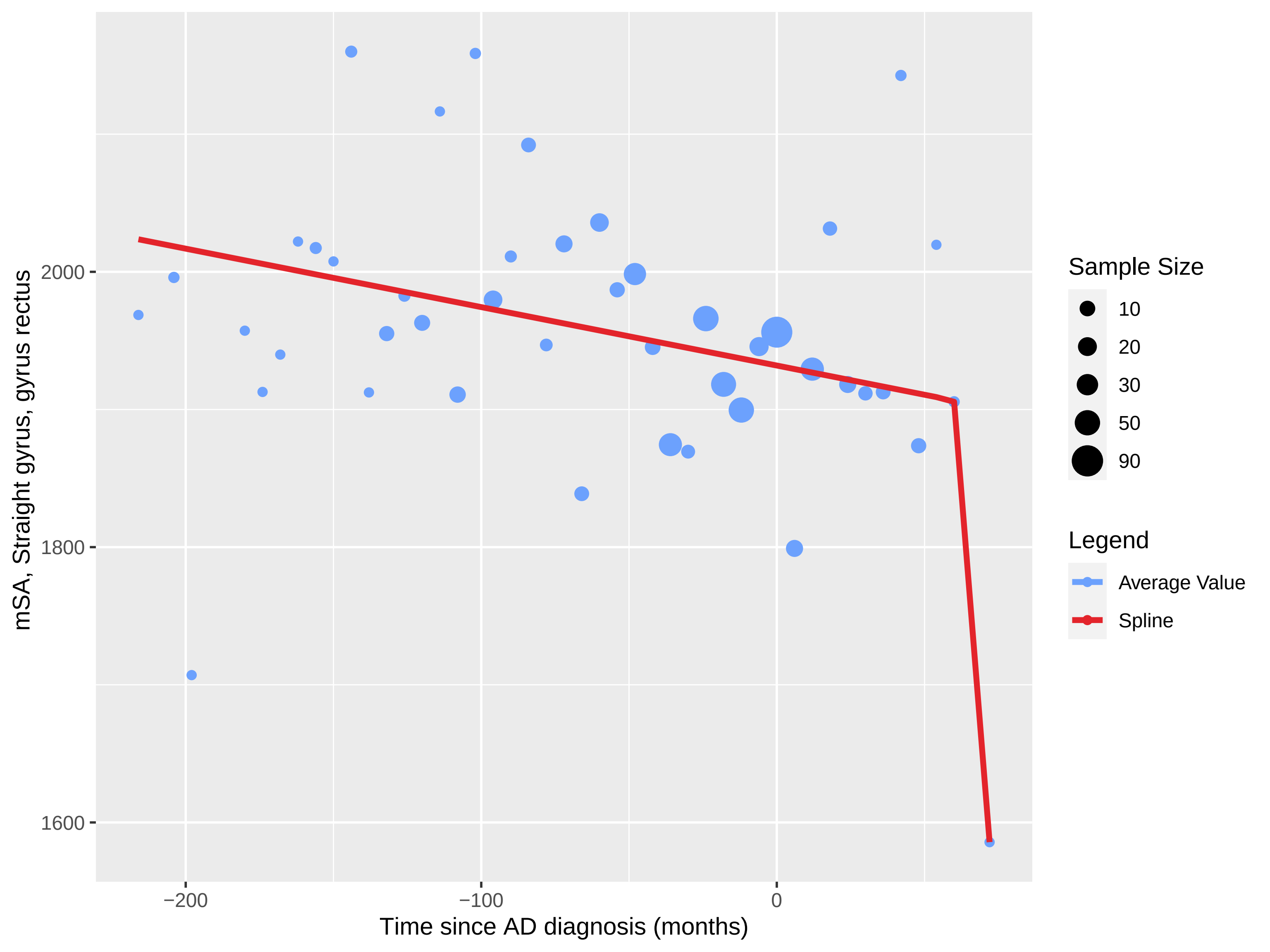

mSA, Straight gyrus, gyrus rectus
2000
1800
1600
−200
−100
0
Time since AD diagnosis (months)
Sample Size
10
20
30
50
90
Legend
Average Value
Spline

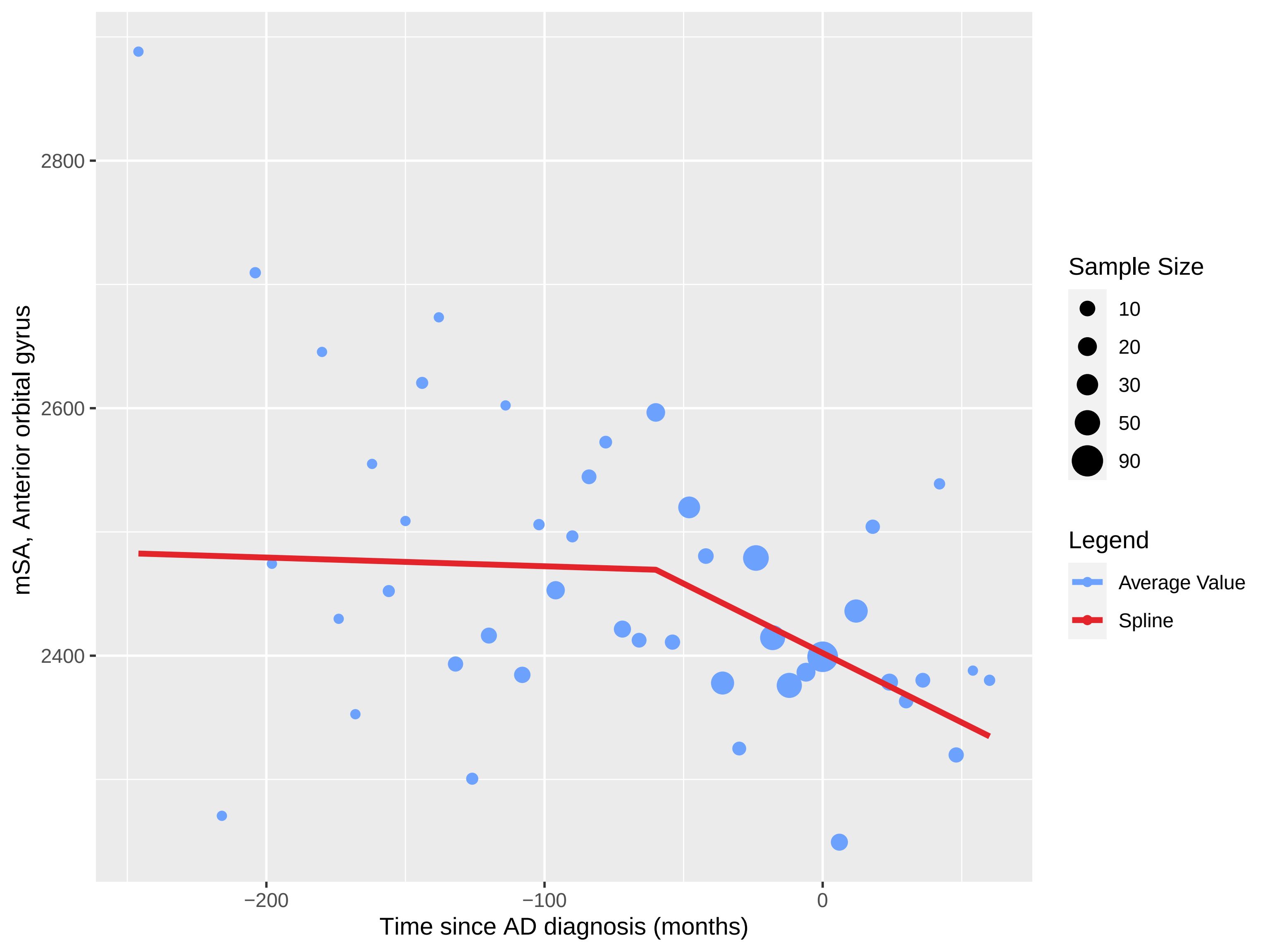

mSA, Anterior orbital gyrus
2800
2600
2400
−200
−100
0
Time since AD diagnosis (months)
Sample Size
10
20
30
50
90
Legend
Average Value
Spline

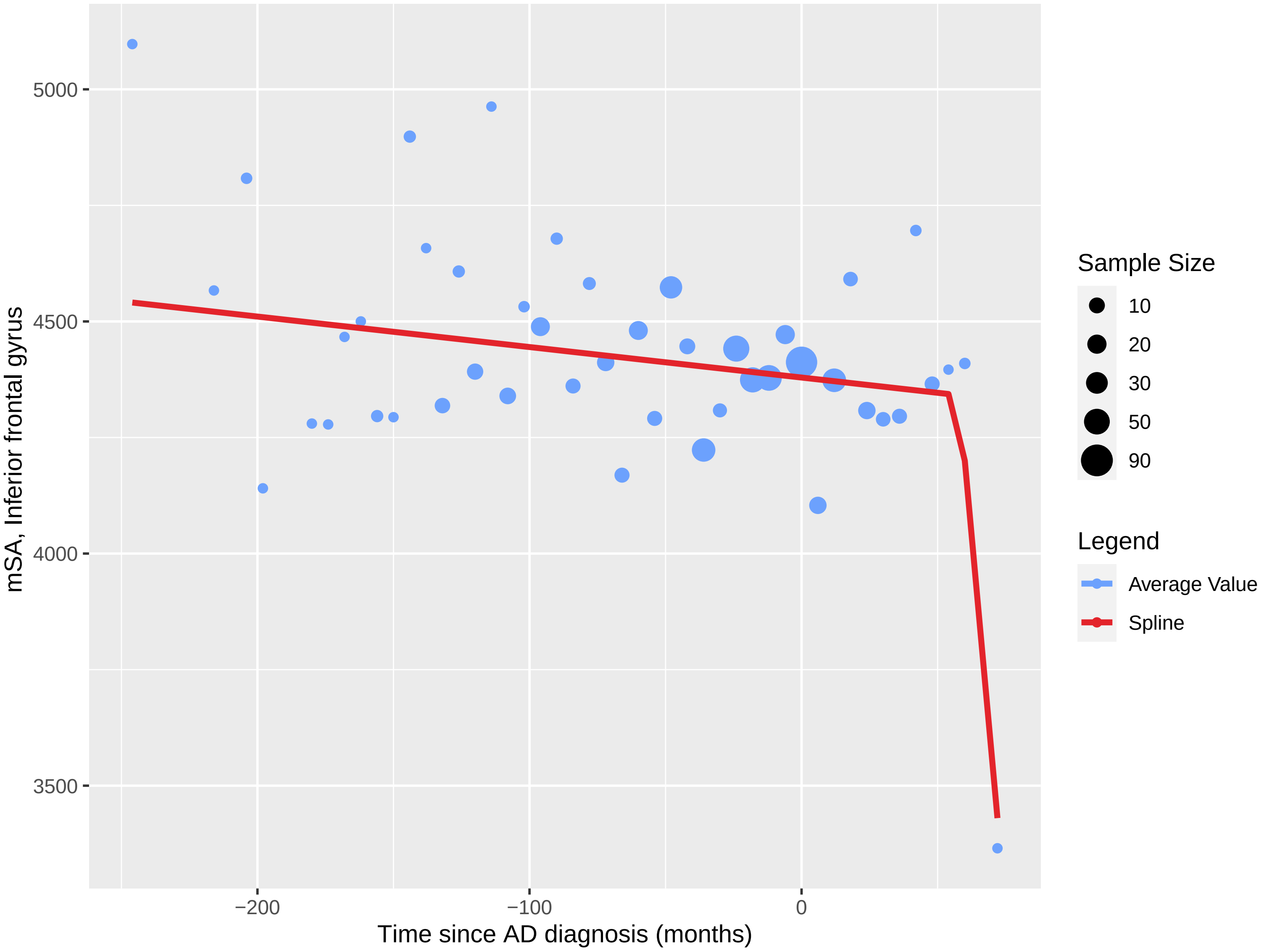

5000
4500
4000
3500
−200
−100
0
mSA, Inferior frontal gyrus
Time since AD diagnosis (months)
Sample Size
10
20
30
50
90
Legend
Average Value
Spline

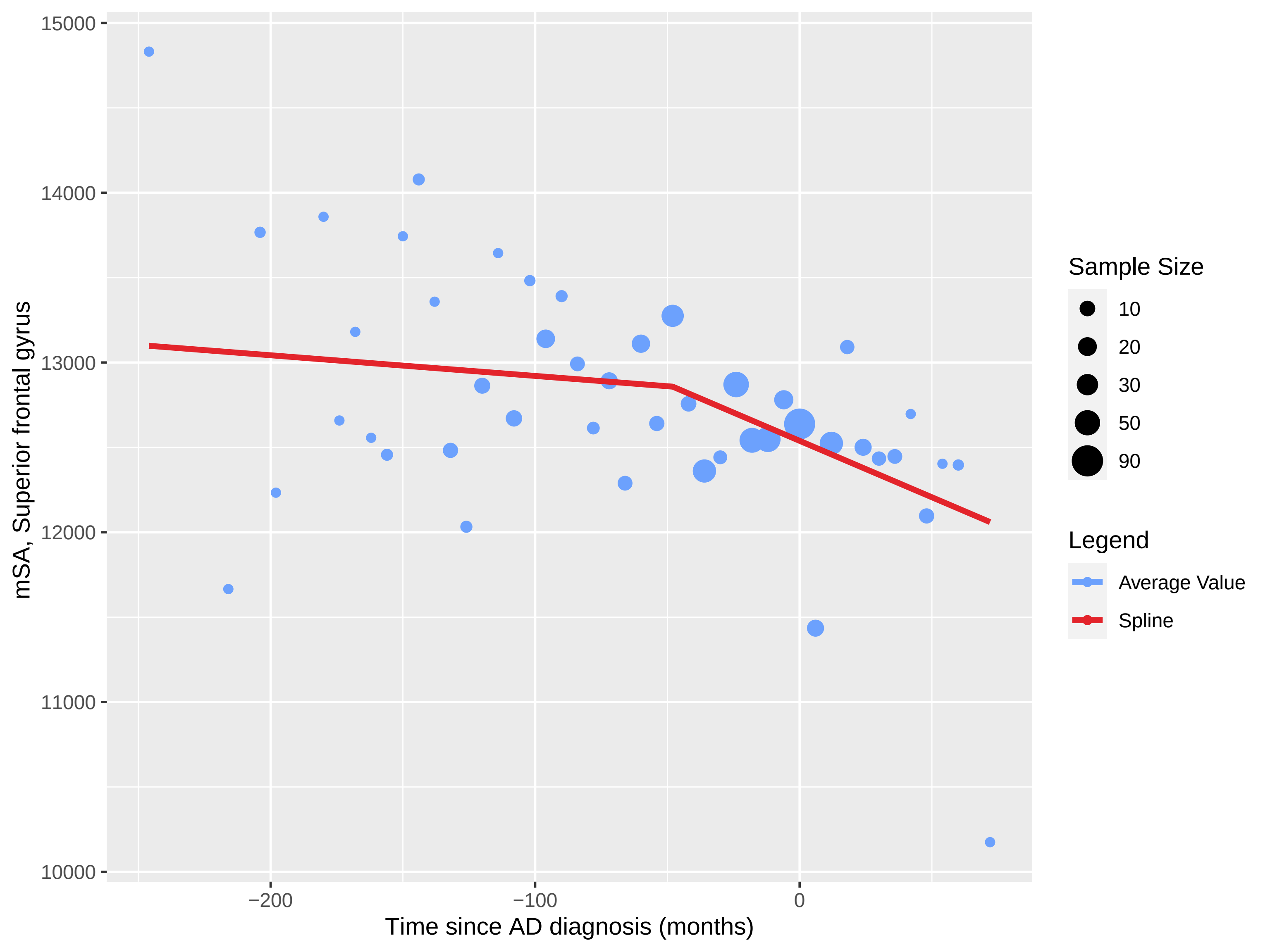

15000
14000
13000
12000
11000
10000
mSA, Superior frontal gyrus
−200
−100
0
Time since AD diagnosis (months)
Sample Size
10
20
30
50
90
Legend
Average Value
Spline

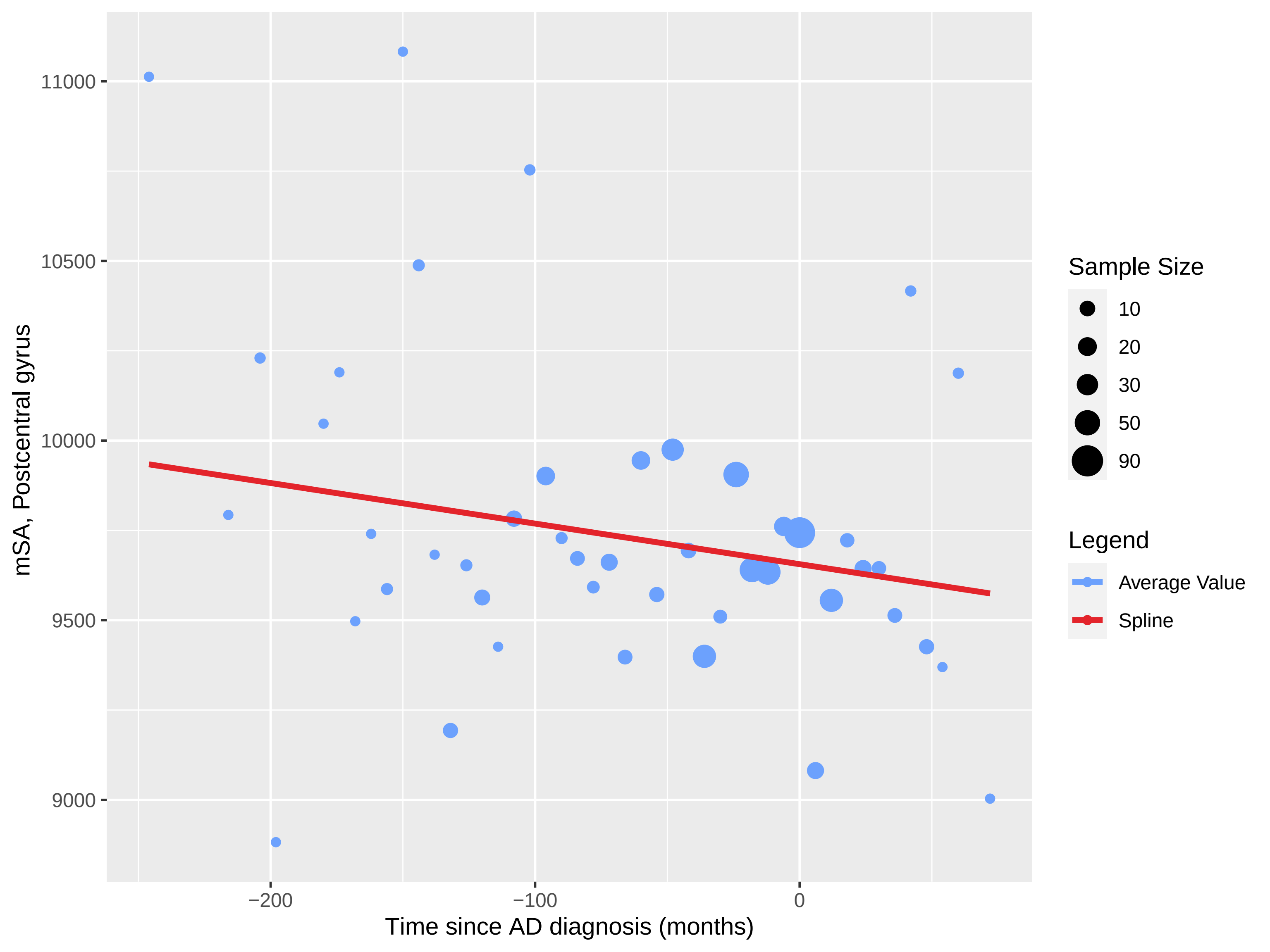

11000
10500
10000
9500
9000
−200
−100
0
mSA, Postcentral gyrus
Time since AD diagnosis (months)
Sample Size
10
20
30
50
90
Legend
Average Value
Spline

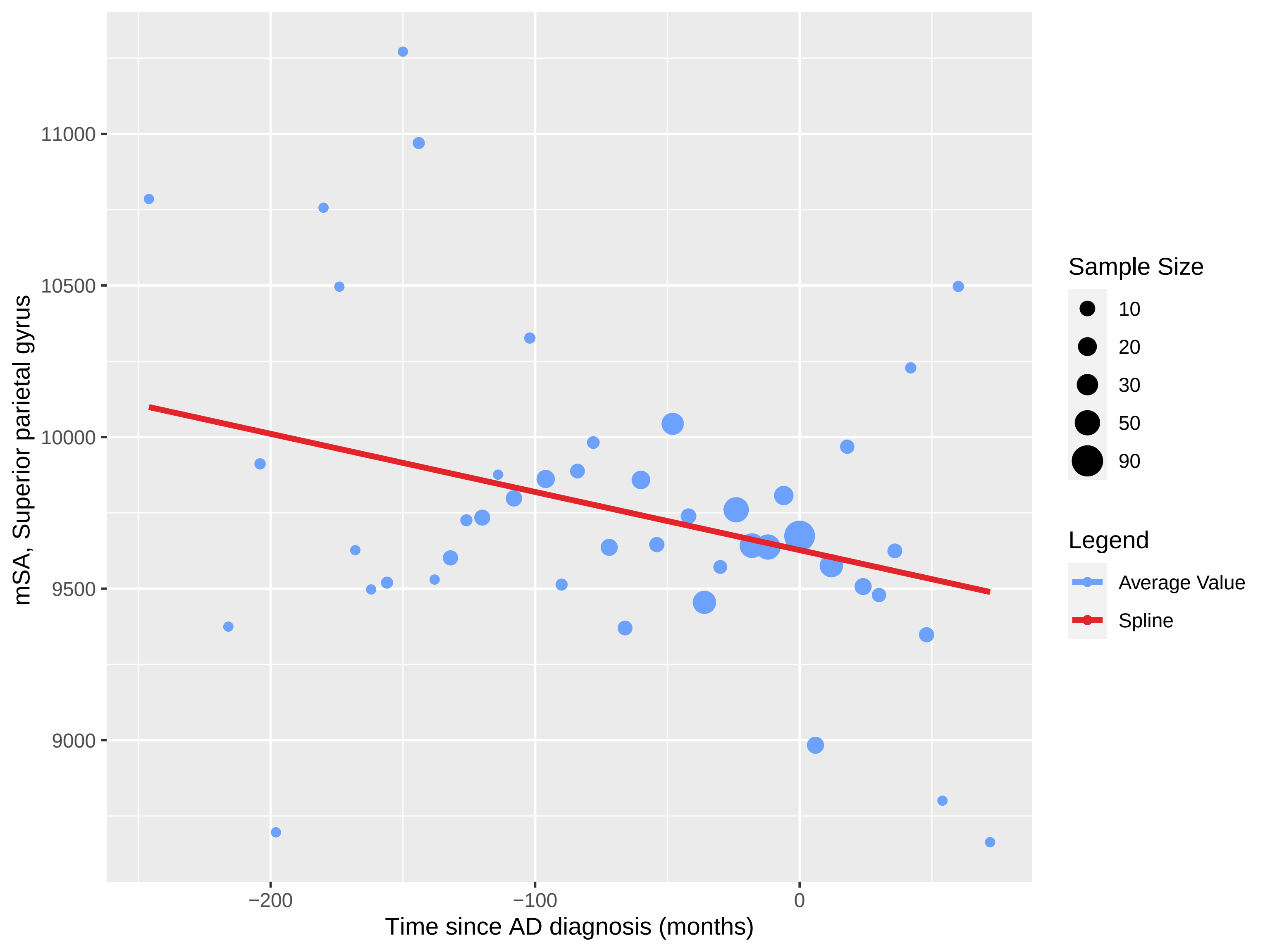

mSA, Superior parietal gyrus
11000
10500
10000
9500
9000
−200
−100
0
Time since AD diagnosis (months)
Sample Size
10
20
30
50
90
Legend
Average Value
Spline

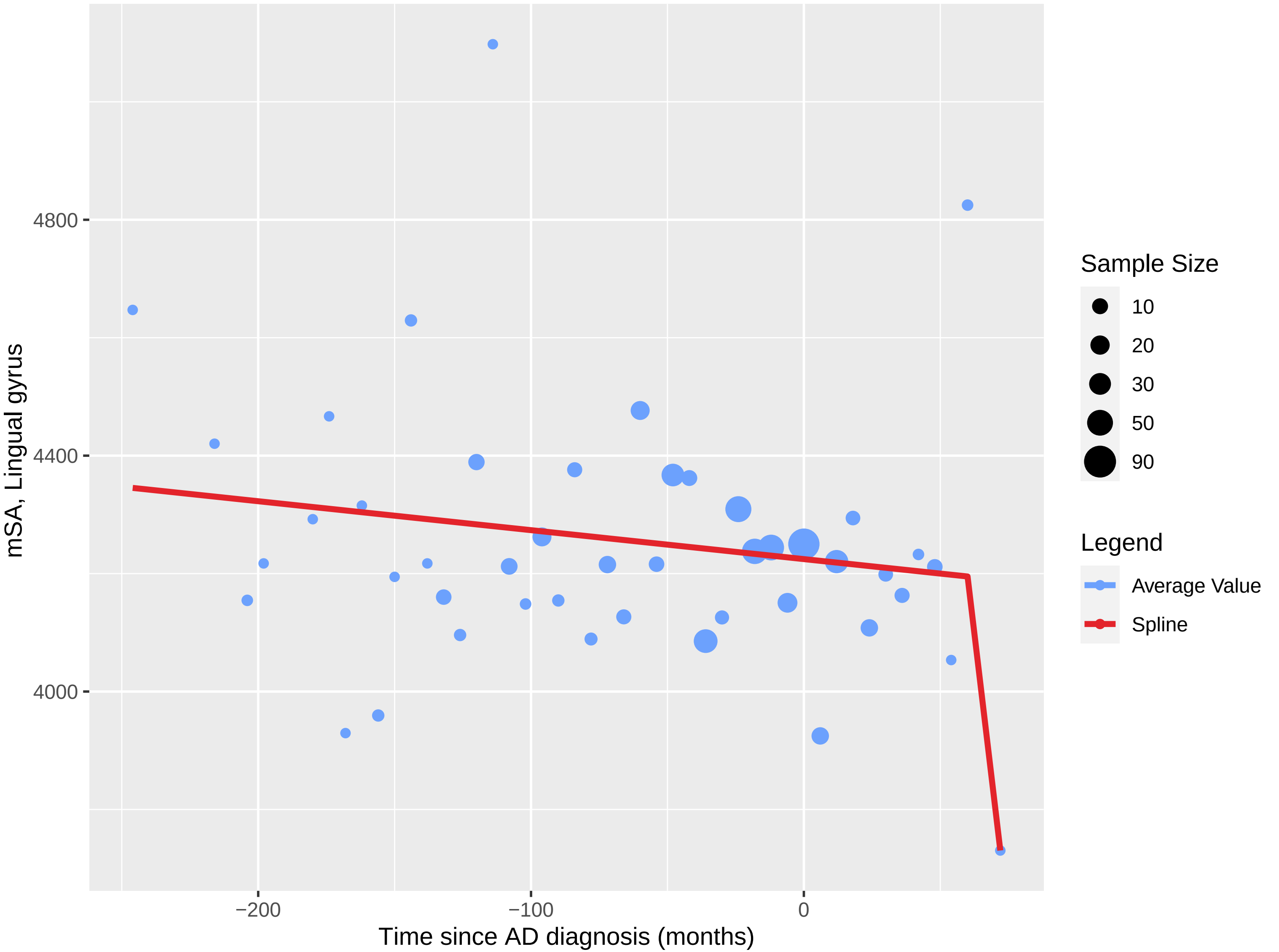

4800
4400
4000
mSA, Lingual gyrus
−200
−100
0
Time since AD diagnosis (months)
Sample Size
10
20
30
50
90
Legend
Average Value
Spline

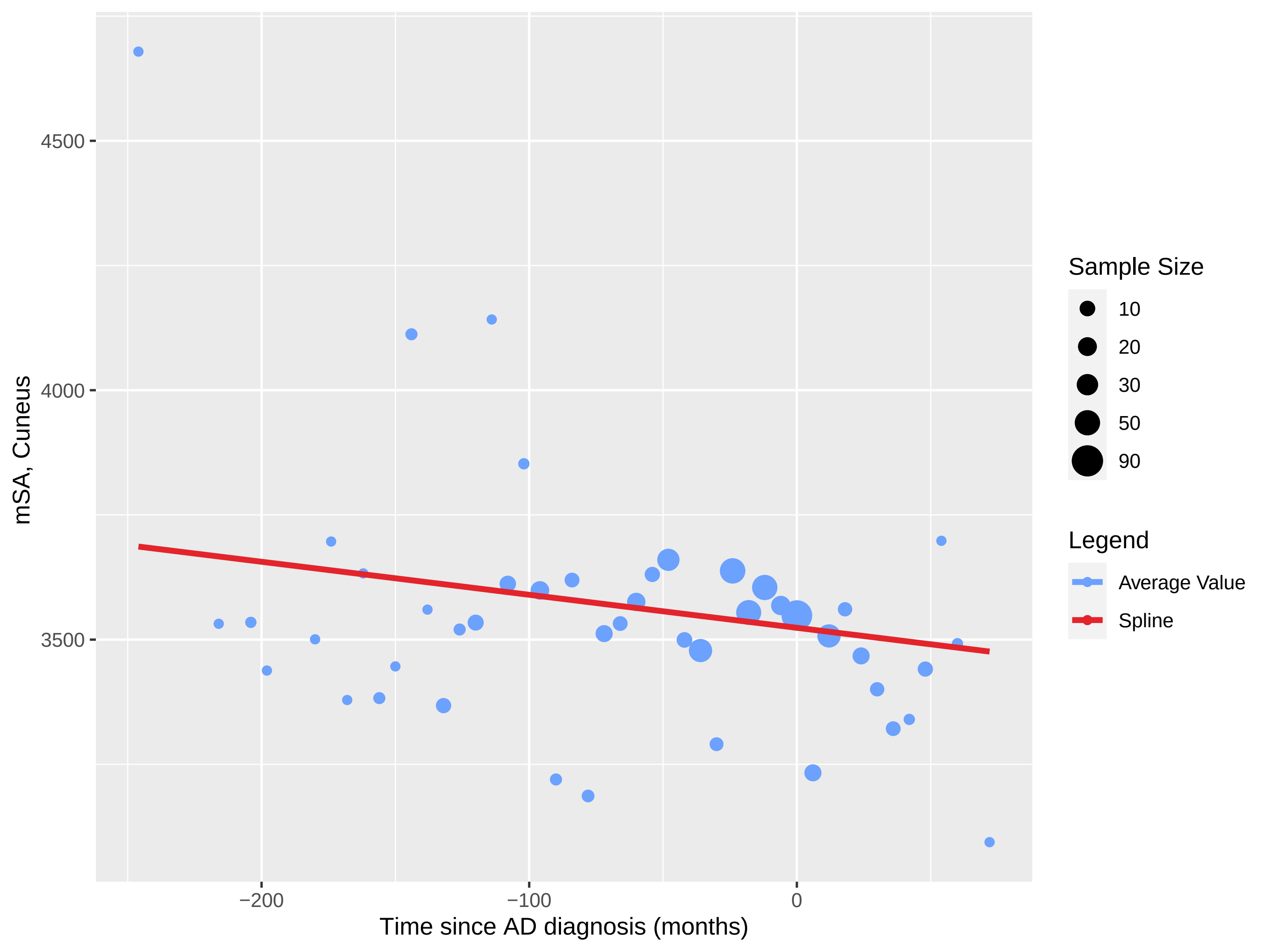

mSA, Cuneus
4500
4000
3500
−200
−100
0
Time since AD diagnosis (months)
Sample Size
10
20
30
50
90
Legend
Average Value
Spline

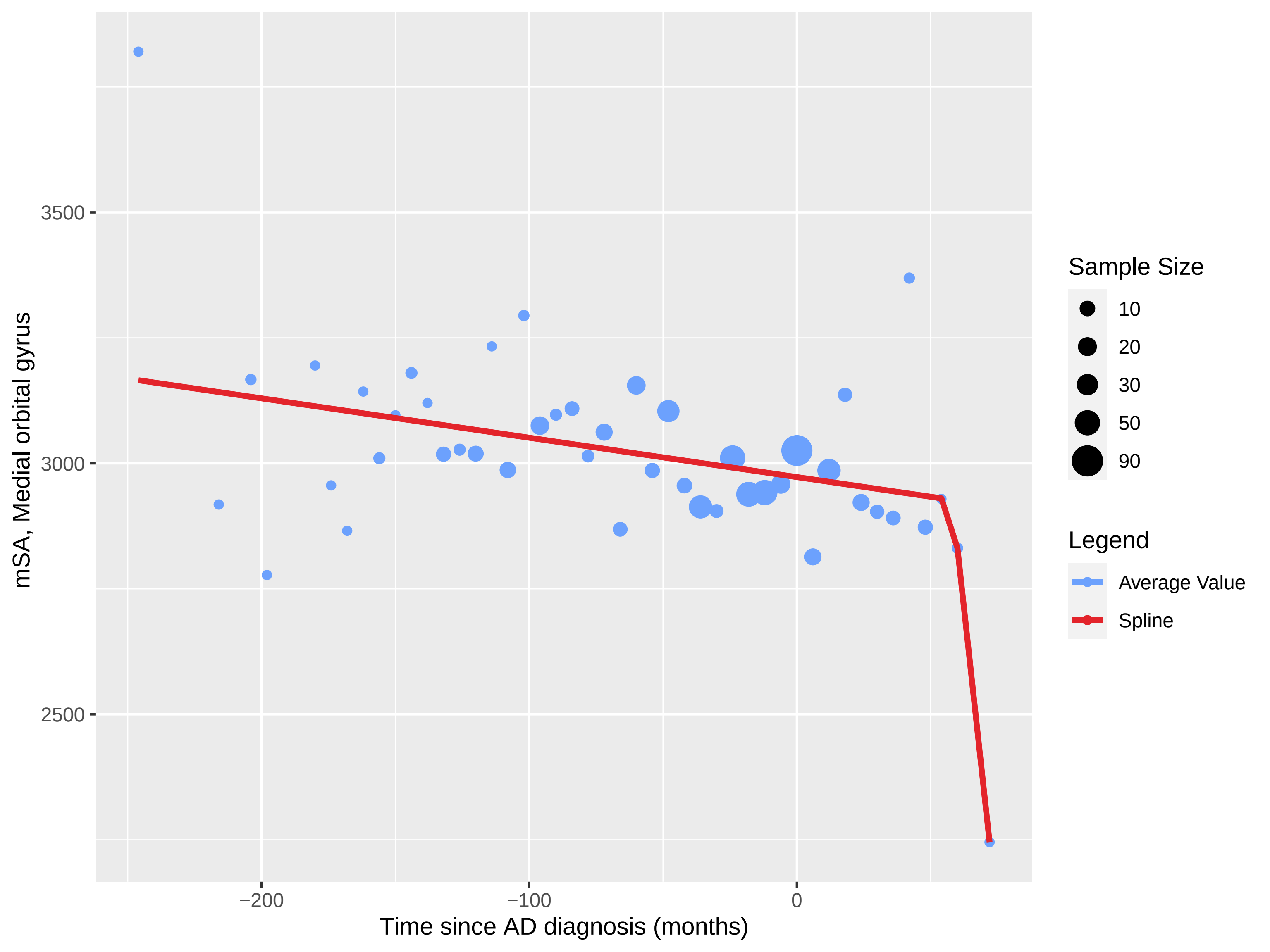

mSA, Medial orbital gyrus
3500
3000
2500
−200
−100
0
Time since AD diagnosis (months)
Sample Size
10
20
30
50
90
Legend
Average Value
Spline

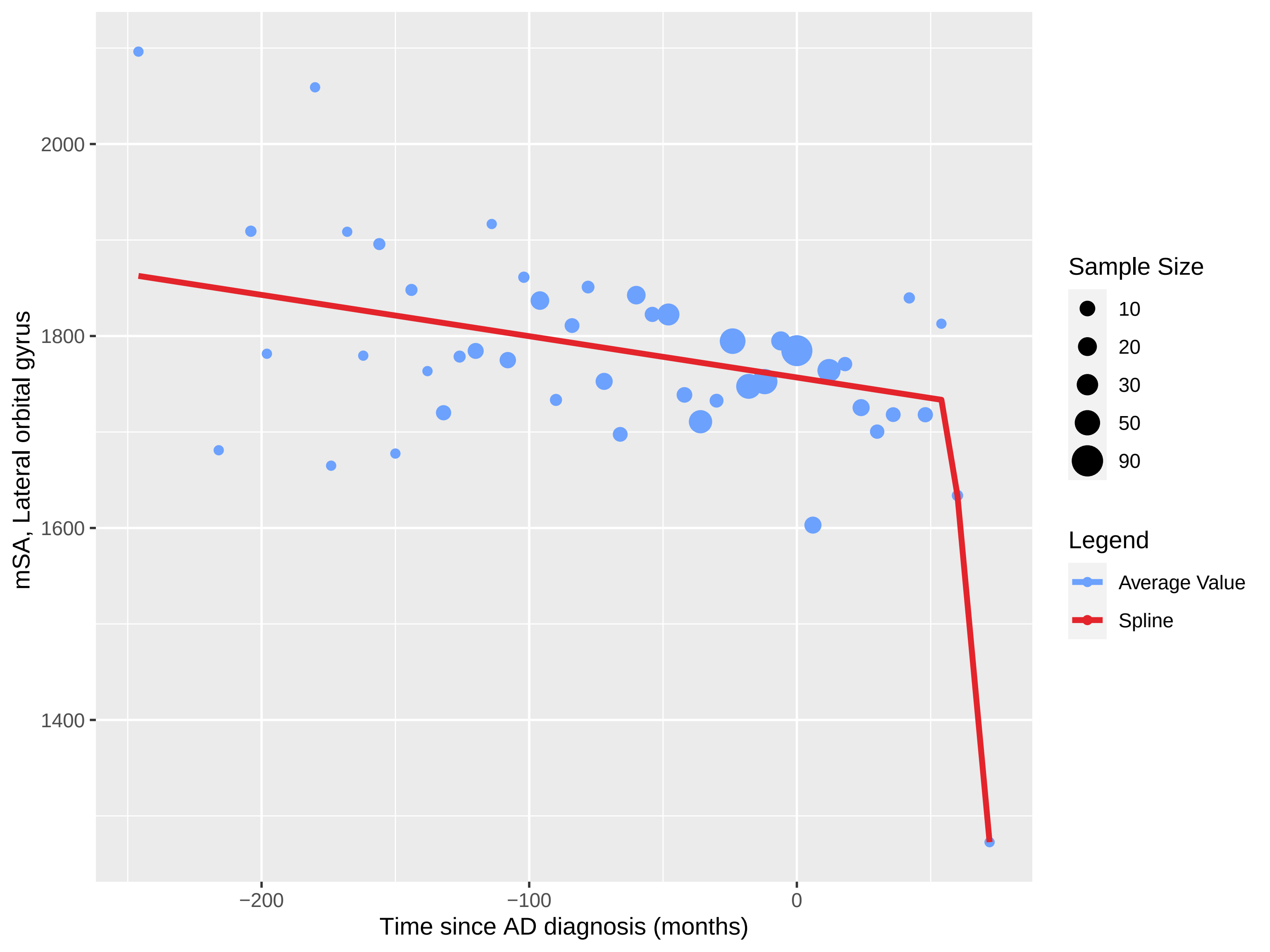

mSA, Lateral orbital gyrus
2000
1800
1600
1400
−200
−100
0
Time since AD diagnosis (months)
Sample Size
10
20
30
50
90
Legend
Average Value
Spline

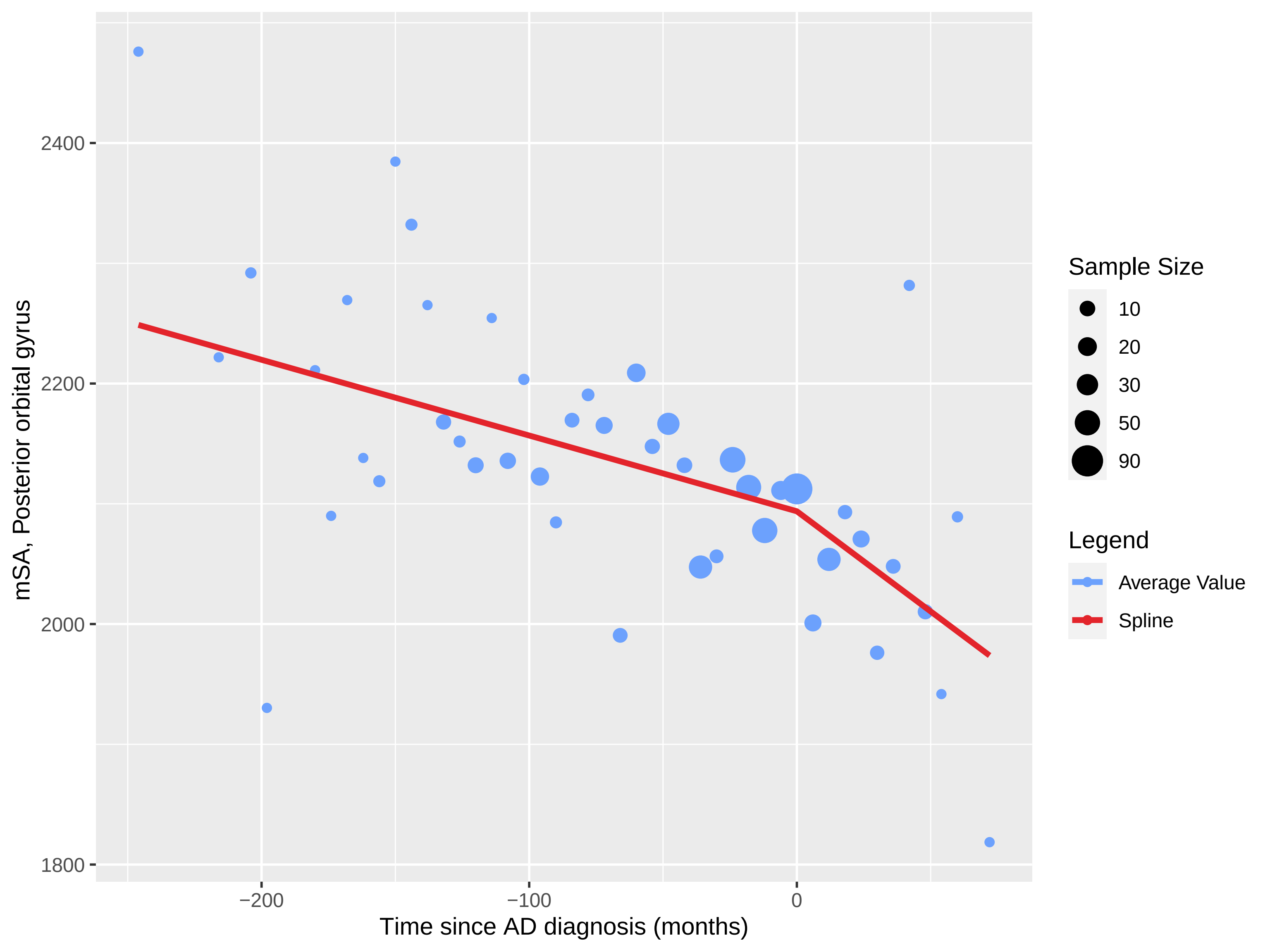
2400
2200
2000
1800
−200
−100
0
Time since AD diagnosis (months)
mSA, Posterior orbital gyrus
Sample Size
10
20
30
50
90
Legend
Average Value
Spline

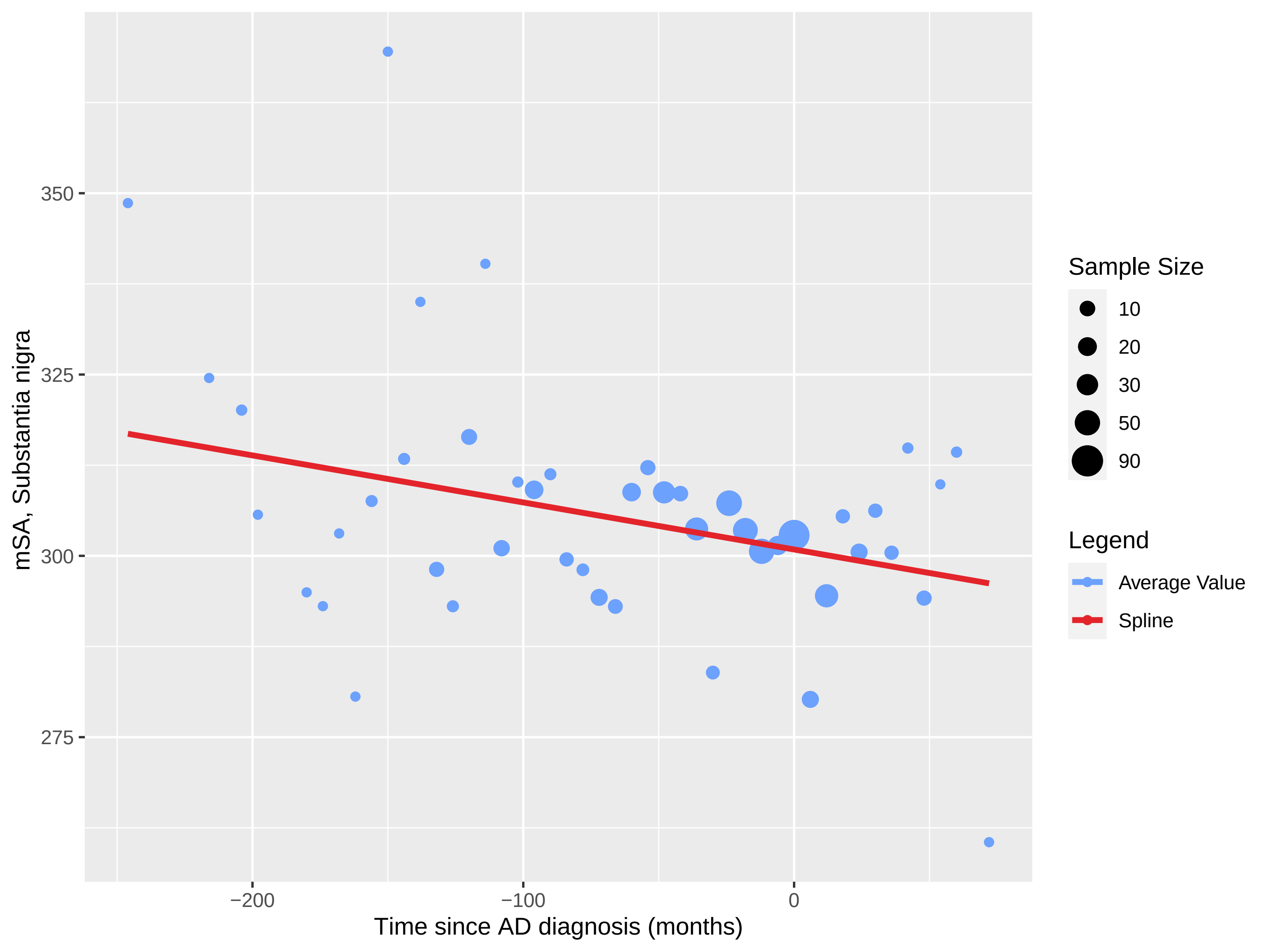

350
325
300
275
mSA, Substantia nigra
−200
−100
0
Time since AD diagnosis (months)
Sample Size
10
20
30
50
90
Legend
Average Value
Spline

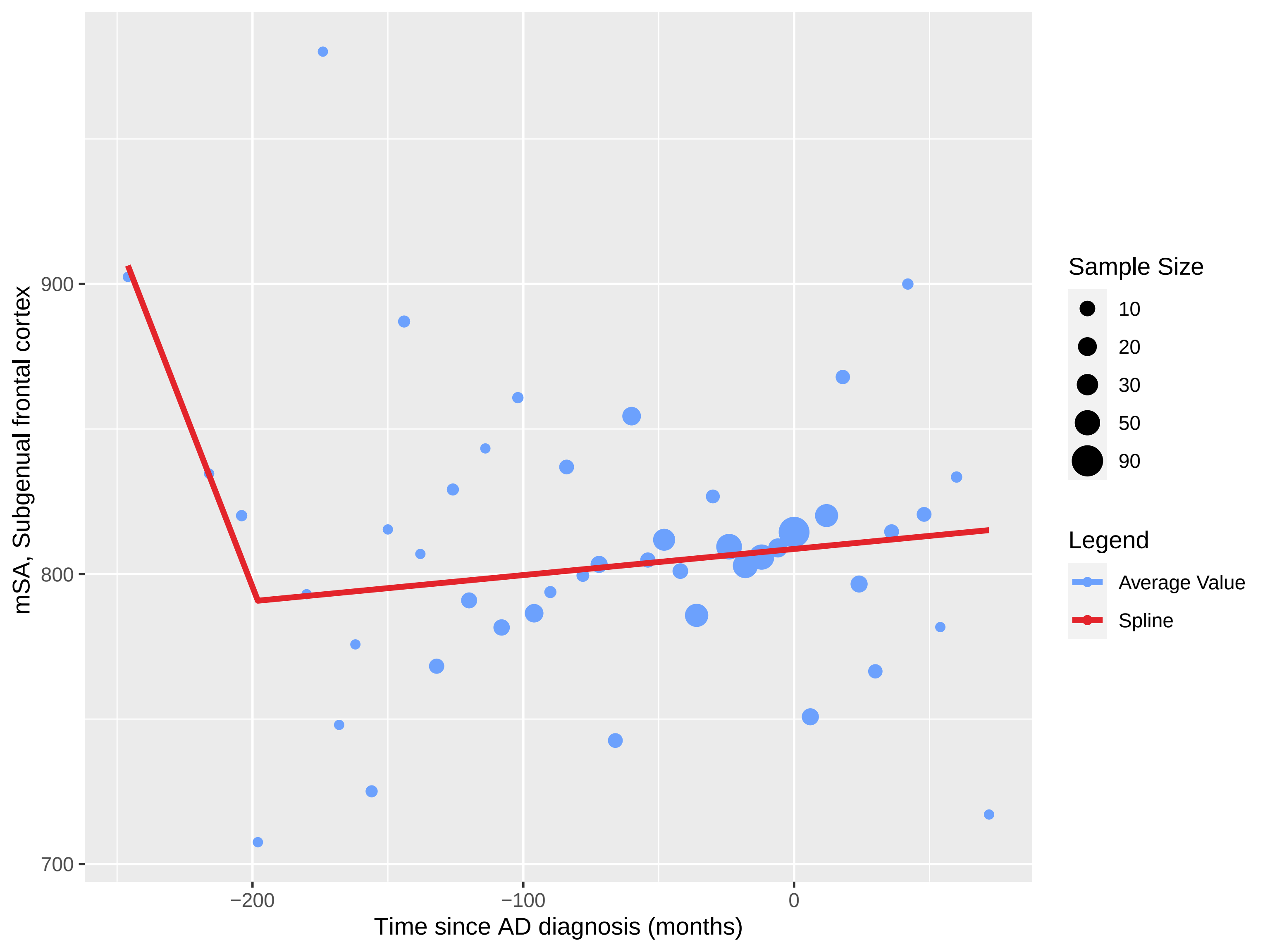

mSA, Subgenual frontal cortex
900
800
700
−200
−100
0
Time since AD diagnosis (months)
Sample Size
10
20
30
50
90
Legend
Average Value
Spline

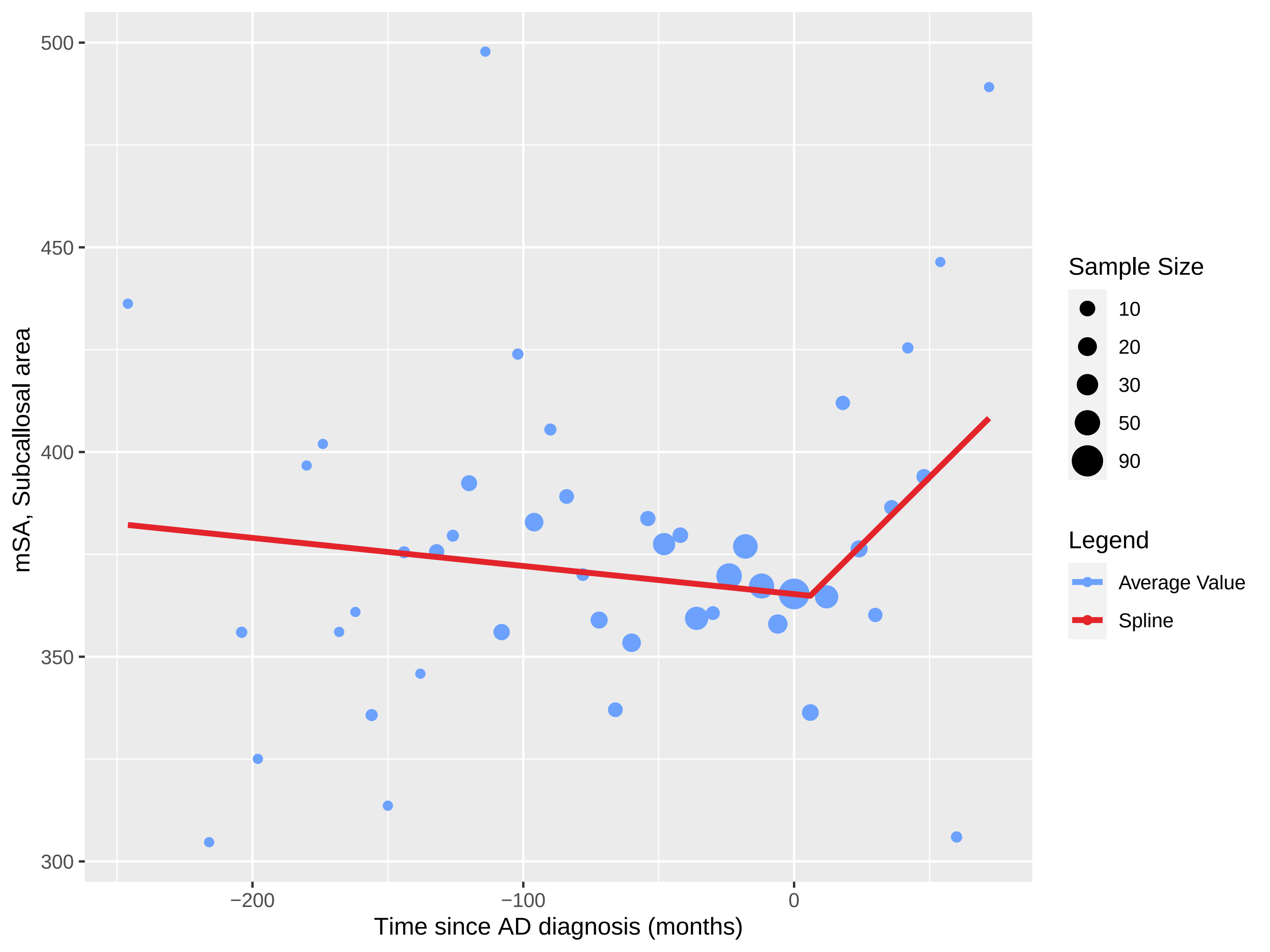

500
450
400
350
300
mSA, Subcallosal area
−200
−100
0
Time since AD diagnosis (months)
Sample Size
10
20
30
50
90
Legend
Average Value
Spline

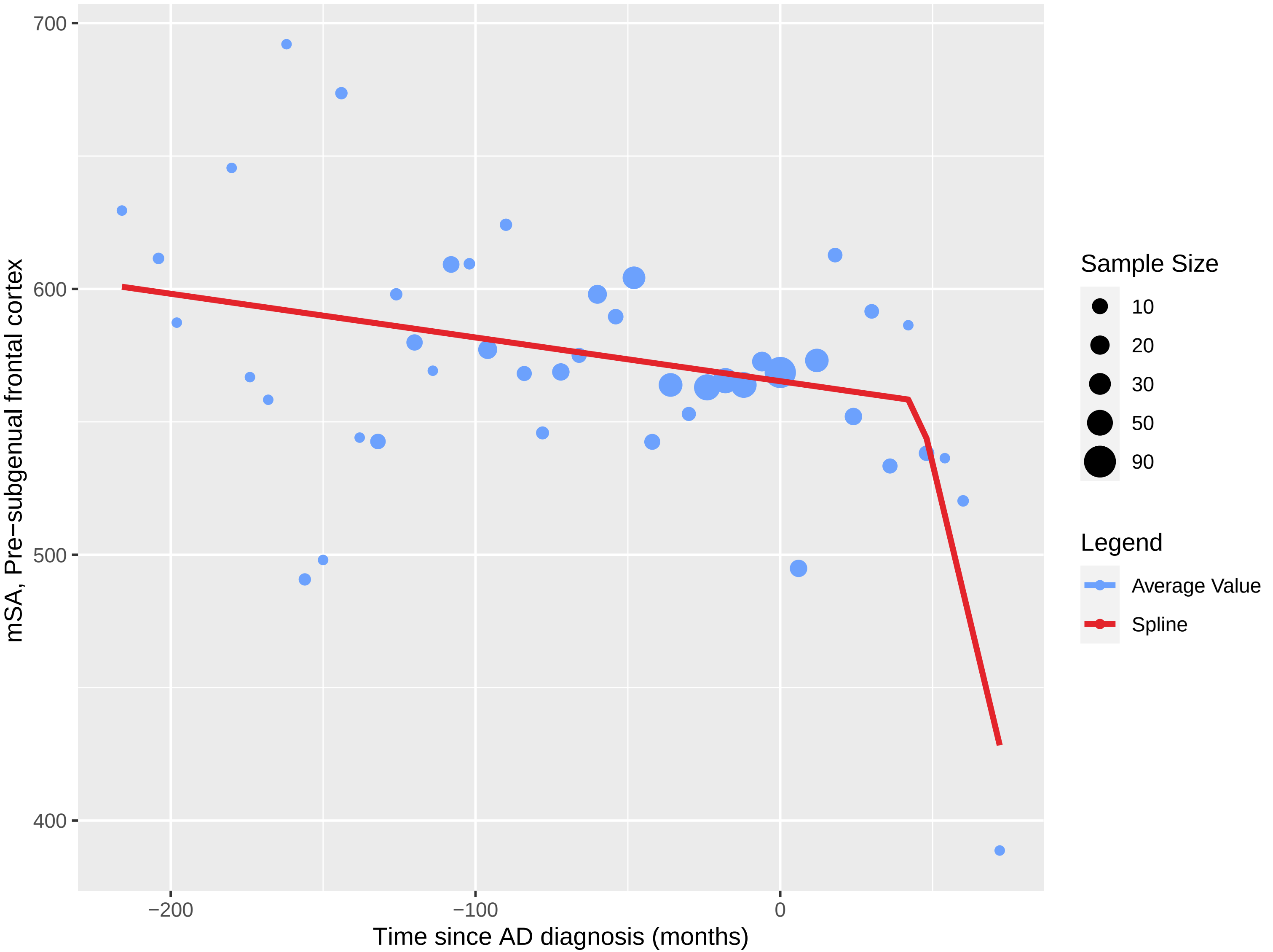

700
600
500
400
mSA, Pre−subgenual frontal cortex
−200
−100
0
Time since AD diagnosis (months)
Sample Size
10
20
30
50
90
Legend
Average Value
Spline

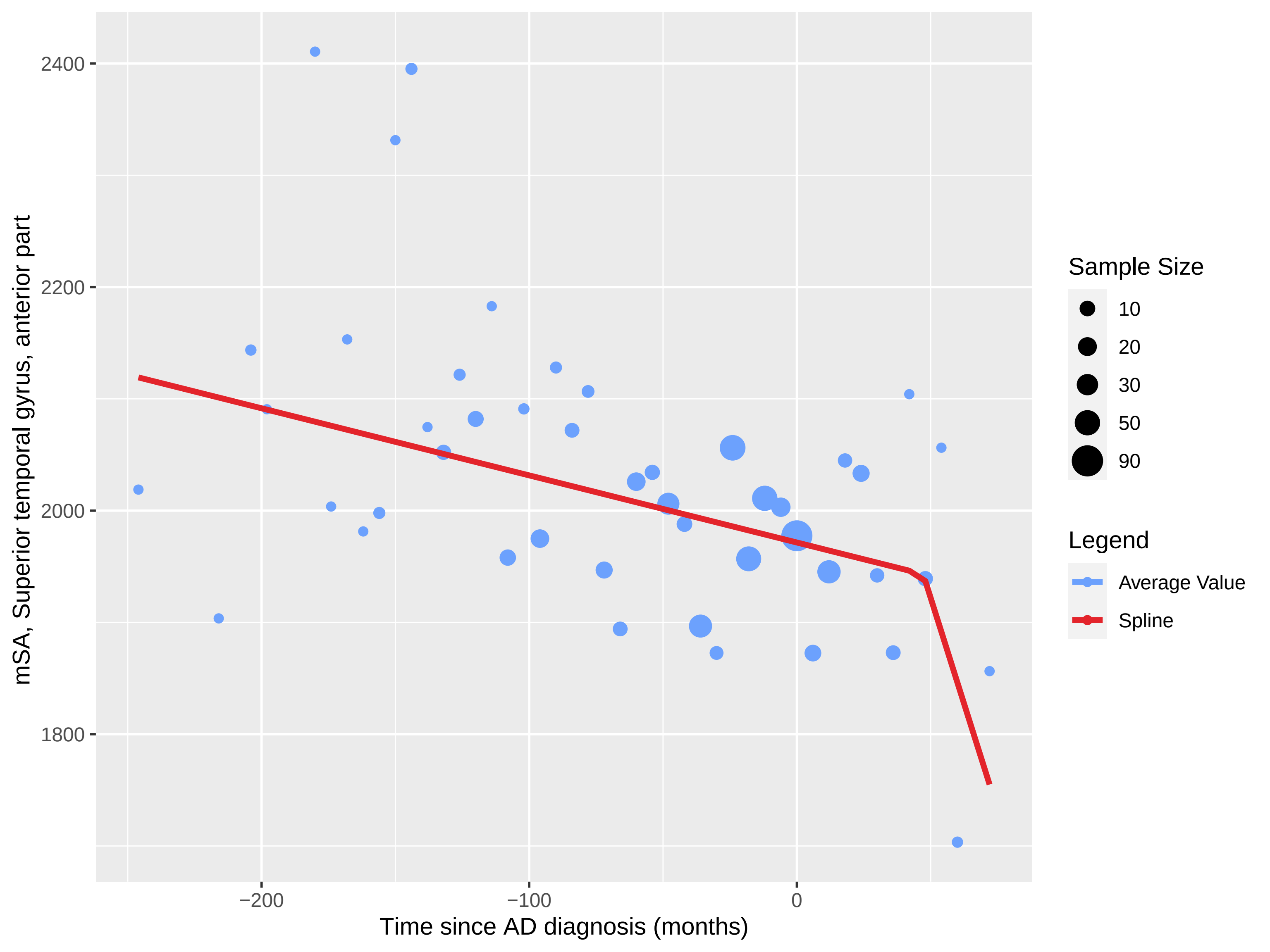

2400
2200
2000
1800
mSA, Superior temporal gyrus, anterior part
−200
−100
0
Time since AD diagnosis (months)
Sample Size
10
20
30
50
90
Legend
Average Value
Spline

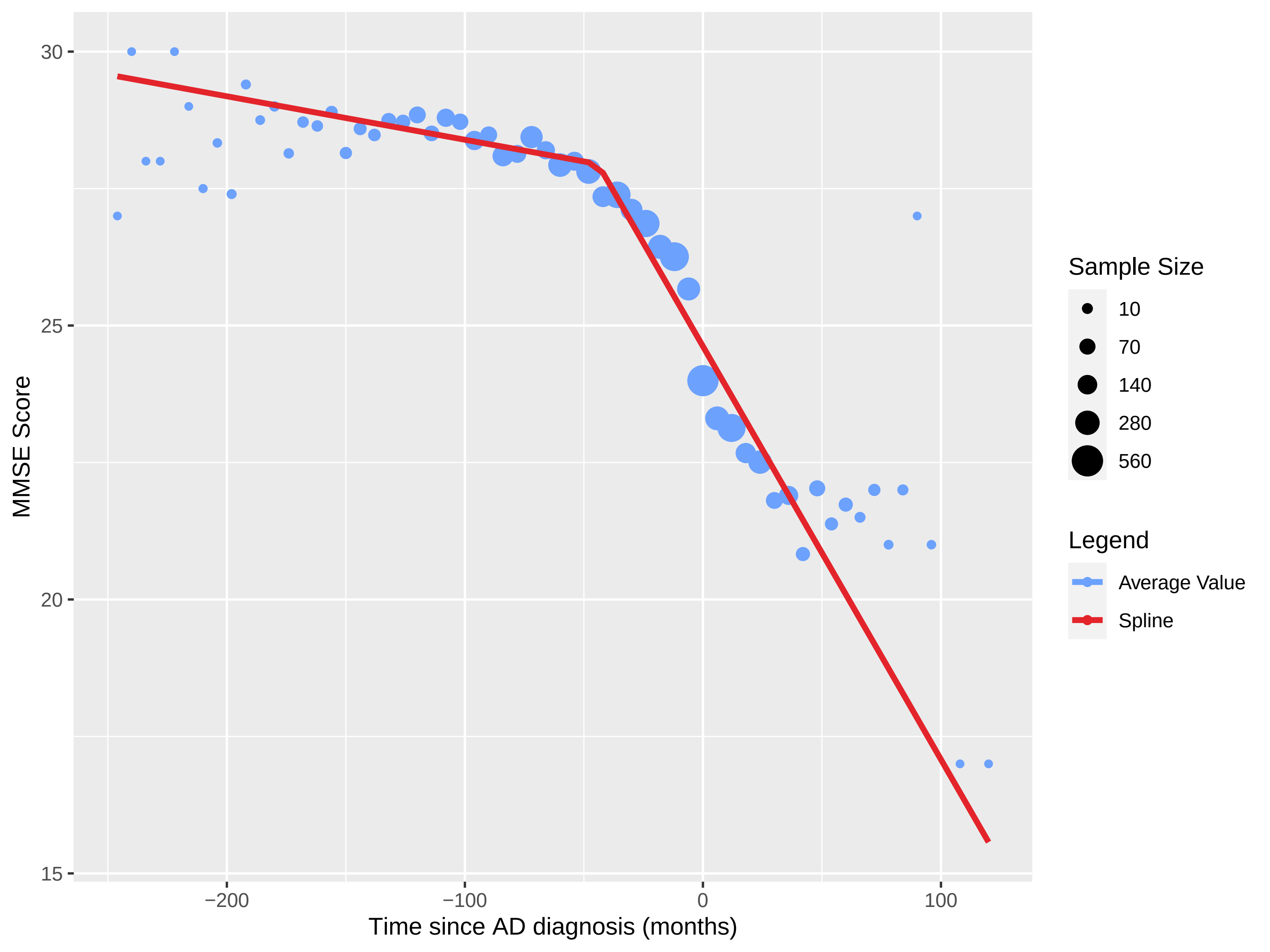
MMSE Score
30
25
20
15
−200
−100
0
100
Time since AD diagnosis (months)
Sample Size
10
70
140
280
560
Legend
Average Value
Spline

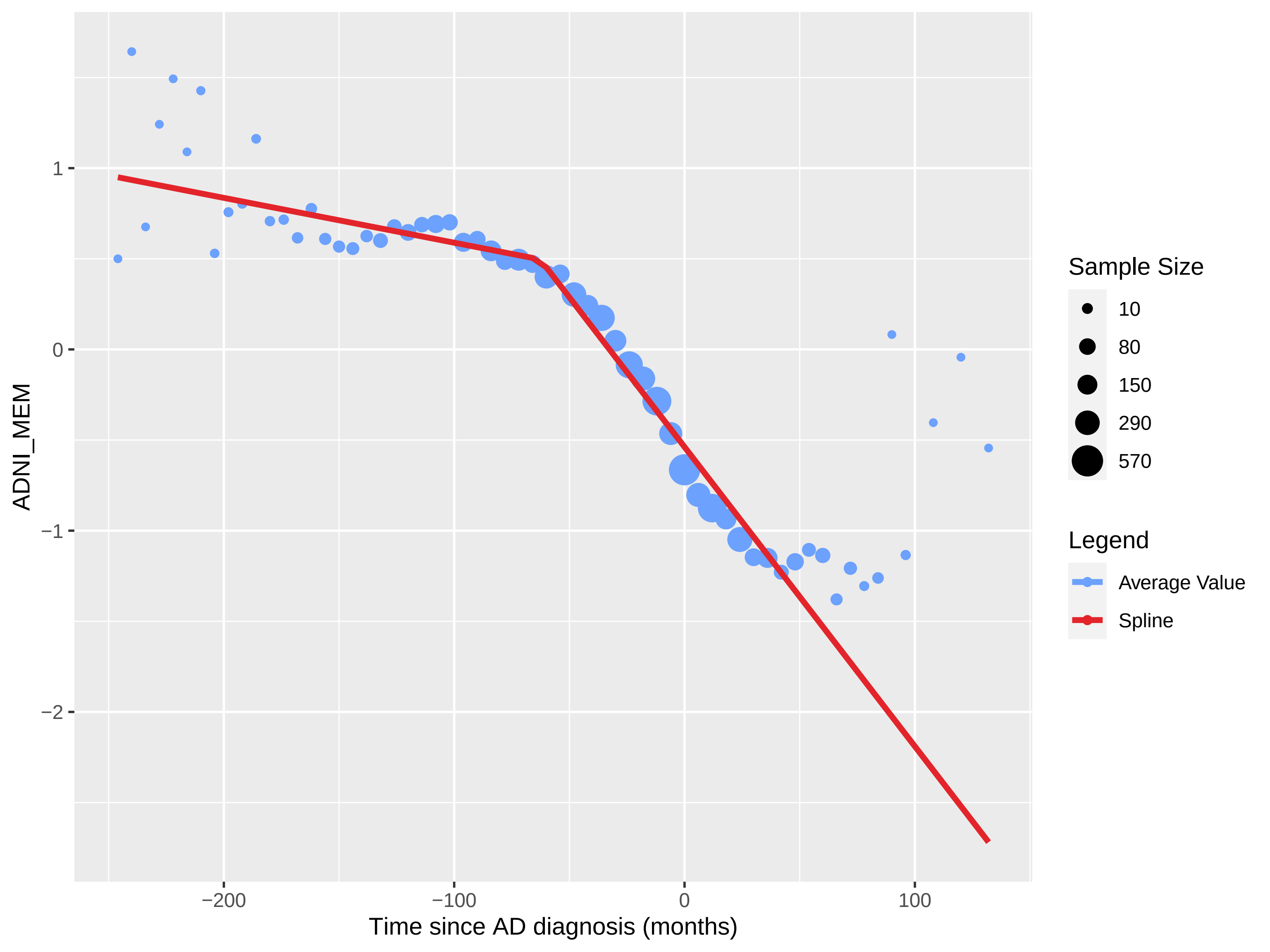
ADNI_MEM
1
0
−1
−2
−200
−100
0
100
Time since AD diagnosis (months)
Sample Size
10
80
150
290
570
Legend
Average Value
Spline

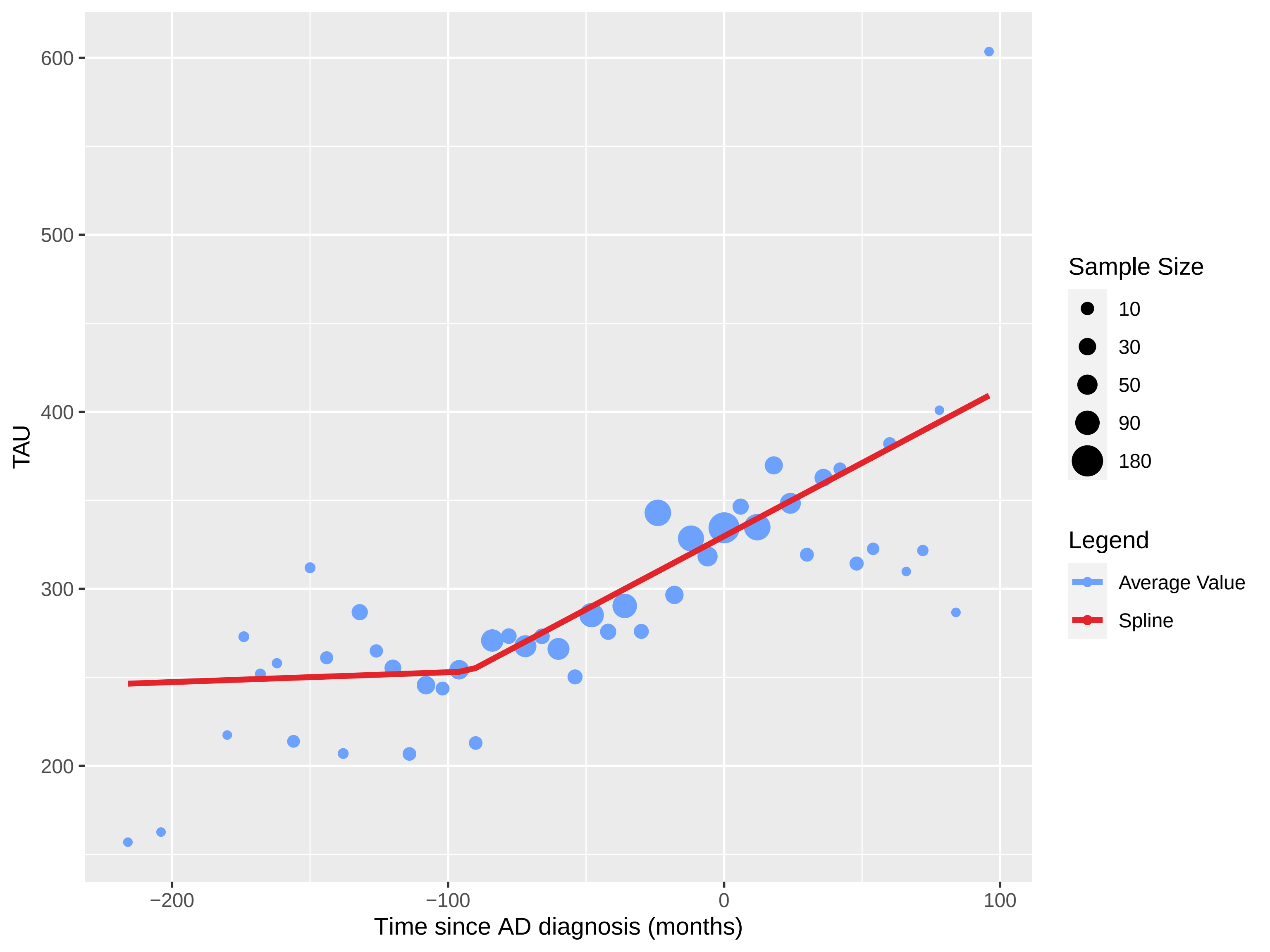

TAU
Time since AD diagnosis (months)
600
500
400
300
200
−200
−100
0
100
Sample Size
10
30
50
90
180
Legend
Average Value
Spline

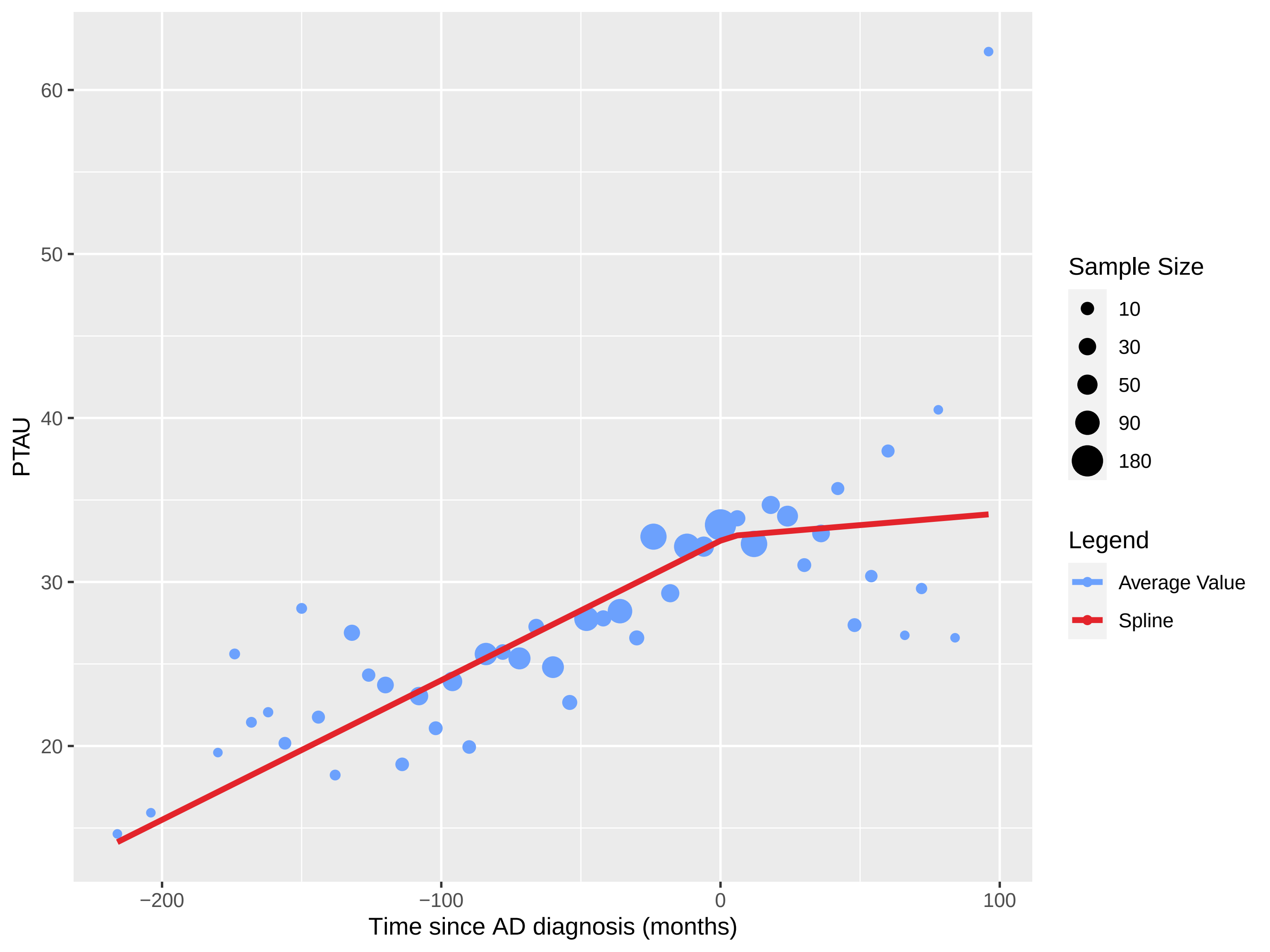

60
50
40
30
20
PTAU
−200
−100
0
100
Time since AD diagnosis (months)
Sample Size
10
30
50
90
180
Legend
Average Value
Spline

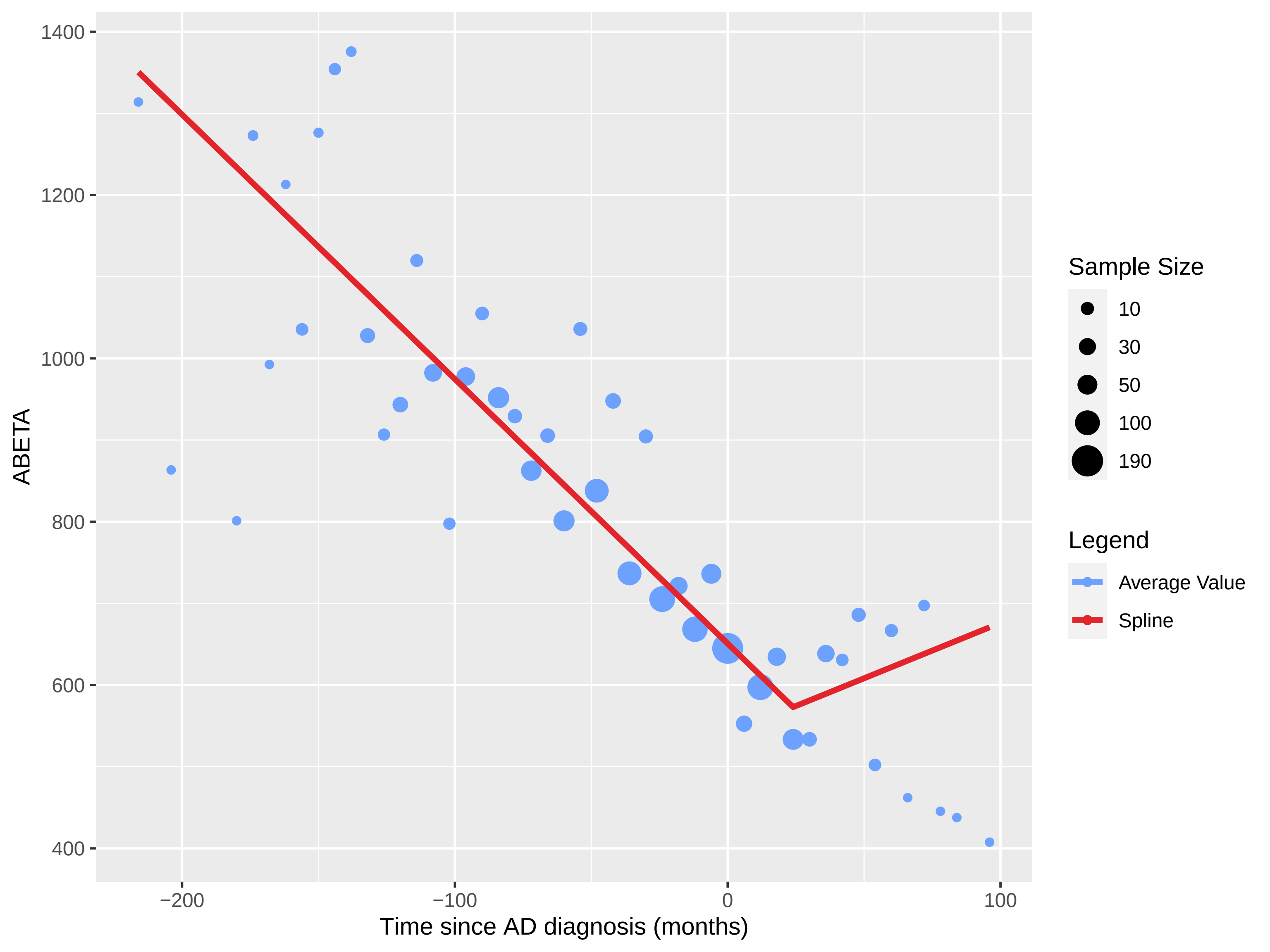

ABETA
1400
1200
1000
800
600
400
−200
−100
0
100
Time since AD diagnosis (months)
Sample Size
10
30
50
100
190
Legend
Average Value
Spline
